# Iron-Catalyzed Miyaura Borylation of Aryl Chlorides and Triflates

**DOI:** 10.1021/acs.orglett.4c04171

**Published:** 2024-12-17

**Authors:** Patrick Daley-Dee, James Clarke, Sebastien Monfette, Robin B. Bedford

**Affiliations:** † School of Chemistry, University of Bristol, Cantock’s Close, Bristol BS8 1TS, U.K.; ‡ Pfizer Chemical Research and Development, Pfizer Inc., Discovery Park House, IPC 533, Sandwich, Kent CT13 9NJ, U.K.; § Pfizer Chemical Research and Development, Pfizer Inc., Groton, Connecticut 06340, United States

## Abstract

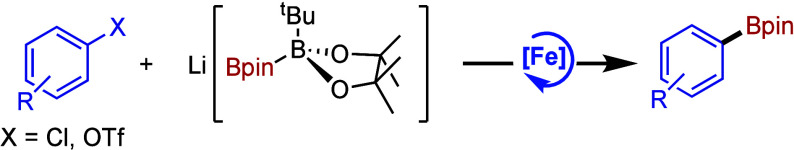

Simple aryl chlorides represent challenging substrates in iron-catalyzed borylation. A combination of Li[B(^t^Bu)pin-Bpin] as the borylating reagent and a catalyst formed in situ from iron(II) triflate and the commercially available N-heterocyclic carbene ligand, IMes, gives significantly improved activity and a much broader scope than previously reported iron-based catalysts. Iron triflate is also a good precatalyst for the borylation of aryl triflates—a previously unreported transformation—and in these cases the IMes ligand is not required.

Organoboronic esters are indispensable reagents in organic synthesis that are applied to Suzuki–Miyaura couplings and numerous other applications.^1^ Traditional methods of synthesis include transmetalation of organolithiums or Grignard reagents^2,3^ or hydroboration and subsequent derivatization of the products.^4^ The former suffers from limited functional group tolerance due to the nucleophilicity of the organometallic reagents, while the latter requires suitable unsaturated starting materials and can be limited by regioselectivity.^5^ Catalytic C–H borylation is an attractive alternative^6−9^ but can also suffer from regioselectivity issues. By contrast, the transition-metal-catalyzed borylation of organohalides (Scheme 1), first described by Miyaura,^10^ has proven to be a powerful method for the synthesis of boronic esters, often boasting milder reaction conditions and higher selectivity, and can even be applied to telescoped one-pot coupling processes.^11,12^

**Scheme 1:**
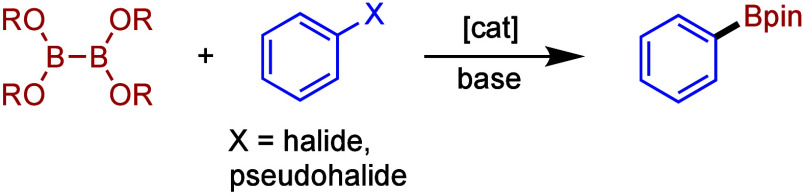
Miyaura Borylation of Aryl Halides and Triflates

While the Miyaura borylation of aryl electrophiles typically employes palladium-based catalysts,^13−16^ those based on more Earth-abundant transition metals such as copper,^17−19^ nickel,^20−25^ cobalt,^26,27^ and Zn^28,29^ have recently been shown to be effective. Of all the transition metals that could be exploited, iron is the most attractive target: it is highly abundant, it is inexpensive, and its production has a far lower carbon footprint than palladium’s (1.5 vs 3,880 kg CO_2_-eq/kg).^30^ Additionally, it has relatively low toxicity, making it better suited to late-stage pharmaceutical synthesis in which trace metal impurities often require additional removal procedures.^31^ Notably the arylB(pin) products themselves are of immediate use in the recently reported iron-catalyzed Suzuki biaryl coupling reaction,^32^ meaning both steps could potentially be catalyzed by iron.

The iron-catalyzed borylation of aryl electrophiles was first demonstrated with arene diazonium salts as substrates^33^ and has more recently been extended to aryl pyridone ethers,^34^ aryl carboxylic acids,^35^ and aryl fluorides.^36,37^ With regard to the iron-catalyzed borylation of aryl halide substrates, we reported the first examples using Li[B(^t^Bu)pin-Bpin], **1**, as the borylating reagent.^38^ In comparison with the results obtained with allyl and alkyl halides, the yields obtained with the limited number of aryl halides tested were poor. Subsequently, Nakamura disclosed the borylation of aryl chlorides in the presence of potassium *tert*-butoxide base, catalyzed by the readily available precatalyst Fe(acac)_3_.^39^ While good results were obtained with fused-ring and 4-aryl-substituted aryl substrates, yields were comparatively poor for simpler aryl substrates.^40^

Herein, we report an improved method for the iron-catalyzed borylation of simple aryl chlorides as well as the first example of iron-catalyzed borylation of aryl triflates.

We began by reoptimizing the reaction we reported previously,^38^ using chlorobenzene **2a** as the substrate, **1** as the borylating reagent, and [FeCl_2_(dppe)] as the precatalyst. Note, **1** is prepared using ^t^BuLi: *Caution! tert-Butyllithium is extremely pyrophoric. It must be handled using proper needle and syringe techniques*. Modifications such as decreasing the reaction volume from 8 to 2 mL of THF, increasing the temperature from 40 to 60 °C while performing the reactions in 7 mL screw-top vials, rather than in a Schlenk tube, and heating with an aluminum block inside the glovebox gave a modest improvement in yield from 5 to 18% of desired product **3a**. Conversely, no improvement in yield was obtained on increasing the reaction time beyond 1 h.

Under these conditions, we screened a range of catalysts made in situ from FeCl_2_ and a variety of phosphines and N-heterocyclic carbenes (NHCs) (see Table S2). Interestingly, the catalyst formed in situ with dppe gave poorer activity than a control with no added ligand. This proved to be the case with most of the diphosphine ligands explored (Table S2). The ligands that gave comparable or improved yields of **3a** relative to the coligand-free control were selected for further screening against a range of iron salts, and the results of this screen are summarized in Figure 1.

**Figure 1:**
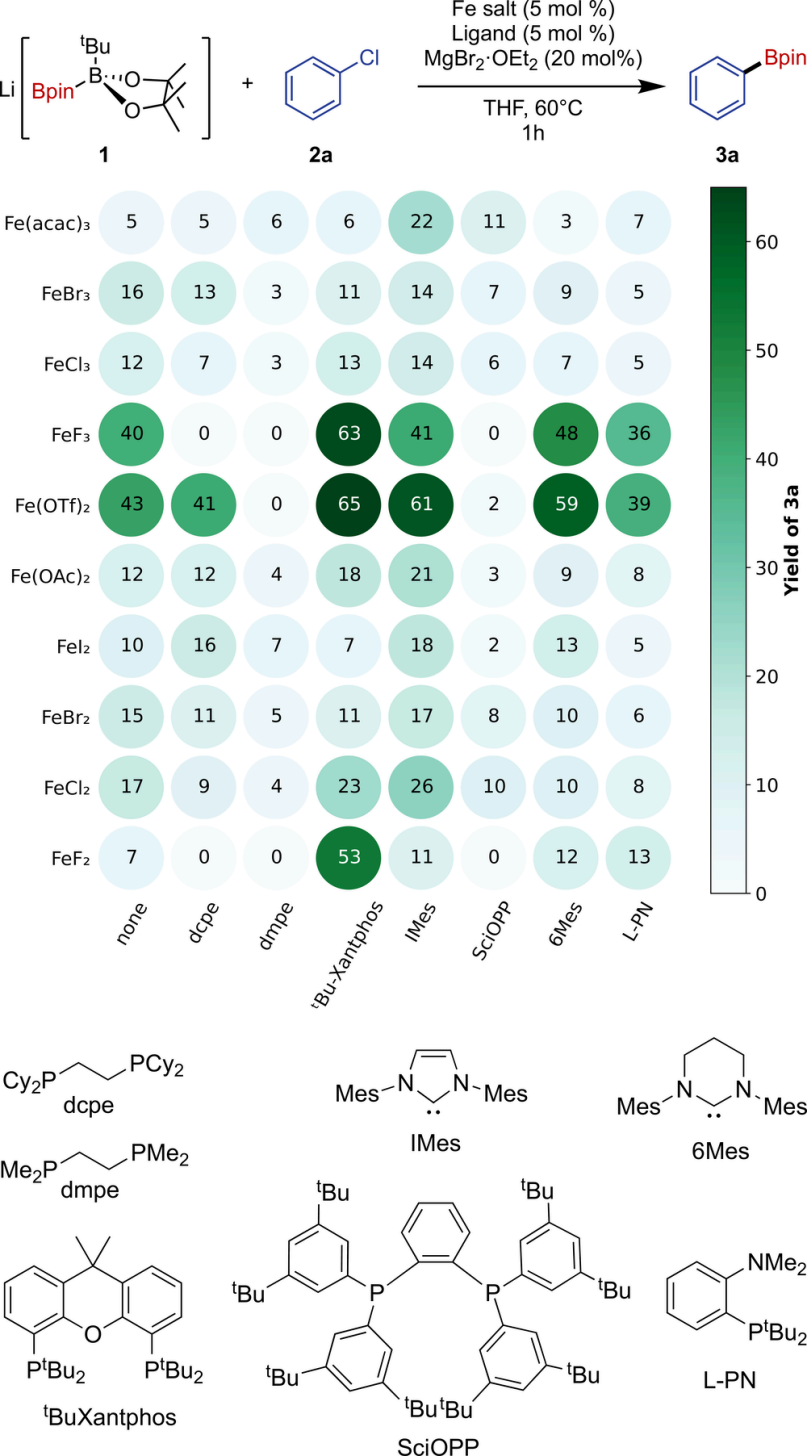
Screening of selected ligands with varying Fe precursors in the Fe-catalyzed borylation of aryl chlorides.

The precatalyst screen revealed Fe(OTf)_2_ and FeF_3_ to be the best iron sources of those tested, while the best performing ligands were the chelating bisphosphine, ^t^BuXantphos, and the N-heterocyclic carbenes IMes and 6Mes. For reasons of cost and availability, a combination of Fe(OTf)_2_ and IMes was chosen for further study. A range of different additives were examined (Table S3), but none improved the 64% yield of **3a**. In some cases in the optimization screenings, we observed the major side product to be benzene, deriving from either hydrodehalogenation of **2a** or protodeborylation of the product **3a**, with only trace amounts (<1%) of biphenyl formed by a homocoupling reaction of **2a**. Subsequent solvent screening (Table S4) showed THF to be optimal. While DMA or 2-MeTHF could also be used, most other solvents tested shut down the reaction. With THF as solvent, we noticed that better yields were obtained when both the ligand and magnesium bromide were added as stock solutions rather than as solids (Table S4).

Tuning the loadings of the reaction components showed the best results were obtained with 1.2 equiv of **1** with respect to **2a** using 3 mol % of Fe(OTf)_2_ and 1.5 mol % of IMes. Under these conditions, **3a** was obtained in 70% yield. The rather unusual metal to ligand ratio may indicate the requirement for differently speciated iron intermediates in the catalytic manifold, or it may be, more prosaically, due to solubility issues associated with the iron salt. Attempts to isolate well-defined iron complexes from this mixture have so far proved fruitless.

With the optimized conditions in hand, we next explored the application of the methodology to a range of aryl chloride substrates. Figure 2 summarizes the results where >20% spectroscopic yield of the desired product **3** was obtained, while poorer performing and unsuccessful reactions are summarized in Figure S2.

**Figure 2:**
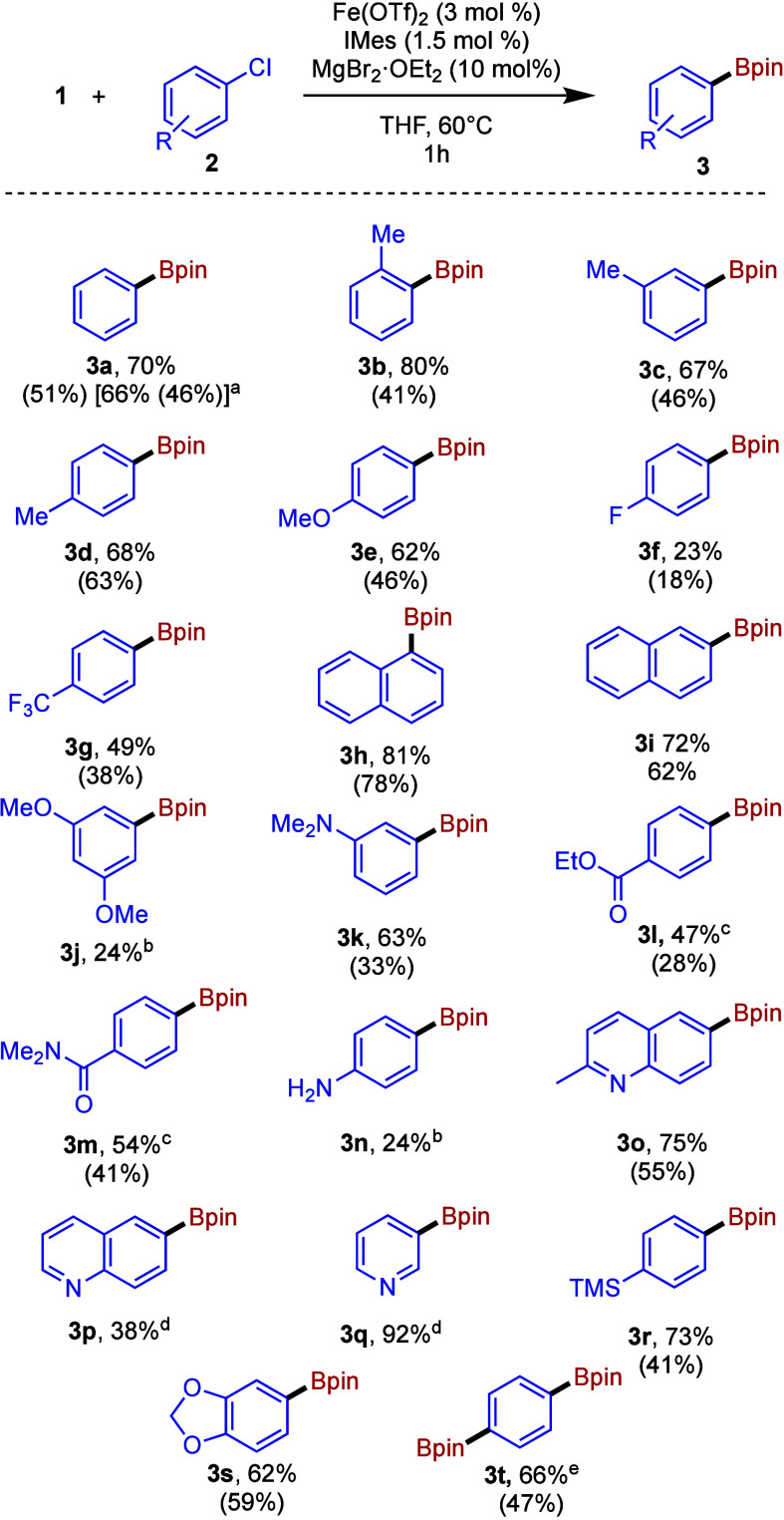
Fe-catalyzed borylation of selected aryl chlorides. Conditions: **1** (0.6 mmol), **2** (0.5 mmol), MgBr_2_·OEt_2_ (0.1 mmol), Fe(OTf)_2_ (0.015 mmol), IMes (0.008 mmol), THF, 60 °C, 1 h. Yields were determined by ^1^H NMR spectroscopy (1,3,5-trimethoxybenzene or mesitylene internal standard). Isolated yields shown in parentheses. ^a^1.0 mmol scale. ^b^Purification was not attempted. ^c^Catalyst loading increased to 5 mol % Fe(OTf)_2_ and 2.5 mol % IMes. ^d^Could not be separated. ^e^2 equiv of **1**.

The reaction performed well with *ortho*-, *meta*-, and *para*-tolyl chlorides (**3b**–**d**). As might be anticipated, both 1- and 2-naphthyl chlorides gave good yields (**3h**,**i**) since substrates such as these with more extensive π-systems have shown a propensity to react particularly well in iron-catalyzed transformations in numerous instances, not least borylation reactions.^34,37,39,41^

Pleasingly, *N*-alkyl anilines (**3k**), esters (**3l**), and amides (**3m**) were well tolerated, as were 6-chloroquinolines and 3-chloropyridine (products **3o**–**q**), the latter giving the highest spectroscopic yield of the substrates tested (92%), although isolating the pure product proved problematic. Interestingly, diborylation of 1,4-dichlorobenzene was readily achieved with 2 equiv of **1**, giving the 1,4-diboronic ester **3t** in 66% yield. Disappointingly, some substrates performed particularly poorly, including 4-fluorochlorobenzene (**2f**) and 1-chloro-3-5-dimethoxybenzene (**2j**), as well as those listed in Figure S2. Comparing the yields obtained on varying 4-substituted aryl chlorides (products **3d**–**g**, **u**), no clear trend is observed on changing electronic properties, in line with observations for the iron-catalyzed Suzuki biaryl coupling of aryl chlorides.^32^

We next turned our attention to the use of aryl triflates as substrates. Pleasingly, phenyl triflate performed essentially identically with chlorobenzene under the same reaction conditions. Notably, leaving out the NHC ligand gave only a slight drop in yield (Table S5); accordingly, we employed coligand free Fe(OTf)_2_ as a precatalyst in the borylation of a range of aryl triflates, as summarized in Figure 3.

**Figure 3:**
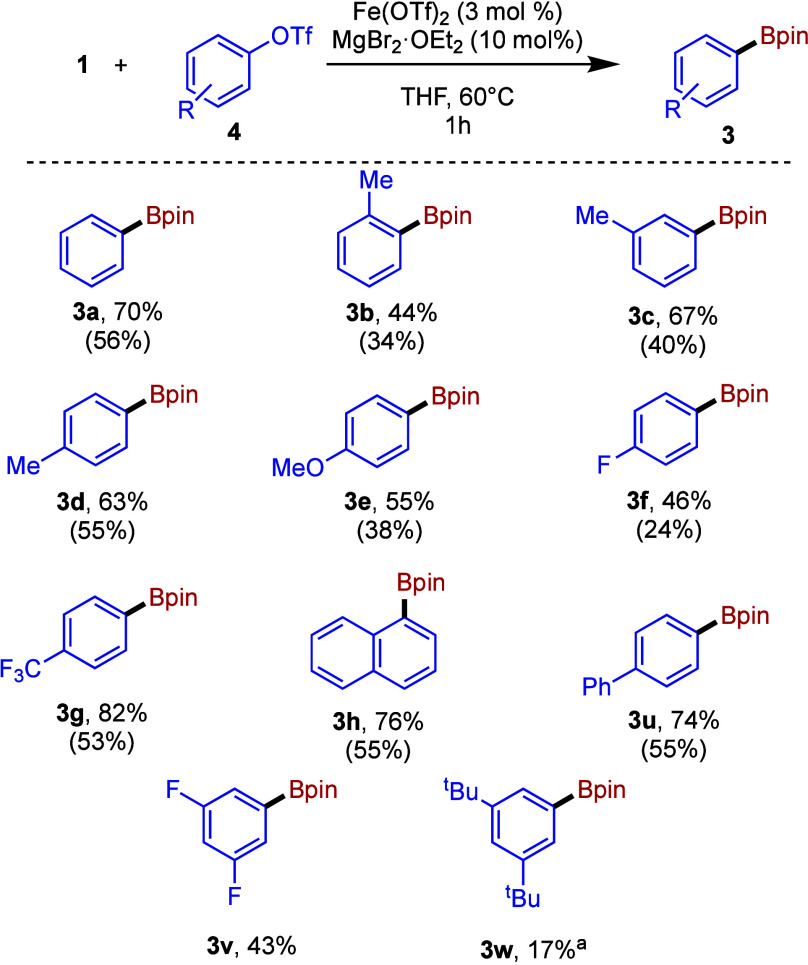
Fe-catalyzed borylation of aryl triflates. Conditions: **1** (0.6 mmol), **4** (0.5 mmol), MgBr_2_·OEt_2_ (0.1 mmol), Fe(OTf)_2_ (0.015 mmol), THF, 60 °C, 1 h. Yields determined by ^1^H NMR spectroscopy (1,3,5-trimethoxybenzene or mesitylene internal standard). ^a^Could not be separated. ^b^Purification not attempted.

With electron-rich aryl-triflate substrates, the performance was generally similar to that observed with the aryl chlorides, with *p*-tolyl and *p*-methoxyphenyl triflate giving slightly lower yields of **3d** and **e**, respectively, as obtained with the chloride counterparts. However, *o*-tolyl triflate furnished **3b** in noticeably poorer yield than the corresponding chloride, suggesting that steric bulk is less well tolerated here. Substrates with extended conjugation again exhibited higher yields, as illustrated by the formation of naphthyl and biphenyl products (**3h** and **3u**). Interestingly, the electron-deficient triflates tested outperformed the respective aryl chlorides, with both **3f** and **g** being formed in significantly higher yields.

With regard to the possible mechanism, we have yet to undertake a detailed study, but the following points are noted. First, we have previously shown that the borylating reagent **1** is capable of reducing iron precatalysts to iron(I) species,^38^ and it is anticipated that this may occur here, although it should be noted that, to the best of our knowledge, such reductions to Fe(I) have not been demonstrated with iron fluoride or triflate precursors—the systems that fare best here.

Second, it has previously been calculated that oxidative addition of aryl chloride substrates to Fe(I) is far more facile than the equivalent reaction at Fe(0), both with^32^ and without^42^ the ligand IMes present. Conversely, transmetalation from boron to iron has been calculated to be challenging;^32^ accordingly, it is likely that transmetalation of a boryl may be turnover limiting here.

Preliminary radical trap experiments do not indicate the involvement of radicals. Addition of 1 equiv of TEMPO to the borylation of **2a** shuts down the reaction, with less than 1% of product **3a** observed, but GC-MS analysis of the crude reaction mixture did not reveal the formation of any adducts formed between TEMPO and either phenyl or BPin radicals. Similarly, the addition of 1,1-diphenylethene to the borylation of **2a** gave only 4% of **3a** and a small amount (17%) of the hydroboration product.^43^ Again, no adducts formed from an aryl radical were observed.

In summary, we have extended the iron-catalyzed borylation of aryl chlorides to simple substrates that have previously shown poor performance. In addition, we have extended the reaction to the first examples, to the best of our knowledge, of the iron-catalyzed borylation of aryl triflates. Ongoing efforts are targeted toward replacing **1** as the borylating reagent with less air- and moisture-sensitive analogues and to probing the mechanism of the catalytic reaction.

## General Procedure for the Iron-Catalyzed Borylation of Aryl Chlorides

In an argon-filled glovebox, Fe(OTf)_2_ (5.3 mg, 15 μmol) was weighed into an oven-dried 7 mL vial, followed by IMes (2.3 mg, 7.5 μmol) dispensed from a 0.015 M stock solution in THF (0.5 mL). A further 0.5 mL of THF was added, and the suspension was stirred at 60 °C for 30 min, during which time a darkening of the color was observed. The appropriate aryl chloride **2** (0.5 mmol) was then added, along with MgBr_2_·OEt_2_ (6.4 mg, 0.05 mmol) dispensed from a 0.1 M stock solution in THF (0.5 mL). A further 0.5 mL of THF was added, and the reaction mixture was stirred for a further 15 min at 60 °C. The boronate **1** was added and rinsed into the vial with another 2 mL of THF (total volume = 4 mL), and the resultant reaction mixture was stirred at 60 °C for 1 h. The vial was then removed from the glovebox and allowed to cool to room temperature. The reaction was then quenched by exposure to air, followed by addition of an internal standard (either 1,3,5-trimethoxybenzene or mesitylene, 0.5 mmol, SI for details) from a stock solution in dichloromethane (0.5 M) and immediate filtration through a pad of Celite, eluting with hexane. An aliquot was taken for analysis by ^1^H NMR spectroscopy to determine the spectroscopic yield. The NMR sample was then recombined with the crude mixture, which was then transferred to a Schlenk tube. Volatiles were removed under vacuum and heating at 35 °C to ensure removal of the ^t^BuBpin byproduct. The desired product **3** was then purified by column chromatography on silica (SI for details).

## General Procedure for the Iron-Catalyzed Borylation of Aryl Triflates

In an argon-filled glovebox, Fe(OTf)_2_ (5.3 mg, 15 μmol) was weighed into an oven-dried 7 mL vial, and THF (1 mL) was added. The appropriate aryl triflate **4** (0.5 mmol) was then added, along with MgBr_2_·OEt_2_ (6.4 mg, 0.05 mmol) dispensed from a 0.1 M stock solution in THF (0.5 mL). Another 0.5 mL of THF was added and the reaction stirred for 15 min at 60 °C. The boronate **1** was added and rinsed into the vial with a further 2 mL of THF (total volume = 4 mL), and the resultant reaction mixture was stirred at 60 °C for 1 h. The vial was then removed from the glovebox and allowed to cool to room temperature. The reaction was then quenched by exposure to air, followed by addition of internal standard (either 1,3,5-trimethoxybenzene or mesitylene, 0.5 mmol, SI for details) from a stock solution in dichloromethane (0.5 M) and immediate filtration through a pad of Celite, eluting with hexane. An aliquot was taken for analysis by ^1^H NMR spectroscopy to determine the spectroscopic yield. The NMR sample was then recombined with the crude mixture, which was then transferred to a Schlenk tube. Volatiles were removed under vacuum and heating at 35 °C to ensure removal of the ^t^BuBpin byproduct. The desired product **3** was then purified by column chromatography on silica (SI for details).

## Data Availability

The data underlying this study are openly available in data.bris, at 10.5523/bris.3nt2av52pzf8h1y93y4b8bonas.

## Supporting Information Available

Full experimental details and characterization of new compounds (PDF)
