# Supplementary material for: Iron-Catalyzed Miyaura Borylation of Aryl Chlorides and Triflates
Source: Org Lett. 2024 Dec 17;27(1):197–201. doi: 10.1021/acs.orglett.4c04171 (PMC11731378; doi:10.1021/acs.orglett.4c04171)
Supplement: Supplementary file 1 — ol4c04171_si_001.pdf [file ol4c04171_si_001.pdf]

Supporting Information

Iron-catalyzed Miyaura Borylation of aryl chlorides and triflates

*Patrick Daley-Dee[1], James Clarke[2], Sebastien Monfette[2], Robin B. Bedford[1]**

[1]School of Chemistry, University of Bristol, Cantock's Close, Bristol, BS8 1TS, UK

[2]Pfizer Inc.

*Email: r.bedford@bristol.ac.uk

1 Table of Contents

2 General considerations 2
3 Reaction optimization 2
3.1 General procedure for reaction optimization 2
3.2 Screening data 3
3.2.1 Aryl halide screen 3
3.2.2 Preliminary ligand screen 3
3.2.3 Precatalyst screen 5
3.2.4 Additive screen 7
3.2.5 Solvent and temperature screen 8
3.2.6 Further optimization 9
4 Poorly reactive and unreactive substrates 10
5 Synthesis of aryl triflates (general procedure A) 11
6 Optimized iron-catalyzed borylation 11
6.1 Procedure for the iron-catalyzed borylation of aryl chlorides (general procedure B) 11
6.2 Procedure for the iron-catalyzed borylation of aryl triflates (general procedure C) 11
6.3 1 mmol scale reaction 12
7 Product characterisation 13
7.1 Aryl triflates 13
7.2 Aryl boronic acid pinacol esters 17
8 Radical probe experiments 23
8.1 General procedure for radical probe experiments 23
8.2 Data from radical probe experiments 24
8.2.1 Reaction with TEMPO 24
8.2.2 Reaction with 1,1-DPE 27
9 NMR spectra of isolated compounds 32
10 References 82

2 General considerations

All manipulations, unless otherwise stated were carried out using standard air-sensitive Schlenk line and glovebox techniques. All reagents were purchased from commercial suppliers and used without purification unless specified. The following solvents were obtained from the University of Bristol's dry solvent system and were purified by filtration over a column of activated alumina: THF, toluene, hexane, dichloromethane, Et_2O, acetonitrile. All solvents were stored in sealed ampules over 3 or 4 Å molecular sieves. Aryl halides were purchased from chemical suppliers, as were phenyl trifluoromethanesulfonate (**4a**) and *p*-tolyl trifluoromethanesulfonate (**4d**), the other aryl triflates were synthesized as described below. Aryl halides and triflates were dried either over 3 Å molecular sieves for at least 48 hours prior to use or by azeotropic removal of water with toluene. TLC was performed on Merck silica-aluminium plates and spots visualized with UV light (254 nm), or by staining with a basic aqueous potassium permanganate solution or an acidic ethanolic solution of curcumin. Flash column chromatography was performed using technical grade silica gel, pore size 60 Å, 230-400 mesh particle size. $Li[B_2pin_2(^tBu)]$,**1**, was prepared by a published method.[1] Note, this requires using tBuLi: *Caution! Tert-butyllithium is extremely pyrophoric. It must be handled using proper needle and syringe techniques.* The following were also prepared according to literature procedures: $6IPr{\cdot}HBF_4$, $6Mes{\cdot}HBF_4$, $7Mes{\cdot}HBF_4$, $8Mes{\cdot}HBF_4$,[2] $[FeCl_2(dppe)]$.[3]

NMR spectra were obtained using Bruker Avance 400 MHz Jeol, ECZ 400 MHz or Jeol ECS 400 MHz spectrometers and analysed in MestReNova x64 software. Chemical shifts are reported in ppm and referenced to residual solvent peaks. Multiplicities are reported as singlet (s), doublet (d), triplet (t), quartet (q), multiplet (m), broad (br) or combinations thereof. GC-MS data was obtained using an Agilent Technologies 7820A GC / MSD 5977E, with the following settings: 45 °C (3 min); ramp from 45 °C to 300 °C (7.5 min), hold at 300 °C (3 min) - total run time 13.5 min. HPLC-MS data was obtained using an Agilent Technologies 1260 Infinity III LC System with the following settings:

H_2O (%)	MeCN (%)	Time from injection (min)	Flow rate (mL/min)
90	10	0	1
10	90	15	1
5	95	17	1
95	5	17.2	0.1

Calibration curves with five different concentrations of chlorobenzene (R^2 = 0.9999), phenylboronic acid pinacol ester (R^2 = 0.9994) and 4,4'-di-tert-butylbiphenyl (internal standard, R^2 = 0.996) were constructed to enable quantitative analysis by HPLC-MS. Mass spectrometry was performed by the University of Bristol mass spectrometry service by electron ionisation (EI) using a Thermo Scientific QExactive instrument or electrospray ionisation (ESI) using a Thermo Scientific Oribitrap Elite.

3 Reaction optimization

3.1 General procedure for reaction optimization

To an oven-dried vial charged with a stirrer bar, the iron salt (0.0125 mmol, 0.05 equiv.), ligand (0.0125 mmol, 0.05 equiv.), base (0-0.0125 mmol, 0-0.05 equiv., see Table S2, entries 20-27)) and solvent (0.5 mL) were added and stirred at the specified temperature for 30 min (aluminium heating block). To this mixture, the aryl substrate (0.25 mmol, 1 equiv.), additive (0.10 mmol, 0.2 equiv.), 4,4'-di-tert-butylbiphenyl (67 mg, 0.25 mmol, 1 equiv.) and solvent (1 mL) were added and the mixture stirred for a further 15 min. Lithium boronate **1** (110 mg, 0.3 mmol, 1.4 equiv.) was then added and rinsed into the reaction mixture with a further 0.5 mL solvent, bringing the total volume of solvent to 2 mL (0.125 M wrt aryl substrate). The reaction mixture was then stirred at the specified temperature (aluminium heating block) for the indicated time period before allowing to cool to room temperature. The reaction was then exposed to air, diluted to 8 mL with EtOAc, and an aliquot immediately quenched by filtration through a celite plug, eluting fully under a stream of N_2 gas. The aliquot was diluted further in HPLC grade MeCN and taken for analysis by LC-MS.

Changes to the above procedure and loadings of the reagents are documented under the appropriate screening results (Tables S1-S5/Fig S1). Ligand, internal standard and additive were dispensed as stock solutions in the reaction solvent unless otherwise indicated. In instances where HPLC-MS could not be used for quantitative

analysis due to a technical issue with the instrument, spectroscopic yields were determined by ^{1}H NMR using 1,3,5-trimethoxybenzene as internal standard (1 equiv. wrt aryl substrate).

3.2 Screening data

3.2.1 Aryl halide screen

Table S1. Aryl halide and pseudohalide screen

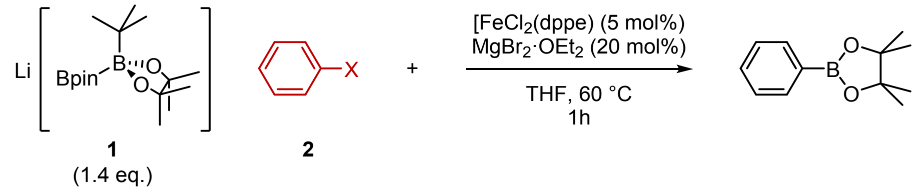

Entry	Halide/pseudohalide	Yield of **3a** (%)[a]
**1**	**Chlorobenzene**	**18**
2	Bromobenzene	12
3	Iodobenzene	≈1
**4**	**Phenyl trifluoromethanesulfonate**	**30**
5	Phenyl carbamate	0[b]

Conditions: aryl halide (0.25 mmol), **1** (0.35 mmol), [$FeCl_2$(dppe)] (0.013 mmol), $MgBr_2$·OEt_2 (0.05 mmol dispensed from 0.1 M stock solution), THF (2 mL total), temperature = 60 °C, time = 1h. [a]Yield determined by ^{1}H NMR with 1,3,5-trimethoxybenzene as internal standard. [b]Not detected by GCMS or ^{1}H NMR.

3.2.2 Preliminary ligand screen

Table S2. Preliminary ligand Screen

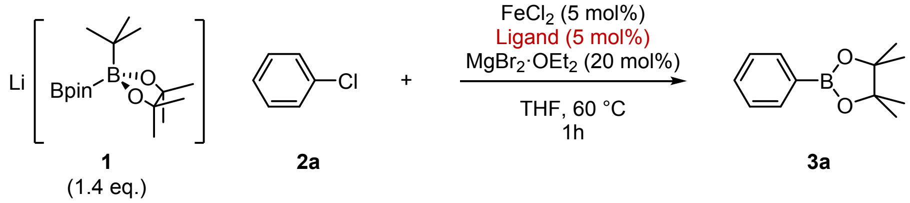

Entry	Ligand		Base	Yield of **3a** (%)[a]	Consumption of **2a** (%)[a]
1	none		none	7	99
2	dppe	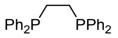	none	4	49
**3**	**dcpe**	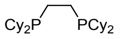	**none**	**8**	**53**
**4**	**dmpe**	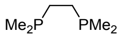	**none**	**7**	**98**
5	depe	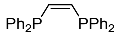	none	1	99
6	Xantphos	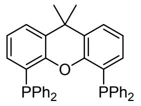	none	2	99
7	dppbz	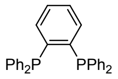	none	1	99
8	dppp	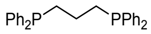	none	1	99
9	dppe[b]	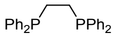	none	2	50
10	PPh_3	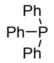	none	2	48

11	$P(4\text{-}MeOPh)_3$	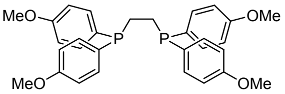	none	3	44
12	1,8-dppn	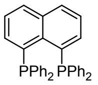	none	2	21
13	dClpbz	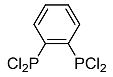	none	3	26
14	dippa	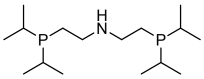	none	5	28
15	*cis*-dppe	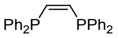	none	3	26
16	*trans*-dppe	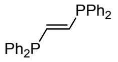	none	1	17
**17**	**^{t}Bu-Xantphos**	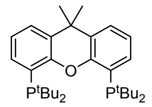	**none**	**7**	**21**
**18**	**SciOPP**	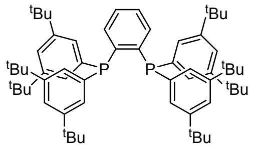	**none**	**4**	**25**
**19**	**L-PN**	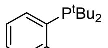	**none**	**6**	**31**
20	SIMes.HCl	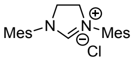	LiHMDS[c]	6	47
21	SIPr.HCl	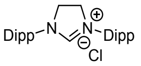	LiHMDS[c]	4	39
22	IMes.HCl	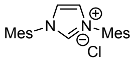	LiHMDS[c]	11	39
23	Ipr.HCl	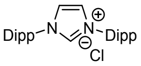	LiHMDS[c]	5	59
**24**	**$6Mes.HBF_4$**	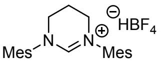	**LiHMDS[c]**	**23**	**59**
25	$7Mes.HBF_4$	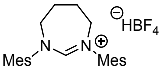	LiHMDS[c]	3	38
26	$8Mes.HBF_4$	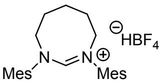	LiHMDS[c]	8	34
27	$6IPr.HBF_4$	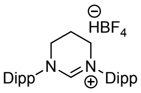	LiHMDS[c]	8	35
28	IMes	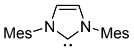	none	15	39

Conditions: chlorobenzene (0.25 mmol), **1** (0.35 mmol), $FeCl_2$ (0.013 mmol dispensed from 0.025 M stock solution), ligand (0.013 mmol, manually weighed), $MgBr_2{\cdot}OEt_2$ (0.05 mmol dispensed from 0.1 M stock solution), 4,4'-ditertbutylbiphenyl (0.25 mmol dispensed from 0.5 M stock solution), THF (2 mL total) temperature = 60 °C, time = 1h. [a]Determined by HPLC-MS calibrated against 4,4'-di-tert-butylbiphenyl as internal standard. [b]dppe (0.025 mmol). [c]Base LiHMDS (0.0125 mmol) added as base to enable formation of the free ligand.

The ligands highlighted in bold were carried forward for further screening (see ligand/precatalyst screen below Figure S1)

3.2.3 Precatalyst screen

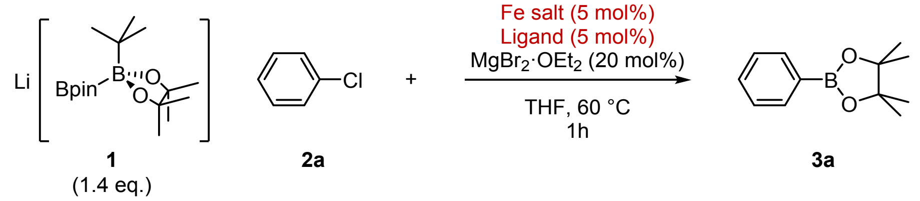

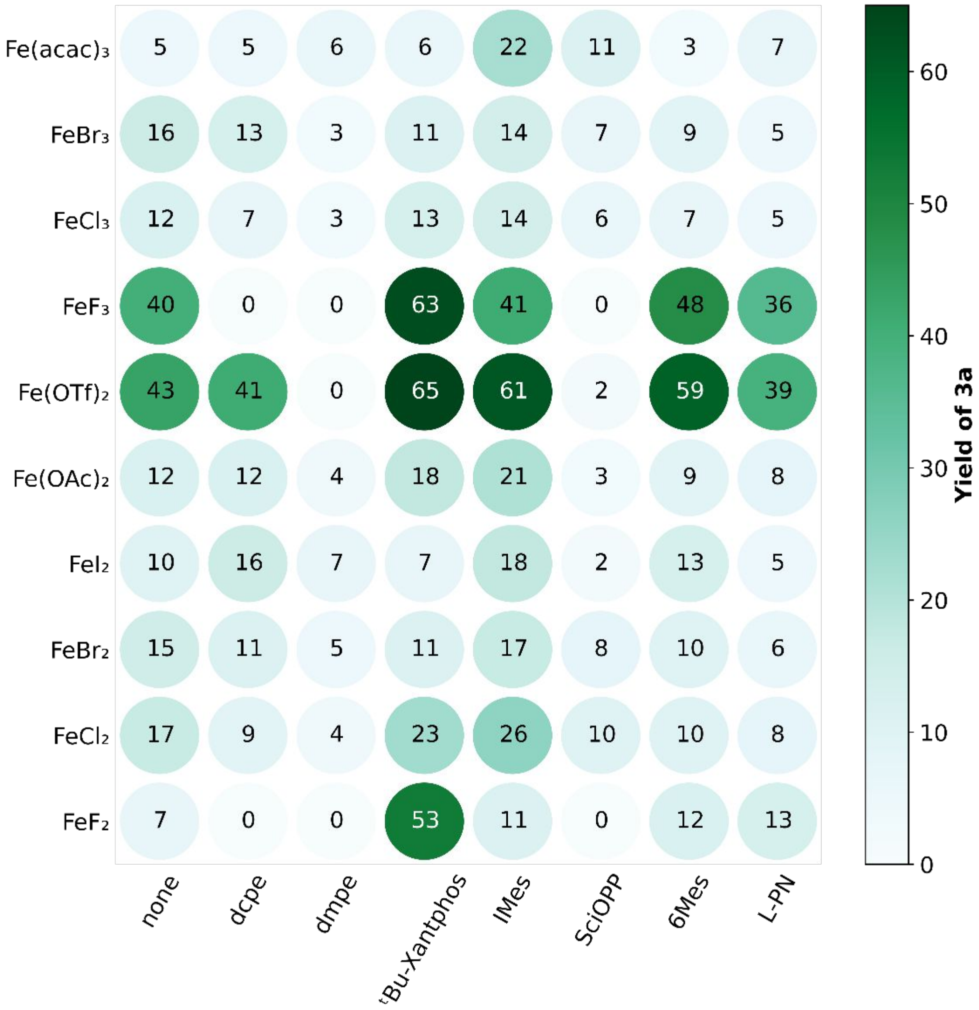

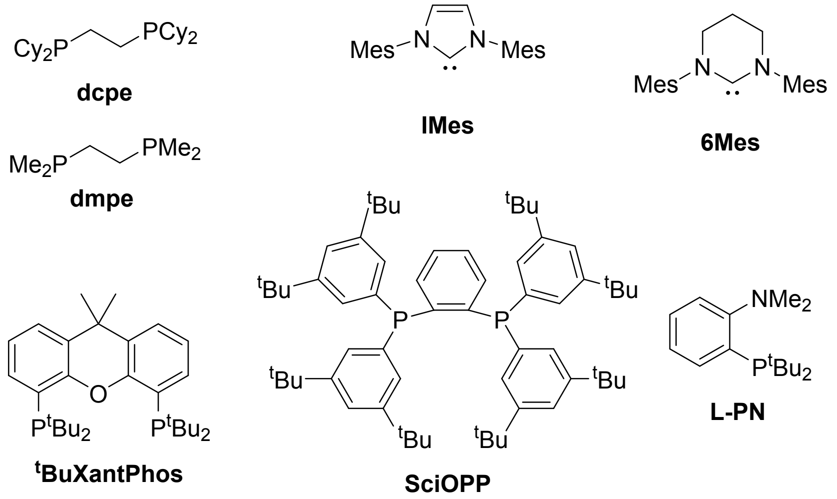

Figure S1. Precatalyst screen. Conditions: chlorobenzene (0.25 mmol), **1** (0.35 mmol), iron salt (0.013 mmol), ligand (0.013 mmol dispensed from 0.025 M stock solution), $MgBr_2 \cdot OEt_2$ (0.05 mmol dispensed from 0.1 M stock solution), 4,4'-ditertbutylbiphenyl (0.25 mmol dispensed from 0.5 M stock solution), THF (2 mL total), temperature = 60 °C, time = 1h. Yields determined by HPLC-MS calibrated against 4,4'-di-tert-butylbiphenyl as internal standard. Note: $6Mes \cdot HBF_4$ deprotonated *in situ* by addition of LiHMDS (0.125 mmol).

3.2.4 Additive screen

Table S3. Additive screen

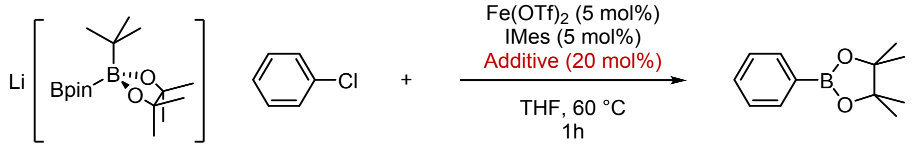

Entry	Additive	Yield of **3a** (%)[a]	Consumption of **2a** (%)[a]
1	none	38	75
2	LiF	44	81
3	NaF	49	87
4	KF	36	60
5	LiCl	25	64
6	NaCl	58	87
7	KCl	44	70
8	$MgCl_2$	44	83
9	$AlCl_3$	53	89
10	$ZnCl_2$	57	80
11	TMAC	42	67
12	TEAC	6	77
13	TBAC	45	68
14	LiBr	57	78
15	NaBr	54	93
16	KBr	32	60
17	$MgBr_2$	41	79
**18**	**$MgBr_2{\cdot}OEt_2$**	**64**	**88**
19	$AlBr_3$	33	73
20	$GaBr_3$	7	40
21	TMAB	34	67
22	TEAB	35	68
23	TBAB	15	49
24	NaI	44	79
25	KI	16	49

Conditions: chlorobenzene (0.25 mmol), **1** (0.35 mmol), $Fe(OTf)_2$ (0.013 mmol), IMes (0.013 mmol dispensed from 0.025 M stock solution), $MgBr_2{\cdot}OEt_2$ (0.05 mmol dispensed from 0.1 M stock solution), THF (2 mL total), temperature = 60 °C, time = 1h. [a]Yields determined by HPLC-MS calibrated against 4,4'-ditertbutylbiphenyl as internal standard.

3.2.5 Solvent and temperature screen

Table S4. Solvent and temperature screen

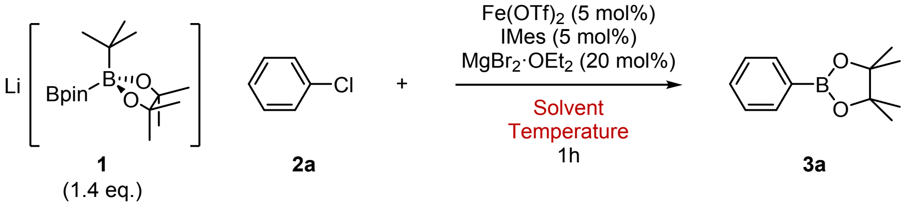

Entry	Solvent	Temperature (°C)	Yield of **3a** (%)[a]	Consumption of **2a** (%)[a]
**1**	**THF**	**60**	**48**	**83**
2	2-MeTHF	60	25	66
3	MeCN	60	0	34
4	Toluene	60	0	n/a[b]
5	DCE	60	1	35
6	MTBE	60	5	36
7	1,4-dioxane	60	3	40
8	DMA	60	32	73
9	DMA	100	38	84
10	DMA	120	41	87

Conditions: chlorobenzene (0.25 mmol), **1** (0.35 mmol), $Fe(OTf)_2$ (0.013 mmol), IMes (0.013 mmol), $MgBr_2{\cdot}OEt_2$ (0.05 mmol), solvent (2 mL total), time = 1h. [a]Yields determined by HPLC-MS calibrated against 4,4'-ditertbutylbiphenyl as internal standard. All reagents weighed manually. [b]Unable to obtain accurate data due to peak overlap with solvent in HPLC-MS.

3.2.6 Further optimization

Note: due an issue with the HPLC-MS instrument, some reactions had to be analysed by ^{1}H NMR (Table S5, entries 12-19).

Table S5. Further optimization of the reaction conditions

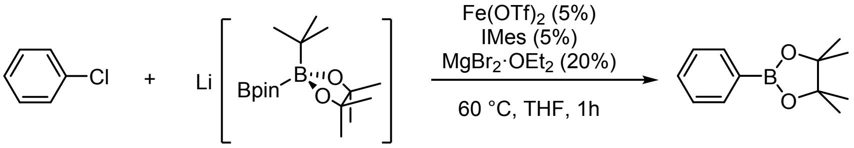

1.2 eq

1 **3a**

Entry	Deviation from standard reaction conditions	Yield of 3a (%)[a]	Consumption of 2a (%)[a]
1	No Fe or ligand	1	33
2	IMes only	1	32
3	Boronate weighed in air	44	84
4	In air	12	68
5	0.25 eq IMes (vs Fe)	59[b]	N/A
6	0.33 eq IMes (vs Fe)	57[b]	N/A
7	0.5 eq IMes (vs Fe)	68[b,c]	78
8	0.75 eq IMes (vs Fe)	57[b]	N/A
9	10% $MgBr_2{\cdot}OEt_2$	56	82
10	20% $MgBr_2{\cdot}OEt_2$	50	82
11	25% $MgBr_2{\cdot}OEt_2$	47	80
12	1% Fe, 0.5 % IMes	9[b]	N/A
**13**	**3% Fe, 1.5 % IMes**	**70[b]**	**N/A**
14	10% Fe, 5% IMes	47[b]	N/A
15	1.0 eq **1**	53[b]	N/A
16	1.2 eq **1**	61[b]	N/A
17	1.6 eq **1**	47[b]	N/A
18	1.8 eq **1**	55[b]	N/A
19	2.0 eq **1**	58[b]	N/A
20	ArX = Phenyl triflate	72[b]	N/A
21	ArX = Phenyl triflate, no ligand	69[b]	N/A

Conditions: chlorobenzene (0.25 mmol), **1**, $Fe(OTf)_2$ IMes, $MgBr_2{\cdot}OEt_2$ (amounts specified above), solvent (2 mL total), time = 1h. [a]Determined by HPLC-MS calibrated against 4,4'-di-tert-butylbiphenyl as internal standard. [b]Determined by ^{1}H NMR with 1,3,5-trimethoxybenzene as internal standard. [c]Average across 2 runs.

4 Poorly reactive and unreactive substrates

Figure S2. Substrates that gave little or no desired product[a]

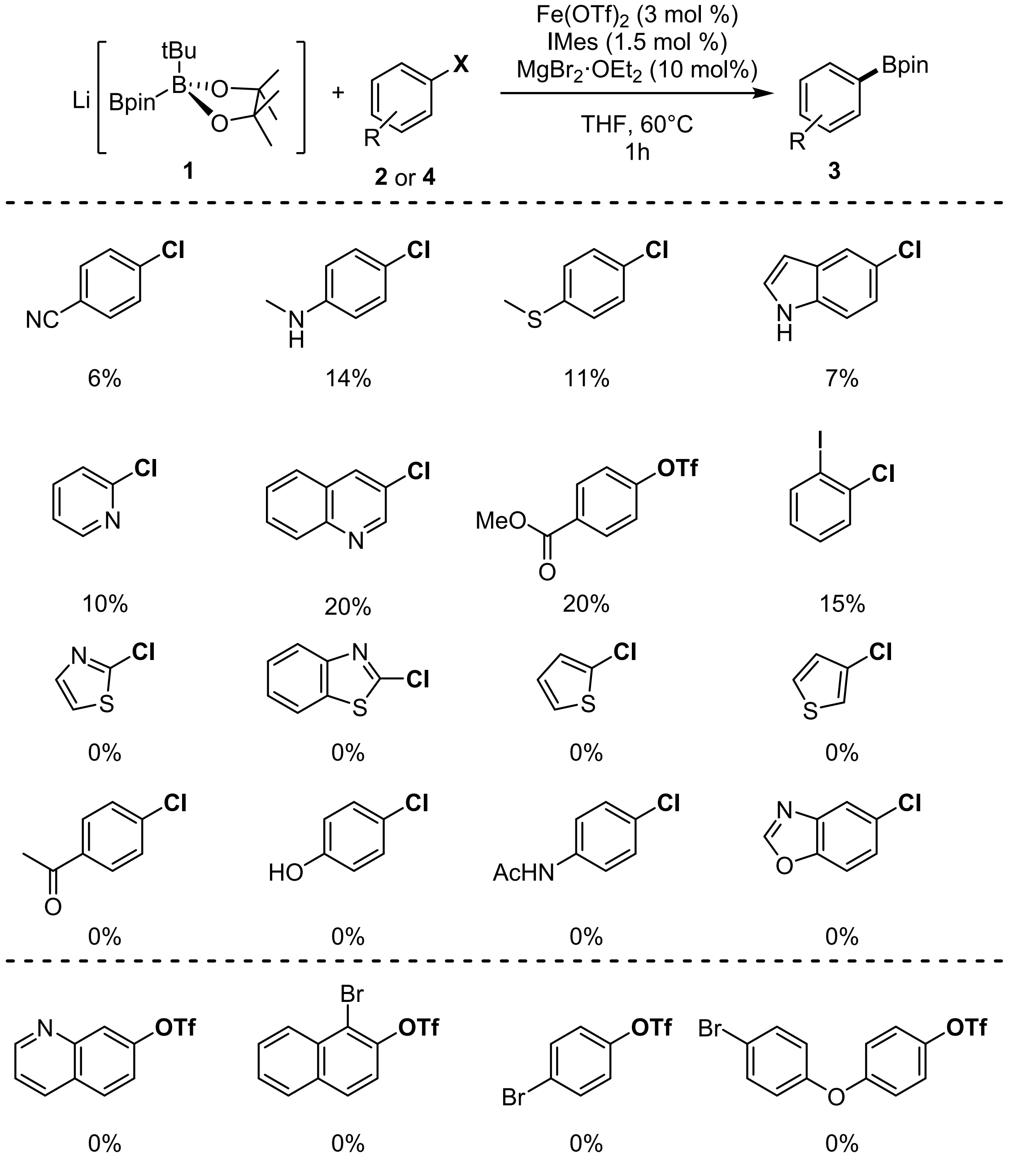

Conditions: **1** (0.6 mmol), **2** or **4** (0.5 mmol), $MgBr_2{\cdot}OEt_2$ (0.1 mmol), $Fe(OTf)_2$ (0.015 mmol), ligand (0.008 mmol), THF, 60 °C, 1h (after addition of boronate **1**). Yields determined by ^1H NMR of the crude reaction mixture with 1,3,5-trimethoxybenzene (0.5 mmol) or mesitylene (0.50 mmol) as internal standard. [a]Yield of borylation product shown.

5 Synthesis of aryl triflates (general procedure A)

Aryl triflates were synthesised from the corresponding phenols according to a modified general procedure reported by Joseph *et al.*[4]

The phenol (1 mmol) and pyridine (0.162 mL , 2 mmol, 2 equivalents) were added to an oven-dried Schlenk tube and dissolved in anhydrous dichloromethane (5 mL). The solution was cooled to 0 °C and trifluoromethanesulfonic anhydride (0.202 mL, 1.2 mmol, 1.2 equivalents) was added dropwise with stirring. The progress of the reaction was monitored by TLC and, if necessary, a further 0.2 equivalents (0.034 mL, 0.2 mmol) of the anhydride were added. Upon completion, the reaction was quenched with water and washed with saturated $NaHCO_3$ (3 × 5 mL) and brine (5 mL). The organic components were dried with Na_2SO_4 and the solvent removed under vacuum. The crude product was purified by column chromatography if necessary. Solid products were dried by azeotropic removal of water with toluene before use in the borylation reaction. Oils were dissolved in anhydrous THF (1 mL) and stored over 3 Å molecular sieves for a period of at least 48 hours before use.

6 Optimized iron-catalyzed borylation

6.1 Procedure for the iron-catalyzed borylation of aryl chlorides (general procedure B)

$Fe(OTf)_2$ (5.3 mg, 15 µmol, 0.03 equiv.) was weighed into an oven-dried 7 mL vial, followed by IMes (2.3 mg, 7.5 µmol, 0.015 equiv.) dispensed from a 0.015 M stock solution in THF (0.5 mL). A further 0.5 mL THF was added and the suspension stirred at 60 °C (aluminium heating block, external temperature) for 30 mins, during which time a darkening of colour occurred. The appropriate aryl chloride (0.5 mmol, 1 equiv.) was then added, along with $MgBr_2·OEt_2$ (13 mg, 0.05 mmol, 0.1 equiv.) dispensed from a 0.1 M stock solution in THF (0.5 mL). A further 0.5 mL THF was added and the reaction stirred for 15 min at 60 °C. The boronate **1** (190 mg, 0.6 mmol, 1.2 equiv) was added and rinsed into the vial with a further 2 mL THF (total volume = 4mL, 0.125M wrt aryl chloride) and the reaction mixture was stirred at 60 °C for 1h. The vial was then removed from the glovebox and allowed to cool to room temperature. The reaction was then quenched by exposure to air, followed by addition of internal standard (0.5 mmol, 1 equiv.) from a stock solution in DCM (0.5 M) and immediate filtration through a pad of celite, eluting fully with hexane. An aliquot was taken for analysis by ^1H NMR spectroscopy.

The NMR sample was recombined with the crude mixture, which was then transferred to a Schlenk tube. Volatiles were removed under vacuum, heating at 35 °C (oil bath) to ensure removal of the ^tBuBpin byproduct. The boronic ester was then purified by column chromatography on silica.

Note: yield analysis was carried out by ^1H NMR using 1,3,5-trimethoxybenzene (84 mg, 0.50 mmol, 1 equiv.) as internal standard, added after completion of the reaction. In some instances, this led to co-elution of the desired product with the internal standard during column chromatography. In such instances, mesitylene (120 mg, 0.5 mmol, 1 equiv.) was added as internal standard.

6.2 Procedure for the iron-catalyzed borylation of aryl triflates (general procedure C)

$Fe(OTf)_2$ (5.3 mg, 15 µmol, 0.03 equiv.) was weighed into an oven-dried 7 mL vial and THF (1 mL) was added. The aryl triflate (0.5 mmol, 1 equiv.) was then added, along with $MgBr_2·OEt_2$ (13 mg, 0.05 mmol, 0.1 equiv.) dispensed from a 0.1 M stock solution in THF (0.5 mL). A further 0.5 mL THF was added and the reaction stirred for 15 min at 60 °C. The boronate **1** (190 mg, 0.6 mmol, 1.2 equiv.) was added and rinsed into the vial with a further 2 mL THF (total volume = 4mL, 0.125M wrt aryl triflate) and the reaction mixture was stirred at 60 °C (aluminium heating block, external temperature) for 1h. The vial was then removed from the glovebox and allowed to cool to room temperature. The reaction was then quenched by exposure to air, followed by addition of internal standard (0.5 mmol) from a stock solution in DCM (0.5 M) and immediate filtration through a pad of celite, eluting fully with hexane. An aliquot was taken for analysis by ^1H NMR spectroscopy.

The NMR sample was recombined with the crude mixture, which was then transferred to a Schlenk tube. Volatiles were removed under vacuum, heating at 35 °C (oil bath) to ensure removal of the ^tBuBpin byproduct. The boronic ester was then purified by column chromatography.

6.3 1 mmol scale reaction

$Fe(OTf)_2$ (10.6 mg, 30 μmol, 0.03 equiv.) was weighed into an oven-dried Schlenk tube, followed by IMes (4.6 mg, 15 μmol, 0.015 equiv.). THF (2 mL) was added and the suspension stirred at 60 °C (aluminium heating block, external temperature) for 30 mins, during which time a darkening of colour occurred. Chlorobenzene (112 mg, 1 mmol, 1 equiv.) was then added, along with $MgBr_2 \cdot OEt_2$ (26 mg, 0.1 mmol, 0.1 equiv.) dispensed from a 0.1 M stock solution in THF (1 mL). A further 1 mL THF was added and the reaction stirred for 15 min at 60 °C. The boronate **1** (380 mg, 1.2 mmol, 1.2 equiv.) was added and rinsed into the reaction vessel with a further 4 mL THF (total volume = 8mL, 0.125M wrt chlorobenzene) and the reaction mixture was stirred at 60 °C for 1h. The reaction vessel was then removed from the glovebox and allowed to cool to room temperature. The reaction was then quenched by exposure to air, followed by addition of mesitylene (internal standard, 120 mg, 1 mmol, 1 equiv.) and immediate filtration through a pad of celite, eluting fully with hexane. An aliquot was taken for analysis by ^1H NMR spectroscopy. The NMR sample was recombined with the crude mixture and the volatiles removed under vacuum, heating at 35 °C (oil bath) to ensure removal of the ^tBuBpin byproduct. The boronic ester **3a** was then purified by column chromatography on silica (2% Et_2O in petroleum ether) to afford a colourless oil (94 mg, 46%).

7 Product characterisation

7.1 Aryl triflates

***o*-tolyl trifluoromethanesulfonate (4b)**

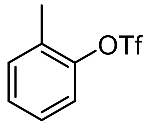

Prepared from *o*-cresol (216 mg, 2 mmol) according to general procedure A. Product purified by flash column chromatography (0-10% EtOAc in hexane) to afford a clear oil (362 mg, 75%). Spectroscopic data consistent with literature.[5]

^{1}H NMR (400 MHz, $CDCl_3$) δ 7.34 – 7.21 (m, 4H), 2.39 (s, 3H).
^{13}C NMR (101 MHz, $CDCl_3$) δ 148.7, 132.3, 131.0, 128.4, 127.8, 121.4, 118.8 (q, *J* = 320.0 Hz), 16.4.
^{19}F NMR (377 MHz, $CDCl_3$) δ -73.85.
MS (EI) m/z: M^+ Calcd for $C_8H_7F_3O_3S$ 240.01; Found 240.0.

***m*-tolyl trifluoromethanesulfonate (4c)**

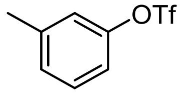

Prepared from *m*-cresol (226 mg, 2.1 mmol) according to general procedure A. Product purified by flash column chromatography (0-10% EtOAc in hexane) to afford a clear oil (301 mg, 60%). Spectroscopic data consistent with literature.[6]

^{1}H NMR (400 MHz, $CDCl_3$) δ 7.33 (t, *J* = 7.5 Hz 1H), 7.20 (d, *J* = 7.4 Hz, 1H), 7.10 – 7.03 (m, 2H), 2.41 (s, 3H).
^{13}C NMR (101 MHz, $CDCl_3$) δ 149.7, 141.0, 130.0, 129.3, 118.9 (q, J = 320.7 Hz), 68.1, 25.7, 21.3.
^{19}F NMR (376 MHz, $CDCl_3$) δ -72.88.
MS (EI) m/z: M^+ Calcd for $C_8H_7F_3O_3S$ 240.01; Found 240.0.

4-methoxyphenyl trifluoromethanesulfonate (4e)

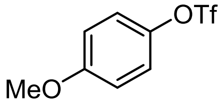

Prepared from mequinol (248 mg, 2 mmol) according to general procedure A. Purified by flash column chromatography (0-10% EtOAc in hexane) to afford a clear oil (331 mg, 65%). Spectroscopic data consistent with the literature.[6]

^{1}H NMR (400 MHz, $CDCl_3$) δ 7.20 (d, *J* = 9.3 Hz, 2H), 6.92 (d, *J* = 9.3 Hz, 2H), 3.82 (s, 3H).
^{13}C NMR (101 MHz, CDCl3) δ 159.3, 143.2, 118.9 (q, J = 320.8 Hz), 55.8
^{19}F NMR (377 MHz, $CDCl_3$) δ -72.70.
MS (EI) m/z: M^+ Calcd for $C_8H_7F_3O_4S$ 256.00; Found 256.0.

4-fluorophenyl trifluoromethanesulfonate (4f)

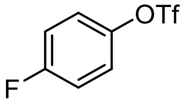

Prepared from 4-fluorophenol (112 mg, 1 mmol) according to general procedure A to afford a clear oil (223 mg, 91%). Crude product of sufficient purity to be used immediately. Spectroscopic data consistent with literature.[7]

^{1}H NMR (400 MHz, $CDCl_3$) δ 7.34 – 7.23 (m, 1H), 7.20 – 7.09 (m, 1H).
^{13}C NMR (101 MHz, $CDCl_3$) δ 161.8 (d, *J* = 248.9 Hz), 145.4 (d, *J* = 3.0 Hz), 123.3 (d, *J* = 9.0 Hz), 118.9 (q, *J*

= 320.8 Hz), 117.3 (d, *J* = 24.1 Hz).
^{19}F NMR (376 MHz, $CDCl_3$) δ -72.69 (3F), -112.28 (1F).
MS (EI) m/z: M^+ Calcd for $[C_8H_4F_4O_3S]^+$ 243.98; Found 244.0.

4-(trifluoromthyl)phenyl trifluoromethanesulfonate (4g)

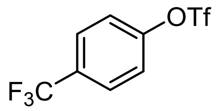

Prepared from 4-(trifluoromethyl)phenol (324 mg, 2 mmol) according to general procedure A. Product purified by flash column chromatography (0-10% EtOAc in hexane) to afford a clear oil (337 mg, 57 %). Spectroscopic data consistent with literature.[6]

^{1}H NMR (400 MHz, $CDCl_3$) δ 7.75 (d, *J* = 8.7 Hz, 2H), 7.46 – 7.38 (m, 2H).
^{13}C NMR (101 MHz, $CDCl_3$) δ 151.6, 131.0 (q, *J* = 33.2 Hz), 127.8 (q, *J* = 3.8 Hz), 123.3 (d, *J* = 272.4 Hz), 122.1, 118.8 (q, *J* = 320.7 Hz).
^{19}F NMR (377 MHz, $CDCl_3$) δ -62.62, -72.67.
MS (EI) m/z: M+ Calcd for $C_8H_4F_6O_3S$ 293.98; Found 294.0.

1-napthyl trifluoromethanesulfonate (4h)

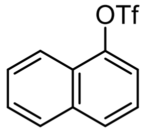

Prepared from 1-naphthol (145 mg, 1 mmol) according to general procedure A to afford a clear oil (227 mg, 82%). Crude product of sufficient purity to be used immediately. Spectroscopic data consistent with literature.[8]

^{1}H NMR (400 MHz, $CDCl_3$) δ 8.09 (dd, *J* = 8.5, 1.2 Hz, 1H), 7.92 (d, *J* = 7.4 Hz, 1H), 7.88 (dd, *J* = 6.5, 2.1 Hz, 1H), 7.70 – 7.56 (m, 2H), 7.53 – 7.39 (m, 2H).
^{13}C NMR (101 MHz, $CDCl_3$) δ 145.8, 135.0, 128.6, 128.2, 128.0, 127.5, 126.5, 125.2, 120.9, 118.9 (q, J = 320.3 Hz),117.9
^{19}F NMR (376 MHz, $CDCl_3$) δ -73.24.
MS (EI) m/z: M^+ Calcd for $C_{11}H_7F_3O_3S$ 276.01; Found 276.0.

4-biphenyl trifluoromethanesulfonate (4u)

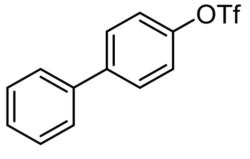

Prepared from 4-phenylphenol (170 mg, 1 mmol) according to general procedure A to afford an off-white solid (94%). Crude product of sufficient purity to be used immediately. Spectroscopic data consistent with literature.[7]

^{1}H NMR (400 MHz, $CDCl_3$) δ 7.72 – 7.61 (m, 2H), 7.60 – 7.54 (m, 2H), 7.52 – 7.44 (m, 2H), 7.44 – 7.39 (m, 1H), 7.39 – 7.31 (m, 2H).
^{13}C NMR (101 MHz, CDCl3) δ 149.1, 141.8, 139.4, 129.1, 129.0, 128.2, 127.3, 121.8, 118.9 (q, J = 320.7 Hz).
^{19}F NMR (376 MHz, $CDCl_3$) δ -72.71.
MS (EI) m/z: M^+ Calcd for $C_{13}H_9F_3O_3S$ 302.02; Found 302.0.

3,5-difluorophenyl trifluoromethanesulfonate (4v)

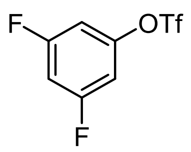

Prepared from 3,5-difluorophenol (260 mg, 2 mmol) according to general procedure A. Purified by flash column chromatography (2-7.5% EtOAc in Hexane). Clear oil (384 mg, 73%). Spectroscopic data consistent with literature.[5]

^{1}H NMR (400 MHz, $CDCl_3$) δ 6.93 – 6.85 (m, 1H).
^{13}C NMR (101 MHz, $CDCl_3$) δ 163.2 (dd, *J* = 252.9, 14.2 Hz), 149.9 (t, *J* = 14.0 Hz), 118.8 (d, *J* = 320.6 Hz), 107.0 – 105.4 (m), 104.8 (t, *J* = 25.1 Hz).
^{19}F NMR (377 MHz, $CDCl_3$) δ -72.64, -105.33 – -105.44 (m).
HRMS (EI) m/z: M^+ Calcd for $C_7H_3F_5O_3S$ 261.9718; Found 261.9714.

3,5-ditertbutylphenyl trifluoromethanesulfonate (4w)

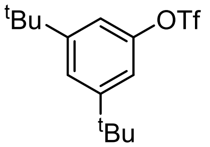

Prepared from 3,5-ditertbutylphenol (206 mg, 2 mmol) according to general procedure A. Purified by flash column chromatography (0-15% EtOAc in hexane). Clear oil (569 mg, 84%). Spectroscopic data consistent with literature.[9]

^{1}H NMR (400 MHz, $CDCl_3$) δ 7.43 (s, 1H), 7.08 (d, *J* = 1.6 Hz, 2H), 1.34 (s, 18H).
^{13}C NMR (101 MHz, $CDCl_3$) δ 153.6, 149.7, 122.3, 118.8 (q, *J* = 320 Hz), 115.6, 35.3, 31.4.
^{19}F NMR (377 MHz, $CDCl_3$) δ -72.93
MS (EI) m/z: M^+ Calcd for $C_{15}H_{21}F_3O_3S$ 338.12; Found 338.1.

4-bromophenyl trifluoromethanesulfonate (see Figure S2)

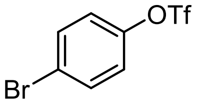

Prepared from 4-bromophenol (173 mg, 1 mmol) according to general procedure A. Purified by flash column chromatography (5% EtOAc in hexane). Clear oil (274 mg, 90%). Spectroscopic data consistent literature.[10]

^{1}H NMR (400 MHz, $CDCl_3$) δ 7.63 – 7.54 (m, 2H), 7.21 – 7.12 (m, 2H).
^{13}C NMR (101 MHz, $CDCl_3$) δ 148.6, 133.6, 123.2, 122.2, 120.4, 118.8 (q, *J* = 320.8 Hz).
^{19}F NMR (377 MHz, $CDCl_3$) δ -72.61.
HRMS (EI) m/z: M^+ Calcd for $C_7H_4BrF_3O_3S$ 303.9011; Found 303.9007

4-(4-bromophenoxy)phenyl trifluoromethanesulfonate (see Figure S2)

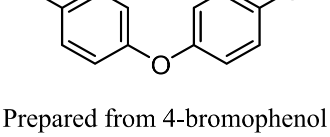

Prepared from 4-bromophenol (265 mg, 1 mmol) according to general procedure A. Purified by flash column chromatography (5% EtOAc in hexane). Clear oil (255 mg, 96%). Novel compound.

^{1}H NMR (400 MHz, $CDCl_3$) δ 7.51 – 7.45 (m, 2H), 7.28 – 7.21 (m, 2H), 7.06 – 6.99 (m, 2H), 6.95 – 6.89 (m, 2H).
^{13}C NMR (101 MHz, $CDCl_3$) δ 156.9, 155.5, 144.9, 133.2, 123.0, 121.3, 119.7, 118.90 (q, *J* = 320.8 Hz), 117.1.
^{19}F NMR (377 MHz, $CDCl_3$) δ -72.64.
HRMS (EI) m/z: M^+ Calcd for $C_{13}H_8BrF_3O_4S$ 395.9273; Found 395.9268

quinolin-7-yl trifluoromethanesulfonate (see Figure S2)

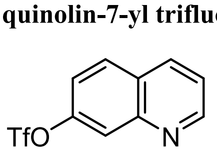

Prepared from 7-hydroxyquinoline (290 mg, 2mmol) according to general procedure A to afford a yellow solid (405 mg, 73%). Crude product of sufficient purity to be used immediately. Spectroscopic data consistent with literature.[11]

^{1}H NMR (400 MHz, $CDCl_3$) δ 9.00 (dd, J = 4.2, 1.7 Hz, 1H), 8.23 (dd, J = 8.4, 1.0 Hz, 1H), 8.04 (d, J = 2.5 Hz, 1H), 7.93 (d, J = 9.0 Hz, 1H), 7.53 – 7.46 (m, 2H).
^{13}C NMR (101 MHz, $CDCl_3$) δ 152.2, 149.8, 148.3, 136.3, 130.4, 127.7, 122.4, 121.3, 120.9, 118.9 (q, J = 320.9, 320.2 Hz).
^{19}F NMR (376 MHz, $CDCl_3$) δ -72.52.
MS (EI) m/z: M^+ Calcd for $C_{10}H_6F_3NO_3S$ 277.0; Found 277.0.

1-bromonaphthalen-2-yl trifluoromethanesulfonate (see Figure S2)

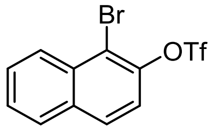

Prepared from 1-bromo-2-naphthol (446 mg mg, 2mmol) according to general procedure A to afford a yellow solid (384 mg, 54%). Crude product of sufficient purity to be used immediately. Spectroscopic data consistent with literature.[12]

^{1}H NMR (400 MHz, $CDCl_3$) δ 8.26 (d, J = 8.4 Hz, 1H), 7.83 (dd, J = 9.1, 2.6 Hz, 2H), 7.69 – 7.51 (m, 2H), 7.41 (d, J = 9.1 Hz, 1H).
^{13}C NMR (101 MHz, $CDCl_3$) δ 145.1, 133.0, 132.7, 129.8, 128.8, 128.4, 127.8, 127.7, 119.9, 118.8 (q, J = 320.6 Hz), 116.2, 68.0, 25.7.
^{19}F NMR (377 MHz, $CDCl_3$) δ -73.30.
MS (EI) m/z: M+ Calcd for $C_{11}H_6BrF_3O_3S$ 353.9; Found 353.9.

4-carbomethoxyphenyl trifluoromethanesulfonate

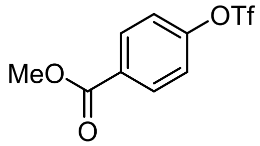

Prepared from methyl 4-hydroxybenzoate (304 mg, 2mmol) according to general procedure A to afford a colourless oil (297 mg, 52%). Crude product of sufficient purity to proced immediately. Spectroscopic data consistent with literature.[13]

^{1}H NMR (400 MHz, $CDCl_3$) δ 8.13 (d, J = 8.9 Hz, 2H), 7.34 (d, J = 9.0 Hz, 2H), 3.92 (s, 3H).
^{13}C NMR (101 MHz, $CDCl_3$) δ 165.5, 152.6, 132.0, 130.5, 121.5, 118.8 (q, J = 320.7 Hz), 68.1, 52.6, 25.7.
^{19}F NMR (376 MHz, $CDCl_3$) δ -72.75.
MS (EI) m/z: M+ Calcd for $C_9H_7F_3O_5S$ 284.0; Found 284.0.

7.2 Aryl boronic acid pinacol esters

4,4,5,5-tetramethyl-2-phenyl-1,3,2-dioxaborolane (3a)

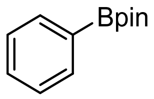

Isolated as a white solid, purified by column chromatography - general procedure B, (2% Et_2O in petroleum ether): 104 mg (51%). General procedure C (2% Et_2O in petroleum ether): 112 mg (55%). Spectroscopic data consistent with literature.[14]

^{1}H NMR (400 MHz, $CDCl_3$) δ 7.83 (dd, J = 1.5 Hz, 2H), 7.51 – 7.44 (m, 1H), 7.42 – 7.34 (m, 2H), 1.36 (s, 12H).
^{13}C NMR (101 MHz, $CDCl_3$) δ 134.9, 131.4, 127.8, 83.9, 25.0.
^{11}B NMR (128 MHz, $CDCl_3$) δ 30.02.
MS (EI) m/z: M^+ Calcd for $C_{12}H_{17}BO_2$ 204.13; Found 204.1.

4,4,5,5-tetramethyl-2-(o-tolyl)-1,3,2-dioxaborolane (3b)

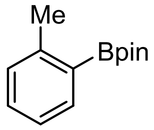

Isolated as a clear oil, purified by column chromatography - general procedure B (2% Et_2O in petroleum ether): 45 mg (41%). General procedure C (2% Et_2O in petroleum ether): 37 mg (34%). Spectroscopic data consistent with literature.[14]

^{1}H NMR (400 MHz, $CDCl_3$) δ 7.80 (dd, J = 7.7, 1.6 Hz, 1H), 7.34 (td, J = 7.5, 1.6 Hz, 1H), 7.25 – 7.13 (m, 2H), 2.58 (s, 3H), 1.38 (s, 12H).
^{13}C NMR (101 MHz, $CDCl_3$) δ 145.0, 136.0, 130.9, 129.9, 124.8, 83.5, 25.0, 22.3.
^{11}B NMR (128 MHz, $CDCl_3$) δ 30.72.
MS (EI) m/z: M^+ Calcd for $C_{13}H_{19}BO_2$ 218.15; Found 218.1.

4,4,5,5-tetramethyl-2-(m-tolyl)-1,3,2-dioxaborolane (3c)

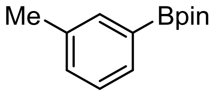

Isolated as a white solid, purified by column chromatography - general procedure B (2% Et_2O in petroleum ether): 51 mg (47%). General procedure C (2% Et_2O in petroleum ether): 44 mg (40%). Spectroscopic data consistent with literature.[15]

^{1}H NMR (400 MHz, $CDCl_3$) δ 7.70 – 7.57 (m, 2H), 7.29 (m, 2H), 2.37 (s, 3H), 1.36 (s, 12H).
^{13}C NMR (101 MHz, $CDCl_3$) δ 137.3, 135.5, 132.2, 131.9, 127.8, 83.8, 25.0, 21.4.
^{11}B NMR (128 MHz, $CDCl_3$) δ 29.77.
MS (EI) m/z: M^+ Calcd for $C_{13}H_{19}BO_2$ 218.15; Found 218.1.

4,4,5,5-tetramethyl-2-(p-tolyl)-1,3,2-dioxaborolane (3d)

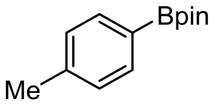

Isolated as a clear oil, purified by column chromatography - general procedure B (2% Et_2O in petroleum ether): 69 mg (63%). General procedure C (2% Et_2O in petroleum ether): 60 mg (55%). Spectroscopic data consistent with literature.[14]

^{1}H NMR (400 MHz, $CDCl_3$) δ 7.73 (d, J = 7.9 Hz, 2H), 7.20 (d, J = 7.5 Hz, 2H), 2.38 (s, 3H), 1.35 (s, 12H).
^{13}C NMR (101 MHz, $CDCl_3$) δ 141.5, 134.9, 128.6, 83.7, 25.0, 21.8.
^{11}B NMR (128 MHz, $CDCl_3$) δ 29.98.
MS (EI) m/z: M^+ Calcd for $C_{13}H_{19}BO_2$ 218.15; Found 218.1.

2-(4-methoxyphenyl)-4,4,5,5-tetramethyl-1,3,2-dioxaborolane (3e)

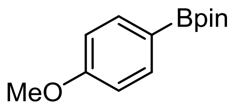

Isolated as a clear oil, purified by column chromatography - general procedure B (5-10% Et_2O in petroleum ether): 54 mg (46%), general procedure C (5-7.5% Et_2O in petroleum ether): 45 mg (38%). Spectroscopic data consistent with literature.[14]

^{1}H NMR (400 MHz, $CDCl_3$) δ 7.77 (d, J = 8.6 Hz, 2H), 6.90 (d, J = 8.7 Hz, 2H), 3.83 (s, 3H), 1.34 (s, 12H).
^{13}C NMR (101 MHz, $CDCl_3$) δ 162.3, 136.6, 113.4, 83.7, 55.2, 25.0.
^{11}B NMR (128 MHz, $CDCl_3$) δ 29.91.
MS (EI) m/z: M^+ Calcd for $C_{13}H_{19}BO_3$ 234.14; Found 234.1.

2-(4-fluorophenyl)-4,4,5,5-tetramethyl-1,3,2-dioxaborolane (3f)

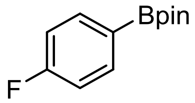

Isolated as a white solid, purified by column chromatography – general procedure B (5% Et_2O in petroleum ether): 20 mg (18%, 1 : 0.14 ratio of title product : ^{t}BuBpin), general procedure C (2% Et_2O in petroleum ether): 29 mg (24%). Spectroscopic data consistent with literature.[14]

^{1}H NMR (400 MHz, $CDCl_3$) δ 7.92 – 7.61 (m, 2H), 7.09 – 7.01 (m, 2H), 1.34 (s, 12H).
^{13}C NMR (101 MHz, $CDCl_3$) δ 166.5, 164.0, 137.2, 137.10, 115.1, 114.9, 84.1, 25.0.
^{19}F NMR (377 MHz, $CDCl_3$) δ -108.35.
^{11}B NMR (128 MHz, $CDCl_3$) δ 30.09.
MS (EI) m/z: M^+ Calcd for $C_{12}H_{16}BFO_2$ 222.12; Found 222.1.

4,4,5,5-tetramethyl-2-(4-(trifluoromethyl)phenyl)-1,3,2-dioxaborolane (3g)

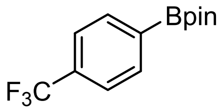

Isolated as a clear oil, purified by column chromatography – general procedure B (5% Et_2O in petroleum ether): 52 mg (38%), general procedure C (5% Et_2O in petroleum ether): 72 mg (53%). Spectroscopic data consistent with literature.[16]

^{1}H NMR (400 MHz, $CDCl_3$) δ 7.91 (d, J = 7.7 Hz, 2H), 7.61 (d, J = 7.6 Hz, 2H), 1.36 (s, 12H).
^{13}C NMR (101 MHz, $CDCl_3$) δ 135.2, 133.0 (q, J = 32.2 Hz), 124.5 (q, J = 3.9 Hz), 124.3 (q, J = 272.4 Hz), 84.4, 25.0.
^{19}F NMR (376 MHz, $CDCl_3$) δ -62.92.
^{11}B NMR (128 MHz, $CDCl_3$) δ 29.67.
MS (EI) m/z: M^+ Calcd for $C_{13}H_{16}BF_3O_2$ 272.12; Found 272.1.

4,4,5,5-tetramethyl-2-(naphthalen-1-yl)-1,3,2-dioxaborolane (3h)

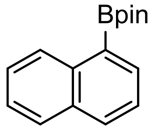

Isolated as a white solid, purified by column chromatography – general procedure B (2.5-5% Et_2O in petroleum ether): 99 mg (78%), general procedure C (5% Et_2O in petroleum ether): 76 mg (55%). Spectroscopic data consistent with literature.[17]

^{1}H NMR (400 MHz, $CDCl_3$) δ 8.82 (d, J = 8.4 Hz, 1H), 8.13 (dd, J = 6.8, 1.4 Hz, 1H), 7.97 (d, J = 8.2 Hz, 1H), 7.93 – 7.81 (m, 1H), 7.61 – 7.54 (m, 1H), 7.54 – 7.43 (m, 2H), 1.46 (s, 13H).
^{13}C NMR (101 MHz, $CDCl_3$) δ 137.1, 135.8, 133.4, 131.7, 128.6, 128.5, 126.5, 125.6, 125.1, 83.8, 25.1.
^{11}B NMR (128 MHz, $CDCl_3$) δ 30.67.
MS (EI) m/z: M^+ Calcd for $C_{16}H_{19}BO_2$ 254.15; Found 254.1.

4,4,5,5-tetramethyl-2-(naphthalen-2-yl)-1,3,2-dioxaborolane (3i)

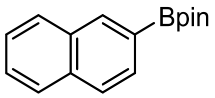

Isolated as a white solid, purified by column chromatography – general procedure B (5% Et_2O in petroleum ether): 79 mg (62%). Spectroscopic data consistent with literature.[18]

^{1}H NMR (400 MHz, $CDCl_3$) δ 8.43 (s, 1H), 7.98 – 7.77 (m, 4H), 7.63 – 7.40 (m, 2H), 1.42 (s, 12H).
^{13}C NMR (101 MHz, $CDCl_3$) δ 136.4, 135.2, 133.0, 130.5, 128.8, 127.8, 127.1, 125.9, 84.0, 25.0.
^{11}B NMR (128 MHz, $CDCl_3$) δ 30.16.
MS (EI) m/z: M^+ Calcd for $C_{16}H_{19}BO_2$ 254.15; Found 254.1.

2-(3,5-dimethoxyphenyl)-4,4,5,5-tetramethyl-1,3,2-dioxaborolane (3j)

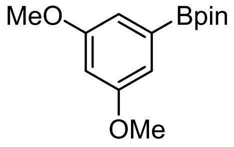

Did not isolate – general procedure B. Spectroscopic yield: 24%.

N,N-dimethyl-3-(4,4,5,5-tetramethyl-1,3,2-dioxaborolan-2-yl)aniline (3k)

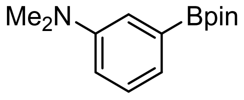

Isolated as a clear oil, purified by column chromatography (5-20% EtOAC in hexane) – general procedure B: 41 mg (33%). Spectroscopic data consistent with literature.[19]

^{1}H NMR (400 MHz, $CDCl_3$) δ 7.27 (t, J = 7.6 Hz, 1H), 7.23 – 7.16 (m, 2H), 6.87 (m, 2H), 2.97 (s, 6H), 1.35 (s, 12H).
^{13}C NMR (101 MHz, $CDCl_3$) δ 150.3, 128.6, 123.4, 118.8, 115.9, 83.7, 40.9, 25.0.
^{11}B NMR (128 MHz, $CDCl_3$) δ 30.00.
MS (ESI) m/z: $[M+H]^+$ Calcd for $C_{14}H_{23}BNO_2$ 247.17; Found 248.18

ethyl 4-(4,4,5,5-tetramethyl-1,3,2-dioxaborolan-2-yl)benzoate (3l)

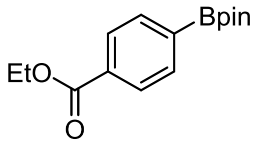

Isolated as a yellow oil, purified by column chromatography – general procedure B (7.5% EtOAc in hexane): 39 mg (28%). Spectroscopic data consistent with literature.[19]

^{1}H NMR (400 MHz, $CDCl_3$) δ 8.02 (d, J = 8.4 Hz, 2H), 7.86 (d, J = 8.4 Hz, 2H), 4.38 (q, J = 7.1 Hz, 2H), 1.40 (t, J = 7.1 Hz, 3H), 1.35 (s, 12H).

^{13}C NMR (101 MHz, $CDCl_3$) δ 166.8, 134.8, 132.8, 128.7, 84.3, 61.2, 25.0, 14.5.
^{11}B NMR (128 MHz, $CDCl_3$) δ 30.18.
MS (EI) m/z: M^+ Calcd for $C_{15}H_{21}BO_4$ 276.15; Found 276.1530.

N,N-dimethyl-4-(4,4,5,5-tetramethyl-1,3,2-dioxaborolan-2-yl)benzamide (3m)

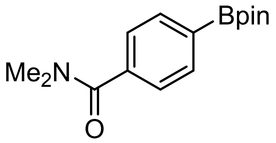

Isolated as a white solid, purified by column chromatography – general procedure B (70% EtOAc in hexane): 57 mg (41%). Spectroscopic data consistent with literature.[20]

^1H NMR (400 MHz, $CDCl_3$) δ 7.81 (d, J = 8.2 Hz, 2H), 7.37 (d, J = 8.1 Hz, 2H), 3.08 (s, 3H), 2.92 (s, 3H), 1.32 (s, 12H).
^{13}C NMR (101 MHz, $CDCl_3$) δ 171.6, 138.9, 134.8, 126.3, 84.1, 39.5, 35.4, 24.9.
^{11}B NMR (128 MHz, $CDCl_3$) δ 28.72.
HRMS (EI) m/z: M^+ Calcd for $C_{15}H_{22}BNO_3$ 275.1693; Found 274.1605 $[M-H]^+$

4-(4,4,5,5-tetramethyl-1,3,2-dioxaborolan-2-yl)aniline (3n)

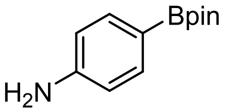

Did not isolate – general procedure B. Spectroscopic yield: 24%

2-methyl-6-(4,4,5,5-tetramethyl-1,3,2-dioxaborolan-2-yl)quinoline (3o)

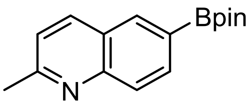

Isolated as a white solid, purified by column chromatography – general procedure B (20% EtOAC in hexane): 74 mg (55%). Spectroscopic data consistent with literature.[21]

^1H NMR (400 MHz, $CDCl_3$) δ 8.26 (d, J = 1.4 Hz, 1H), 8.03 (dd, J = 8.5, 1.4 Hz, 1H), 8.02 (d, J = 8.4 Hz, 1H), 7.97 (d, J = 8.5 Hz, 1H), 7.23 (d, J = 8.4 Hz, 1H), 2.71 (s, 3H), 1.35 (s, 12H).
^{13}C NMR (101 MHz, $CDCl_3$) δ 160.2, 149.5, 136.8, 135.9, 134.4, 127.8, 126.0, 122.1, 84.1, 25.6, 25.0.
^{11}B NMR (128 MHz, $CDCl_3$) δ 30.06.
MS (EI) m/z: M^+ Calcd for $C_{16}H_{20}BNO_2$ 269.16; Found 269.2

6-(4,4,5,5-tetramethyl-1,3,2-dioxaborolan-2-yl)quinoline (3p)

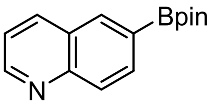

Could not be separated – general procedure B. Spectroscopic yield: 38%

3-(4,4,5,5-tetramethyl-1,3,2-dioxaborolan-2-yl)pyridine (3q)

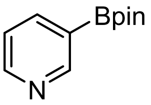

Could not be separated – general procedure B. Spectroscopic yield: 92%

trimethyl(4-(4,4,5,5-tetramethyl-1,3,2-dioxaborolan-2-yl)phenyl)silane (3r)

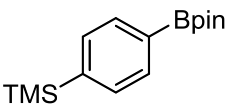

Isolated as a clear oil, purified by column chromatography – general procedure B (2% Et_2O in petroleum ether): 57 mg (41%). Spectroscopic data consistent with literature.[18]

^{1}H NMR (400 MHz, $CDCl_3$) δ 7.81 (d, *J* = 7.8 Hz, 2H), 7.55 (d, *J* = 7.8 Hz, 2H), 1.36 (s, 12H), 0.29 (s, 9H).
^{13}C NMR (101 MHz, $CDCl_3$δ 144.4, 134.0, 132.7, 83.9, 77.5, 77.2, 76.8, 25.0, -1.1.
^{11}B NMR (128 MHz, $CDCl_3$) δ 30.11.
MS (EI) m/z: M^+ Calcd for $C_{15}H_{25}BO_2Si$ 276.17; Found 276.2.

2-(benzo[d][1,3]dioxol-5-yl)-4,4,5,5-tetramethyl-1,3,2-dioxaborolane (3s)

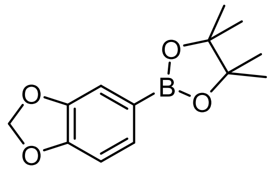

Isolated as a clear oil, purified by column chromatography – general procedure B (5% EtOAc in hexane): 74 mg (59%). Spectroscopic data consistent with literature.[22]

^{1}H NMR (400 MHz, $CDCl_3$) δ 7.36 (dd, *J* = 7.7, 1.2 Hz, 1H), 7.24 (d, *J* = 1.1 Hz, 1H), 6.83 (d, *J* = 7.7 Hz, 1H), 5.95 (s, 2H), 1.33 (s, 12H).
^{13}C NMR (101 MHz, $CDCl_3$) δ 150.3, 147.3, 129.8, 114.1, 108.4, 100.8, 83.8, 25.0.
^{11}B NMR (128 MHz, $CDCl_3$) δ 29.58.
MS (EI) m/z: M^+ Calcd for $C_{13}H_{17}BO_4$ 248.12; Found 248.1.

1,4-bis(4,4,5,5-tetramethyl-1,3,2-dioxaborolan-2-yl)benzene (3t)

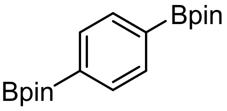

Isolated as a white solid, purified by column chromatography – general procedure B (12.5% EtOAc in hexane): 73 mg (47%). Spectroscopic data consistent with literature.[18]

^{1}H NMR (400 MHz, Chloroform-*d*) δ 7.80 (s, 4H), 1.35 (s, 24H).
^{13}C NMR (101 MHz, Chloroform-*d*) δ 134.0, 84.0, 25.0.
^{11}B NMR (128 MHz, CHLOROFORM-D) δ 30.32.
MS (EI) m/z: M^+ Calcd for $C_{18}H_{28}B_2O_4$ 330.22; Found 330.2.

2-([1,1'-biphenyl]-4-yl)-4,4,5,5-tetramethyl-1,3,2-dioxaborolane (3u)

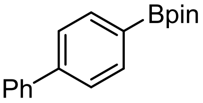

Isolated as a white solid, purified by column chromatography – general procedure C (5% Et_2O in petroleum ether): 77 mg (55%). Spectroscopic data consistent with literature.[23]

^{1}H NMR (400 MHz, Chloroform-*d*) δ 7.94 (d, *J* = 8.1 Hz, 2H), 7.66 (d, *J* = 8.2 Hz, 4H), 7.48 (t, *J* = 7.7 Hz, 2H), 7.39 (t, *J* = 7.4 Hz, 1H), 1.40 (s, 12H).
^{11}B NMR (128 MHz, CHLOROFORM-*D*) δ 30.09.
^{13}C NMR (101 MHz, CHLOROFORM-*D*) δ 144.0, 141.1, 135.4, 128.9, 127.7, 127.3, 126.6, 83.9, 25.0.
MS (ESI) m/z: Calcd for $C_{18}H_{21}BO_2$ 280.16; Found 281.17 $[M+H]^+$

2-(3,5-difluorophenyl)-4,4,5,5-tetramethyl-1,3,2-dioxaborolane (3v)

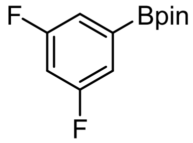

Could not isolate – general procedure C. Spectroscopic yield: 43%.

2-(3,5-di-tert-butylphenyl)-4,4,5,5-tetramethyl-1,3,2-dioxaborolane (3w)

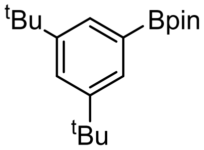

Did not isolate – general procedure C. Spectroscopic yield: 17%

8 Radical probe experiments

8.1 General procedure for radical probe experiments

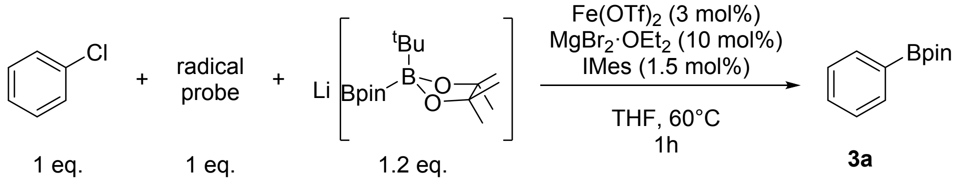

Inside an argon-filled glovebox, a 7 mL vial fitted with a stirrer bar was charged with $Fe(OTf)_2$ (5.3 mg, 0.015 mmol, 0.03 equiv.), IMes (2.6 mg, 0.008 mmol, 0.015 equiv.) and THF (1 mL) and stirred at 60 °C (aluminium heating block, external temperature) for 30 mins. Chlorobenzene (56 mg, 0.25 mmol, 1 equiv.), $MgBr_2{\cdot}OEt_2$ (13 mg, 0.05 mmol, 0.1 equiv.), the appropriate radical probe (0.5 mmol, 1 equiv.) and THF (2 mL) were added and the reaction stirred at 60 °C for a further 15 min. The boronate **1** (190 mg, 0.5 mmol, 1.2 equiv.) was added along with a further 1 mL THF (total reaction volume = 4 mL, 0.125 M wrt chlorobenzene) and the reaction stirred for 1h at 60 °C. The vial was then removed from the glovebox and cooled to room temperature. Mesitylene (60 mg, 0.5 mmol, 1 equiv.) or 1,3,5-trimethoxybenzene (84 mg, 0.5 mmol, 1 equiv.) was added as internal standard and an aliquot was filtered through a celite pad, which washed with hexane. The combined organics were dried *in vacuo* and the sample analyzed by ^{1}H NMR spectroscopy and GCMS.

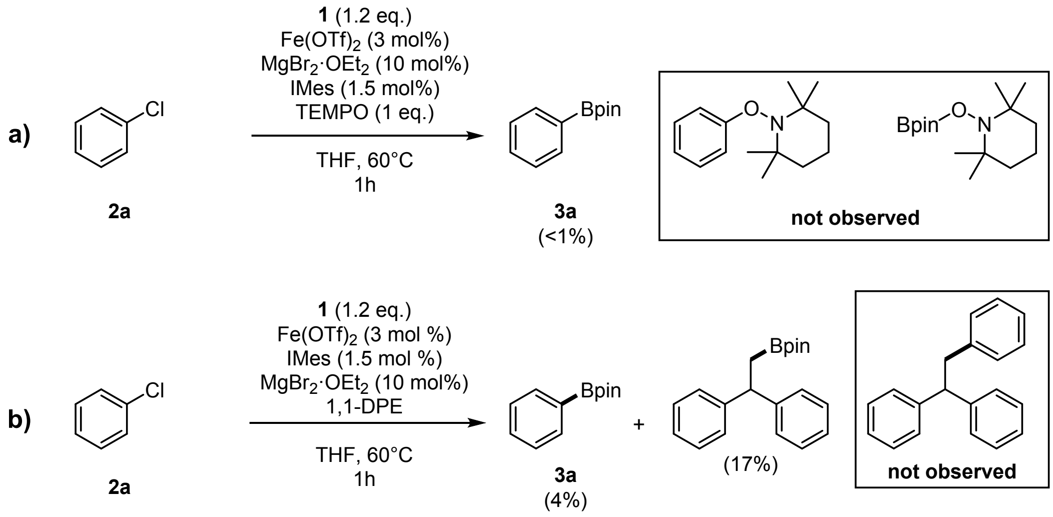

Scheme S1. Radical probe experiments with **a)** (2,2,6,6-Tetramethylpiperidin-1-yl)oxyl (TEMPO) **b)** 1,1-diphenylethylene (1,1-DPE). Conditions: **1** (0.6 mmol), **2** (0.5 mmol), appropriate radical probe (0.5 mmol) $MgBr_2{\cdot}OEt_2$ (0.1 mmol), $Fe(OTf)_2$ (0.015 mmol), ligand (0.008 mmol), THF, 60 °C, 1h (after addition of boronate **1**). Yields determined by ^{1}H NMR of the crude reaction mixture with 1,3,5-trimethoxybenzene (0.5 mmol) or mesitylene (0.50 mmol) as internal standard. [a]Yield of borylation product shown.

8.2 Data from radical probe experiments

8.2.1 Reaction with TEMPO

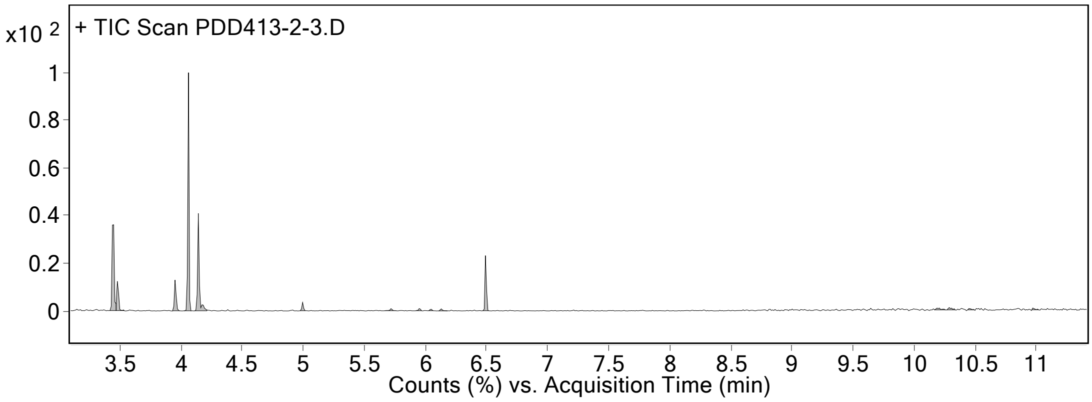

Figure S3.1. GCMS chromatogram of the radical probe experiment with TEMPO.

Table S6. Assignment of peaks in Figure S3.1

RT (min)	m/z (% relative intensity, ion)	Area (%)	Identity of compound	Mass spectrum
3.449	112.0 (100, M^+) 77.05 (65, $[M-Cl]^+$)	66.56	Chlorobenzene	Figure S3.2
3.479	85.2 (9), 59.1 (100)	18.82	Pinacol	Figure S3.3
3.951	125.99 (100), 70.10 (71)	17.79	Tetramethylpiperidine	Figure S3.4
4.061	169.1(76, $[M-Me]^+$), 129.1 (50), 83.09 (100)	100	^{t}BuBpin	Figure S3.5
4.142	120.1 (38), 105.12 (100)	50.21	Mesitylene (NMR internal standard)	Figure S3.6
4.995	140.8 (19), 119.2 (24), 69.41 (52), 56.4(27), 55.01 (100)	4.07	Not determined	Figure S3.7
5.718	205.1 (11), 181.2 (11), 142.27 (100), 58.9 (49)	1.68	Not determined	Figure S3.8
5.949	189.2 (58, $[M-Me]^+$), 147.2 (49), 118.04 (100)	1.25	Phenyl Bpin **3a**	Figure S3.9
6.492	142.4 (60), 142.1 (38), 109.16 (10), 71.1 (100)	27.69	TEMPOH[24]	Figure S3.10

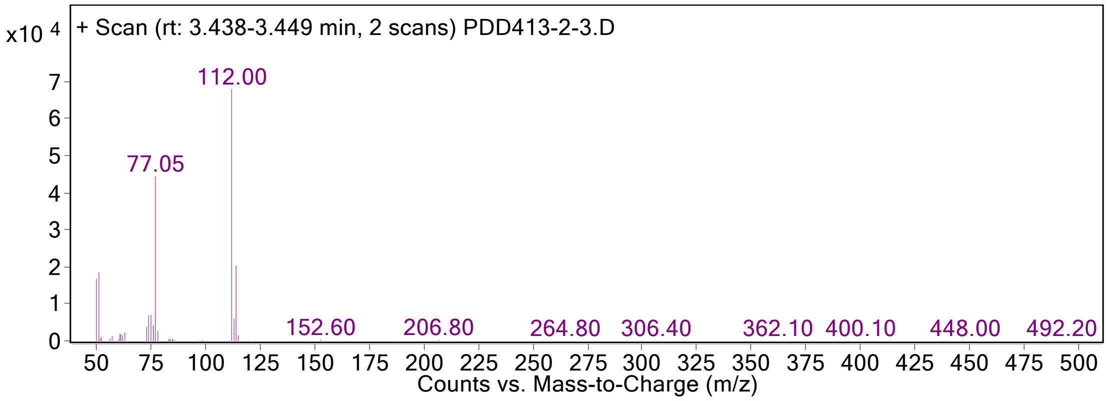

Figure S3.2. Mass-spectrum of the peak at 3.449 min (see Figure S3.1)

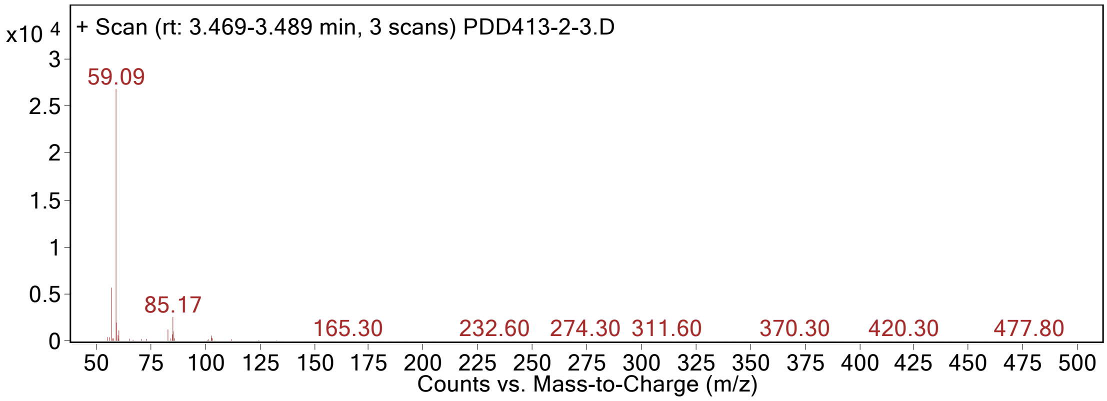

Figure S3.3. Mass-spectrum of the peak at 3.479 min (see Figure S3.1)

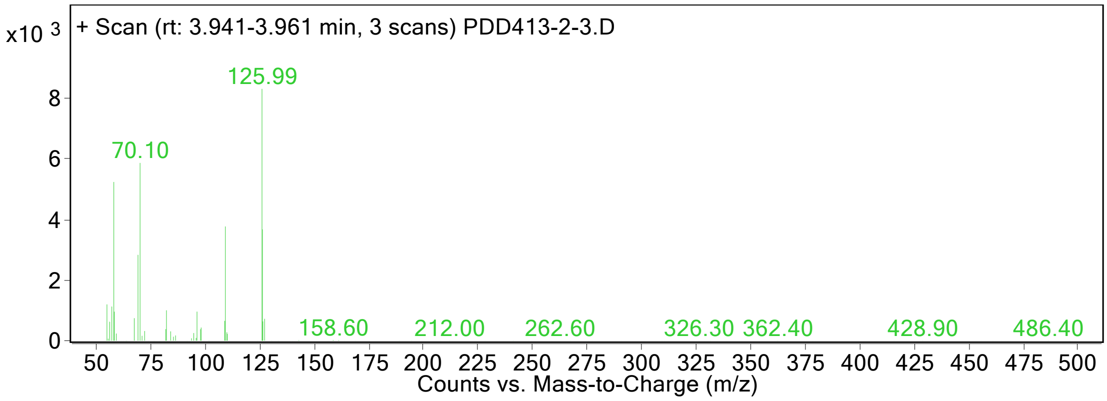

Figure S3.4. Mass-spectrum of the peak at 3.951 min (see Figure S3.1)

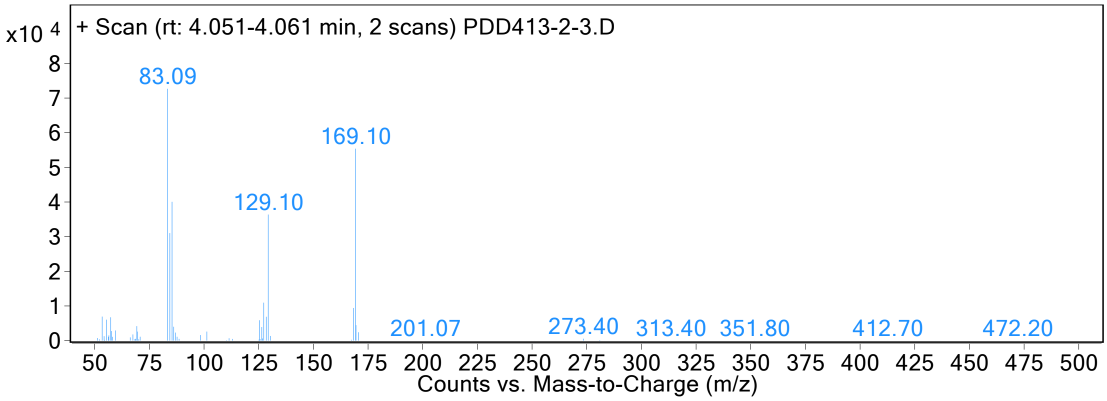

Figure S3.5. Mass-spectrum of the peak at 4.061 min (see Figure S3.1)

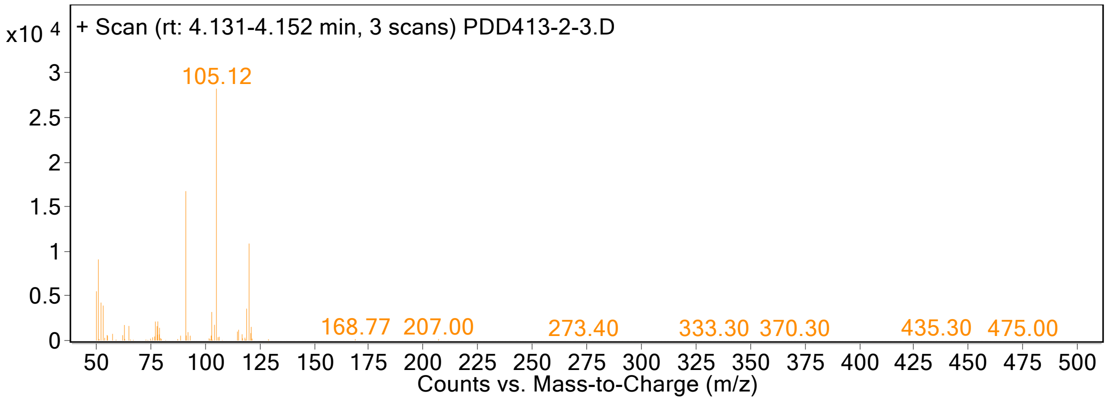

Figure S3.6. Mass-spectrum of the peak at 4.142 min (see Figure S3.1)

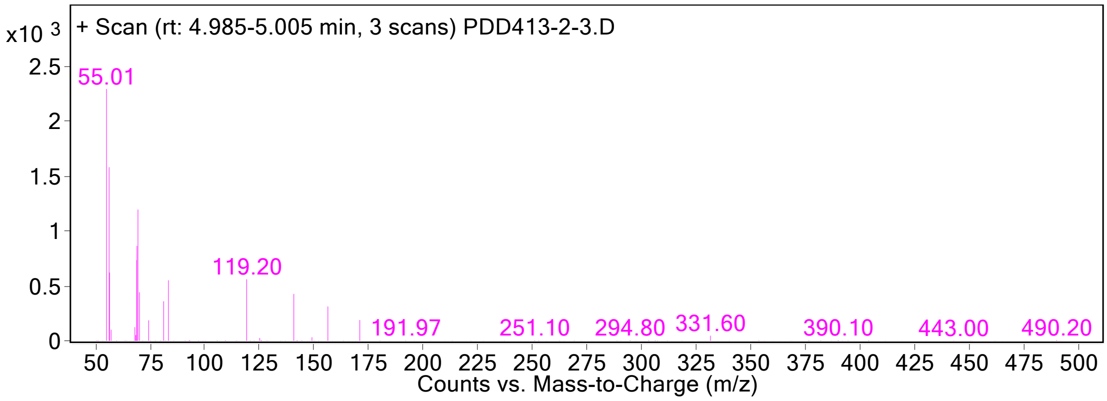

Figure S3.7. Mass-spectrum of the peak at 4.995 min (see Figure S3.1)

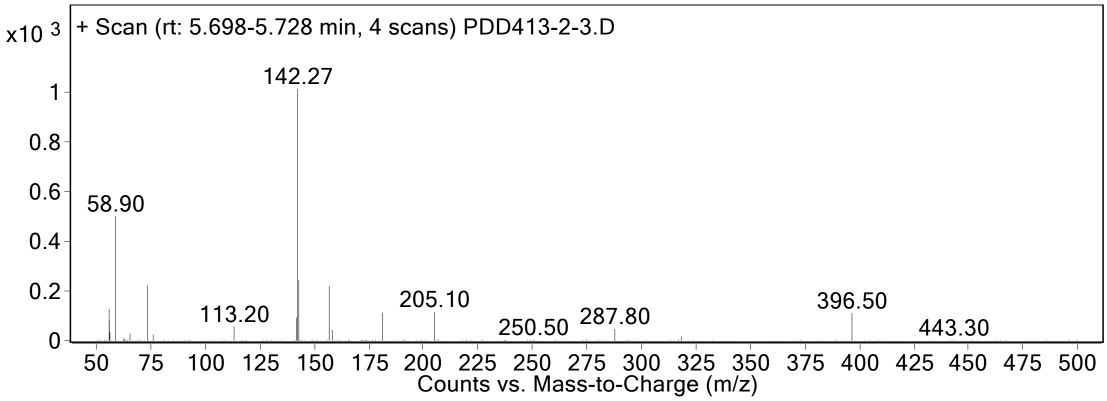

Figure S3.8. Mass-spectrum of the peak at 5.718 min (see Figure S3.1)

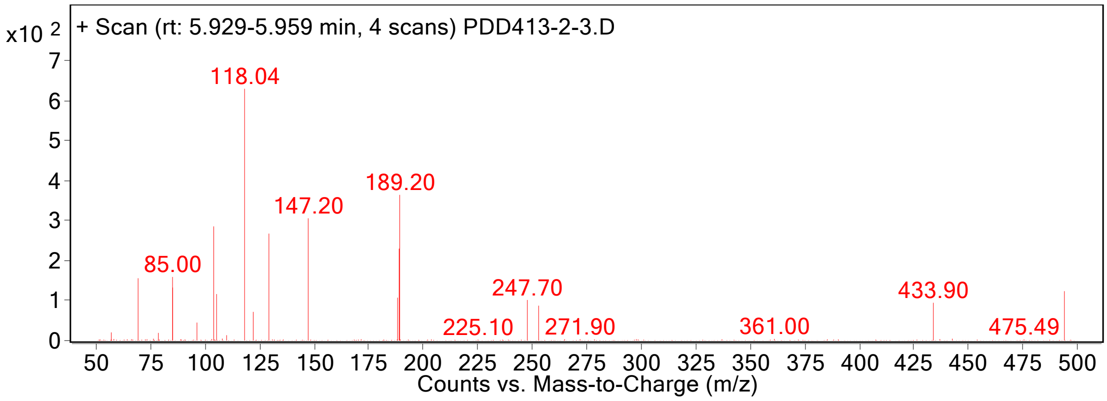

Figure S3.9. Mass-spectrum of the peak at 5.949 min (see Figure S3.1)

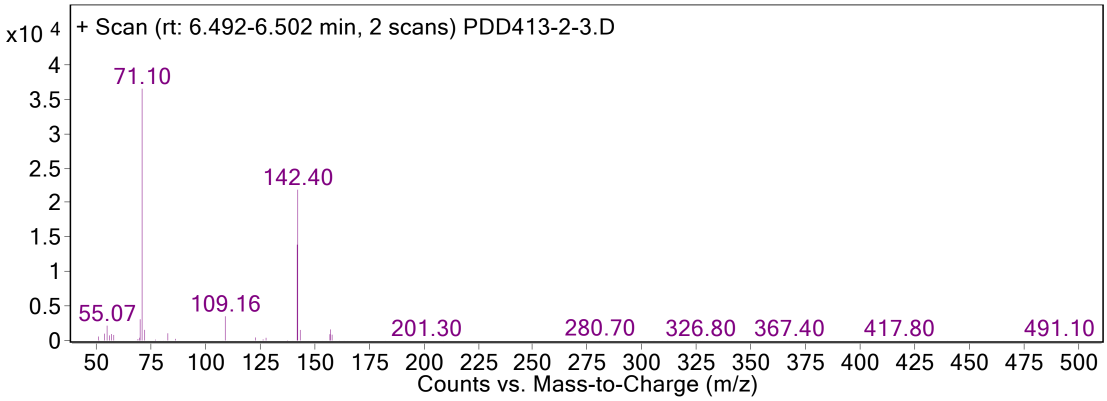

Figure S3.10. Mass-spectrum of the peak at 6.492 min (see Figure S3.1)

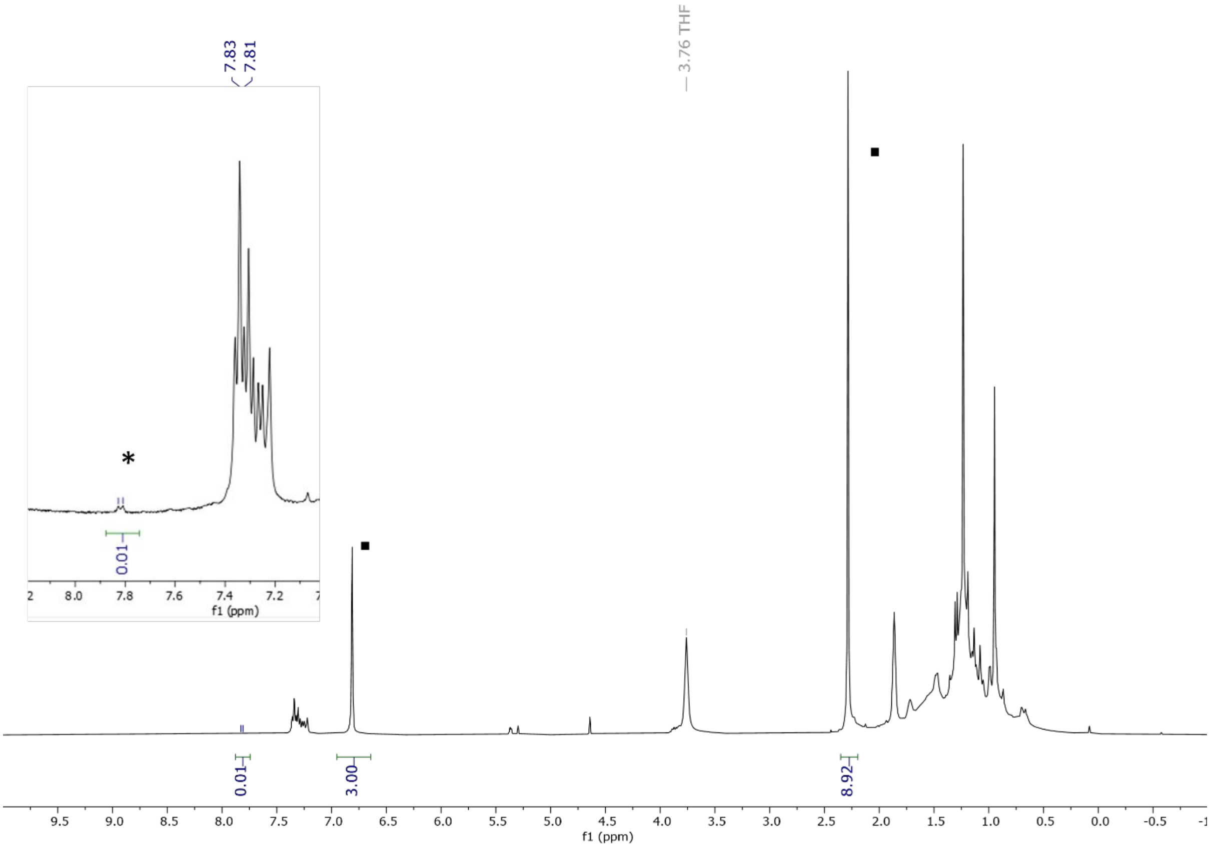

Figure S4. ^{1}H NMR spectrum of the crude reaction mixture from the reaction in the presence of TEMPO. Internal standard (mesitylene, "▪", 2H) and phenyl Bpin **3a** ("*", 2H) highlighted.

8.2.2 Reaction with 1,1-DPE

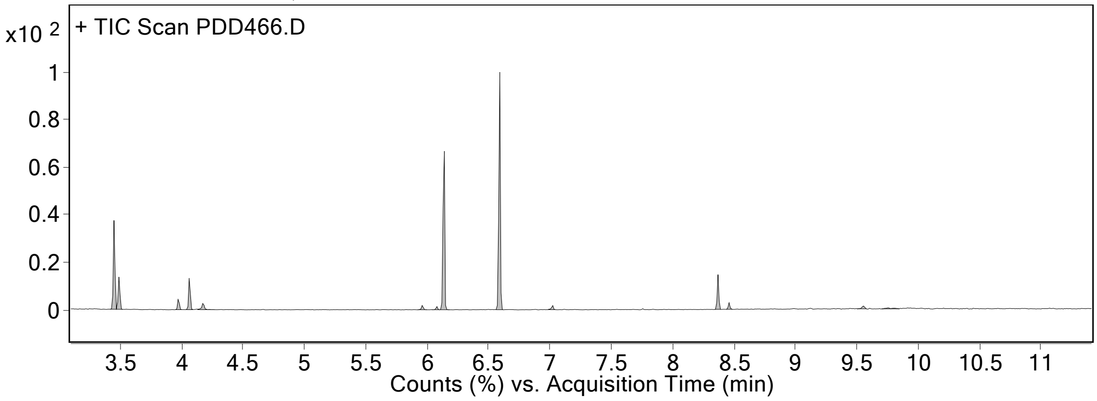

Figure S5.1 GCMS chromatogram of the radical probe experiment with 1,1-DPE.

Table S7. Assignment of peaks in Figure S5.1.

RT (min)	m/z (% relative intensity, ion)	Area (%)	Identity of compound	Mass spectrum
3.448	112.0 (100, M^+), 77.1 (65, $[M\text{-}Cl]^+$	39.73	Chlorobenzene	Figure S5.1
3.489	85.1 (17), 59.1 (100)	15.76	Pinacol	Figure S5.2
3.971	143.00 (85), 101.00 (42), 85.00 (24), 59.10 (100)	5.21	Not determined	Figure S5.3

4.061	169.2 (54, [M-Me]$^+$), 129.1 (53), 83.1 (100)	14.81	^{t}BuBpin	Figure S5.4
4.172	129.0 (100), 84.9 (21), 59.0 (34)	4.8	Not determined	Figure S5.5
5.959	204.1 (25, M$^+$), 188.9 (53), 105.1 (100)	2.25	Phenyl Bpin **3a**	Figure S5.6
6.09	154.1 (100, M$^+$), 97.2 (31), 57.3 (34)	n.d.	biphenyl	Figure S5.7
6.14	167.90 (58, M$^+$), 139.0 (100)	78.69	1,3,5-trimethoxybenzene (NMR internal standard)	Figure S5.8
6.592	180.1 (80, M$^+$), 179.1 (100, [M-H]$^+$), 165.2 (94), 77.1 (22, [Ph]$^+$)	100	1,1-DPE	Figure S5.9
7.014	194.9 (90), 165.1 (100), 105.1 (78)	n.d.	Not determined	Figure S5.10
8.37	308.3 (M$^+$) , 208.1 (6), 167.1 (100, [M-CH_2Bpin]$^+$), 84.1 (36)	14.48	Hydroboration product 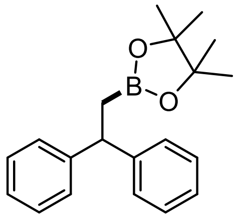	Figure S5.11
8.46	306.2 (45), 190.1 (100), 165.2 (14), 103.0 (19)	2.87	Not determined	Figure S5.12
9.555	306.1 (100), 206.9 (47), 167.	n.d.	Not determined	Figure S5.13

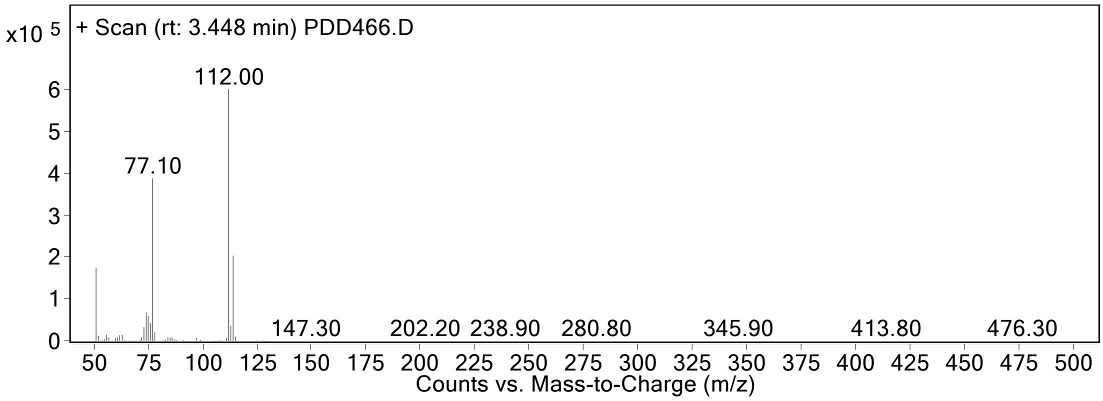

Figure S5.2. Mass-spectrum of the peak at 3.448 min (see Figure S5.1)

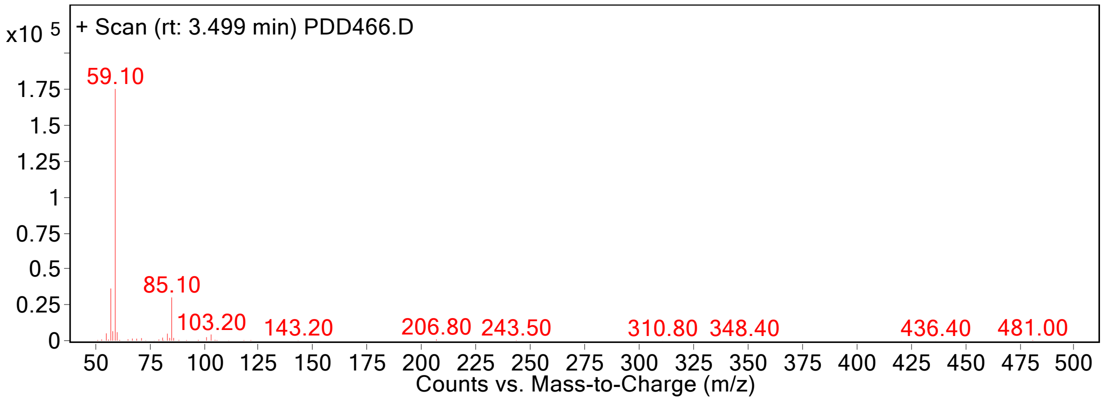

Figure S5.3. Mass-spectrum of the peak at 3.499 min (see Figure S5.1)

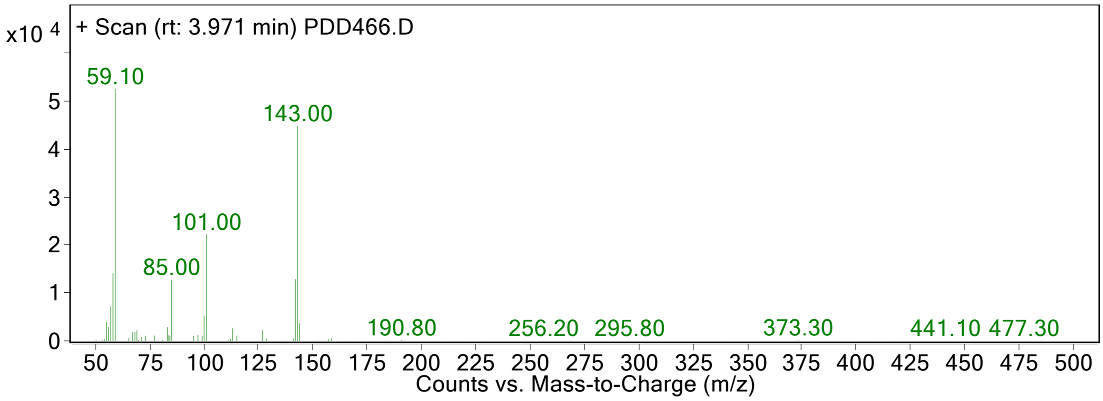

Figure S5.4. Mass-spectrum of the peak at 3.971 min (see Figure S5.1)

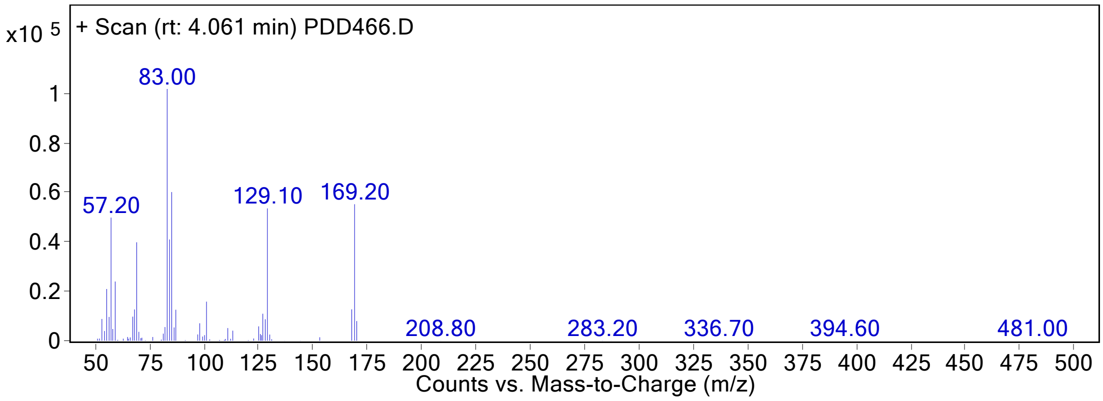

Figure S5.5. Mass-spectrum of the peak at 4.061 min (see Figure S5.1)

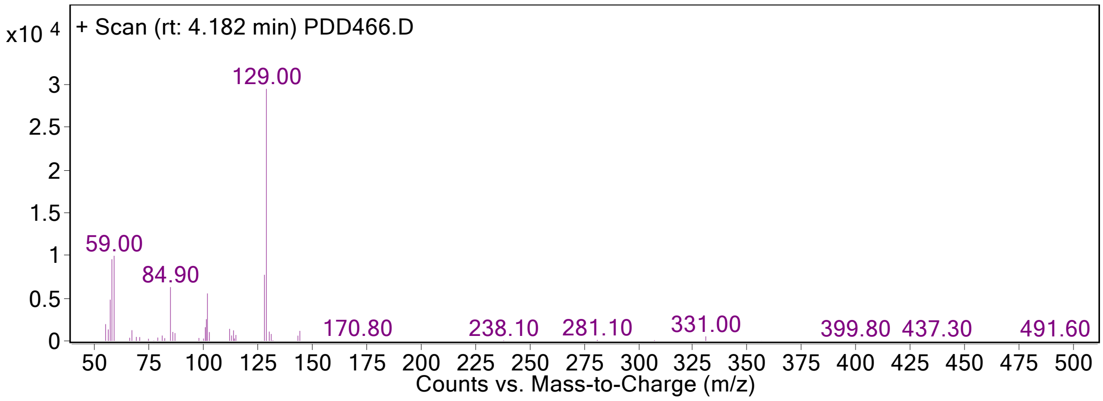

Figure S5.6. Mass-spectrum of the peak at 4.182 min (see Figure S5.1)

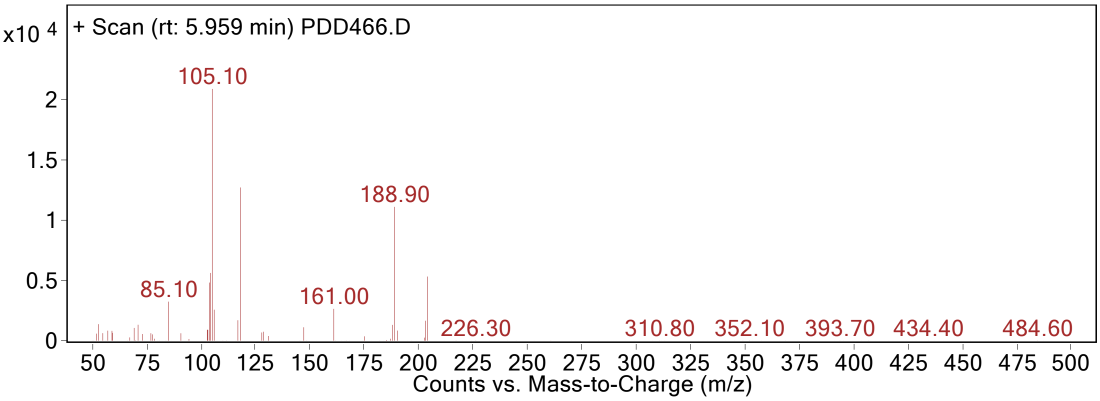

Figure S5.7. Mass-spectrum of the peak at 5.959 min (see Figure S5.1)

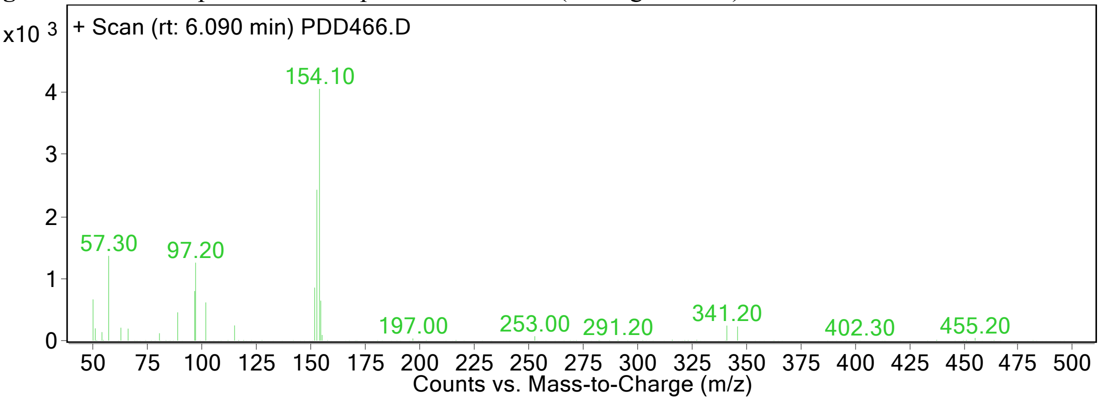

Figure S5.8. Mass-spectrum of the peak at 6.090 min (see Figure S5.1)

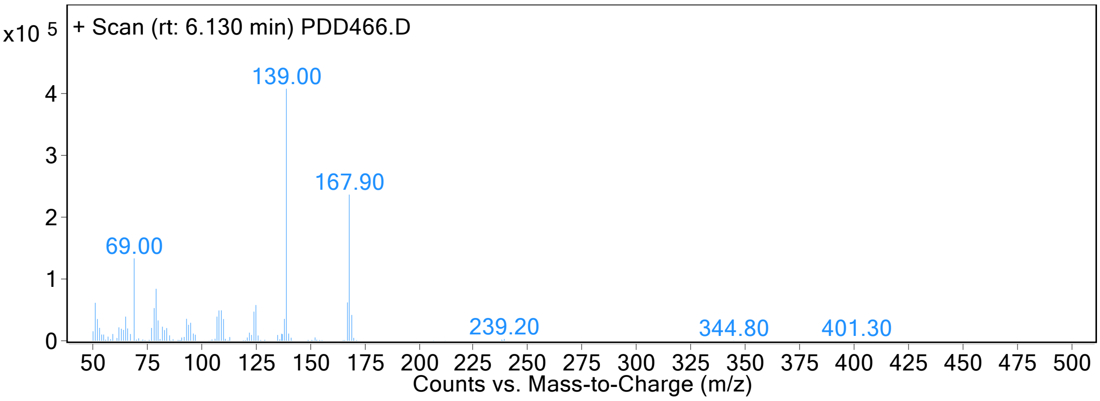

Figure S5.9. Mass-spectrum of the peak at 6.130 min (see Figure S5.1)

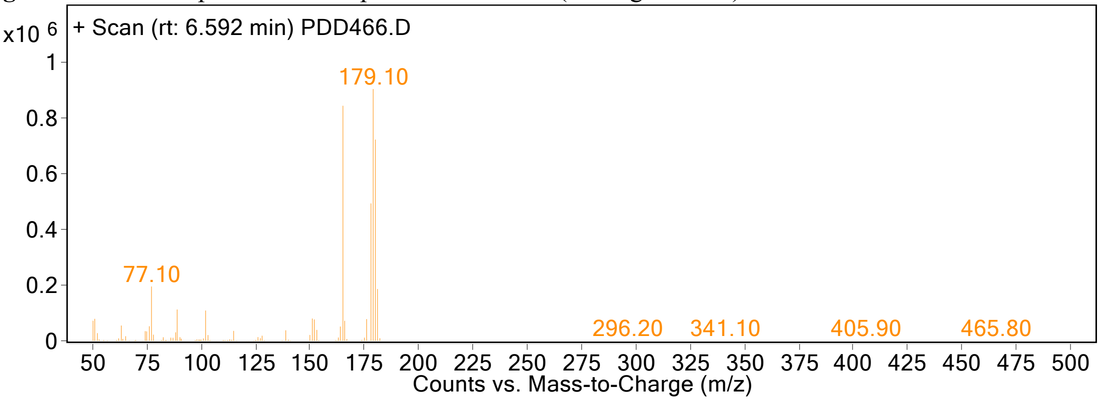

Figure S5.10. Mass-spectrum of the peak at 6.592 min (see Figure S5.1)

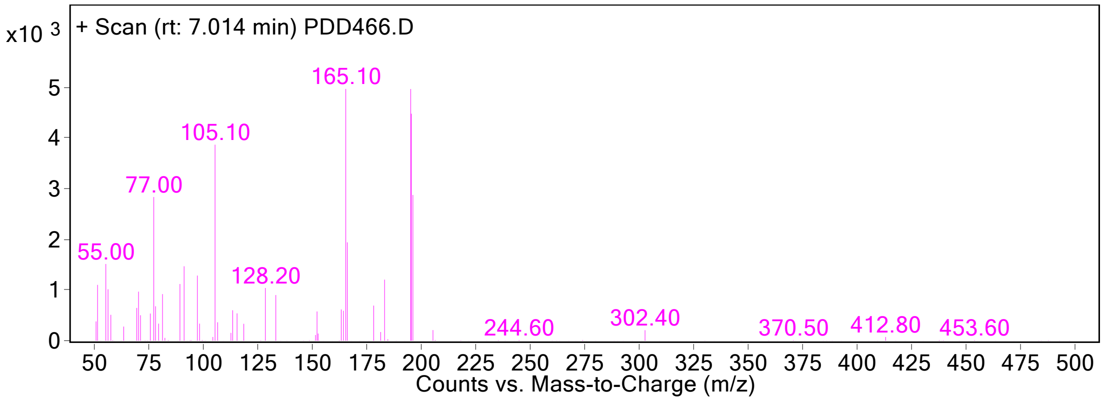

Figure S5.11. Mass-spectrum of the peak at 7.014 min (see Figure S5.1)

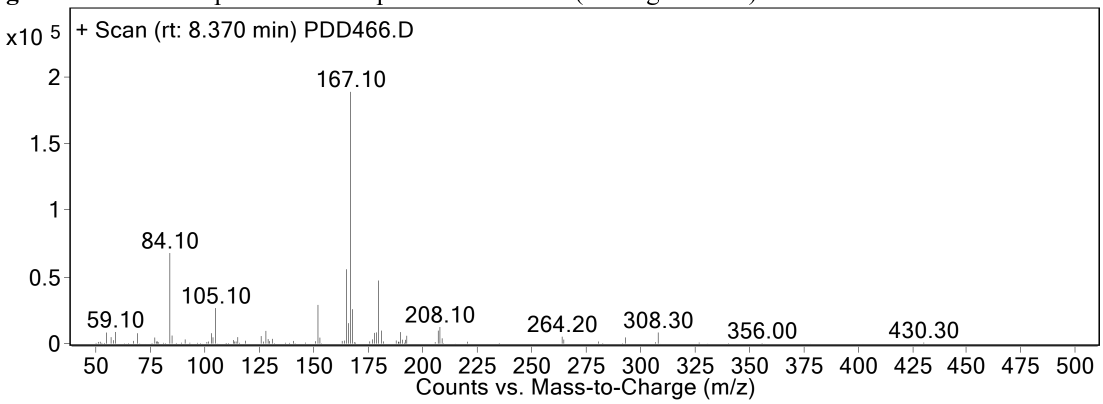

Figure S5.12. Mass-spectrum of the peak at 8.370 min (see Figure S5.1)

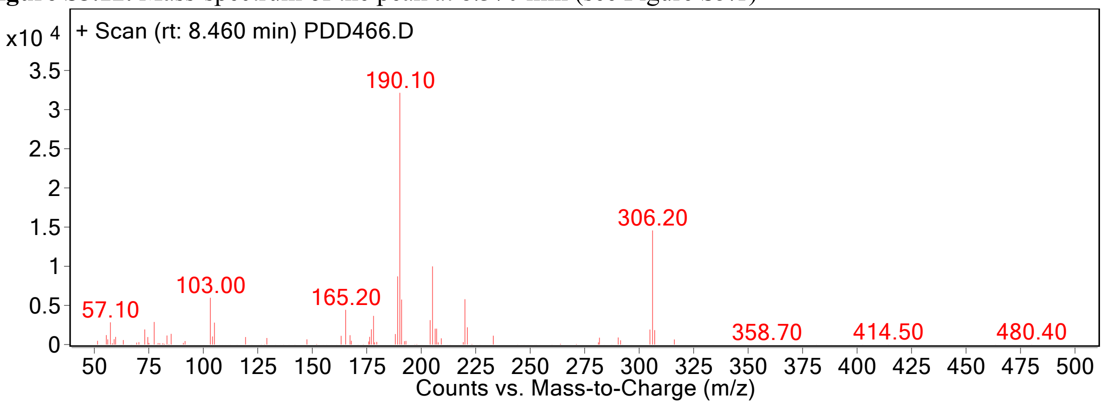

Figure S5.13. Mass-spectrum of the peak at 8.460 min (see Figure S5.1)

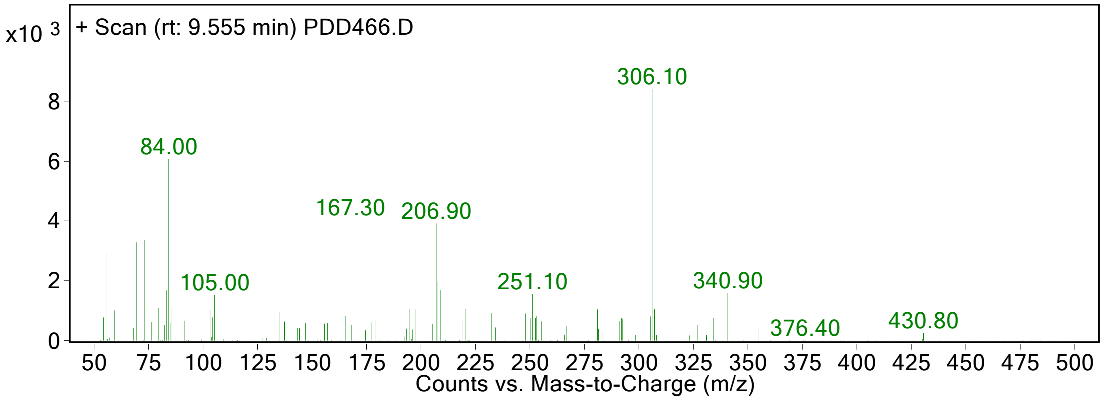

Figure S5.14. Mass-spectrum of the peak at 9.555 min (see Figure S5.1)

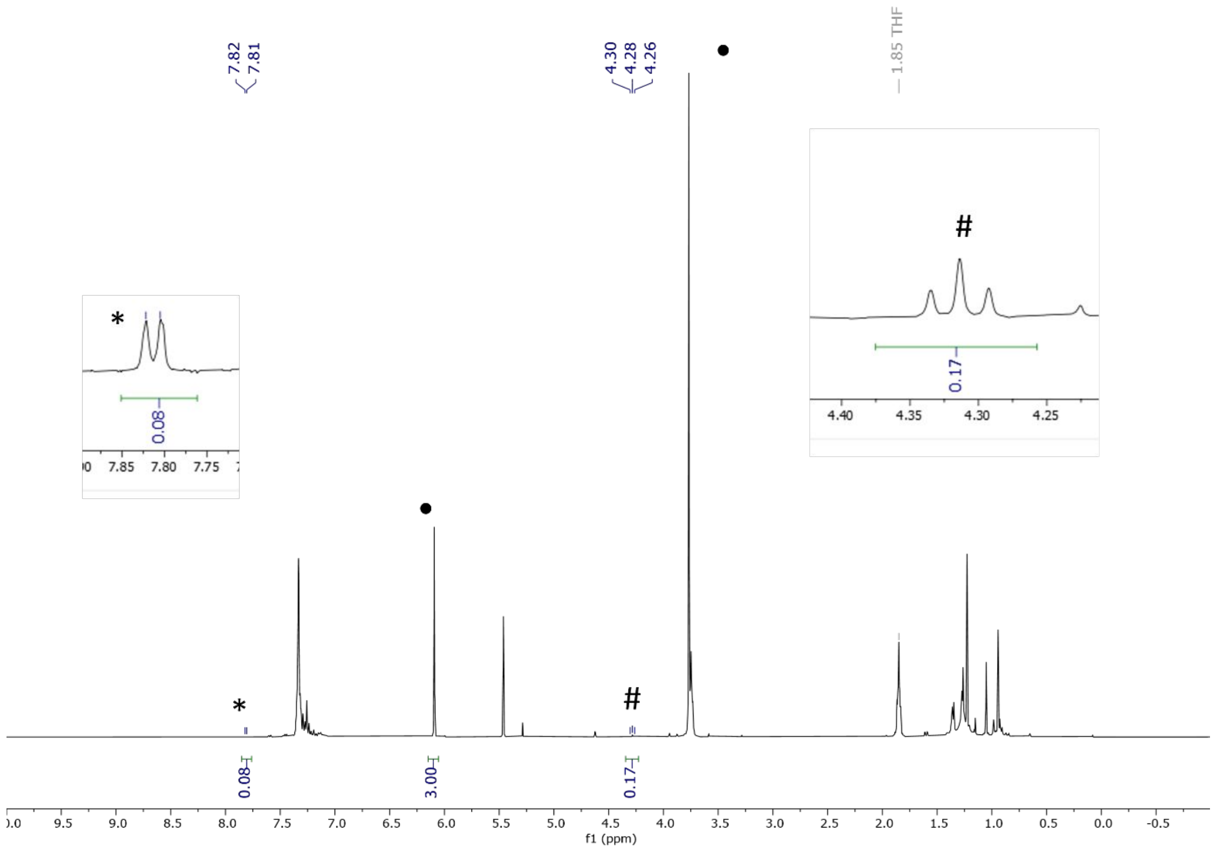

Figure S6. ^{1}H NMR spectrum of the crude reaction mixture from the reaction in the presence of 1,1-DPE. Peaks arising due to internal standard (1,3,5-trimethoxybenzene, "•", 3H), phenyl Bpin **2a** ("*", 2H) and the hydroboration product[25] ("#", 1H) are highlighted.

9 NMR spectra of isolated compounds

***o*-tolyl trifluoromethanesulfonate (4b)**

^{1}H NMR 400 MHz, $CDCl_3$

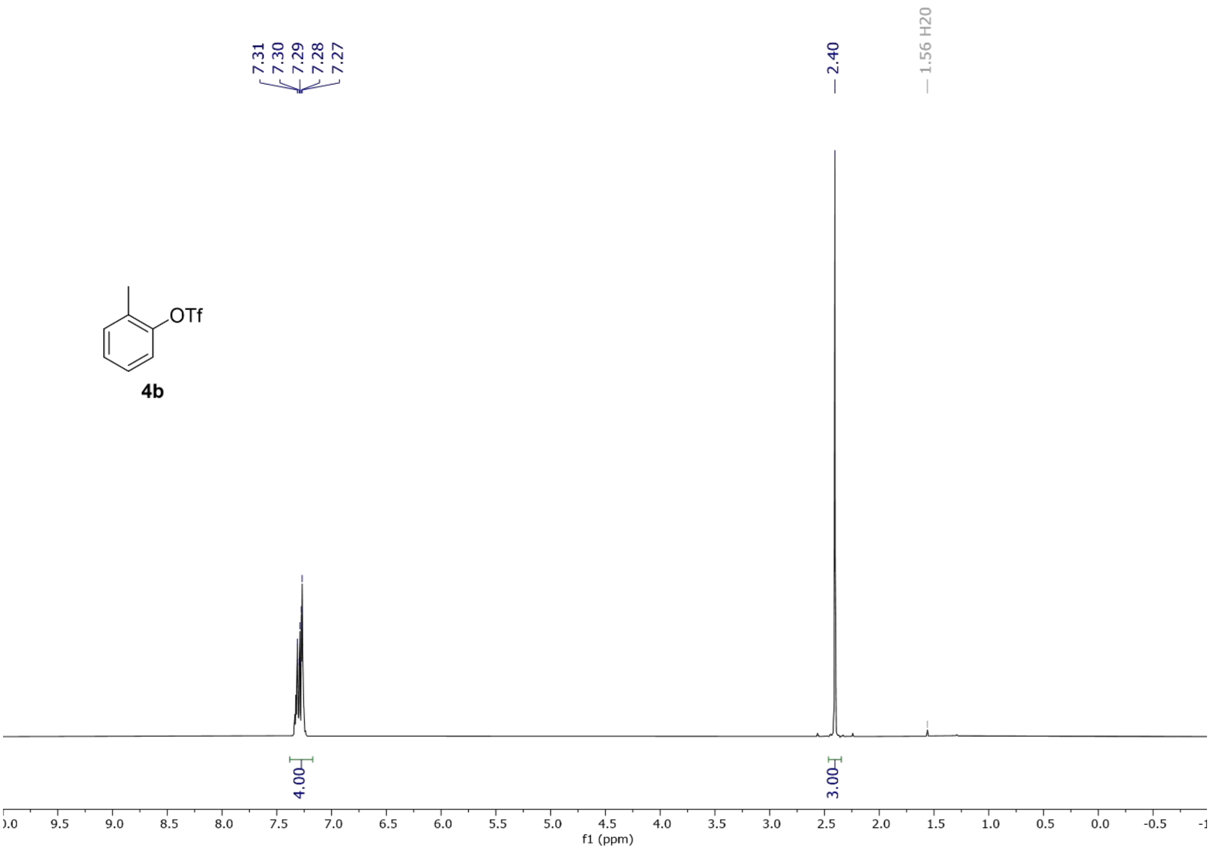

^{13}C NMR 101 MHz, $CDCl_3$

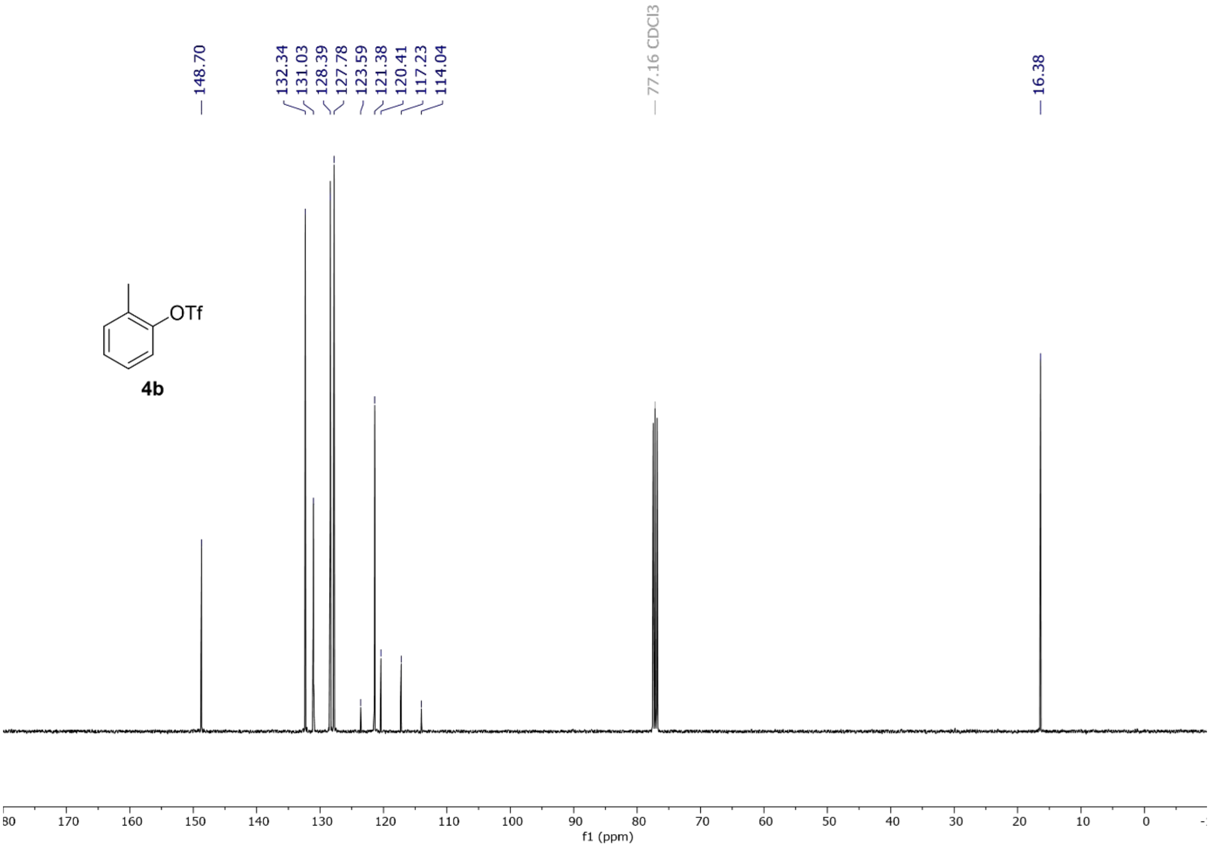

^{19}F NMR 377 MHz, $CDCl_3$

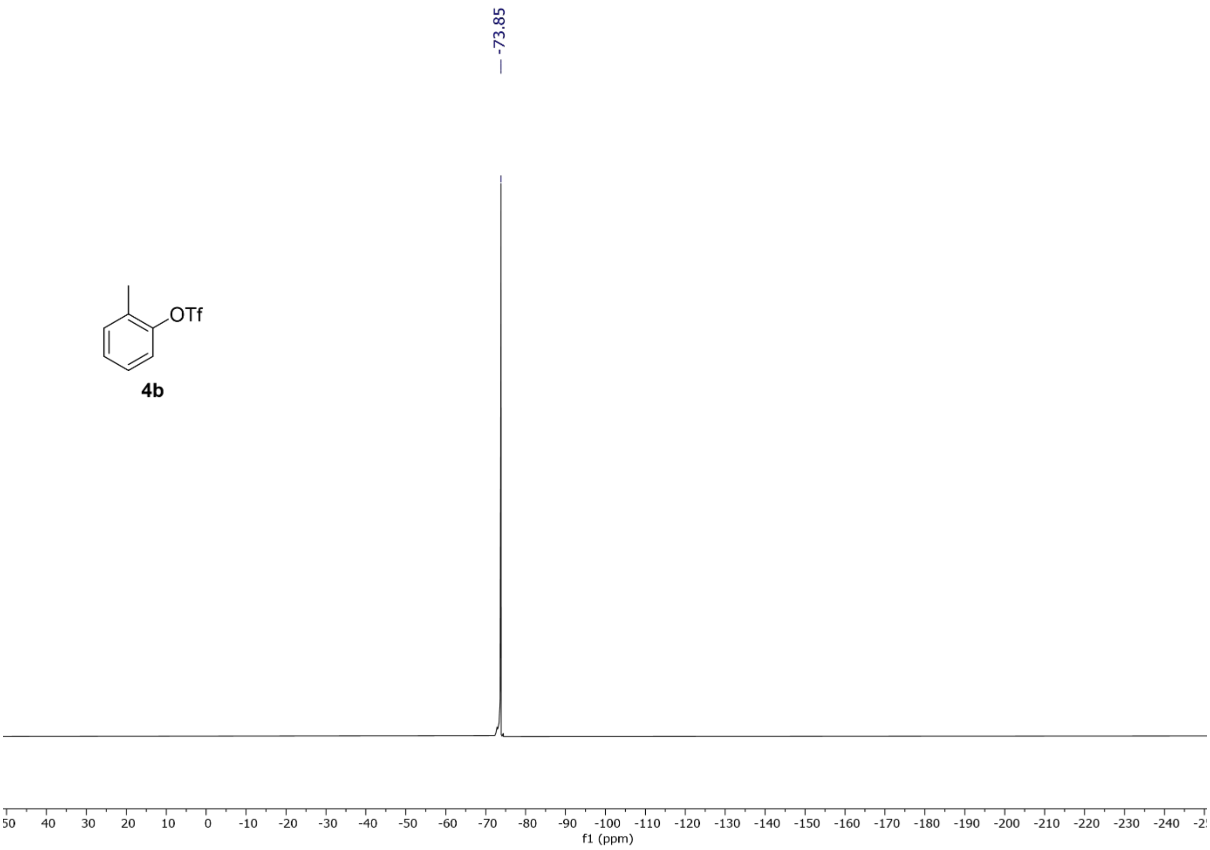

***m*-tolyl trifluoromethanesulfonate (4c)**

^{1}H NMR 400 MHz, $CDCl_3$

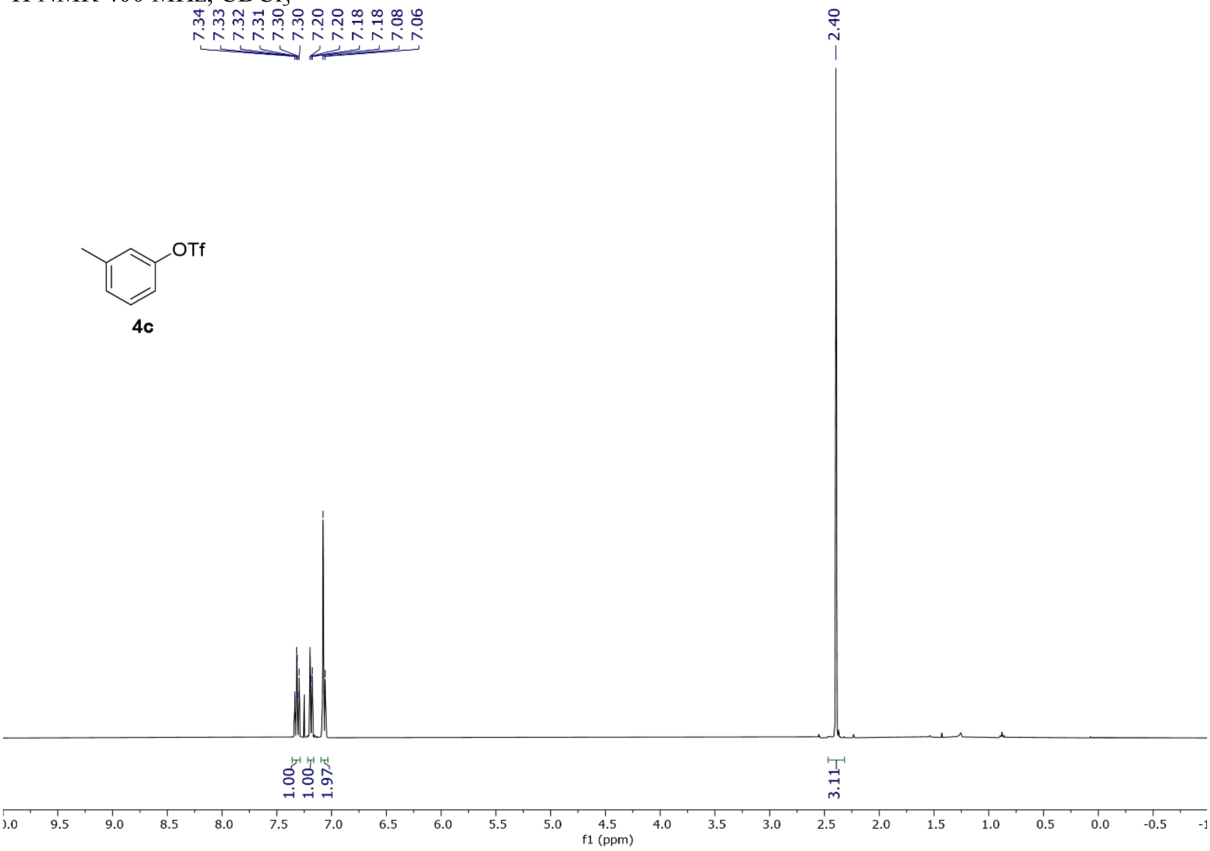

^{13}C NMR 101 MHz, $CDCl_3$

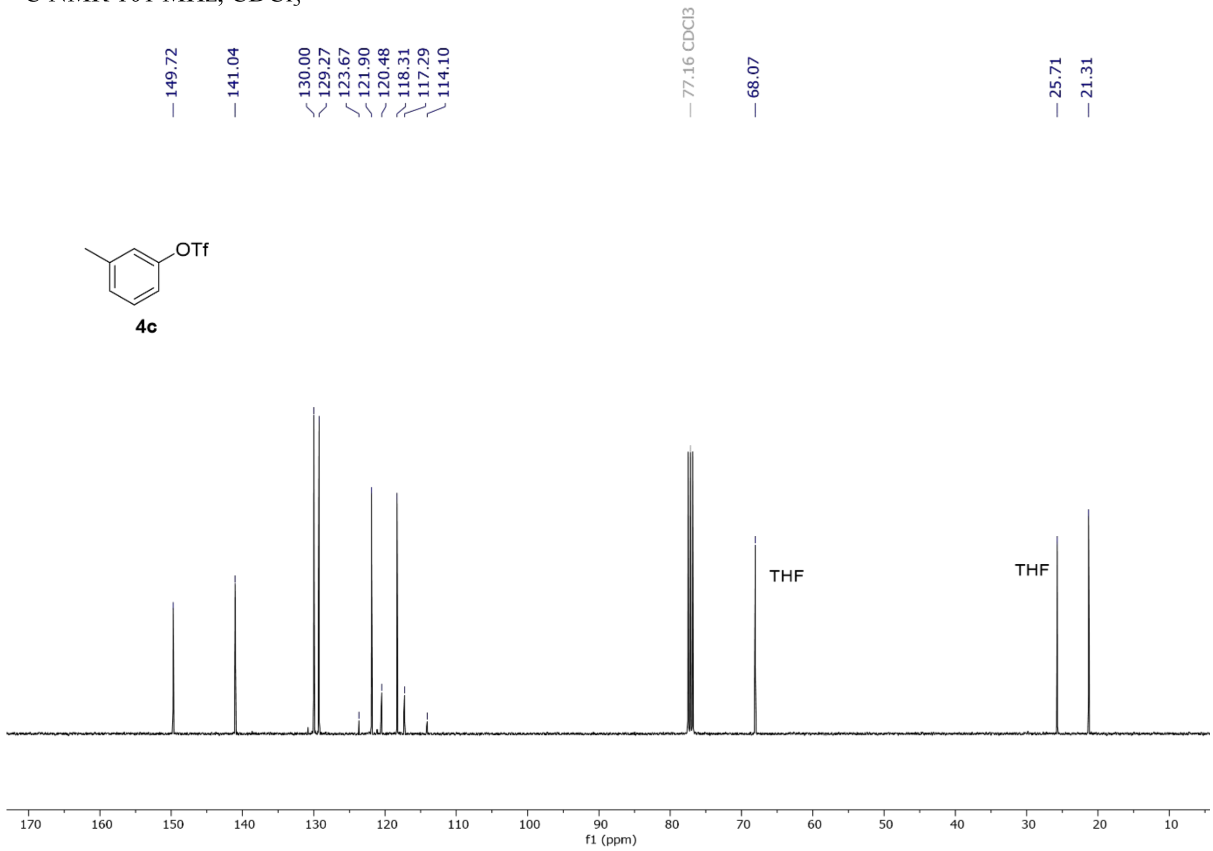

^{19}F NMR 377 MHz, $CDCl_3$

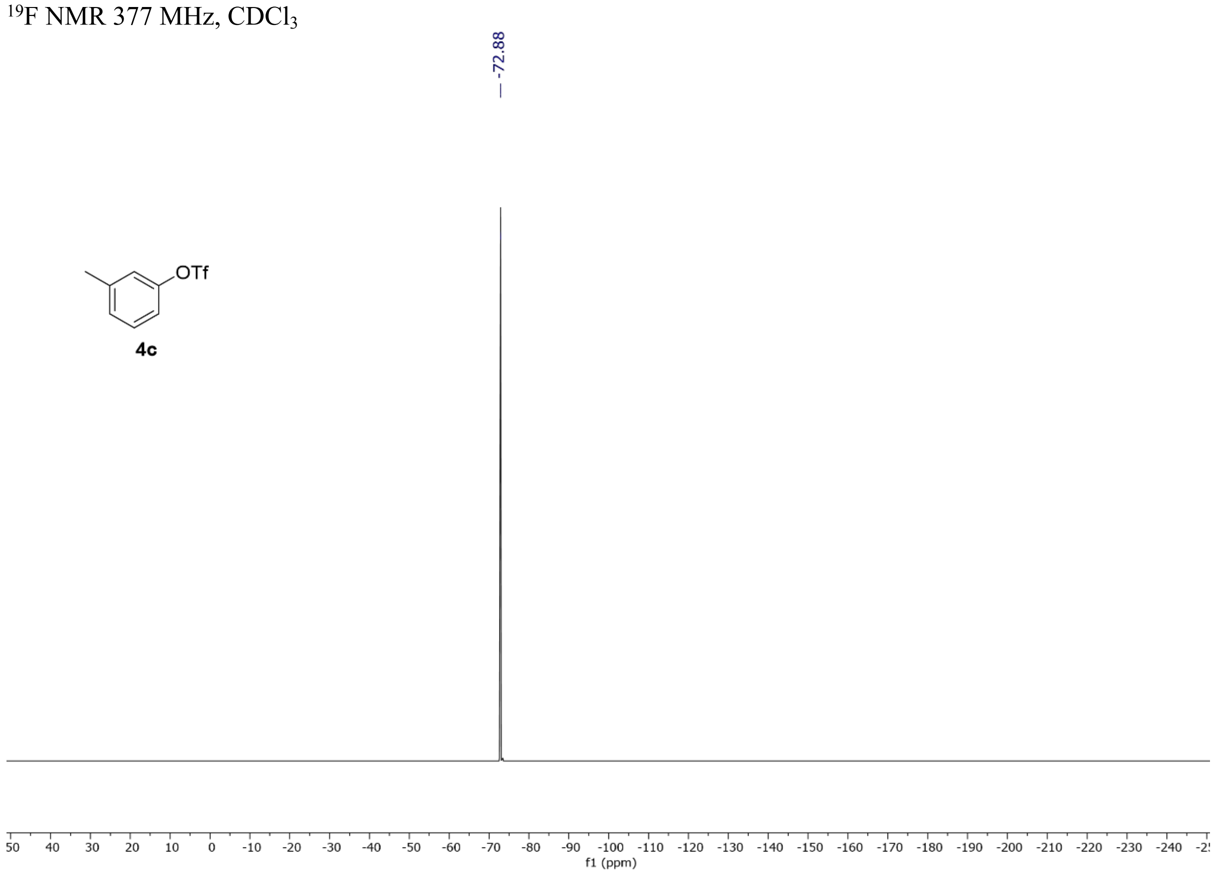

4-methoxyphenyl trifluoromethanesulfonate (4e)

^{1}H NMR 400 MHz, $CDCl_3$

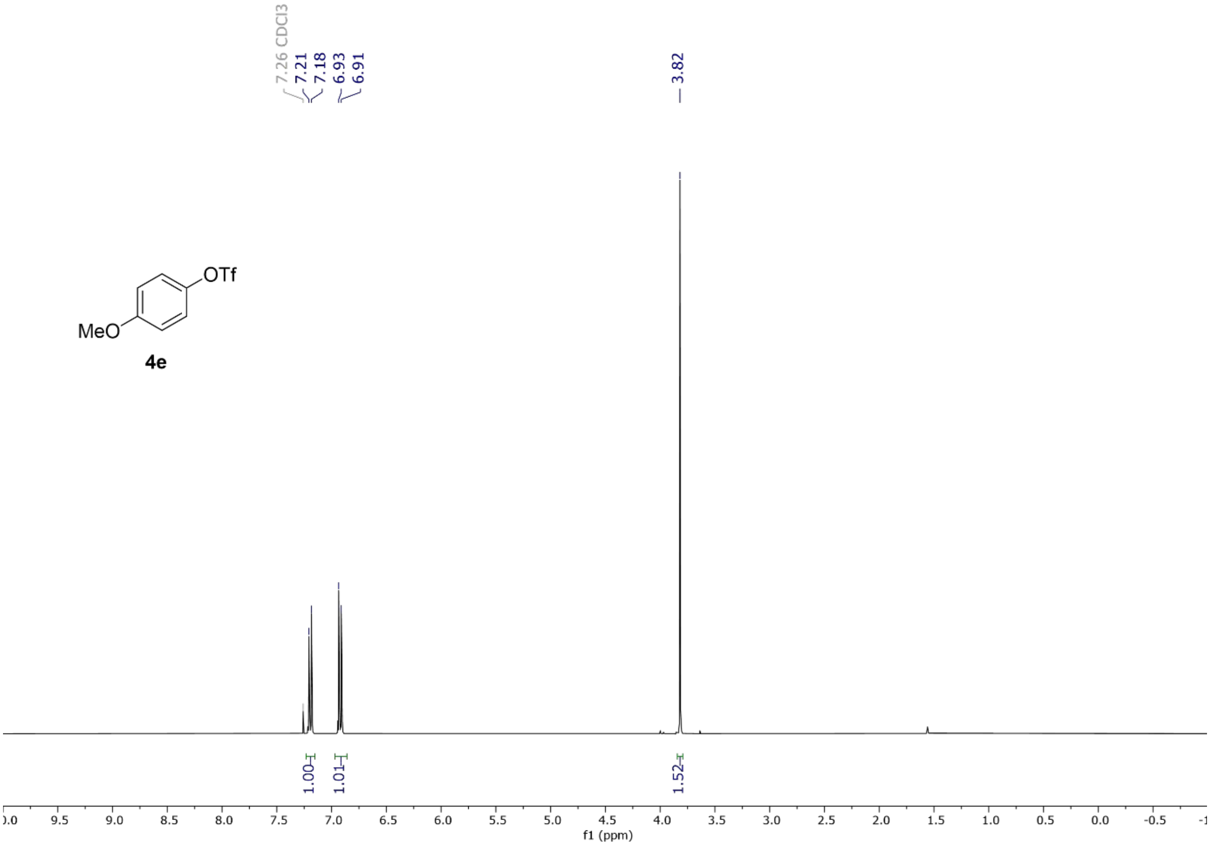

^{13}C NMR 101 MHz, $CDCl_3$

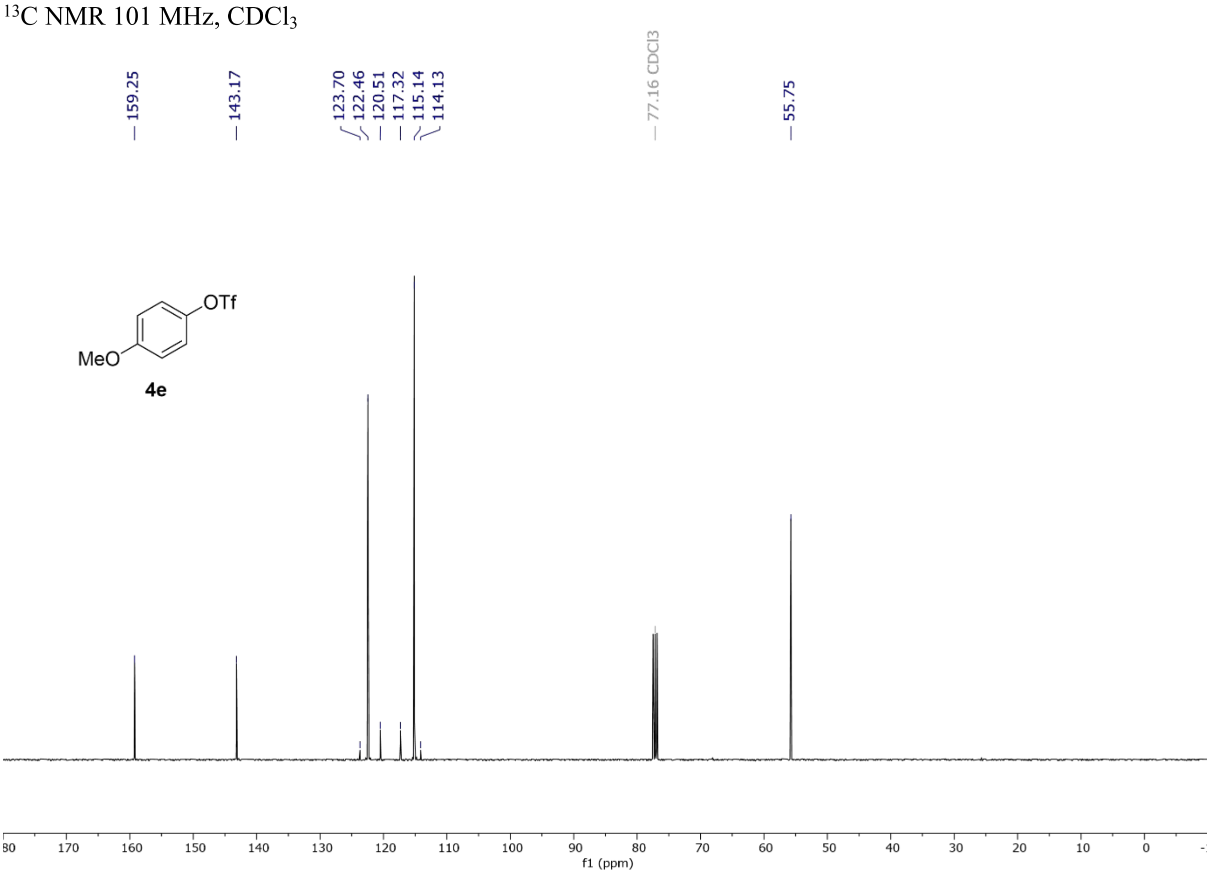

^{19}F NMR 377 MHz, $CDCl_3$

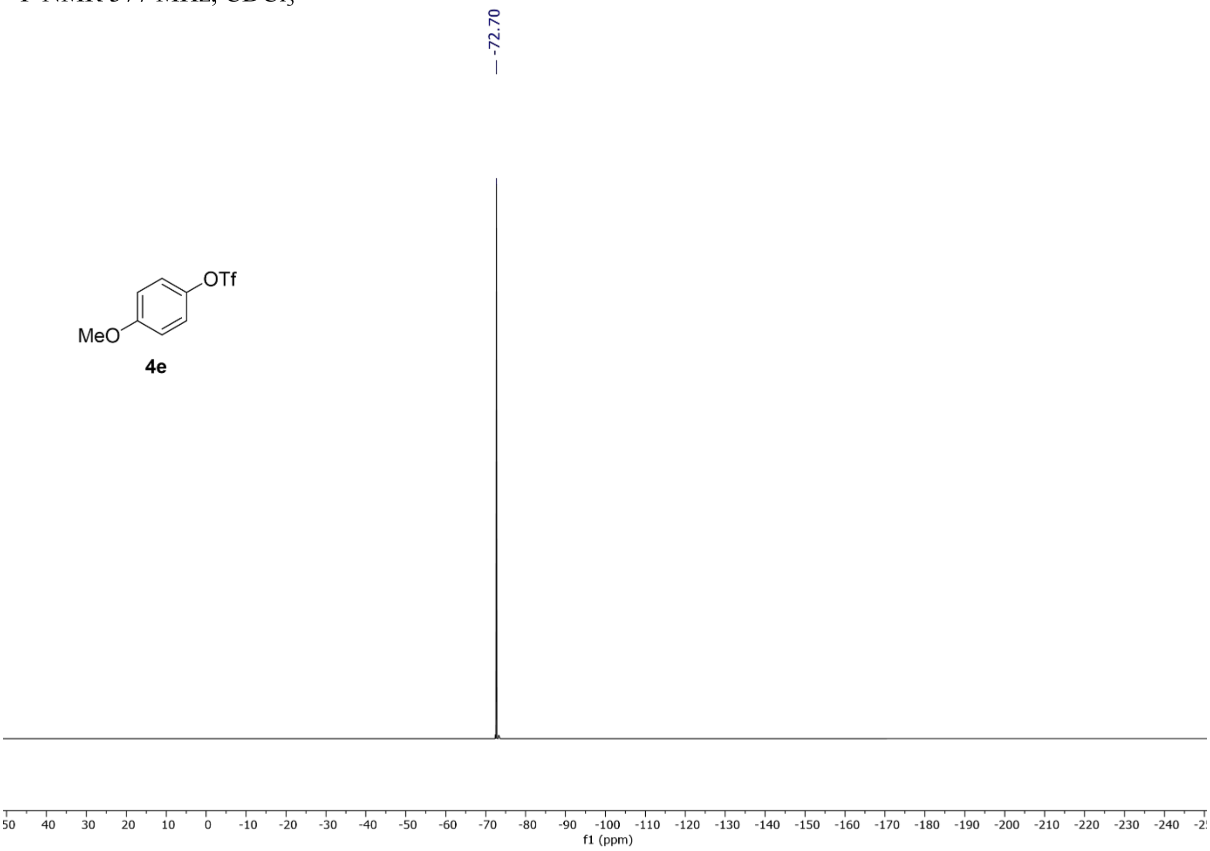

4-fluorophenyl trifluoromethanesulfonate (4f)

^{1}H NMR 400 MHz, $CDCl_3$

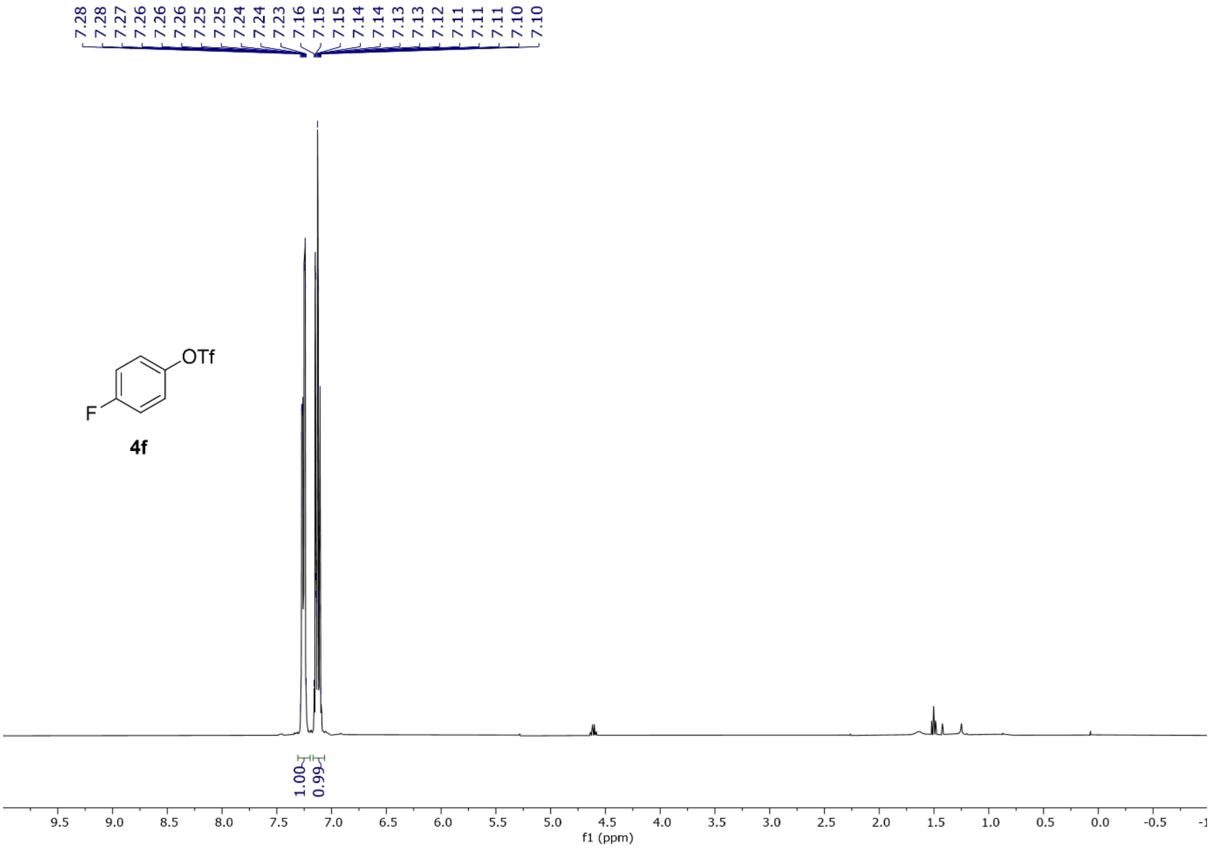

^{13}C NMR 101 MHz, $CDCl_3$

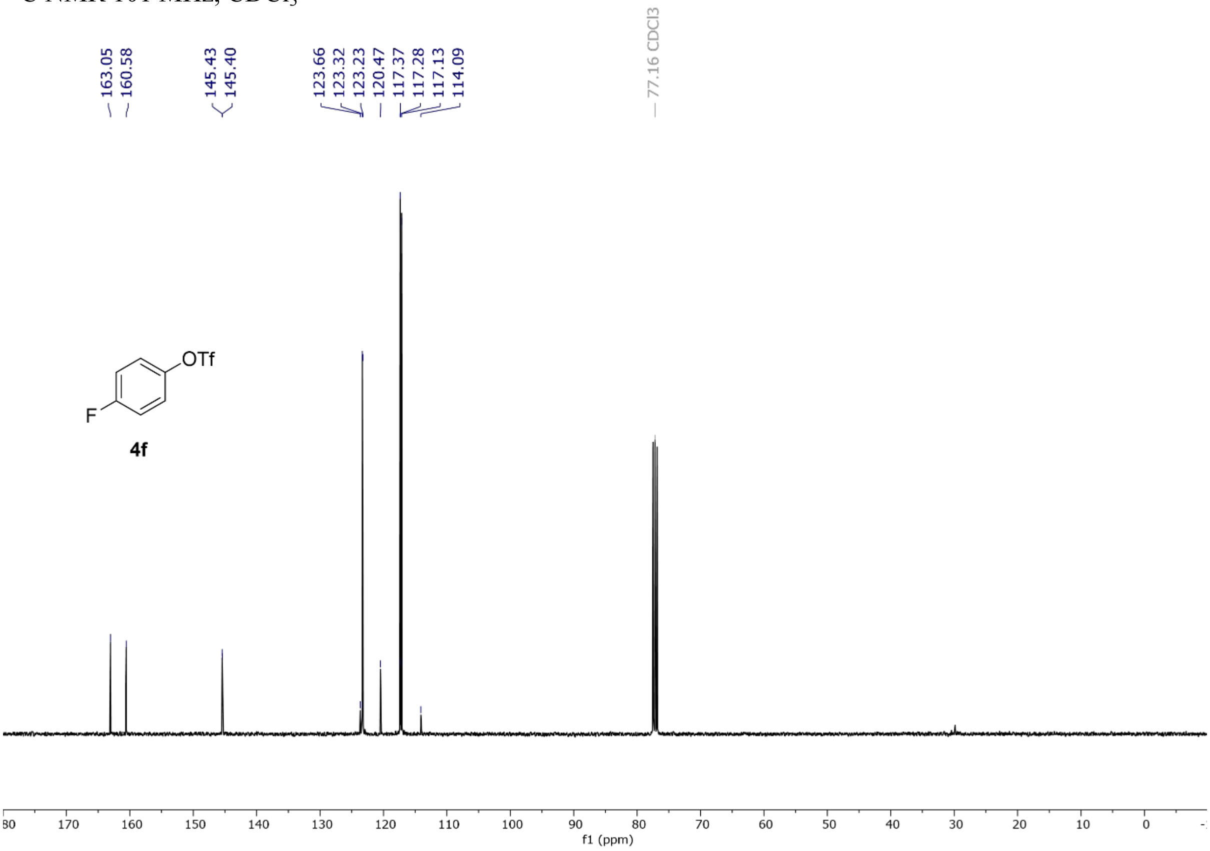

^{19}F NMR 377 MHz, $CDCl_3$

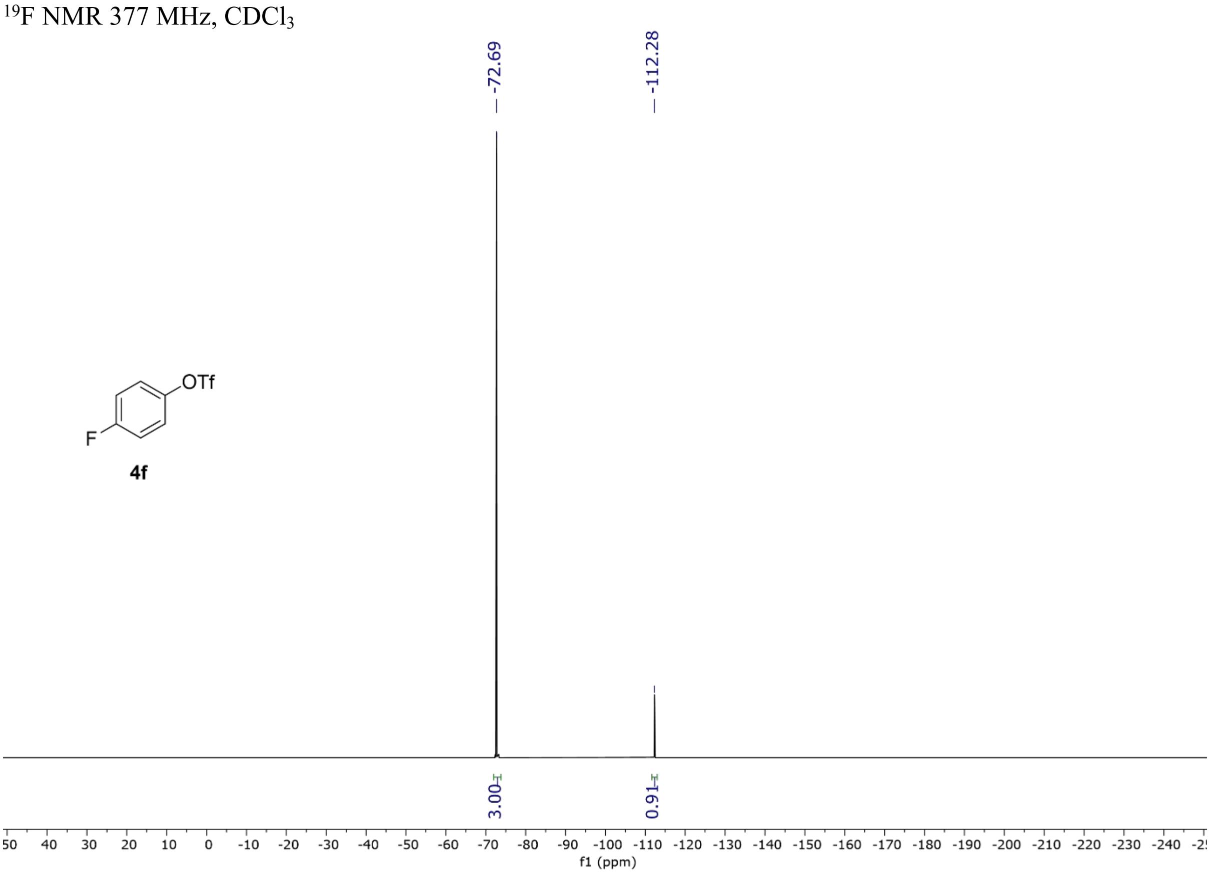

4-(trifluoromethyl)phenyl trifluoromethanesulfonate (4g)

^{1}H NMR 400 MHz, $CDCl_3$

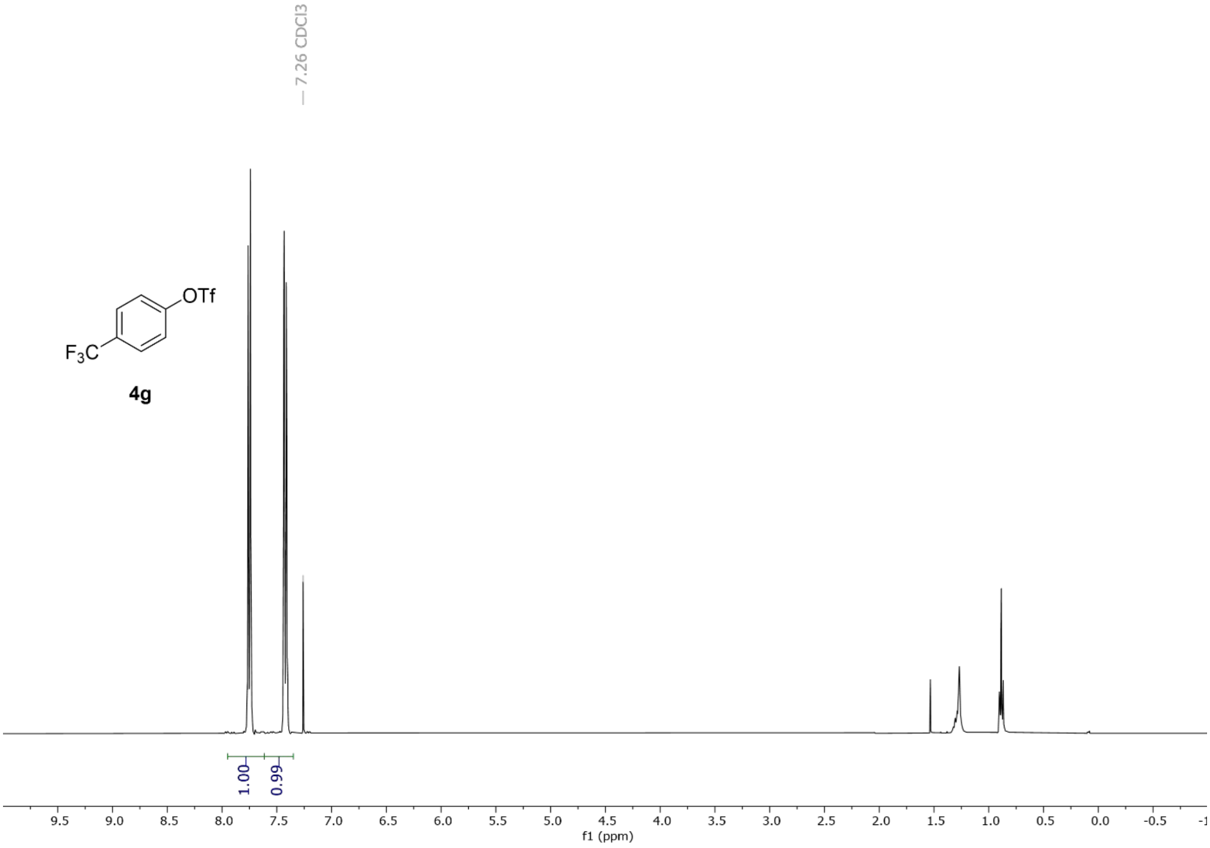

^{13}C NMR 101 MHz, $CDCl_3$

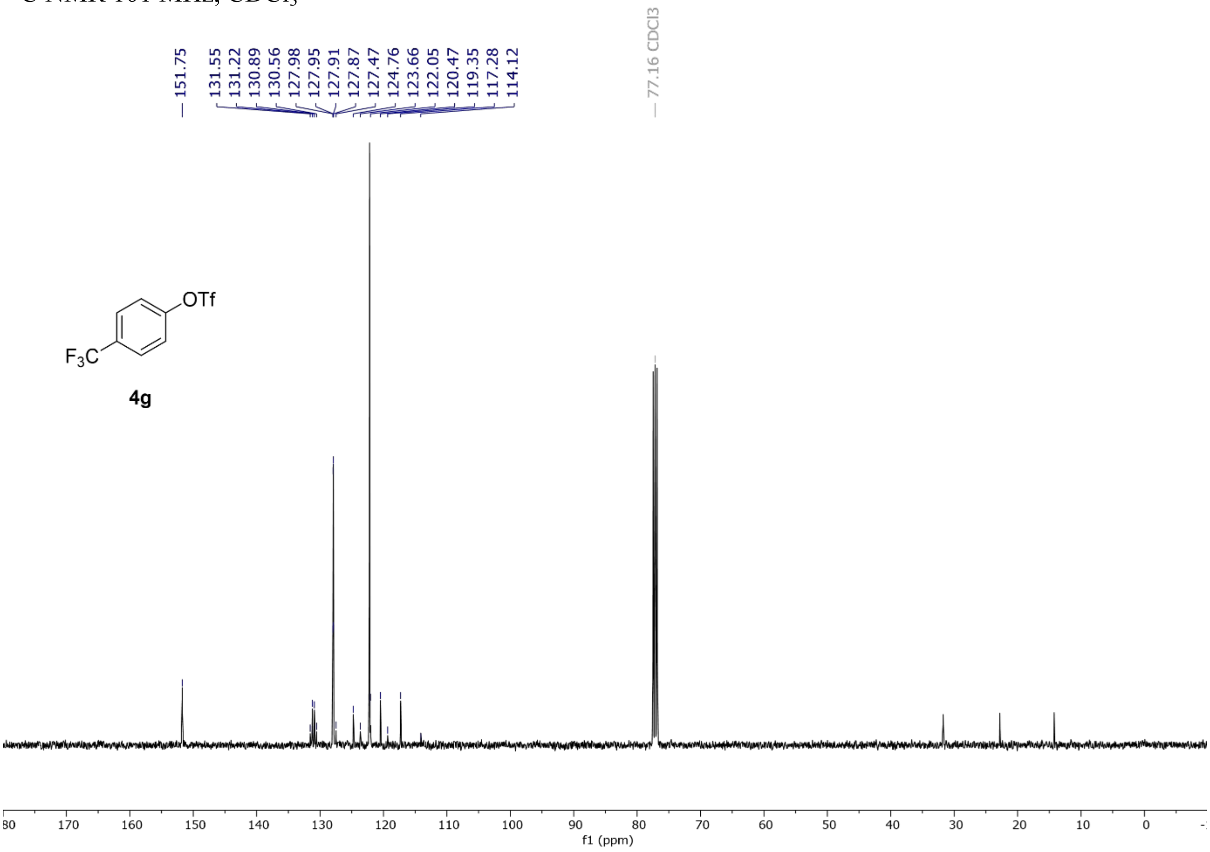

^{19}F NMR 377 MHz, $CDCl_3$

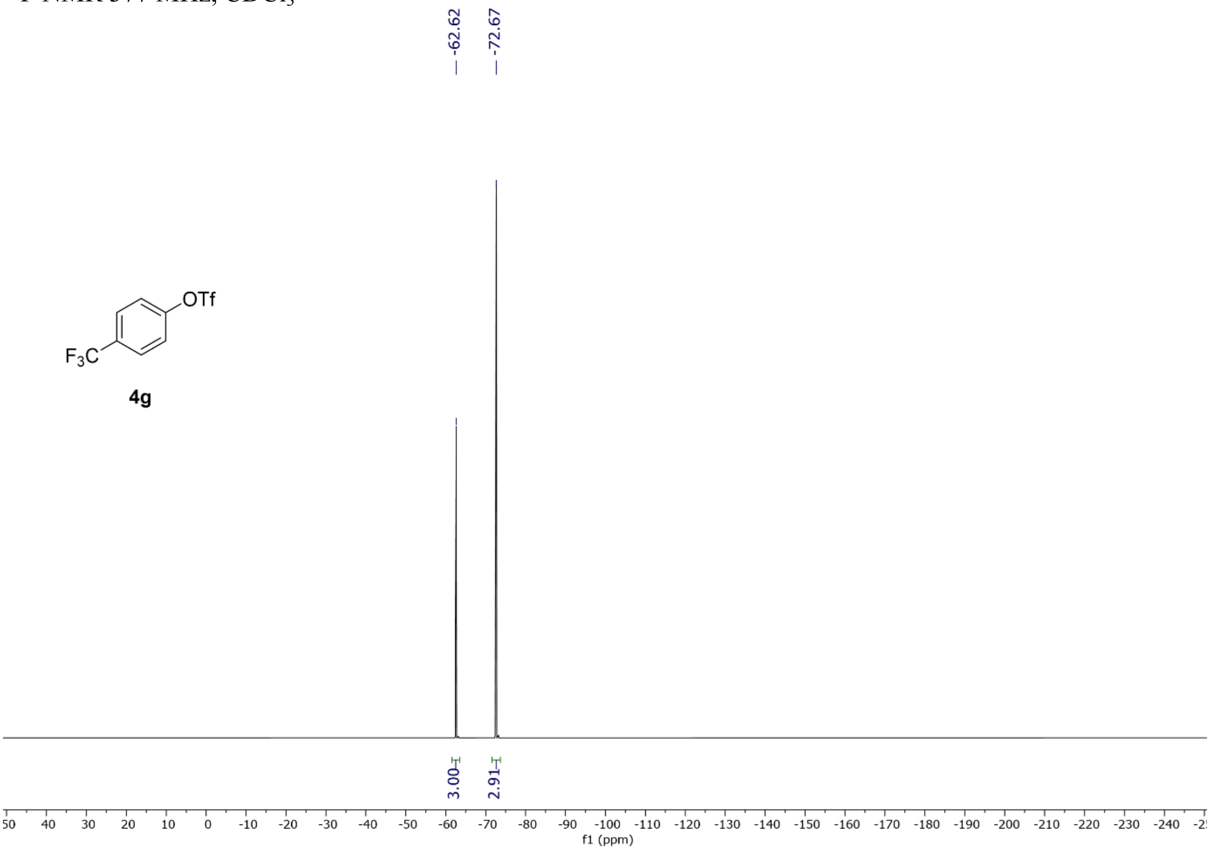

naphthalen-1-yl trifluoromethanesulfonate (4h)

^{1}H NMR 400 MHz, $CDCl_3$

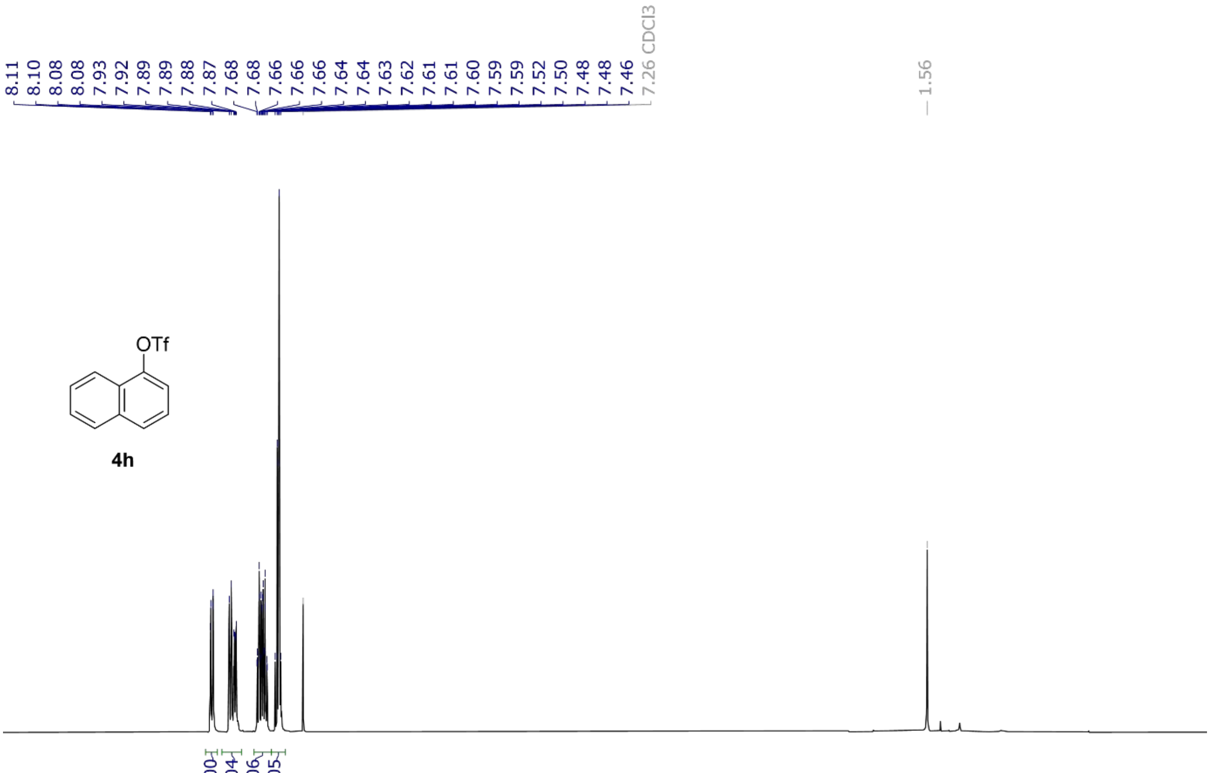

^{13}C NMR 101 MHz, $CDCl_3$

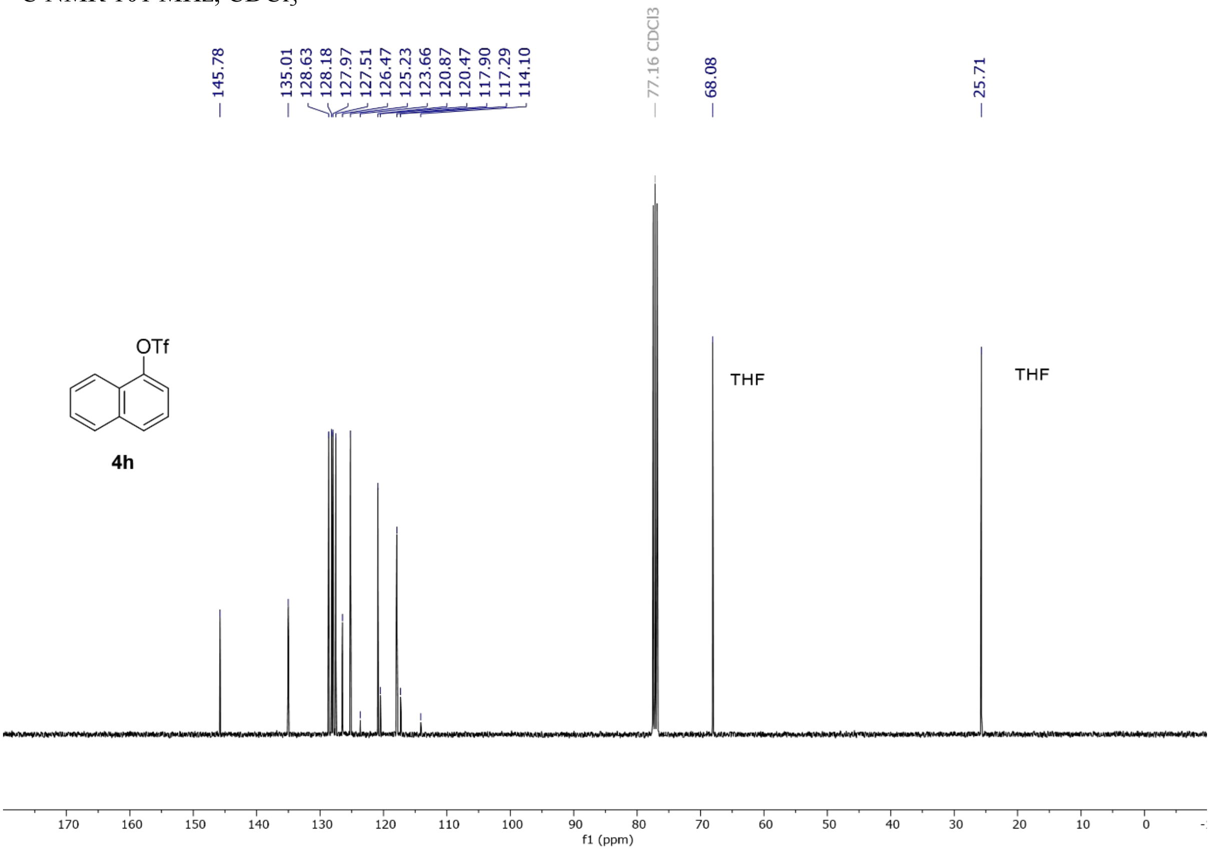

^{19}F NMR 377 MHz, $CDCl_3$

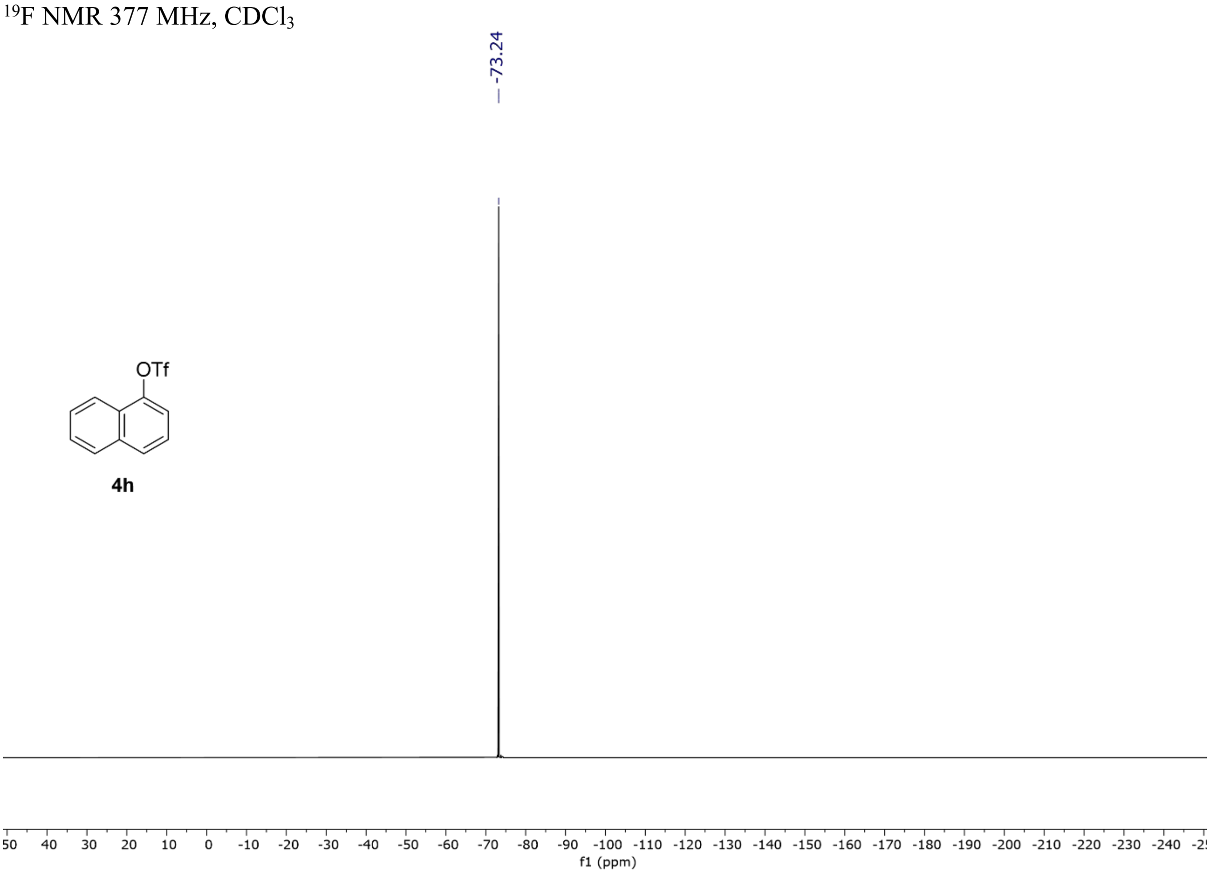

[1,1'-biphenyl]-4-yl trifluoromethanesulfonate (4u)

^{1}H NMR 400 MHz, $CDCl_3$

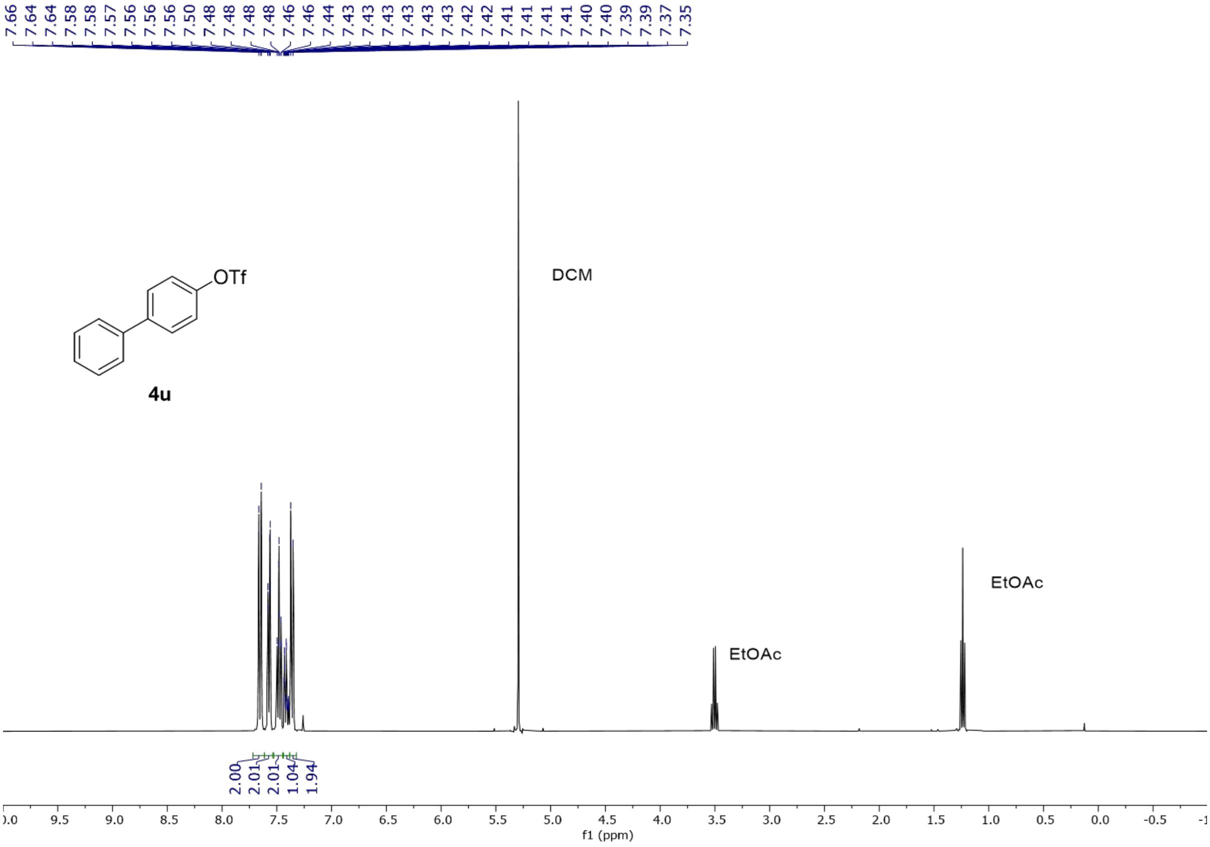

^{13}C NMR 101 MHz, $CDCl_3$

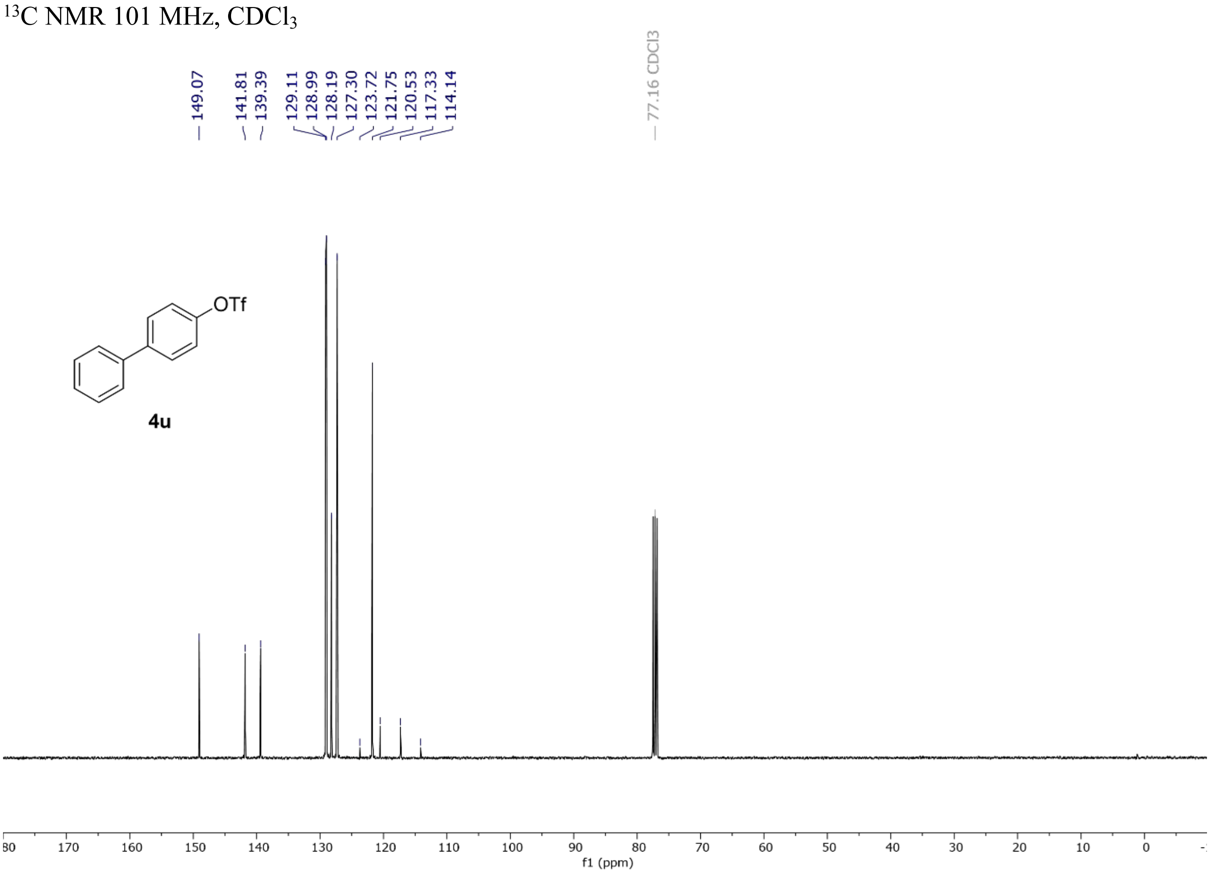

^{19}F NMR 377 MHz, $CDCl_3$

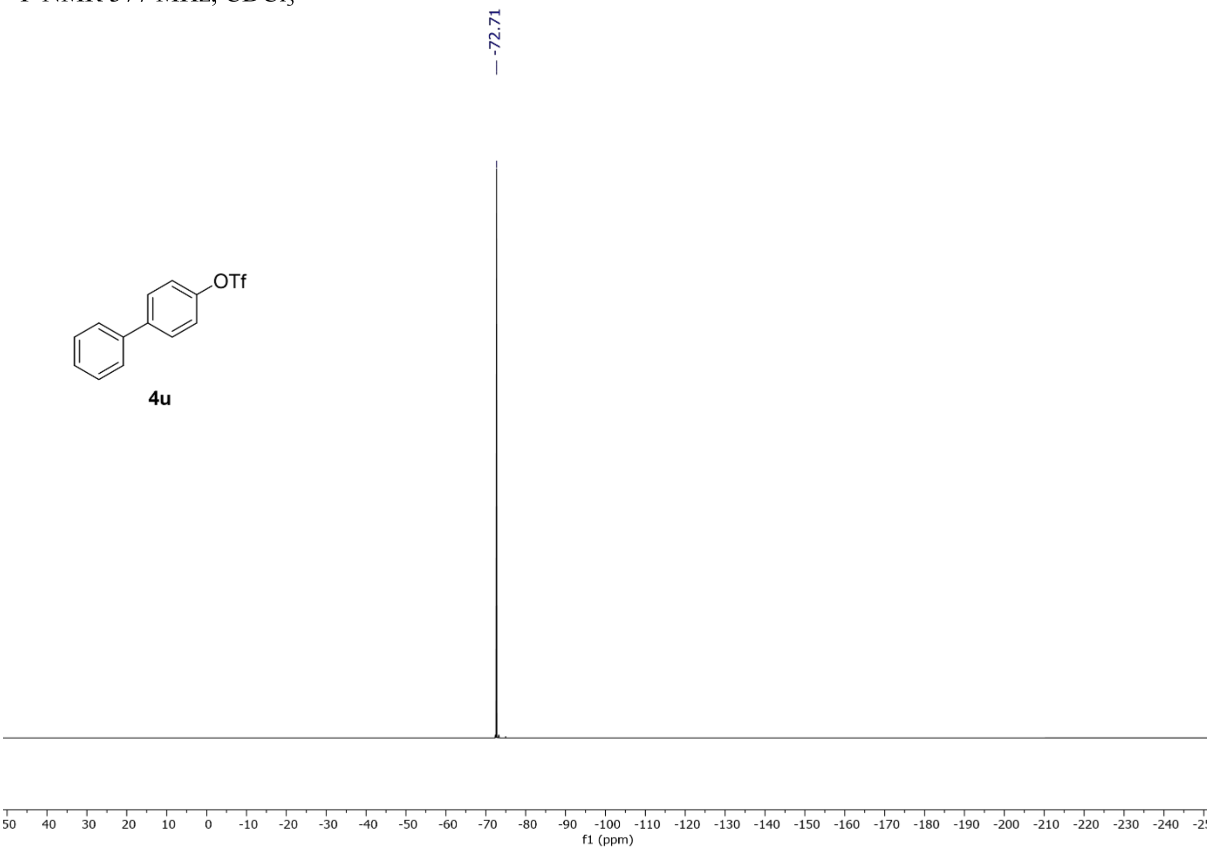

3,5-difluorophenyl trifluoromethanesulfonate (4v)

^{1}H NMR 400 MHz, $CDCl_3$

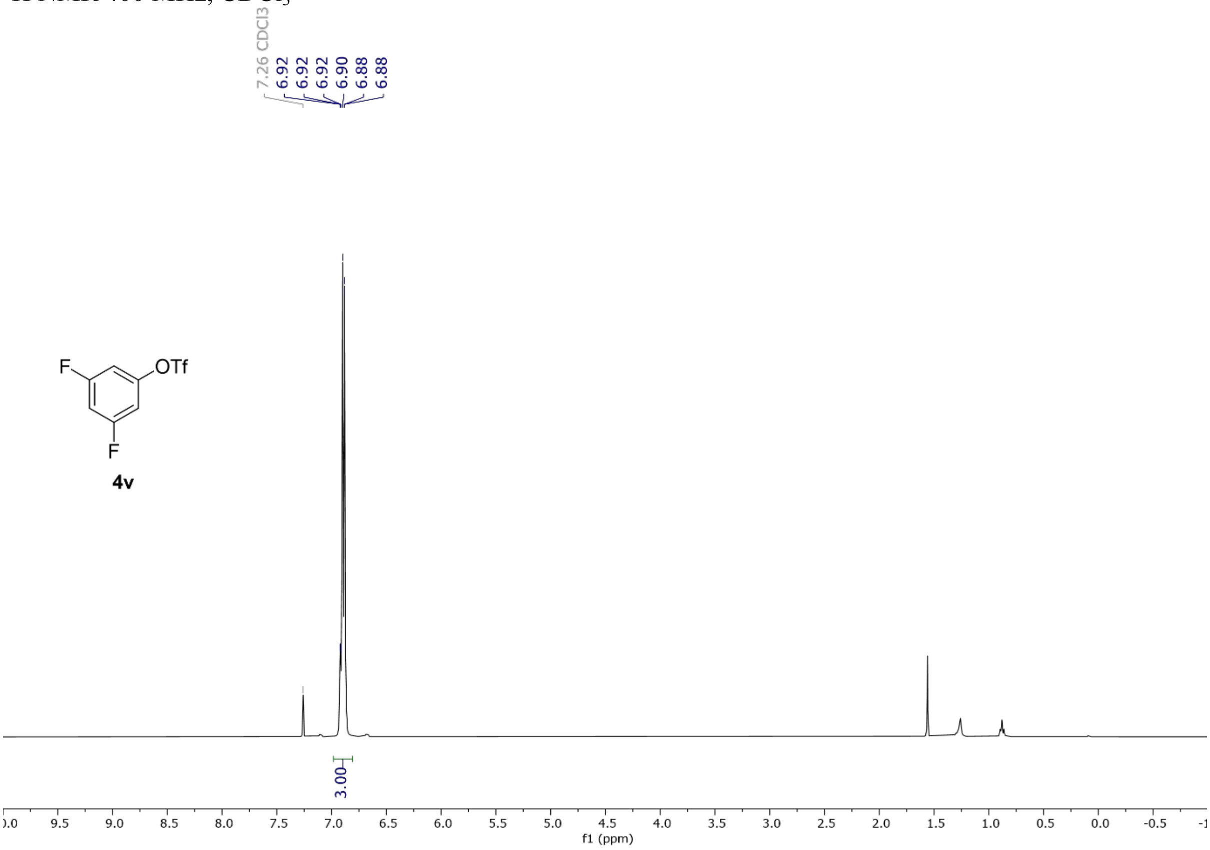

^{13}C NMR 101 MHz, $CDCl_3$

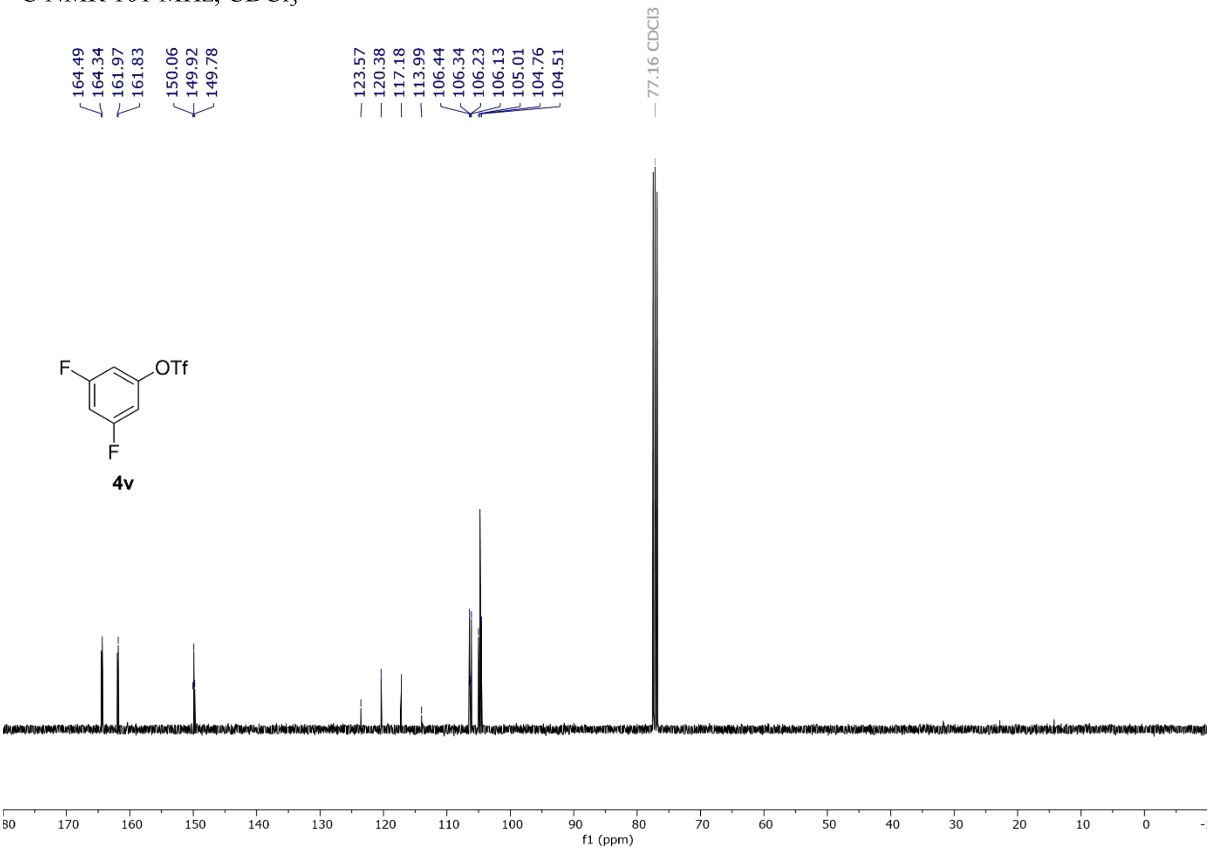

^{19}F NMR 377 MHz, $CDCl_3$

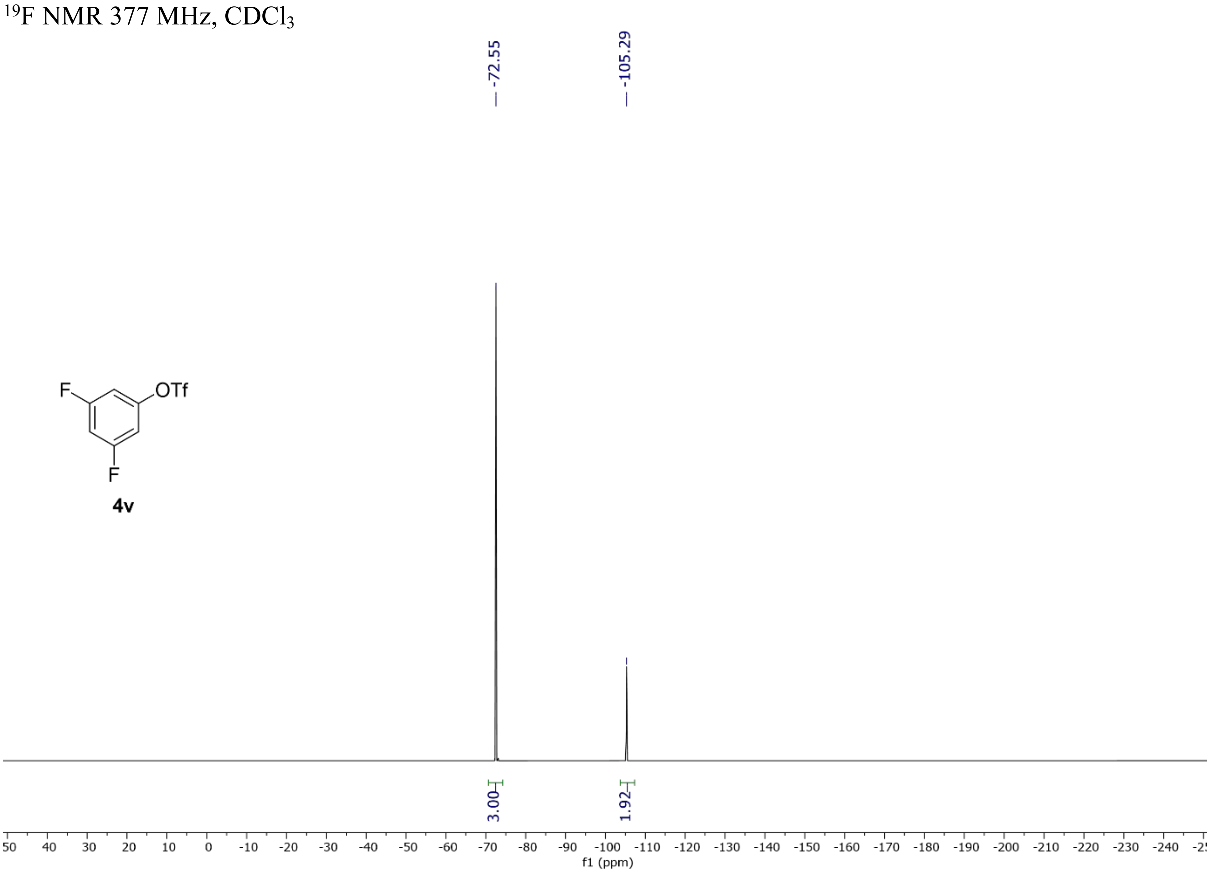

3,5-di-tert-butylphenyl trifluoromethanesulfonate (4w)

^{1}H NMR 400 MHz, $CDCl_3$

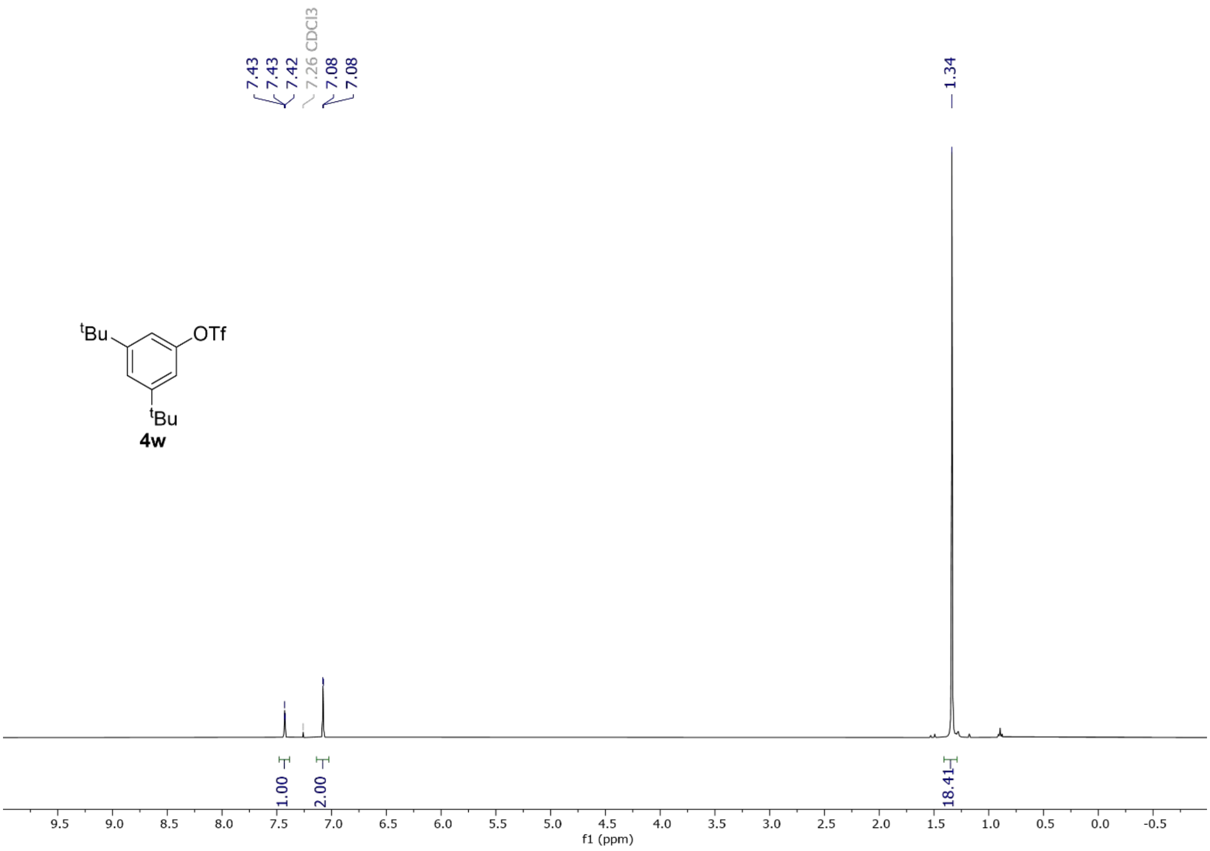

^{13}C NMR 101 MHz, $CDCl_3$

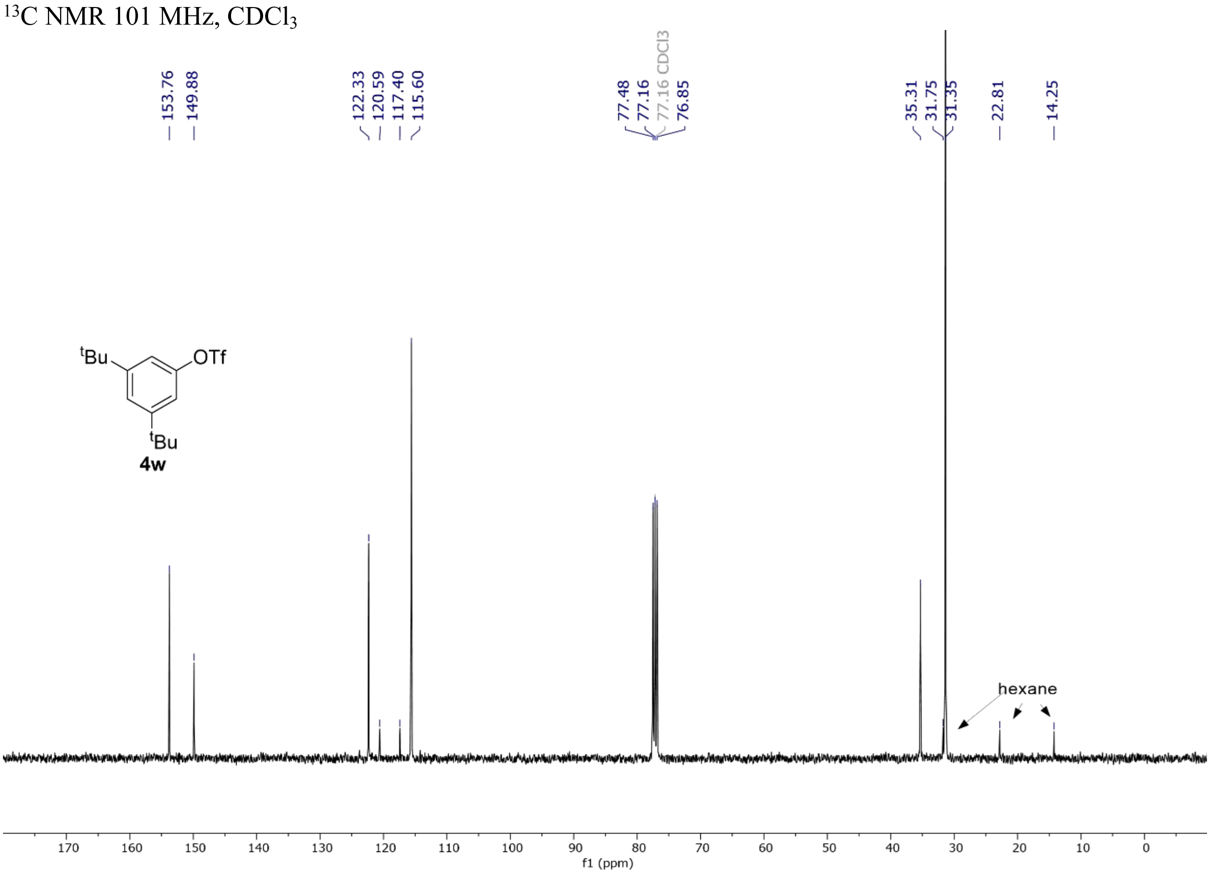

^{19}F NMR 377 MHz, $CDCl_3$

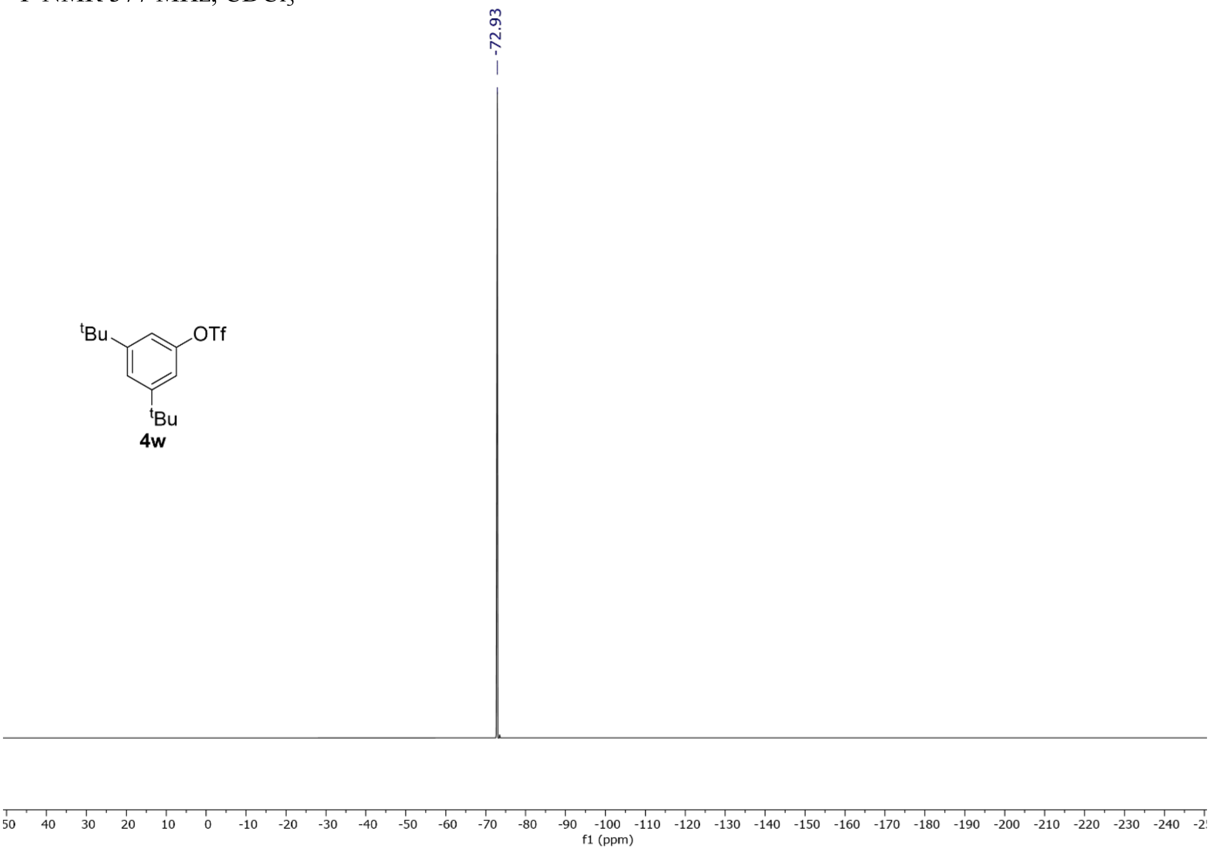

4,4,5,5-tetramethyl-2-phenyl-1,3,2-dioxaborolane (3a)

^{1}H NMR 400 MHz, $CDCl_3$

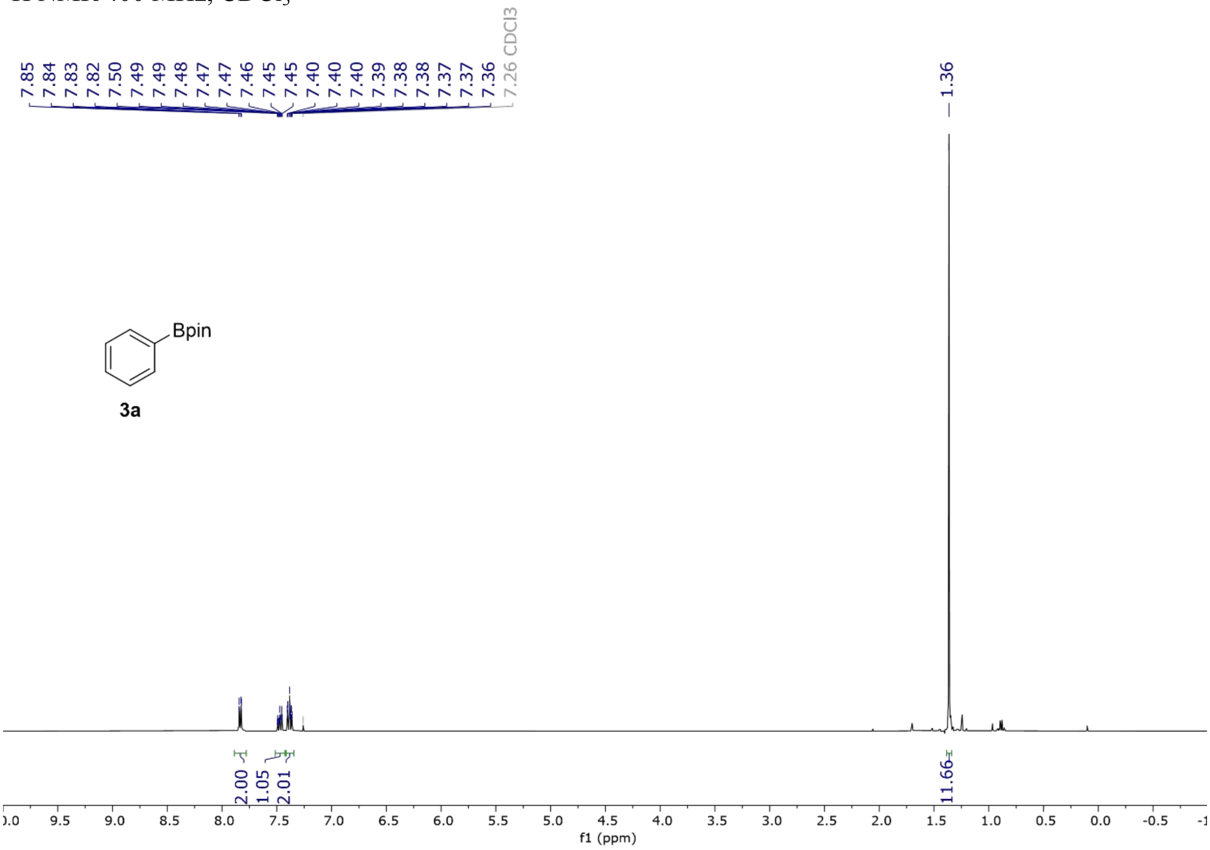

^{13}C NMR 101 MHz, $CDCl_3$

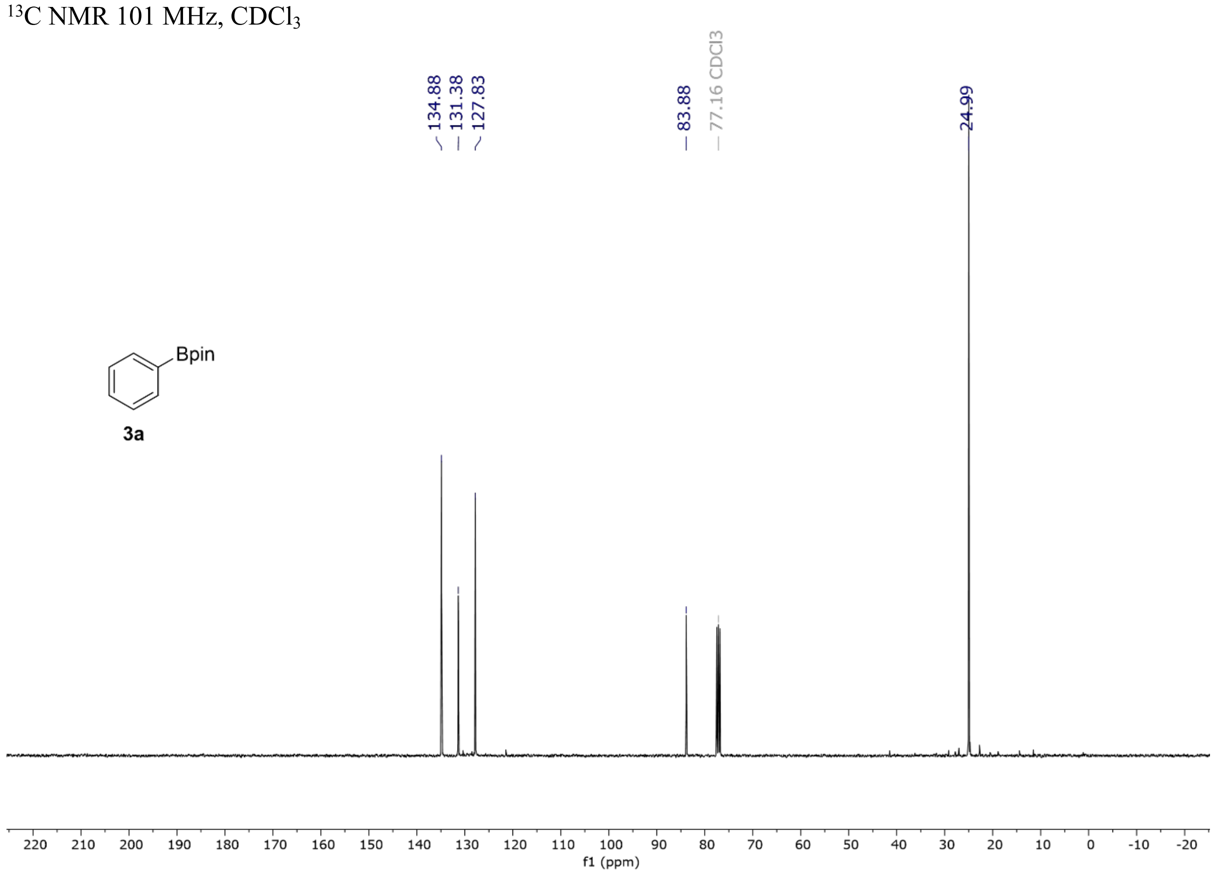

^{11}B NMR 128 MHz, $CDCl_3$

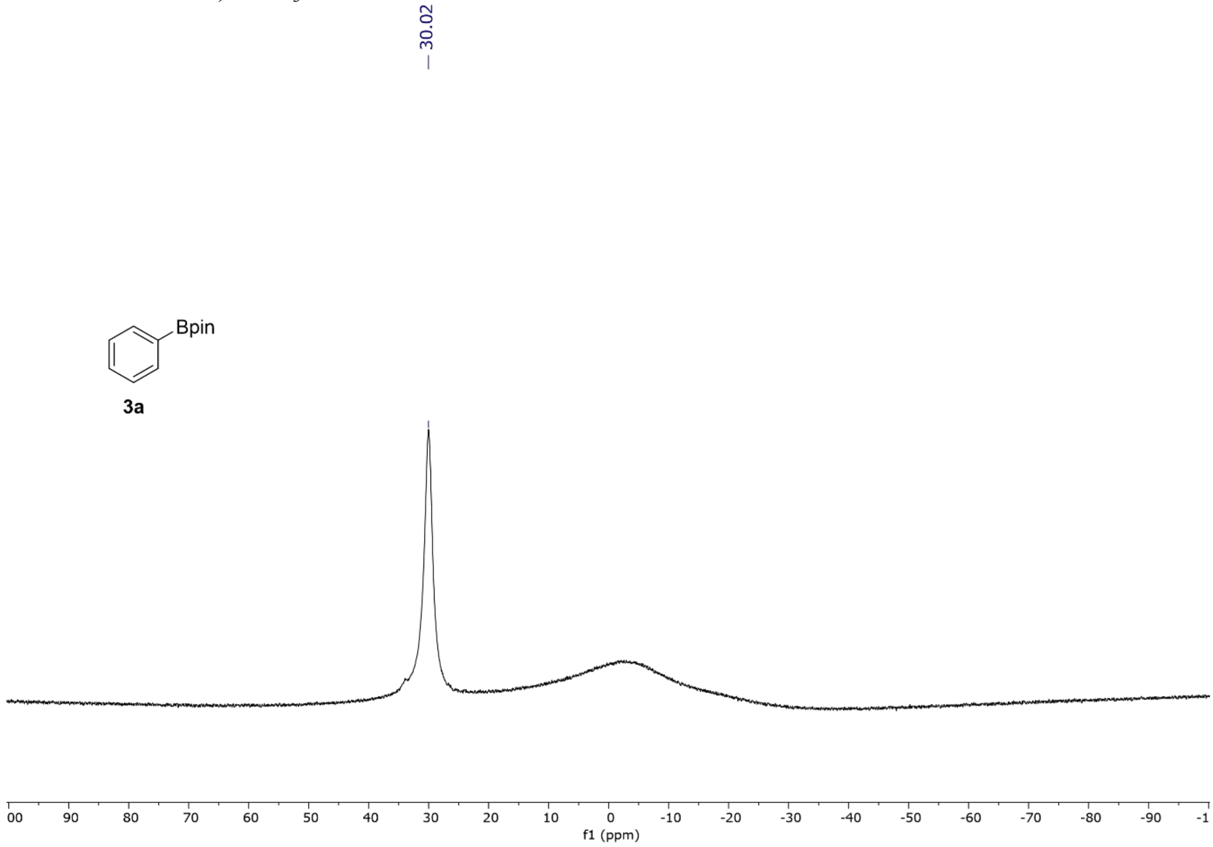

4,4,5,5-tetramethyl-2-(*o*-tolyl)-1,3,2-dioxaborolane (3b)

^{1}H NMR 400 MHz, $CDCl_3$

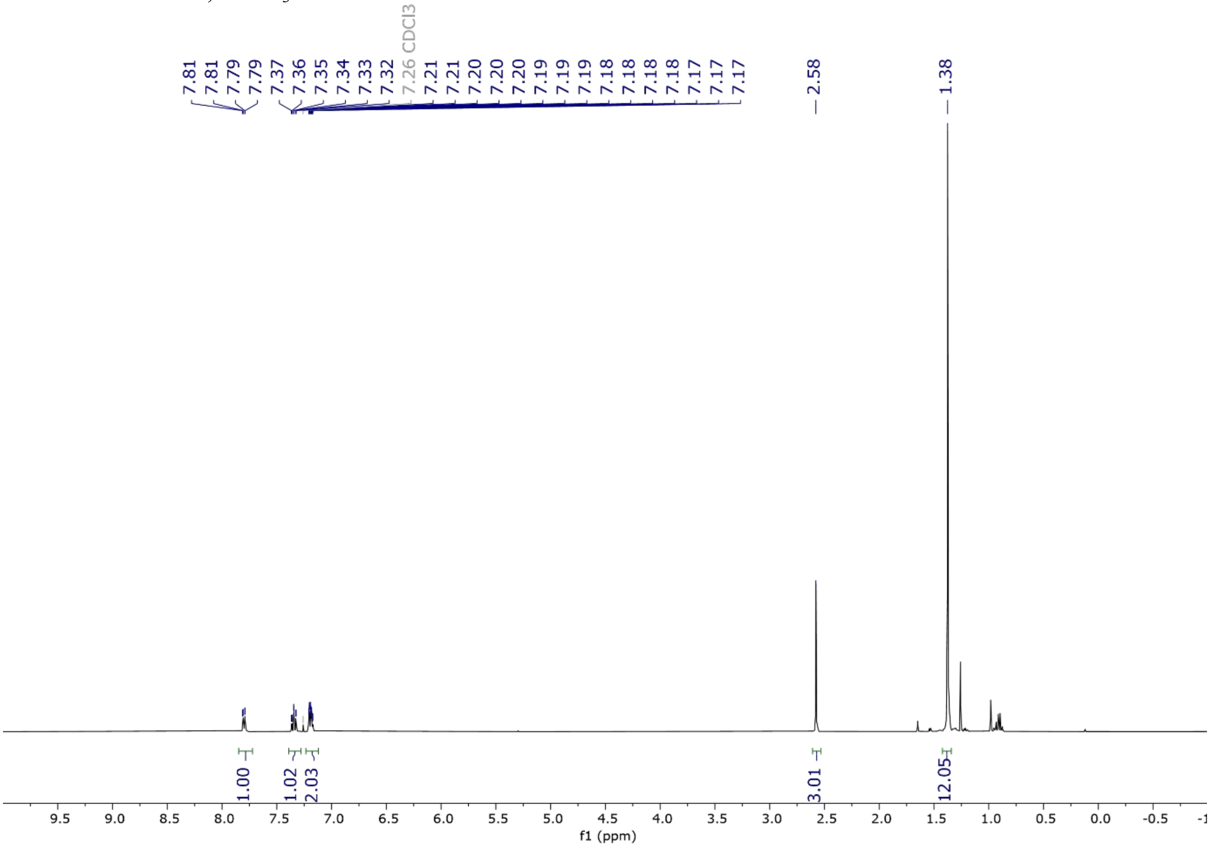

^{13}C NMR 101 MHz, $CDCl_3$

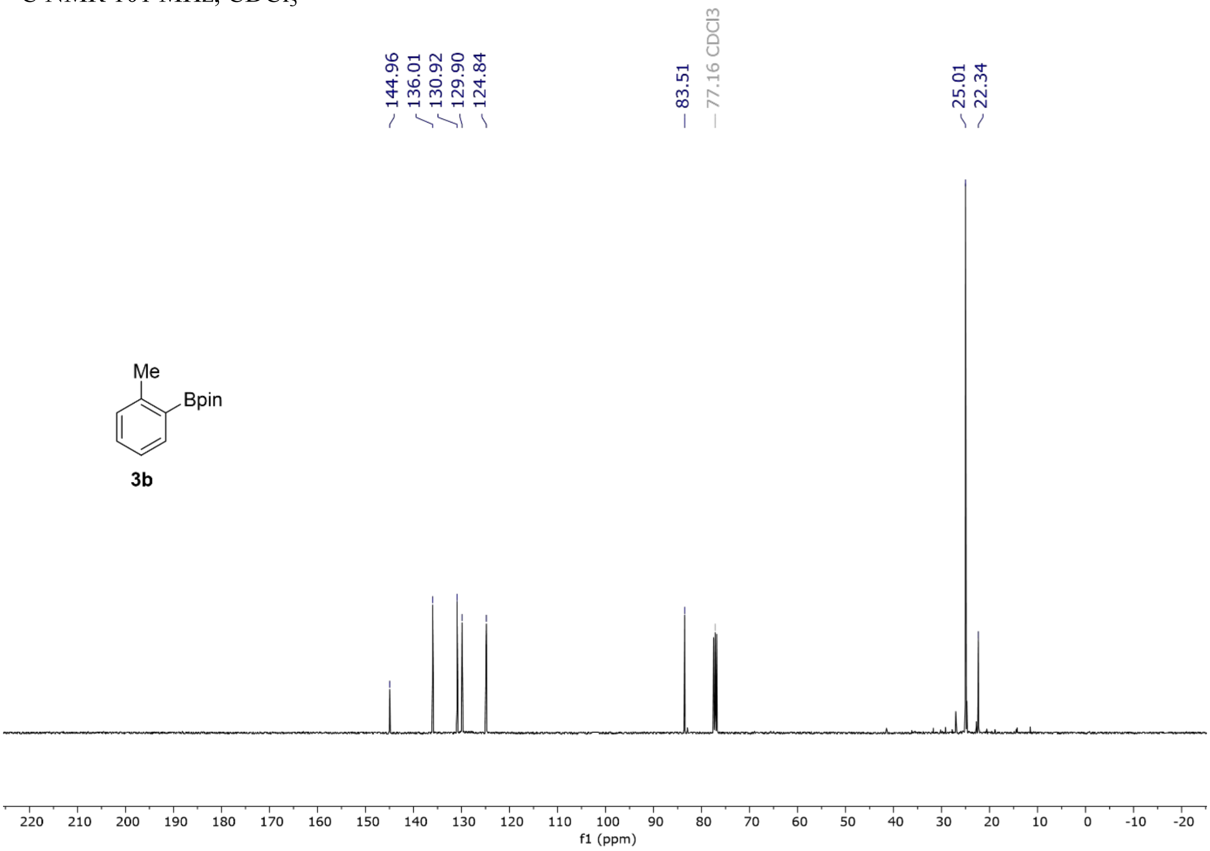

3b

^{11}B NMR 128 MHz, $CDCl_3$

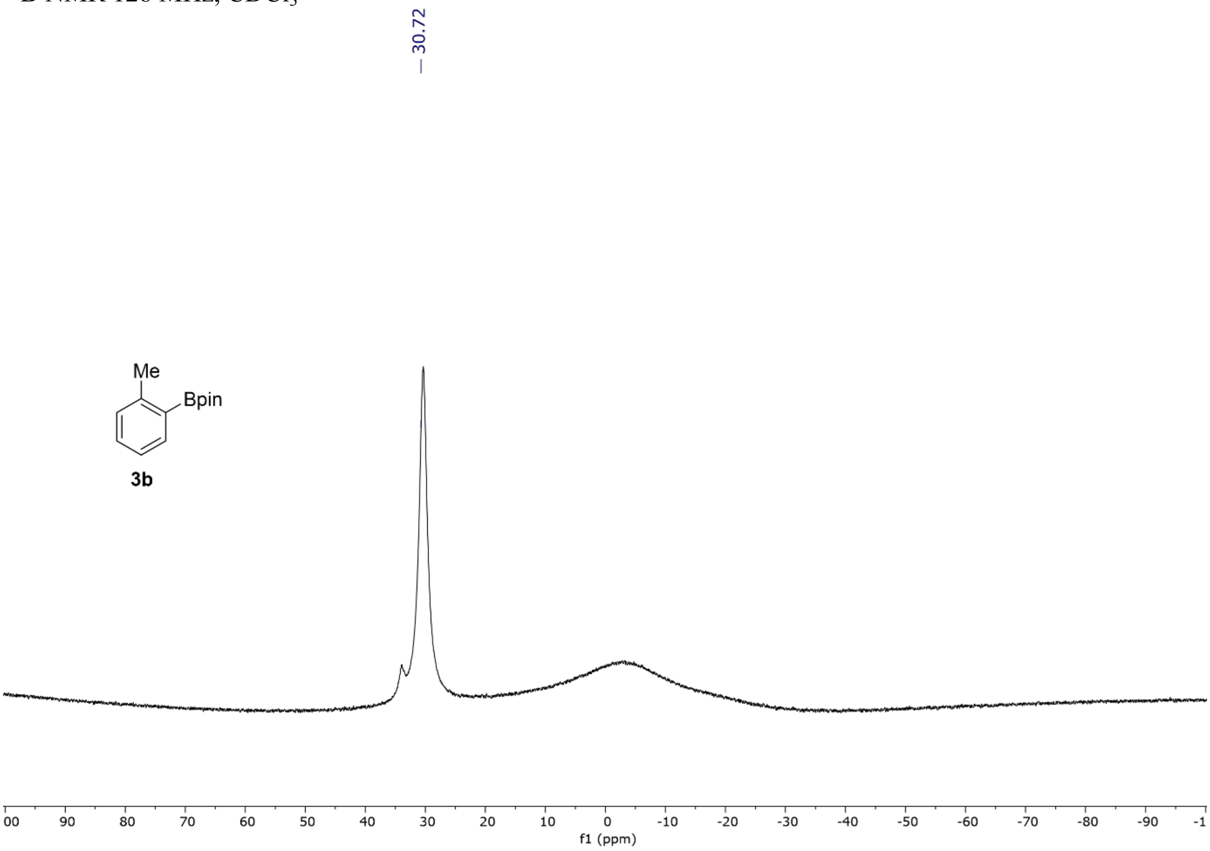

4,4,5,5-tetramethyl-2-(*m*-tolyl)-1,3,2-dioxaborolane (3c)

^{1}H NMR 400 MHz, $CDCl_3$

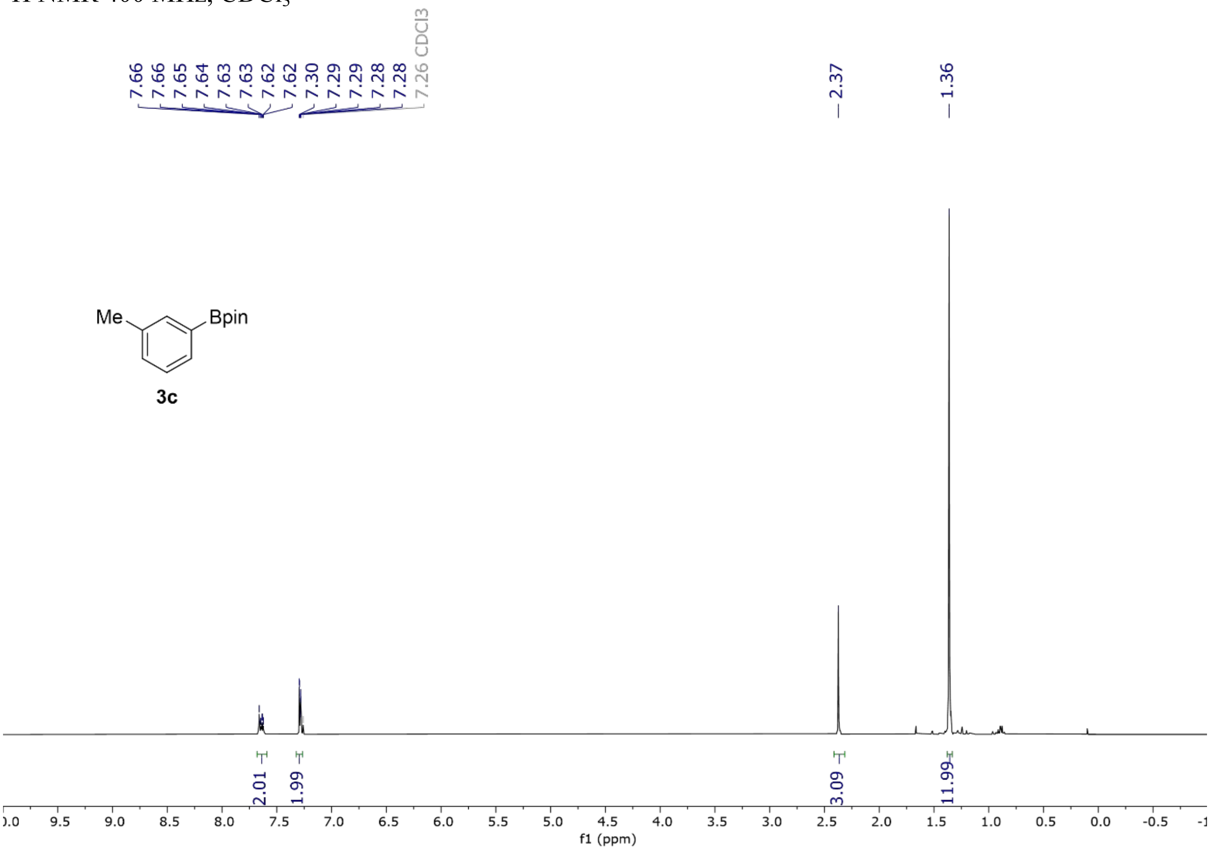

^{13}C NMR 101 MHz, $CDCl_3$

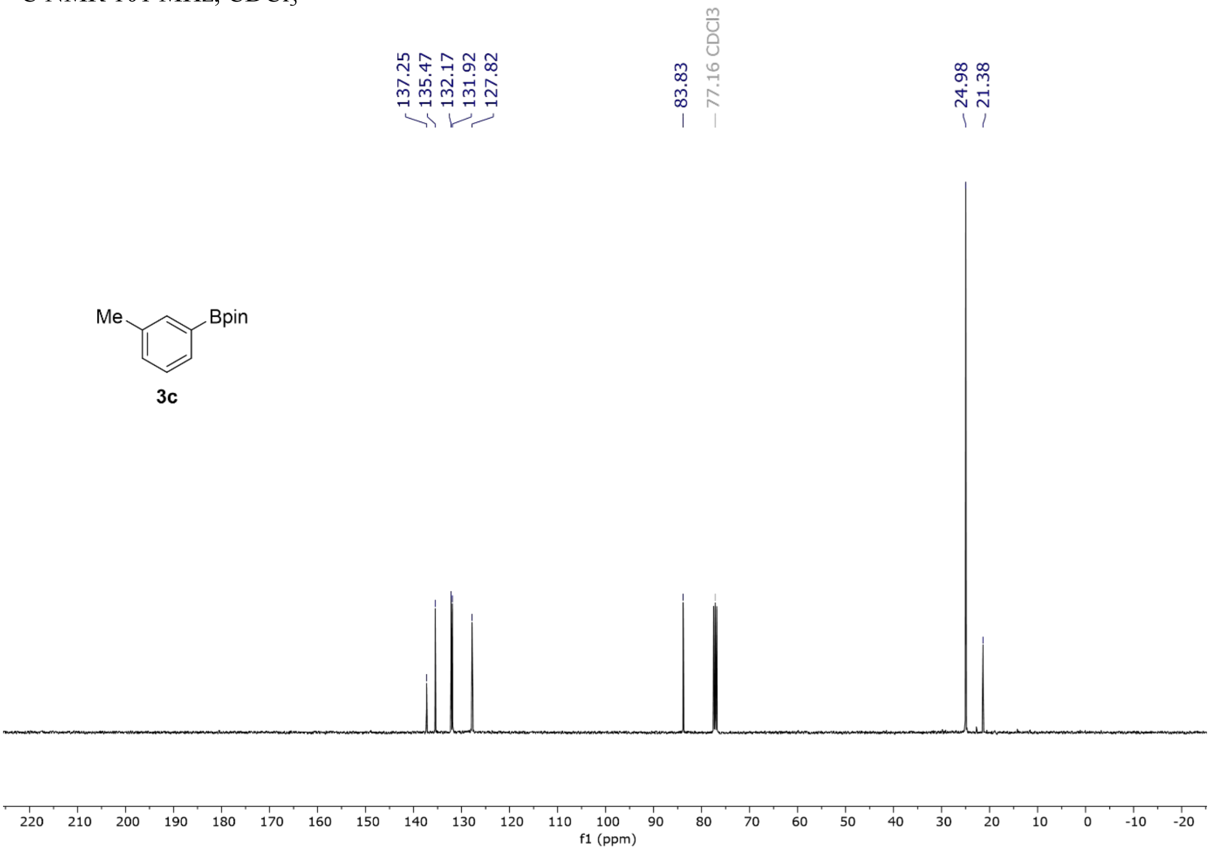

^{11}B NMR 128 MHz, $CDCl_3$

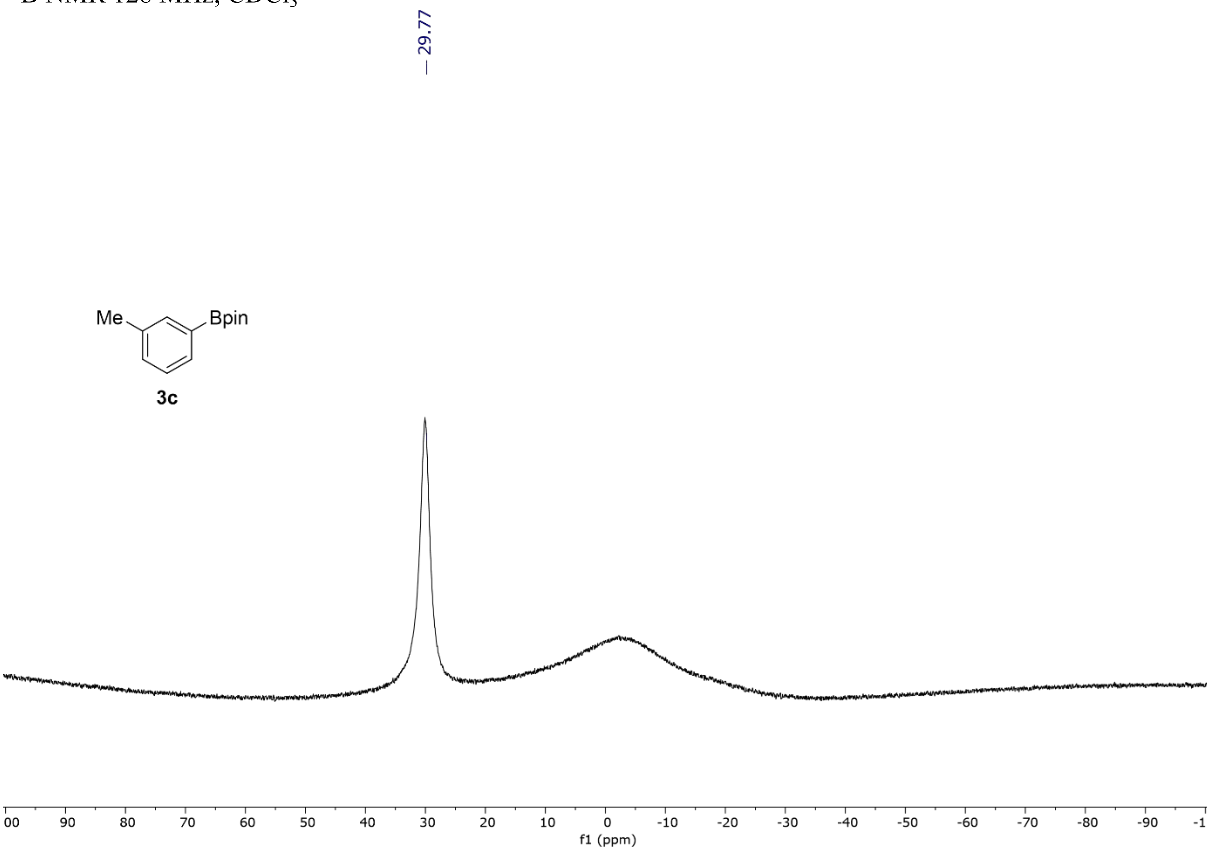

4,4,5,5-tetramethyl-2-(*p*-tolyl)-1,3,2-dioxaborolane (3d)

^{1}H NMR 400 MHz, $CDCl_3$

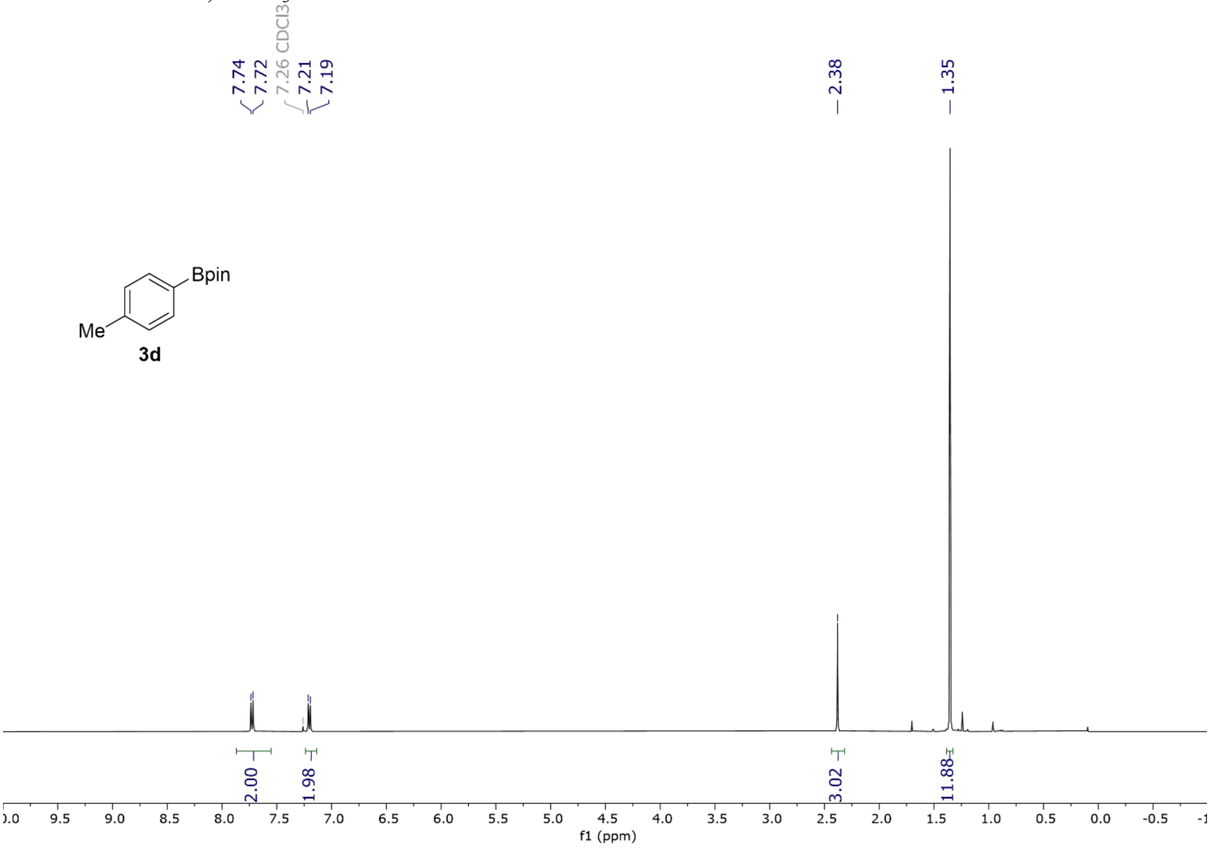

^{13}C NMR 101 MHz, $CDCl_3$

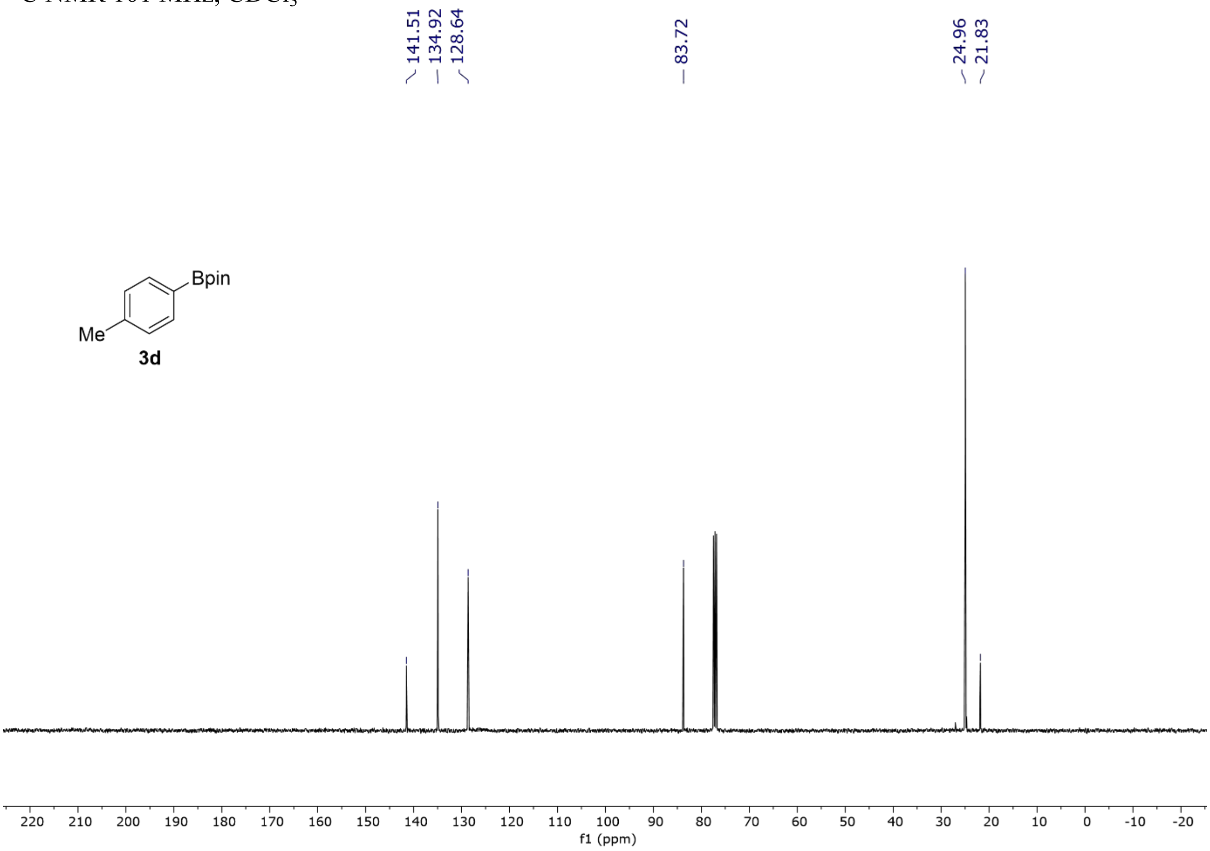

^{11}B NMR 128 MHz, $CDCl_3$

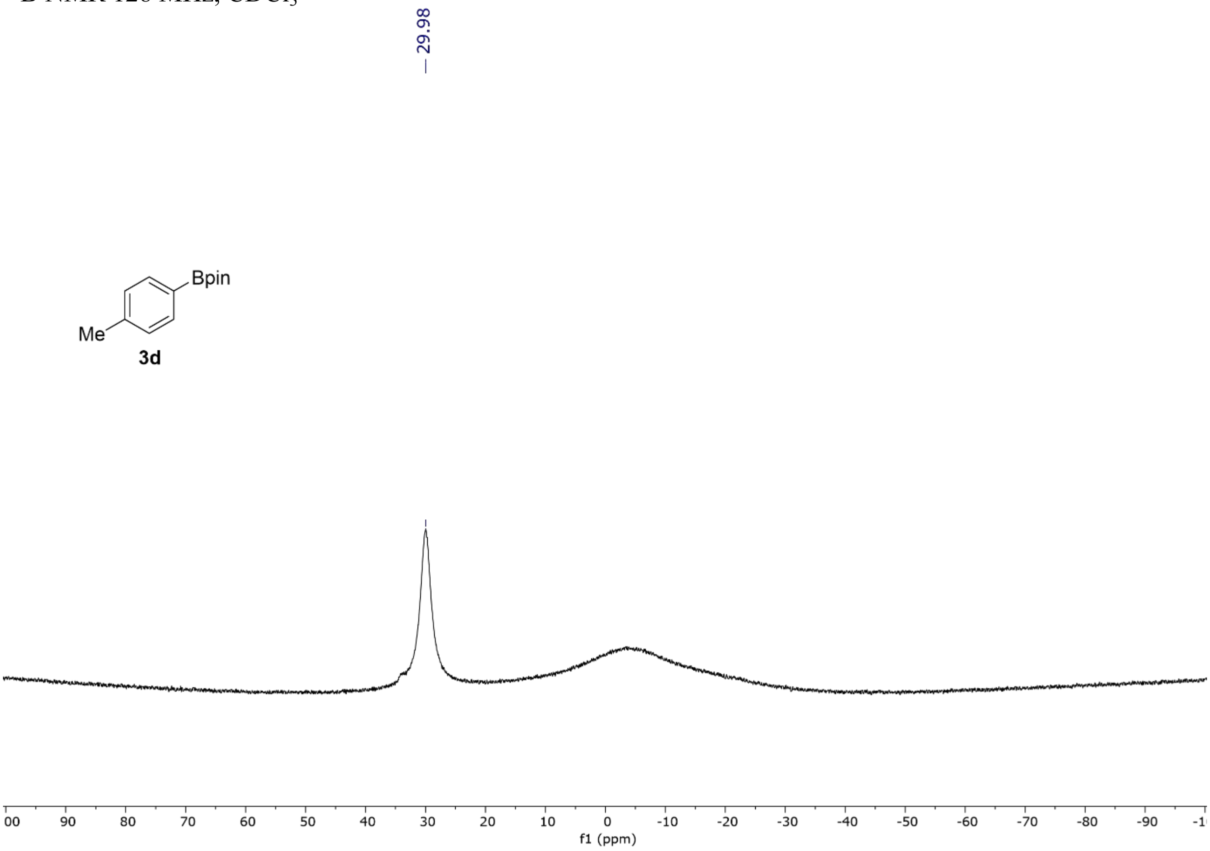

2-(4-methoxyphenyl)-4,4,5,5-tetramethyl-1,3,2-dioxaborolane (3e)

^{1}H NMR 400 MHz, $CDCl_3$

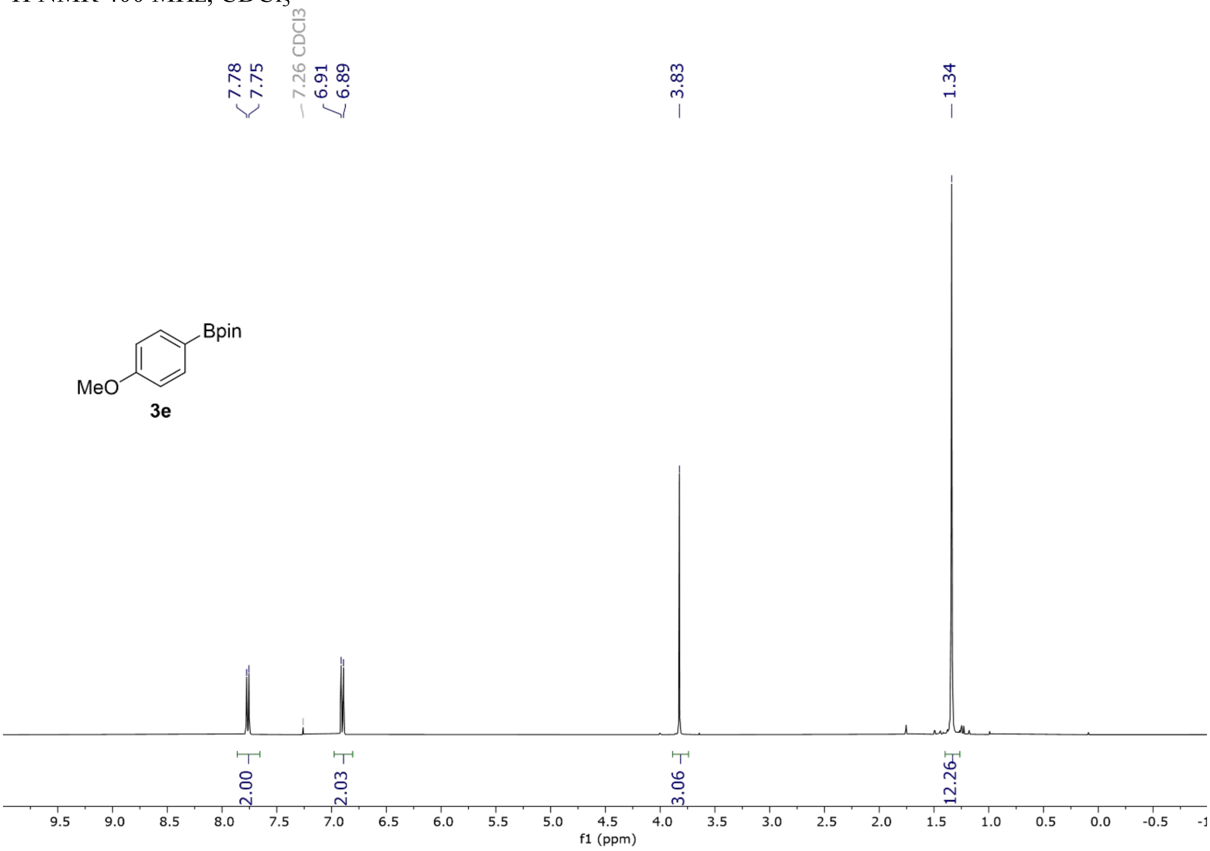

^{13}C NMR 101 MHz, $CDCl_3$

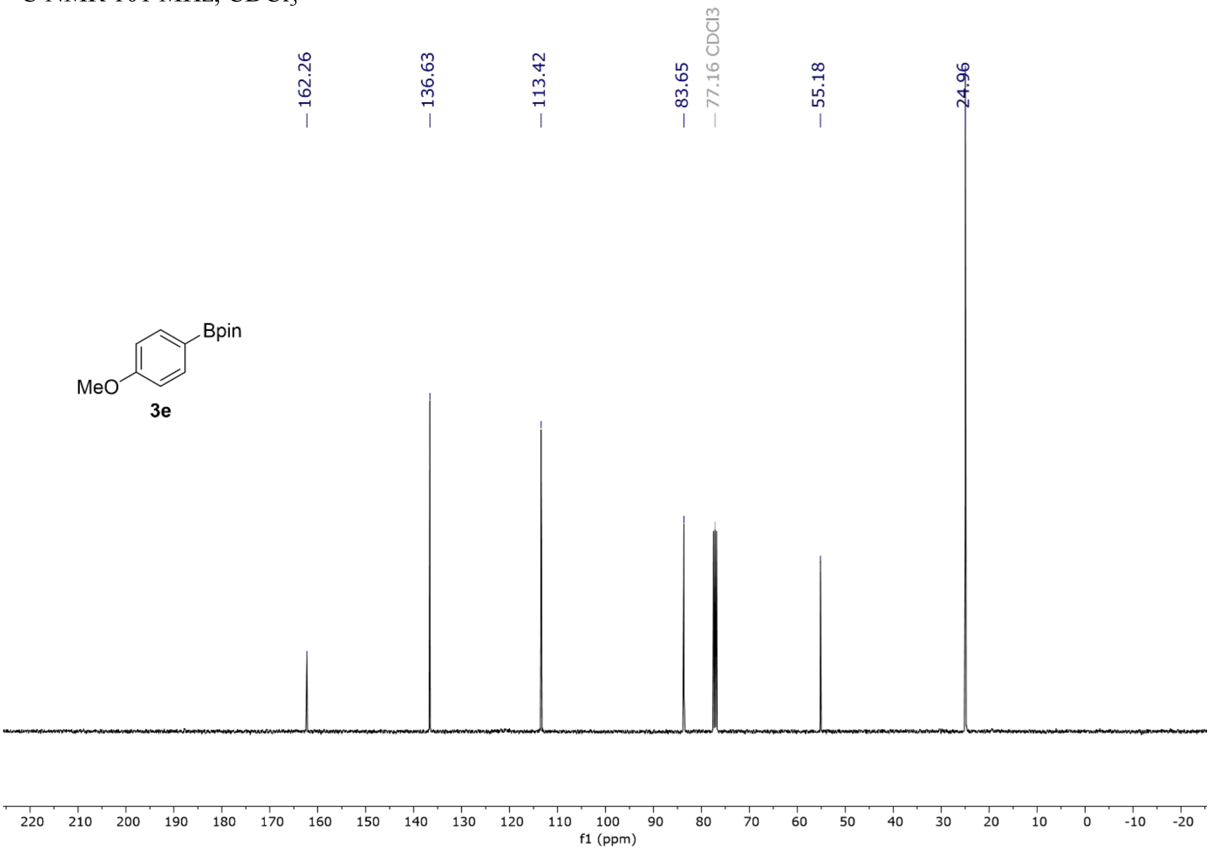

^{11}B NMR 128 MHz, $CDCl_3$

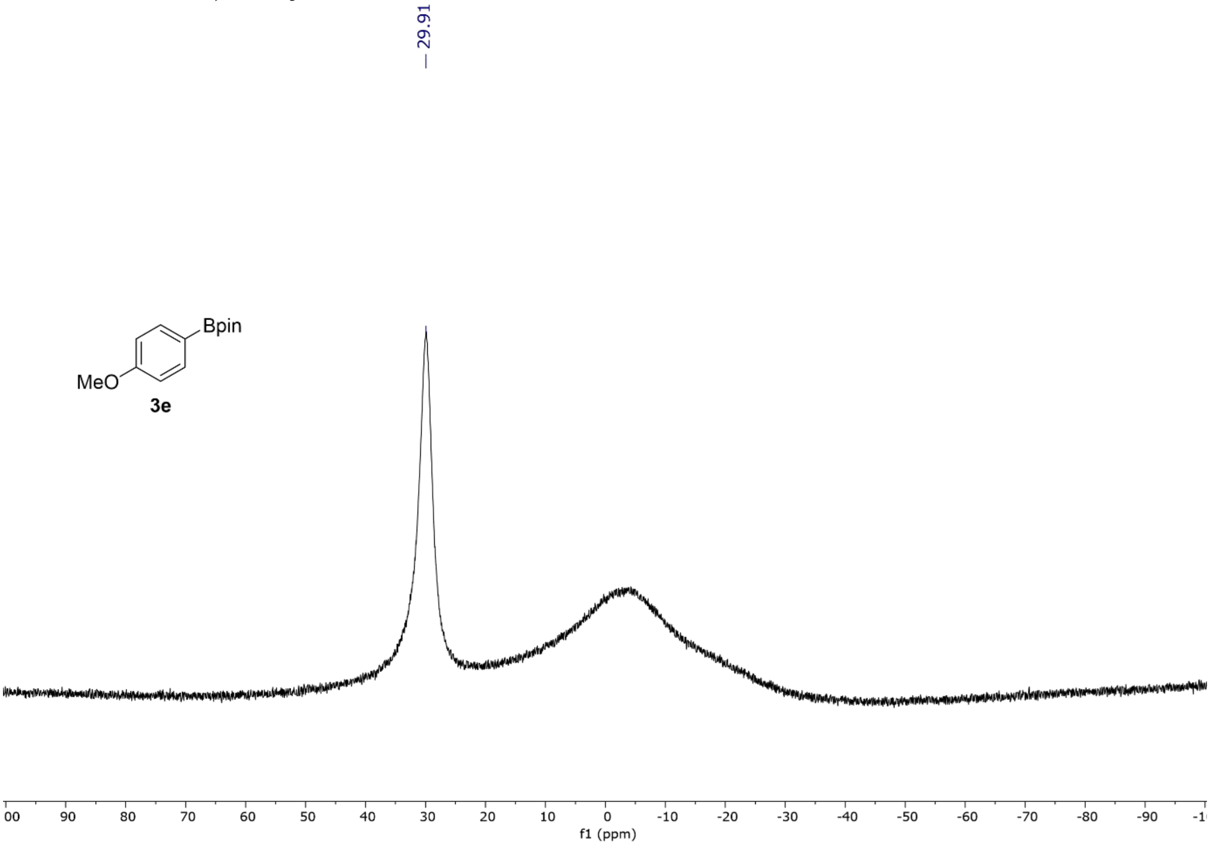

2-(4-fluorophenyl)-4,4,5,5-tetramethyl-1,3,2-dioxaborolane (3f)

^{1}H NMR 400 MHz, $CDCl_3$

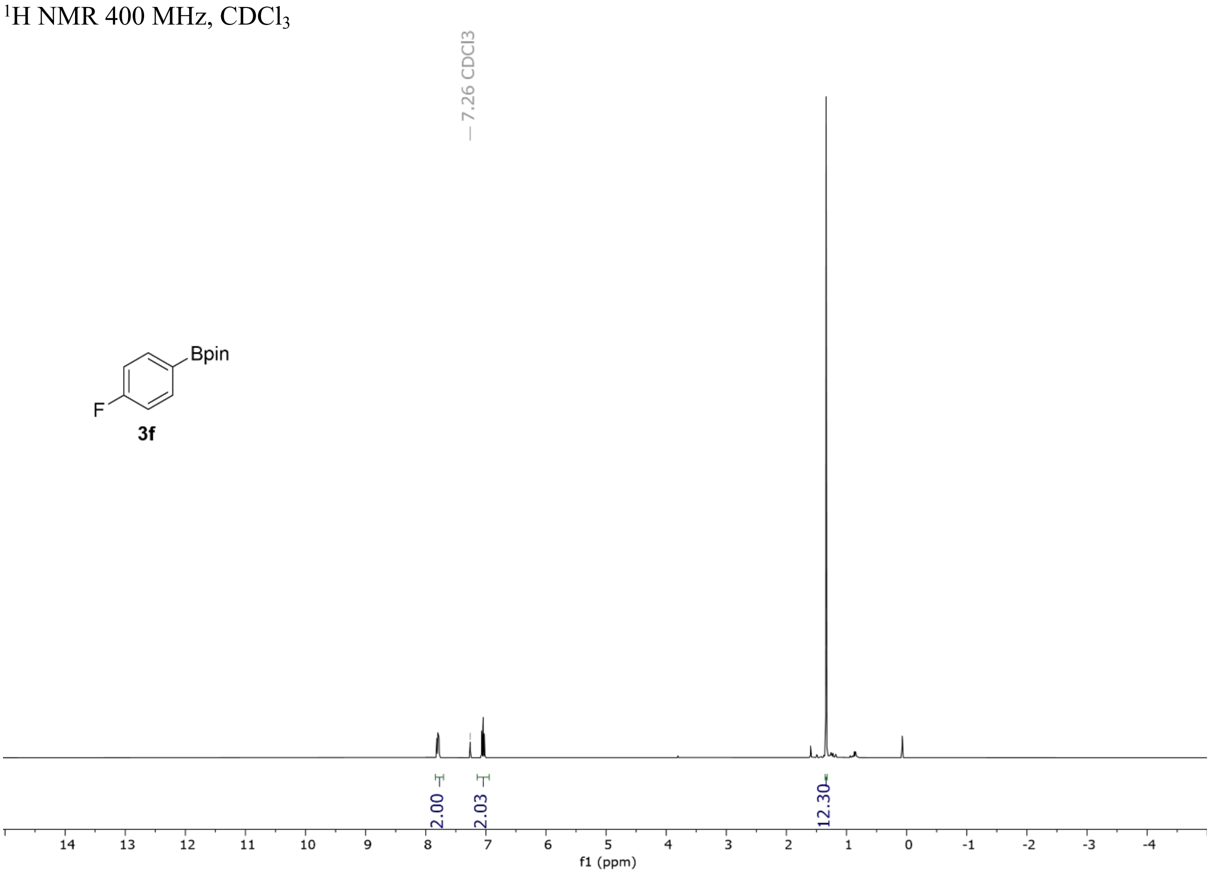

^{13}C NMR 101 MHz, $CDCl_3$

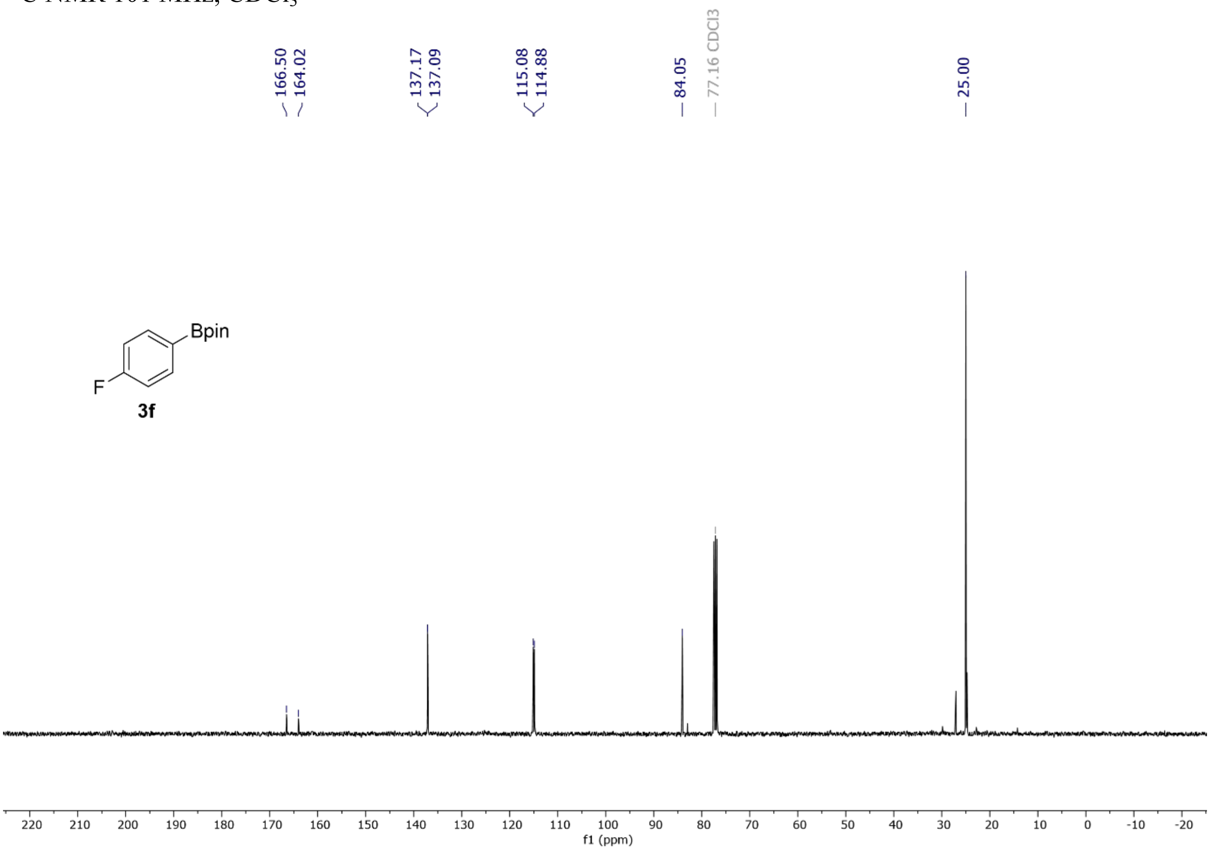

^{11}B NMR 128 MHz, $CDCl_3$

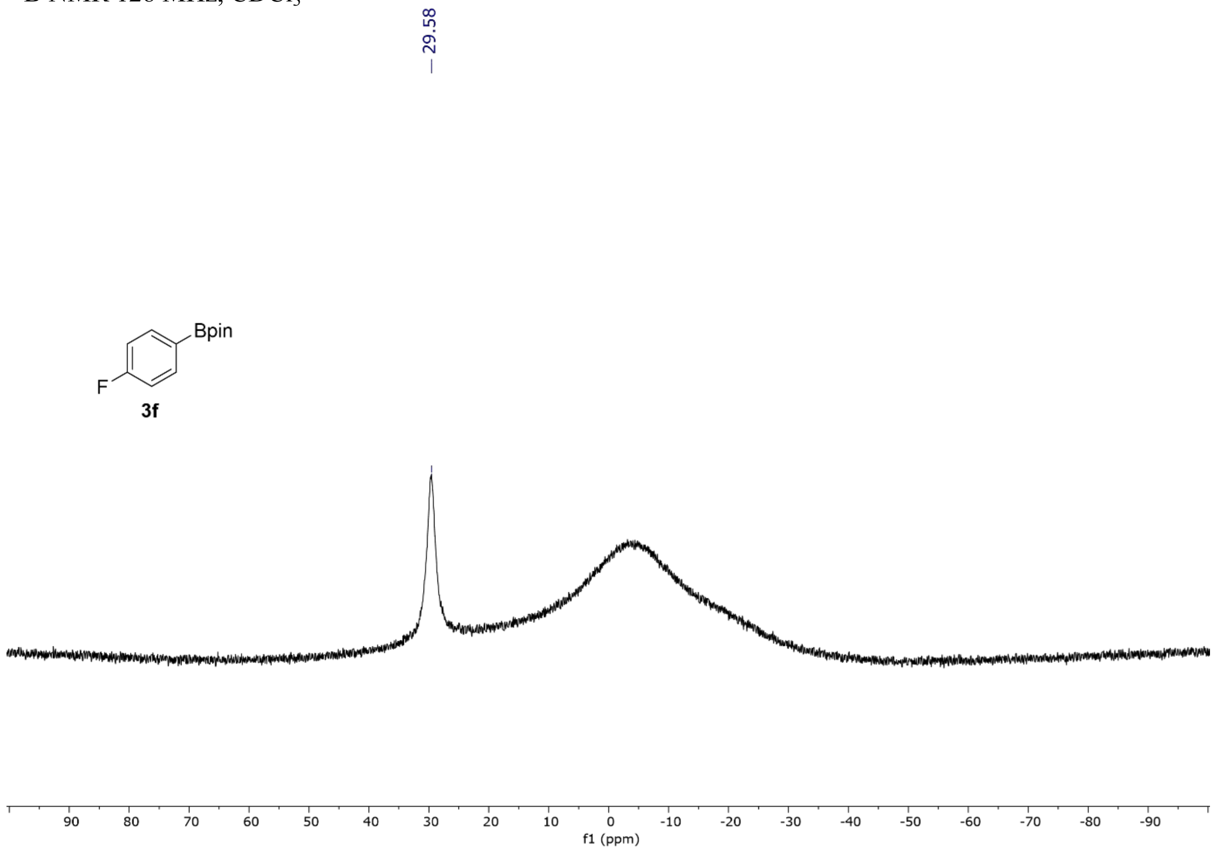

^{19}F NMR 377 MHz, $CDCl_3$

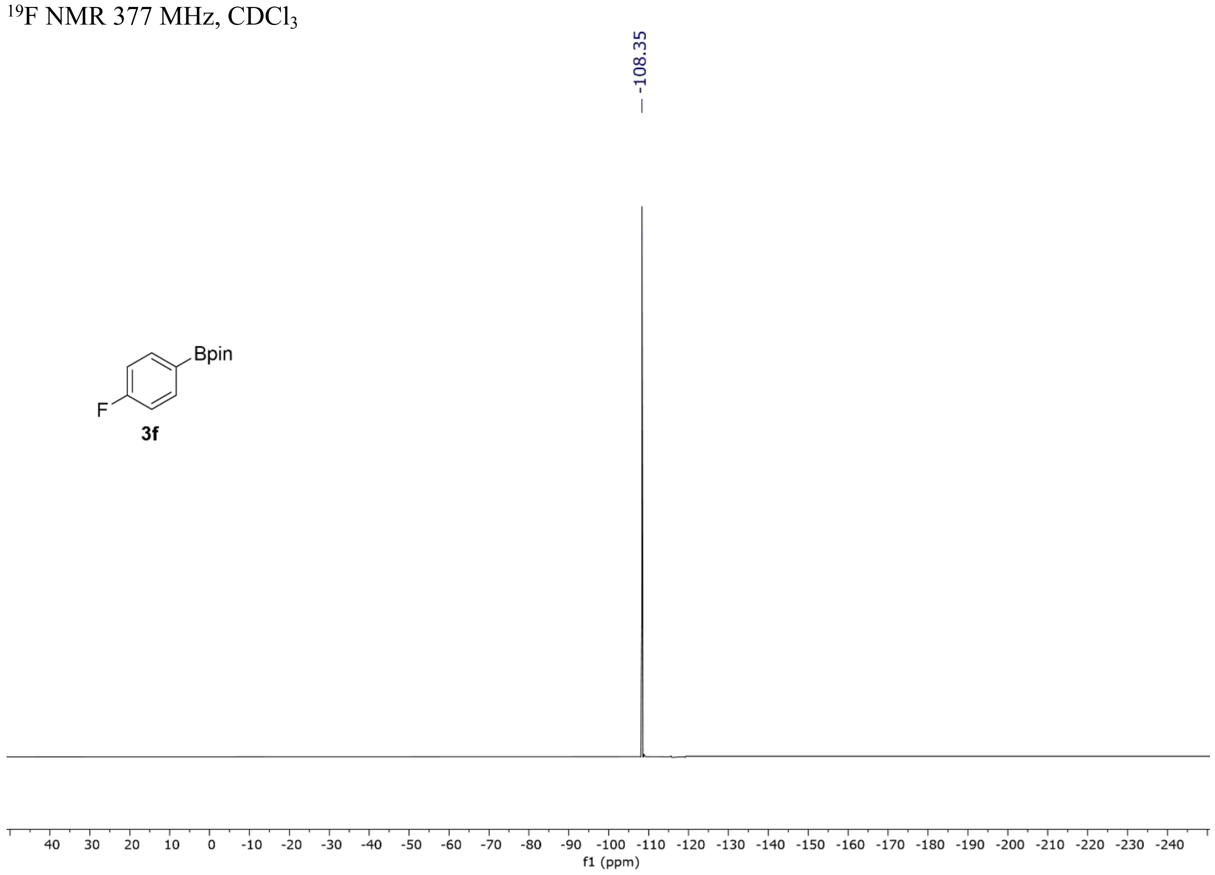

4,4,5,5-tetramethyl-2-(4-(trifluoromethyl)phenyl)-1,3,2-dioxaborolane (3g)

^{1}H NMR 400 MHz, $CDCl_3$

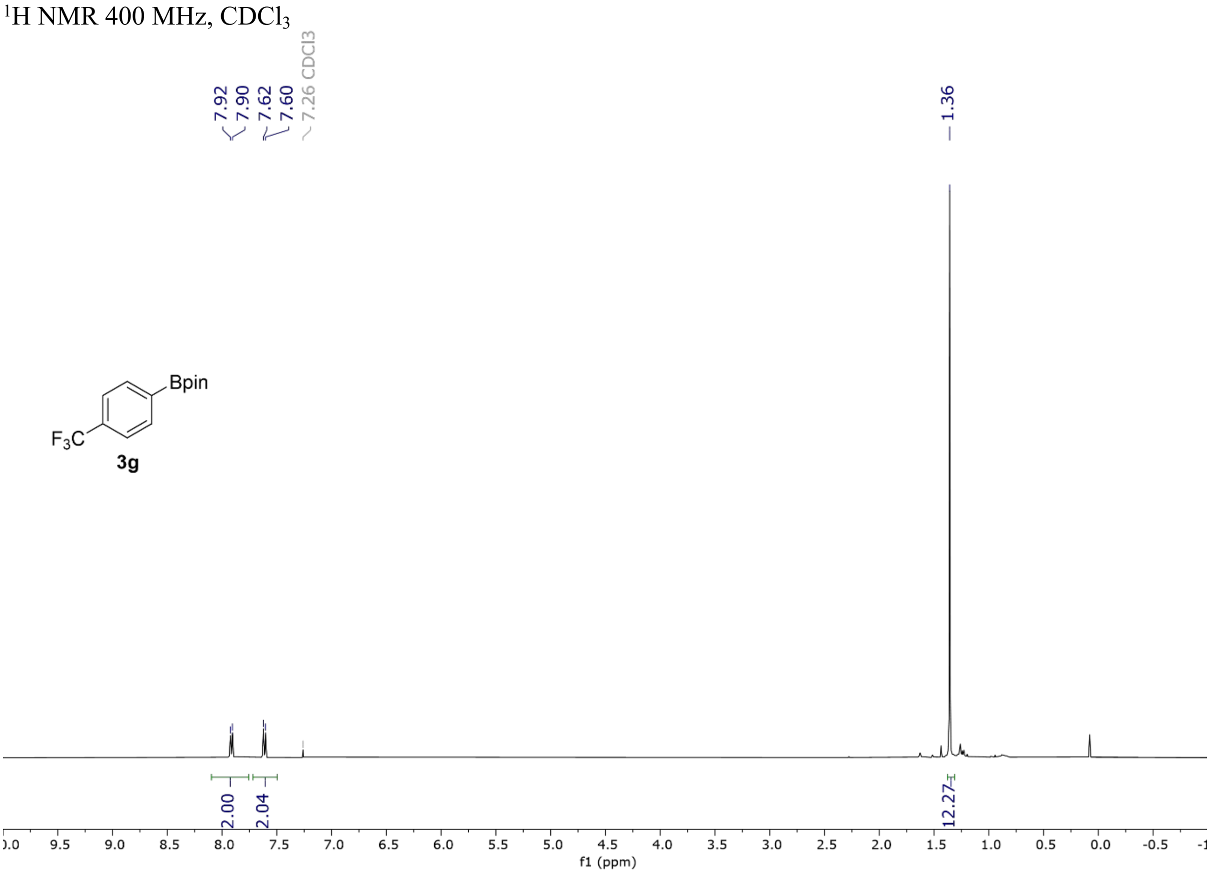

^{13}C NMR 101 MHz, $CDCl_3$

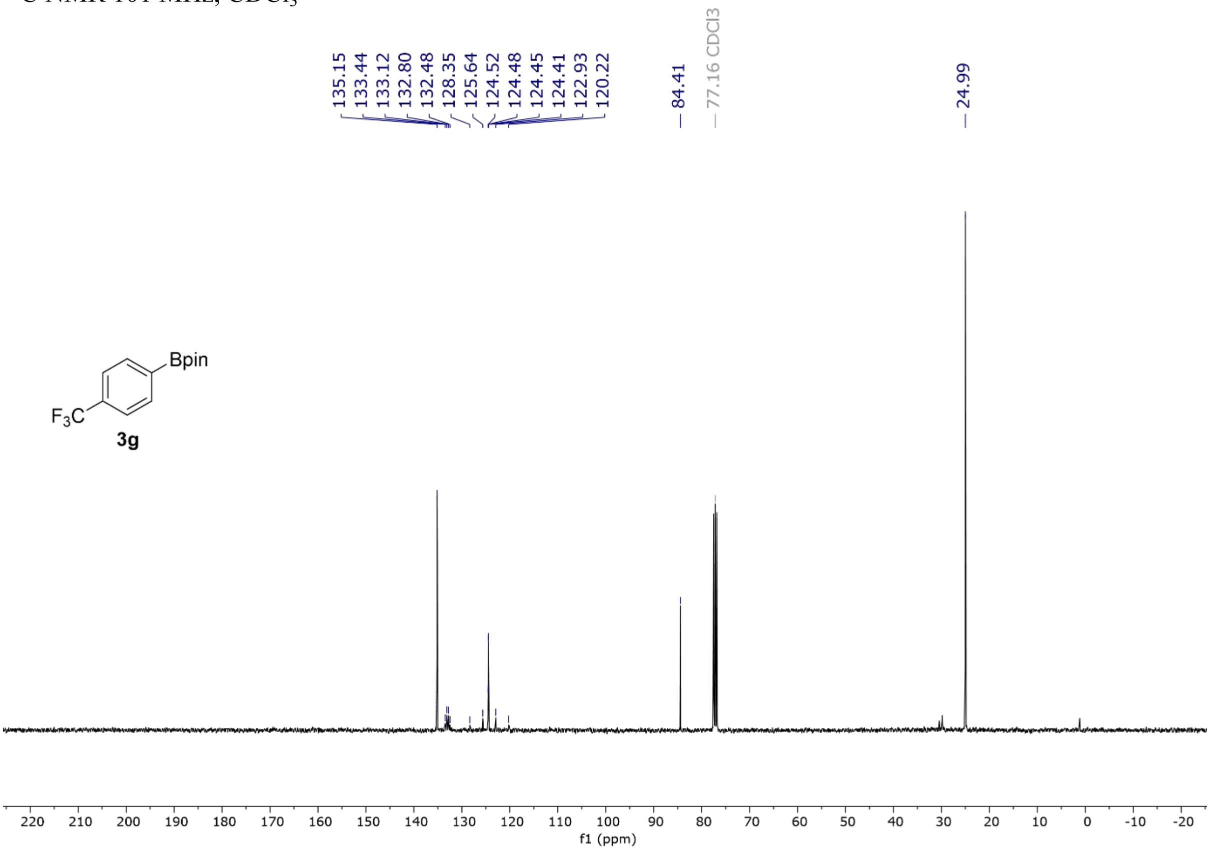

^{11}B NMR 128 MHz, $CDCl_3$

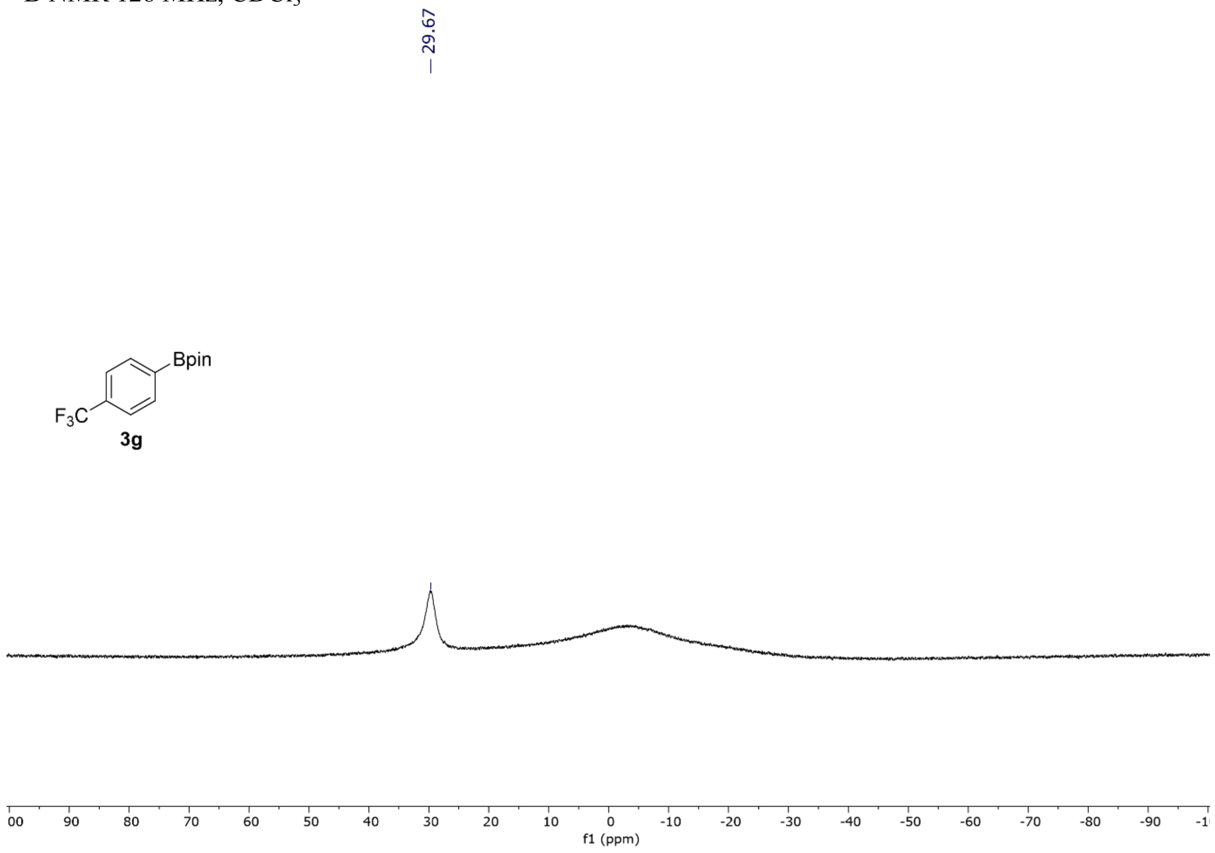

^{19}F NMR 377 MHz, $CDCl_3$

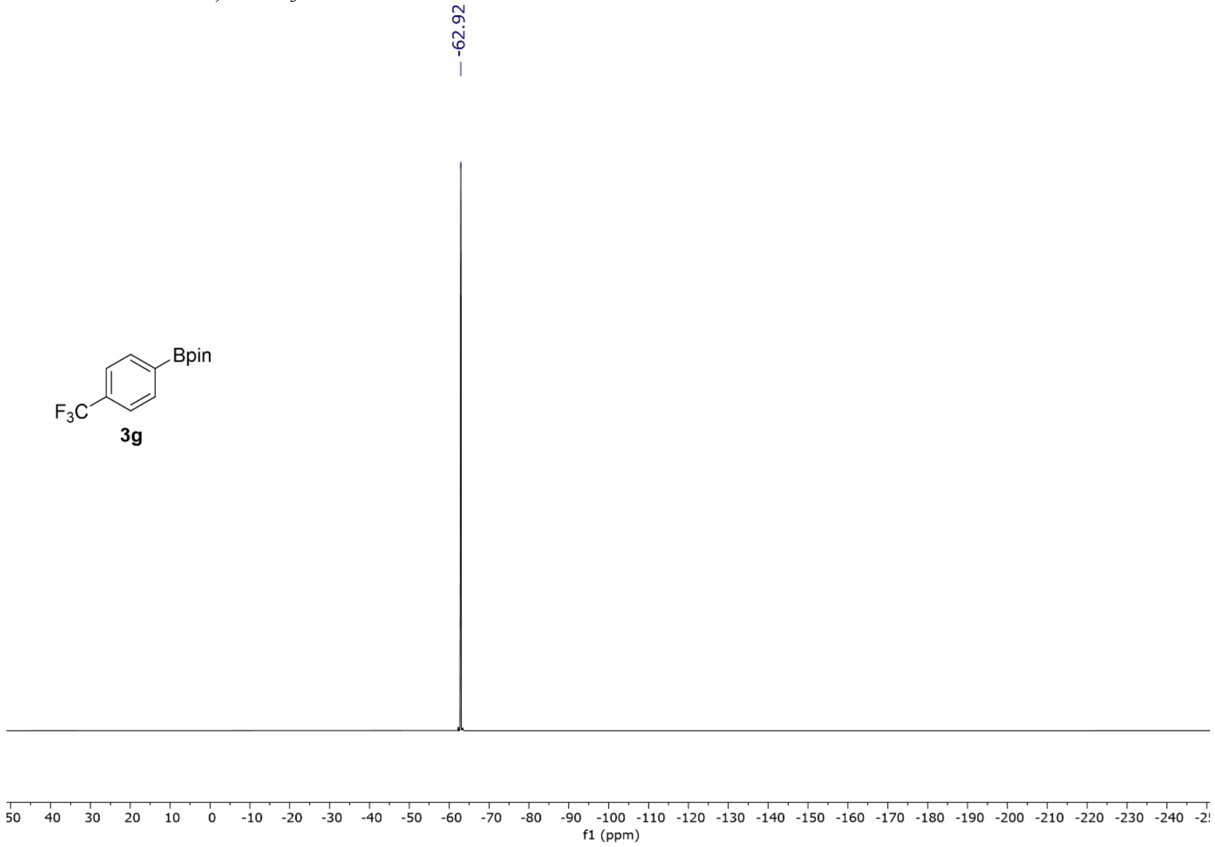

4,4,5,5-tetramethyl-2-(naphthalen-1-yl)-1,3,2-dioxaborolane (3h)

^{1}H NMR 400 MHz, $CDCl_3$

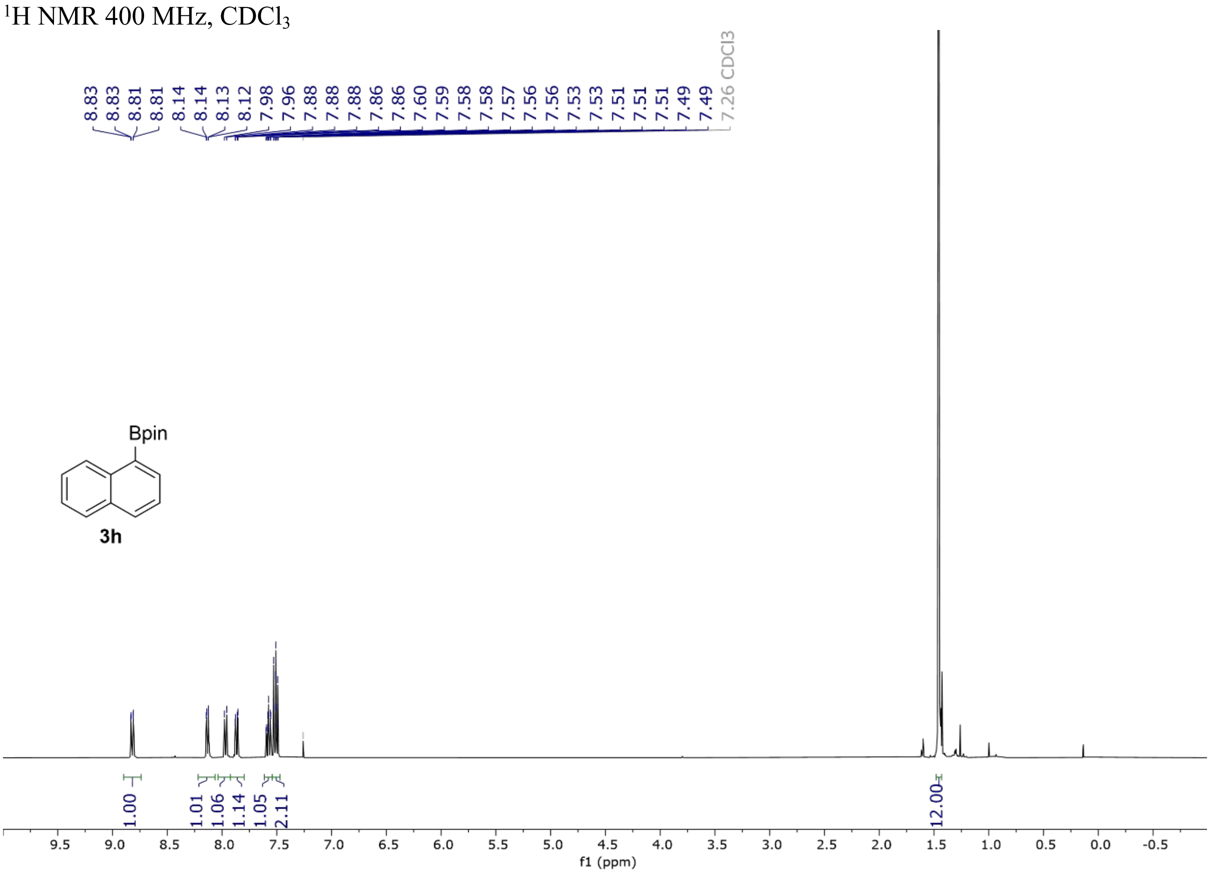

^{13}C NMR 101 MHz, $CDCl_3$

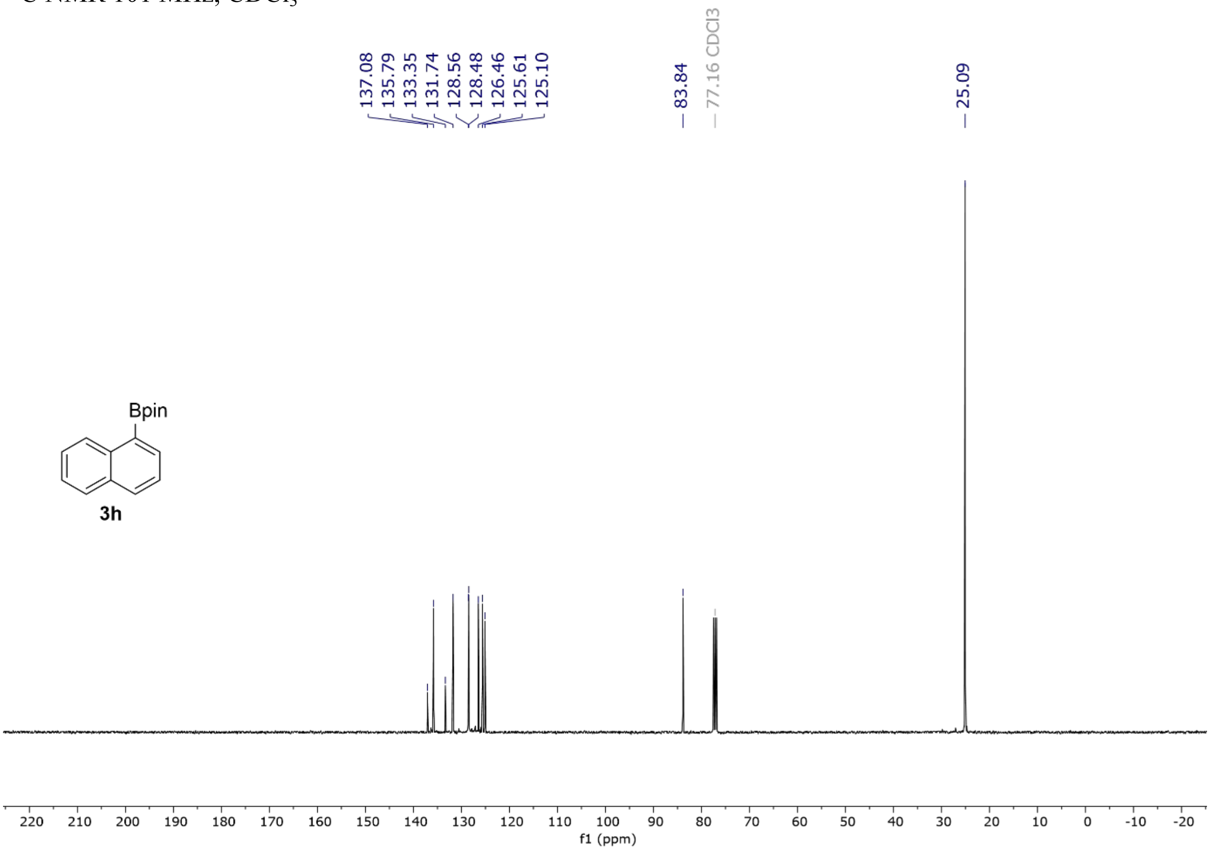

^{11}B NMR 128 MHz, $CDCl_3$

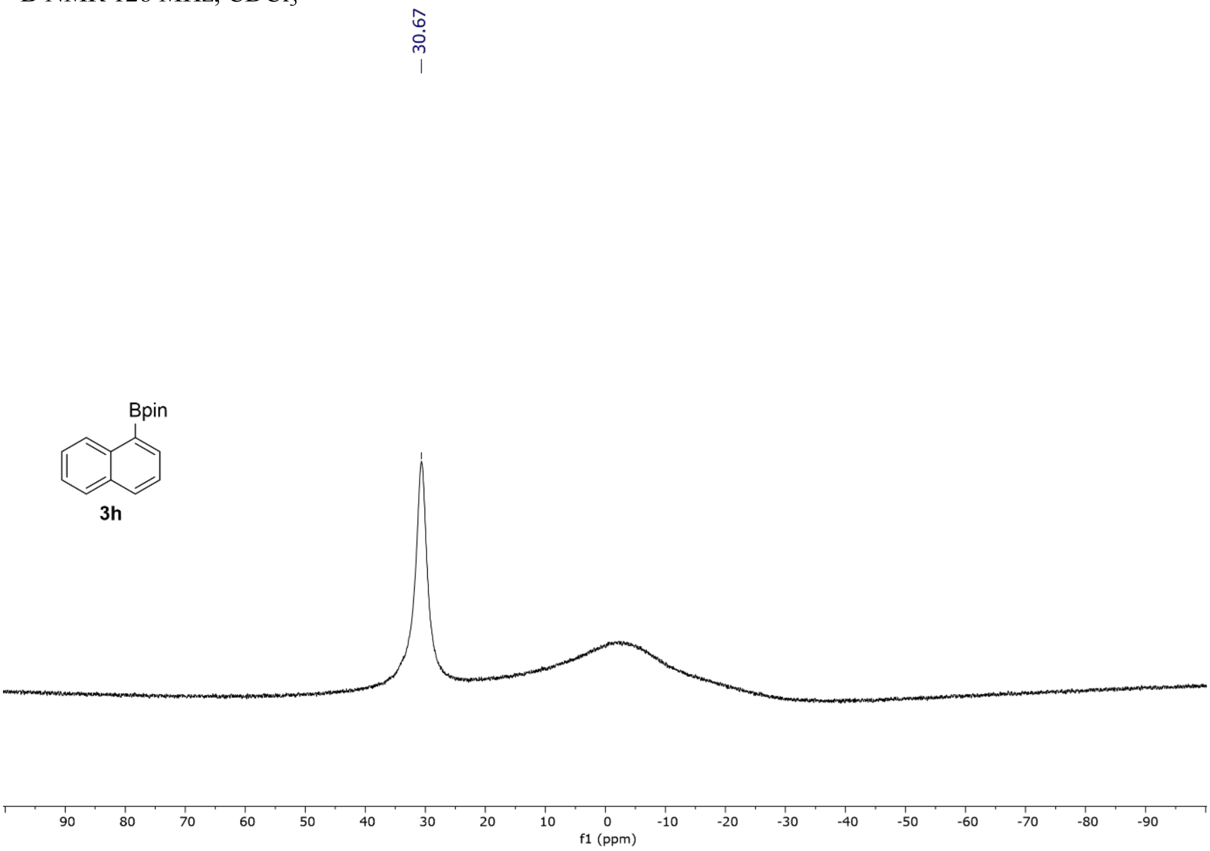

4,4,5,5-tetramethyl-2-(naphthalen-2-yl)-1,3,2-dioxaborolane (3i)

^{1}H NMR 400 MHz, $CDCl_3$

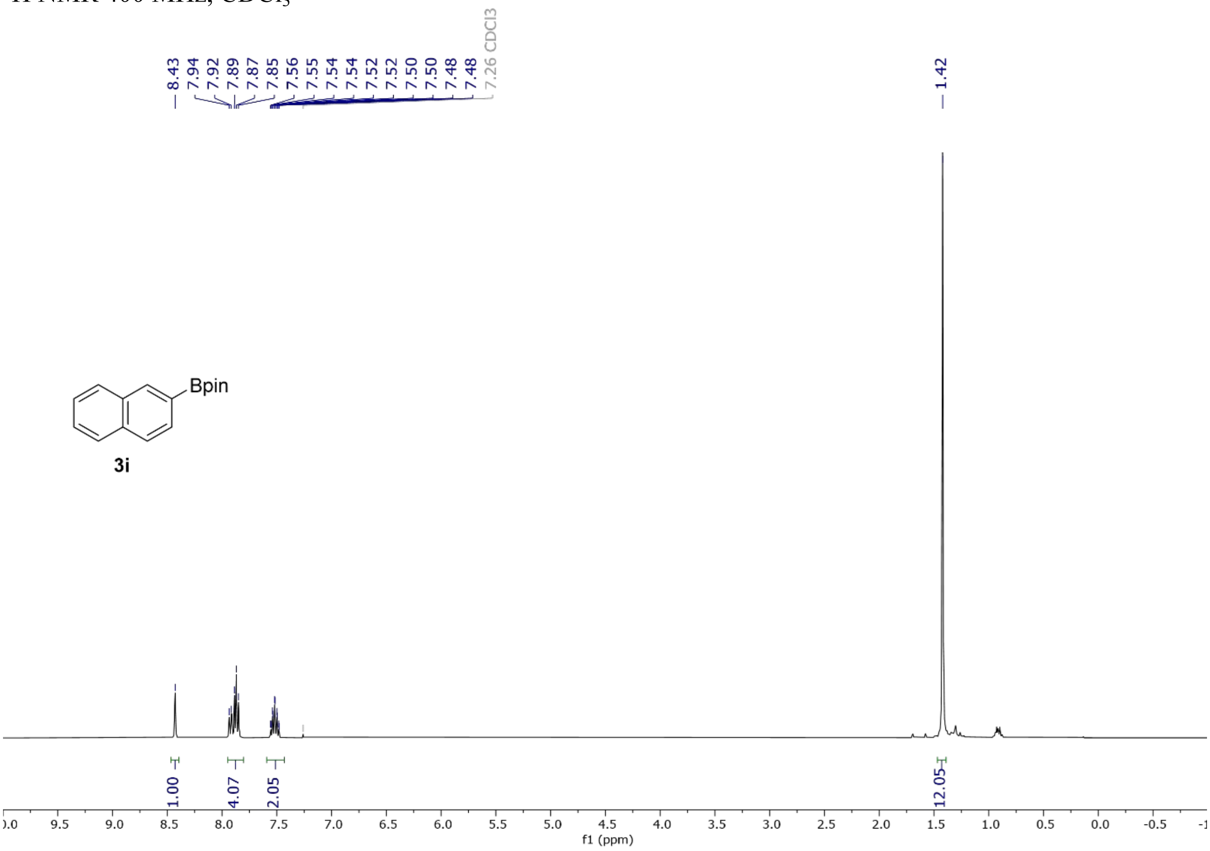

^{13}C NMR 101 MHz, $CDCl_3$

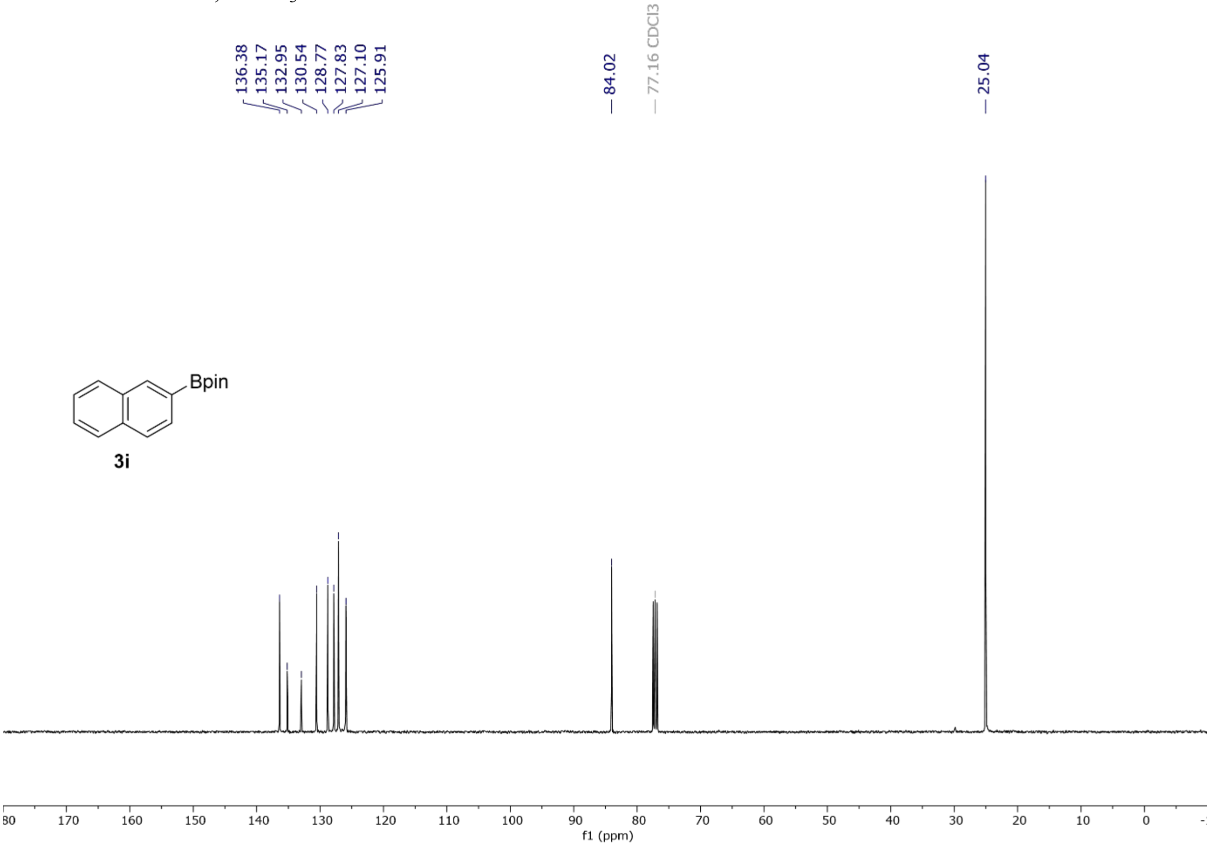

^{11}B NMR 128 MHz, $CDCl_3$

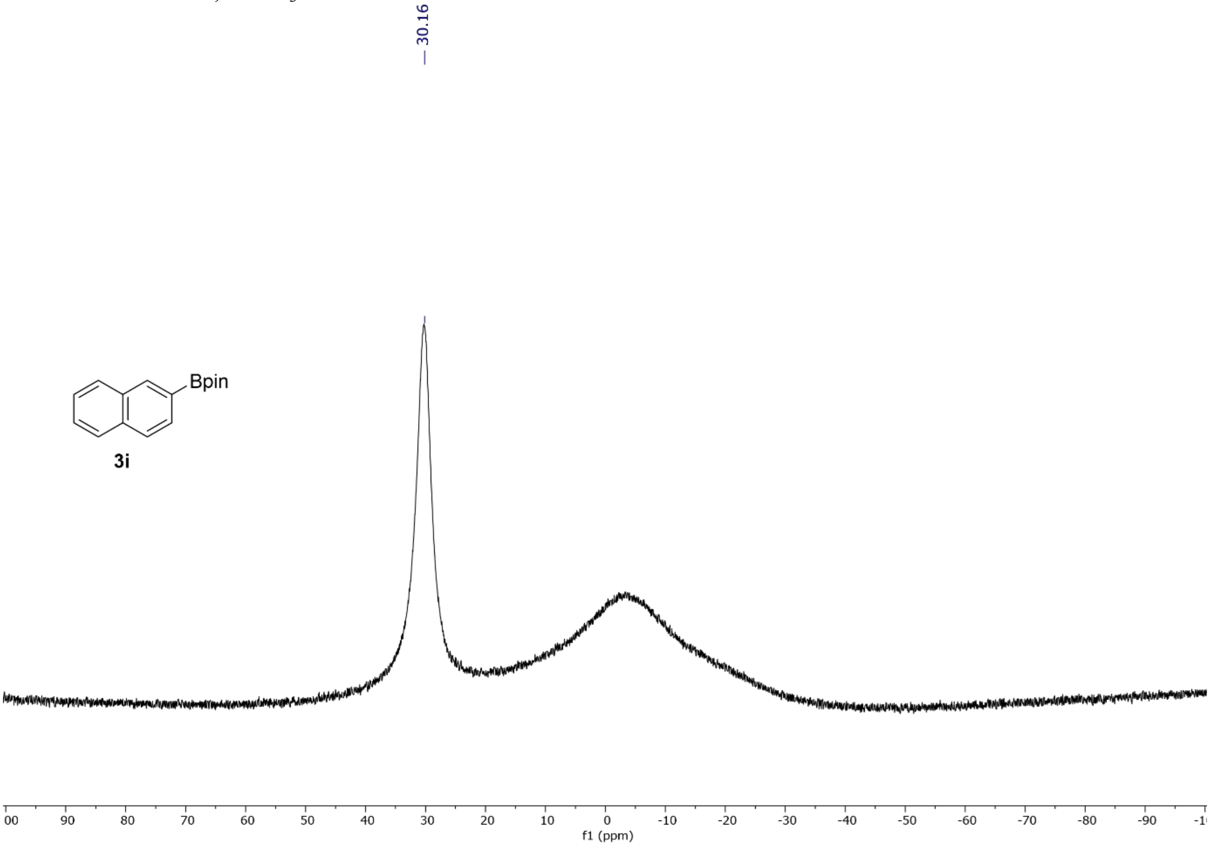

N,N-dimethyl-3-(4,4,5,5-tetramethyl-1,3,2-dioxaborolan-2-yl)aniline (3k)

^{1}H NMR 400 MHz, $CDCl_3$

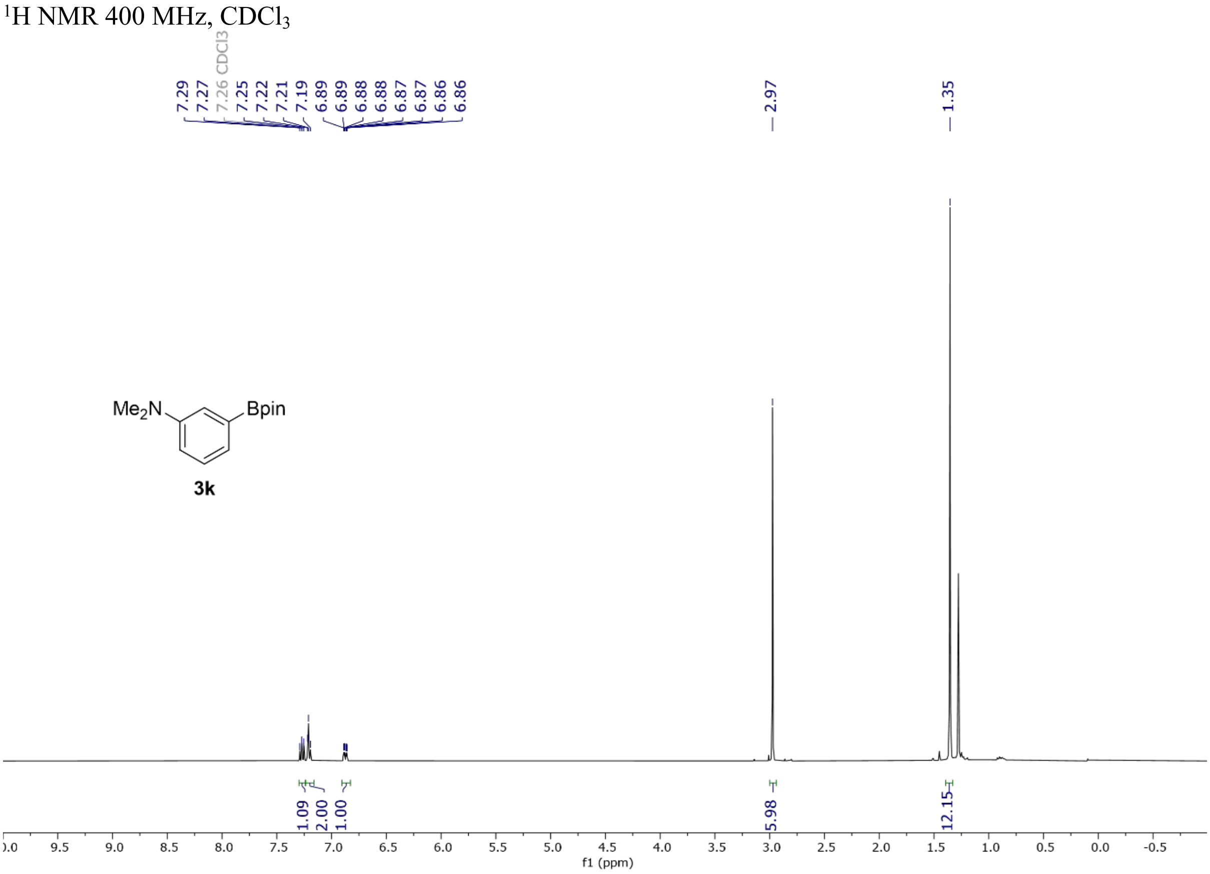

^{13}C NMR 101 MHz, $CDCl_3$

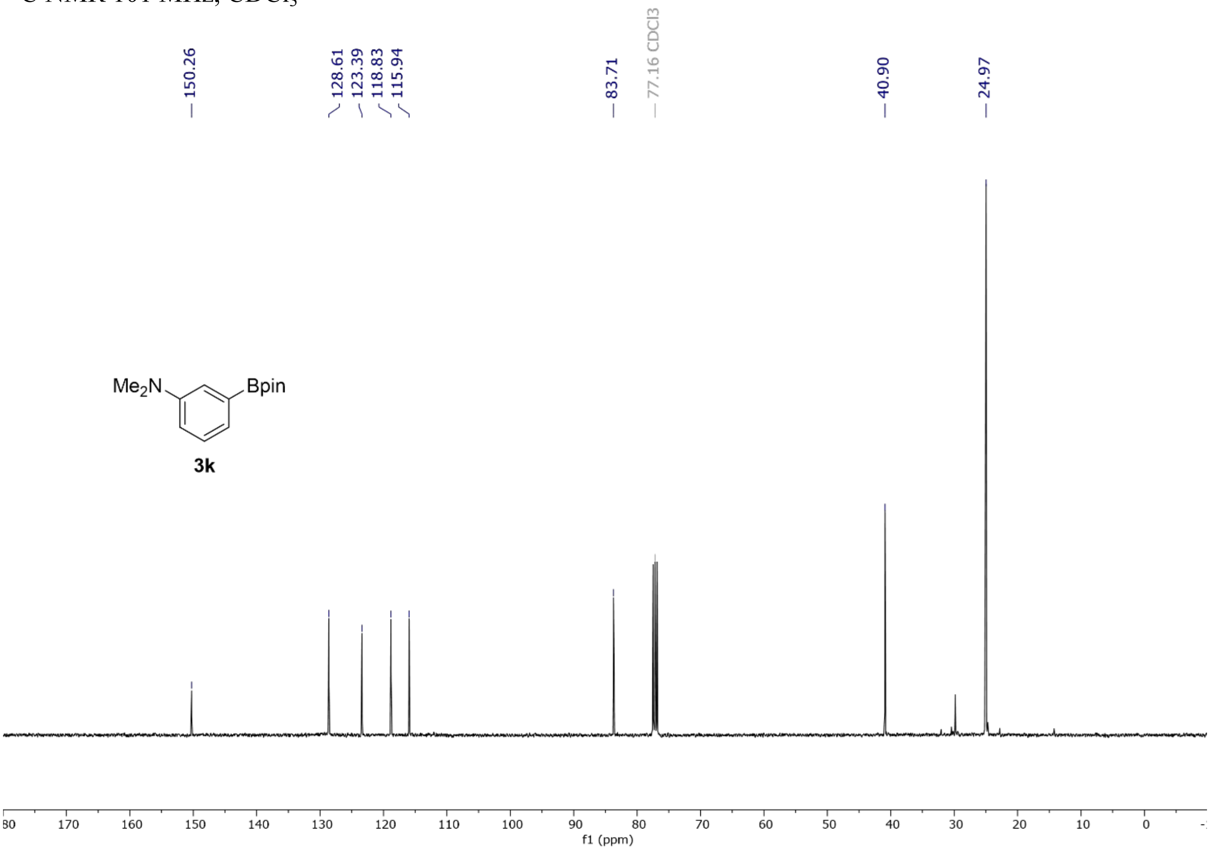

^{11}B NMR 128 MHz, $CDCl_3$

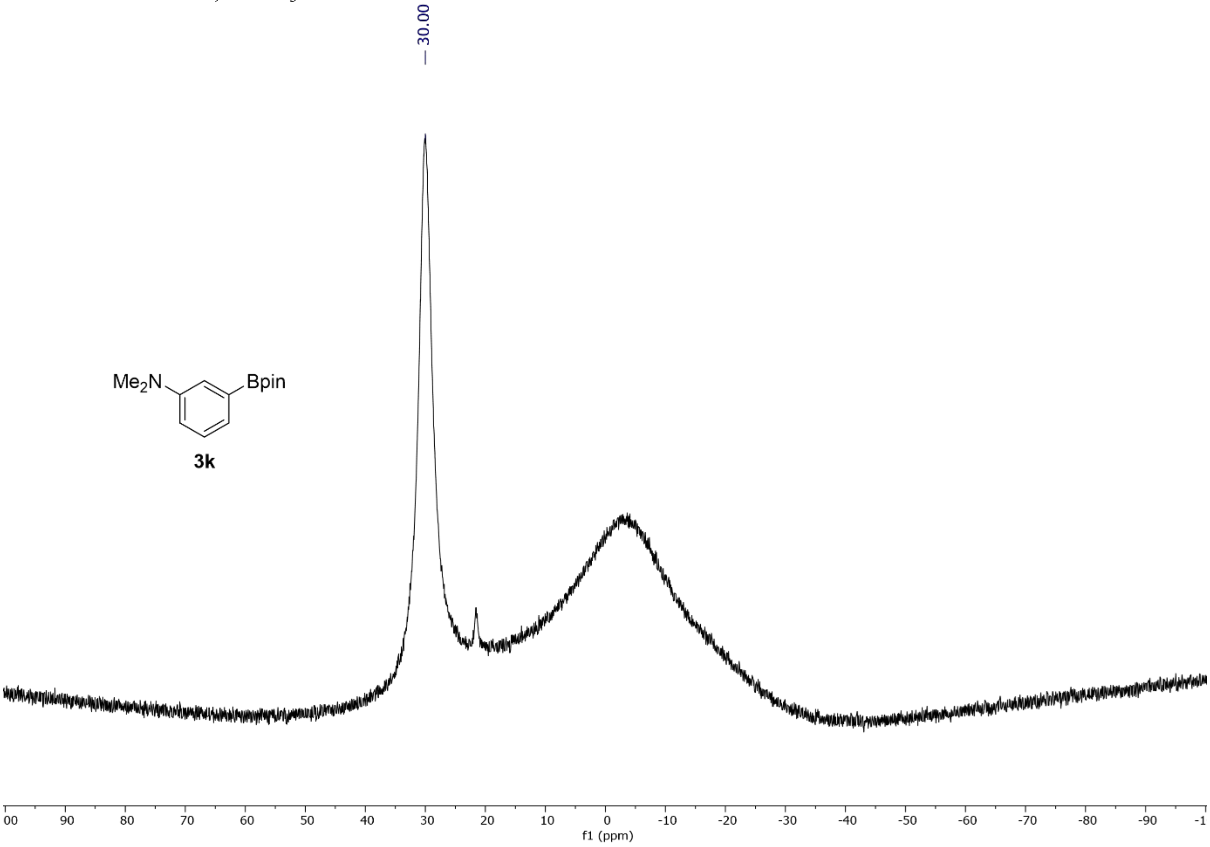

ethyl 4-(4,4,5,5-tetramethyl-1,3,2-dioxaborolan-2-yl)benzoate (3l)

^{1}H NMR 400 MHz, $CDCl_3$

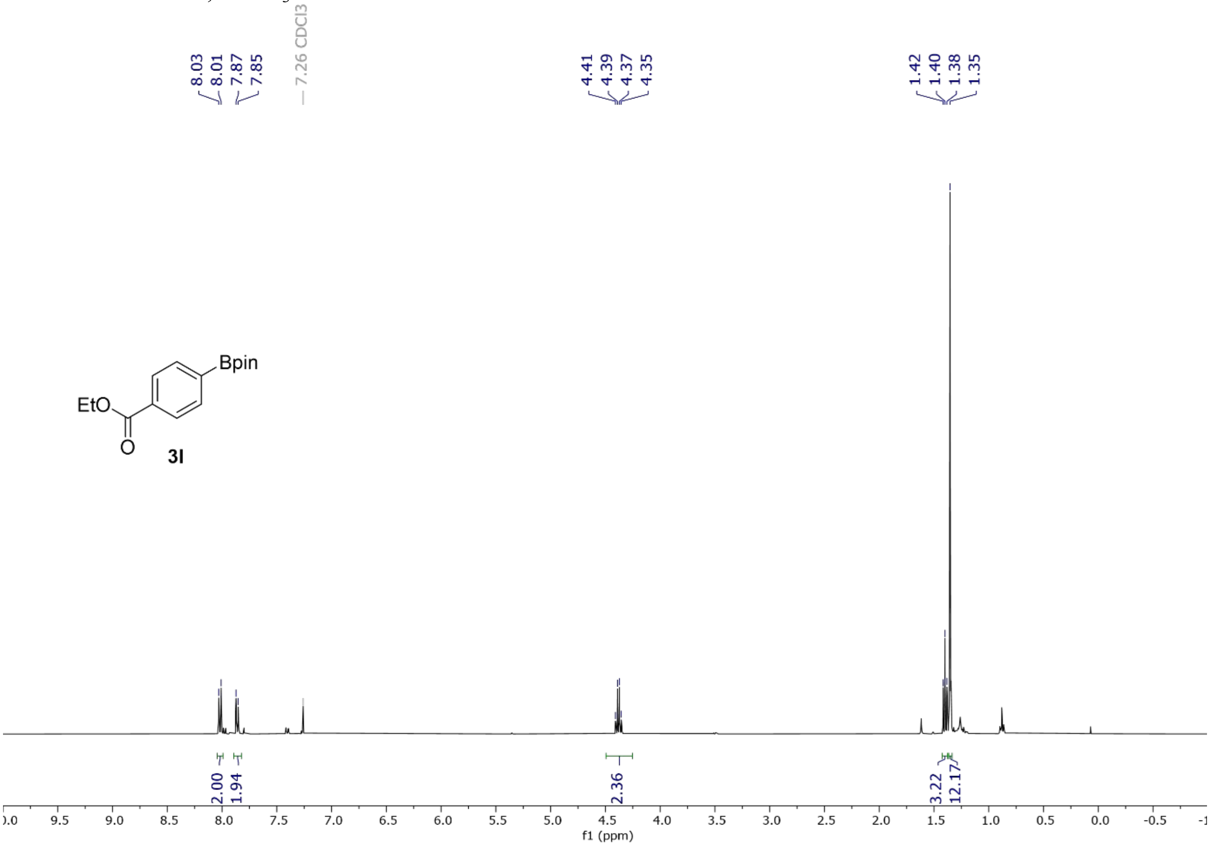

^{13}C NMR 101 MHz, $CDCl_3$

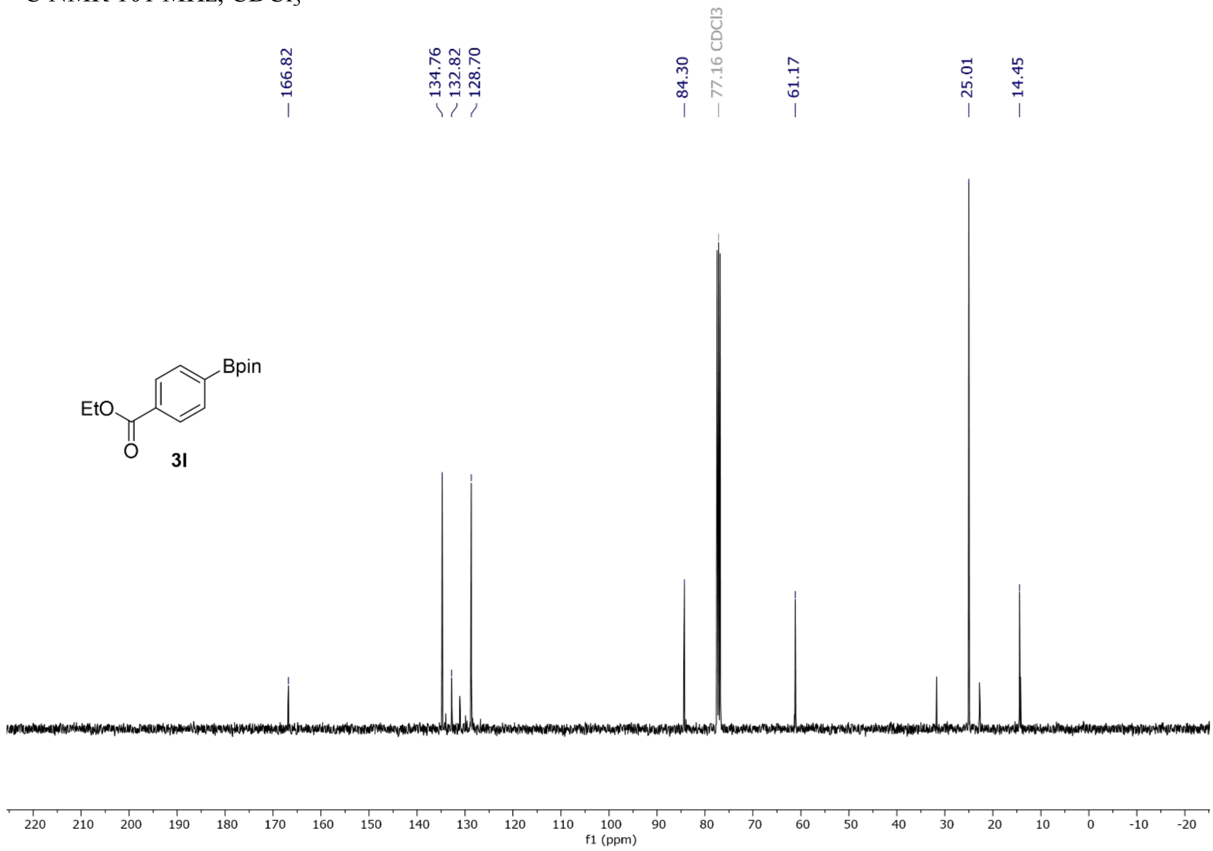

^{11}B NMR 128 MHz, $CDCl_3$

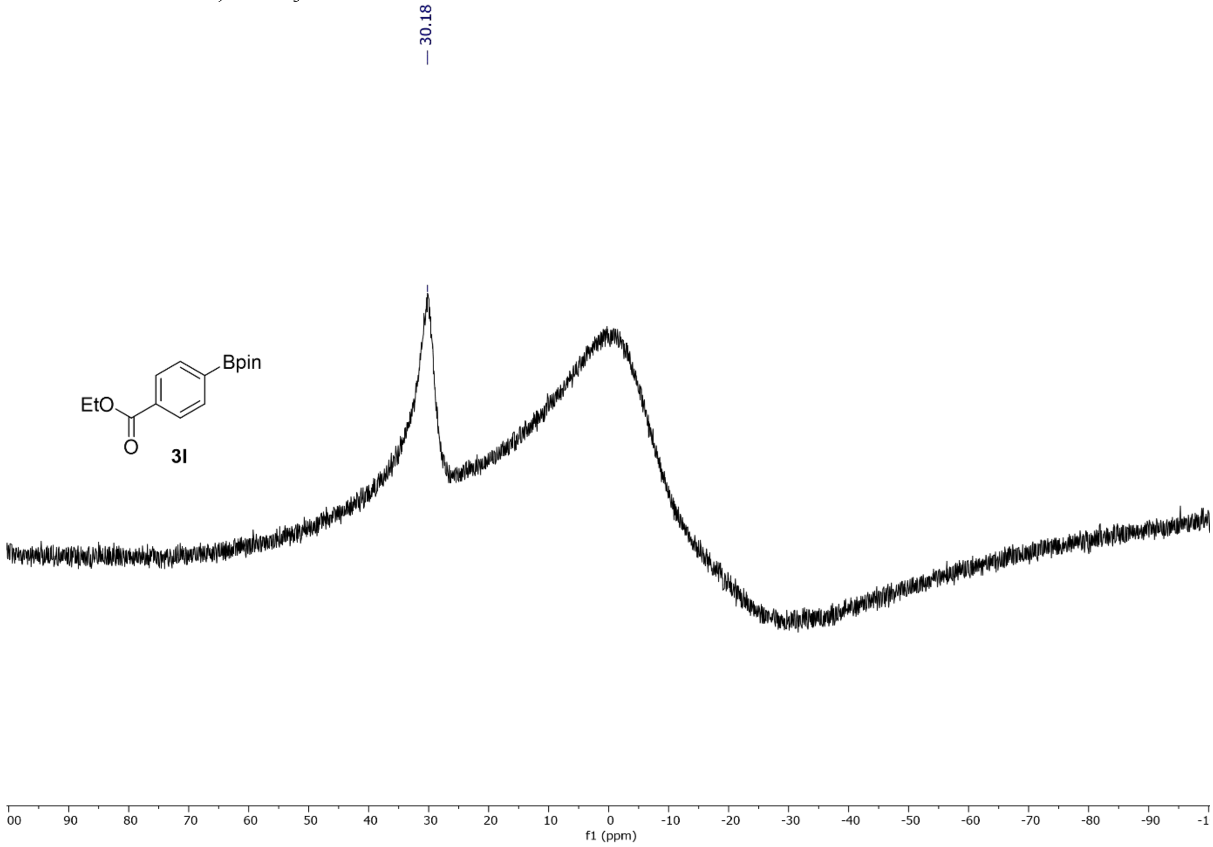

N,N-dimethyl-4-(4,4,5,5-tetramethyl-1,3,2-dioxaborolan-2-yl)benzamide (3m)

^{1}H NMR 400 MHz, $CDCl_3$

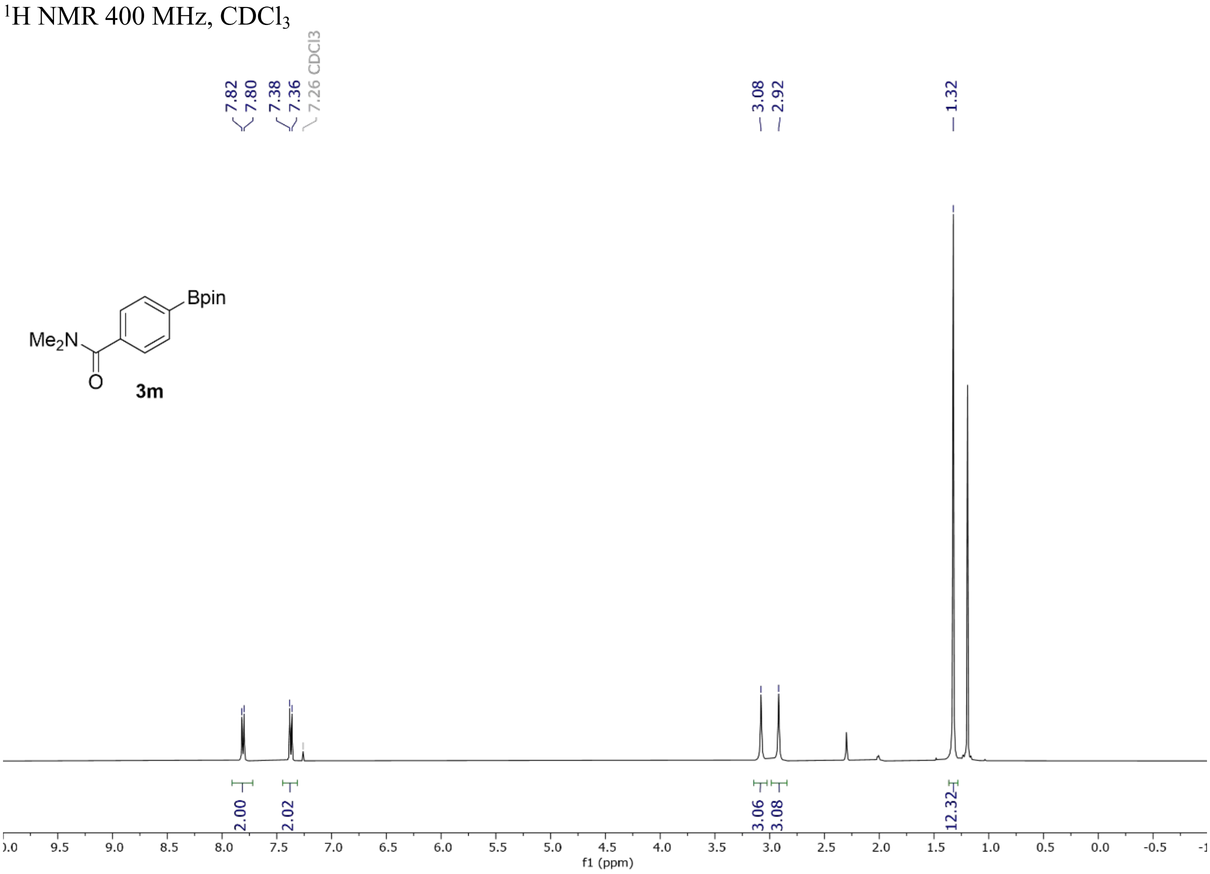

^{13}C NMR 101 MHz, $CDCl_3$

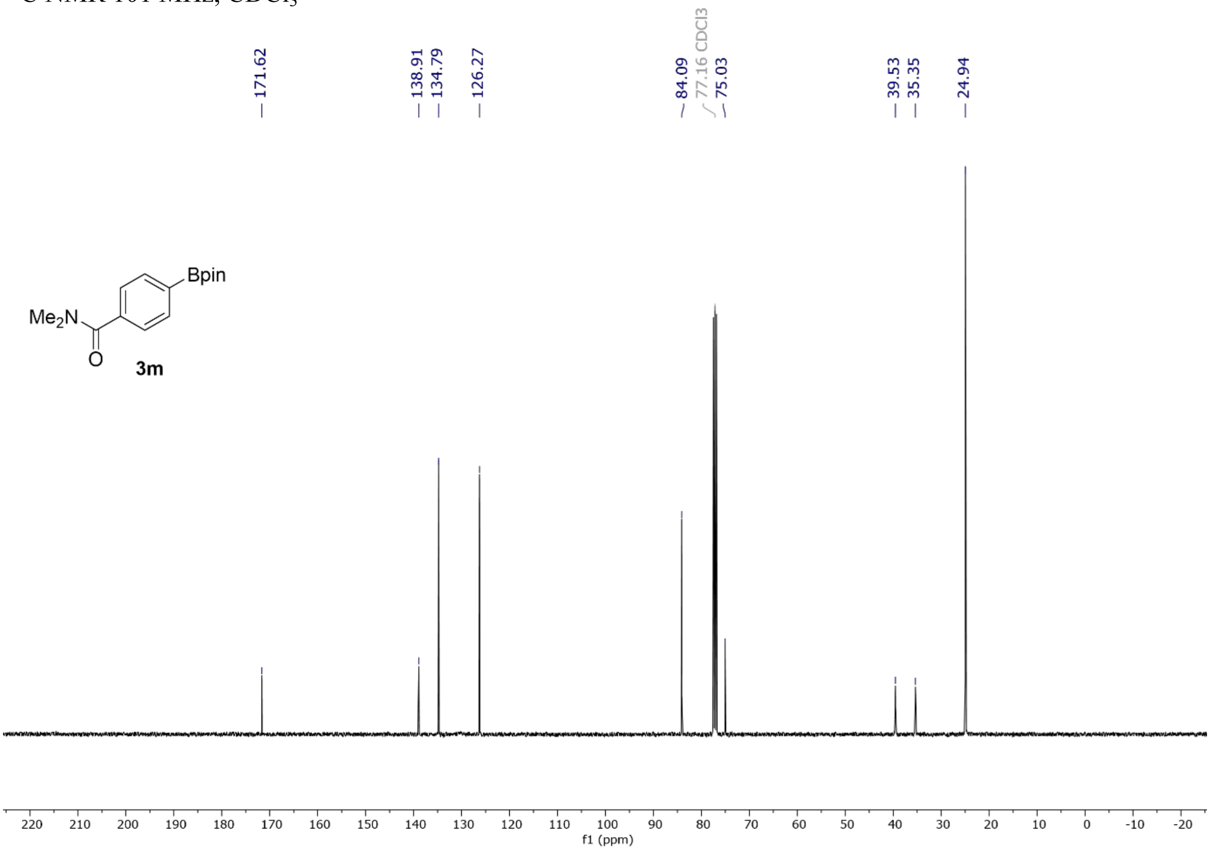

^{11}B NMR 128 MHz, $CDCl_3$

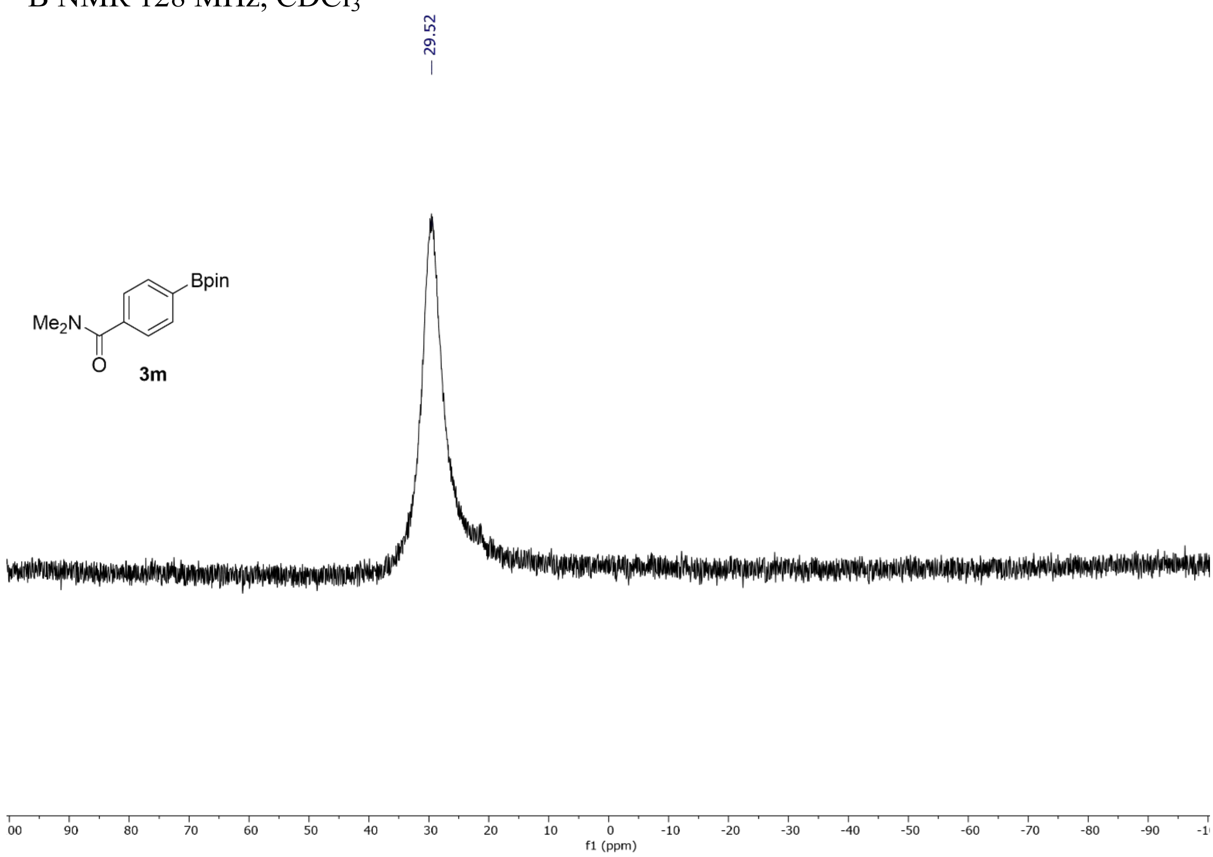

2-methyl-6-(4,4,5,5-tetramethyl-1,3,2-dioxaborolan-2-yl)quinoline (3o)

^{1}H NMR 400 MHz, $CDCl_3$

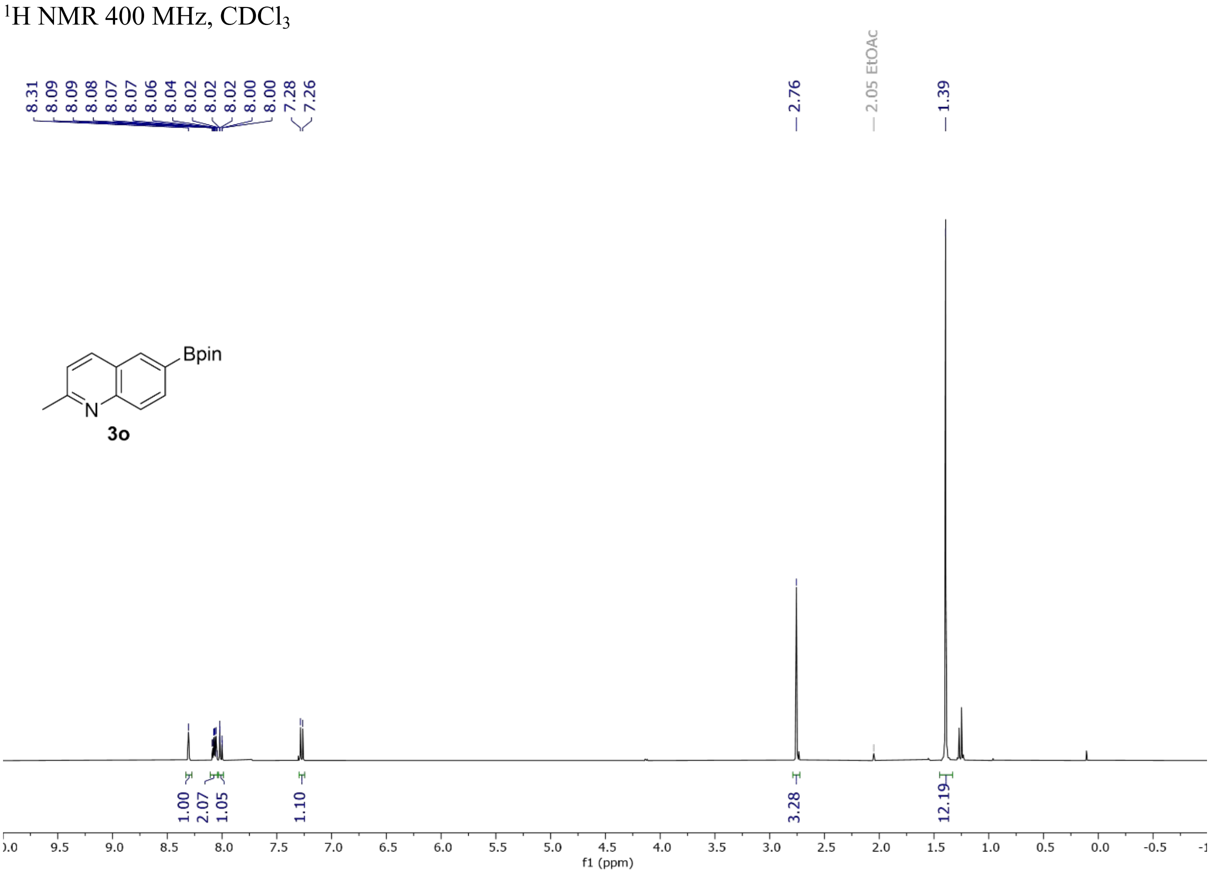

^{13}C NMR 101 MHz, $CDCl_3$

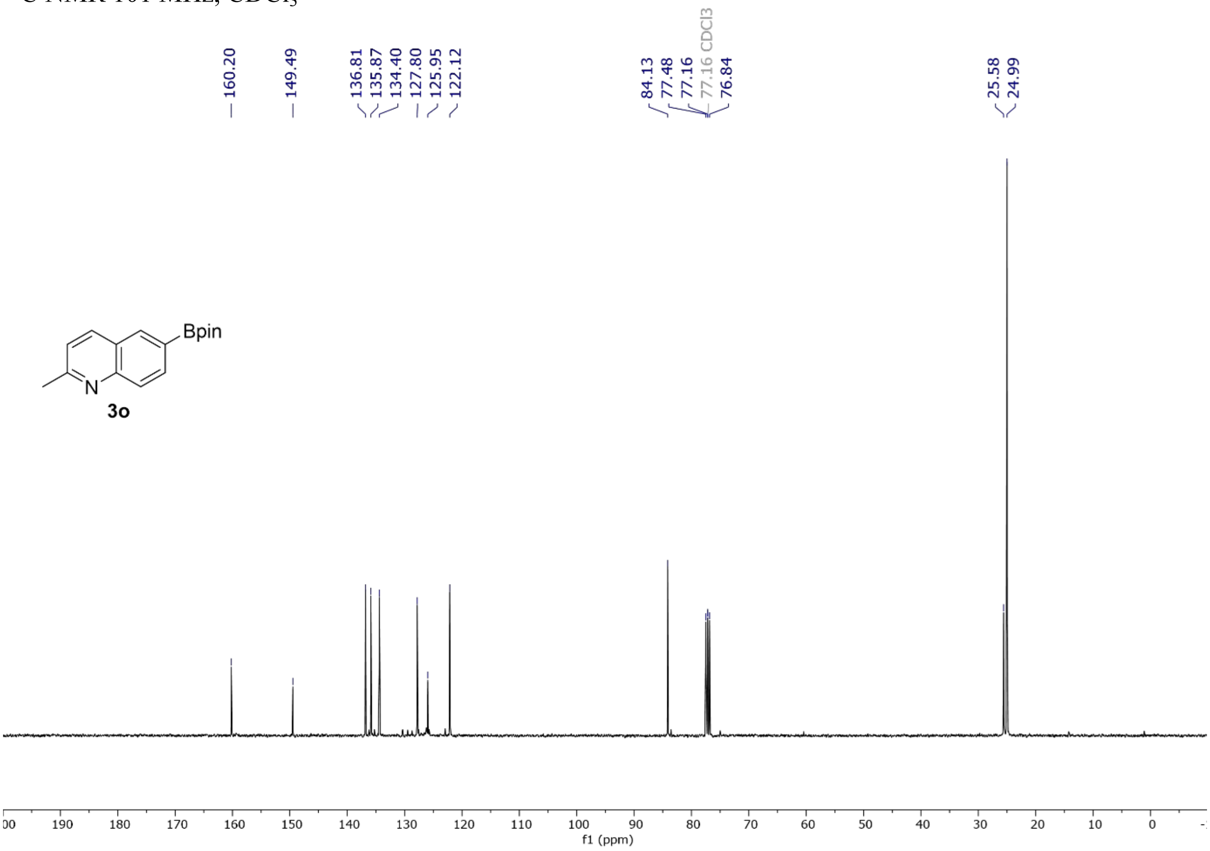

^{11}B NMR 128 MHz, $CDCl_3$

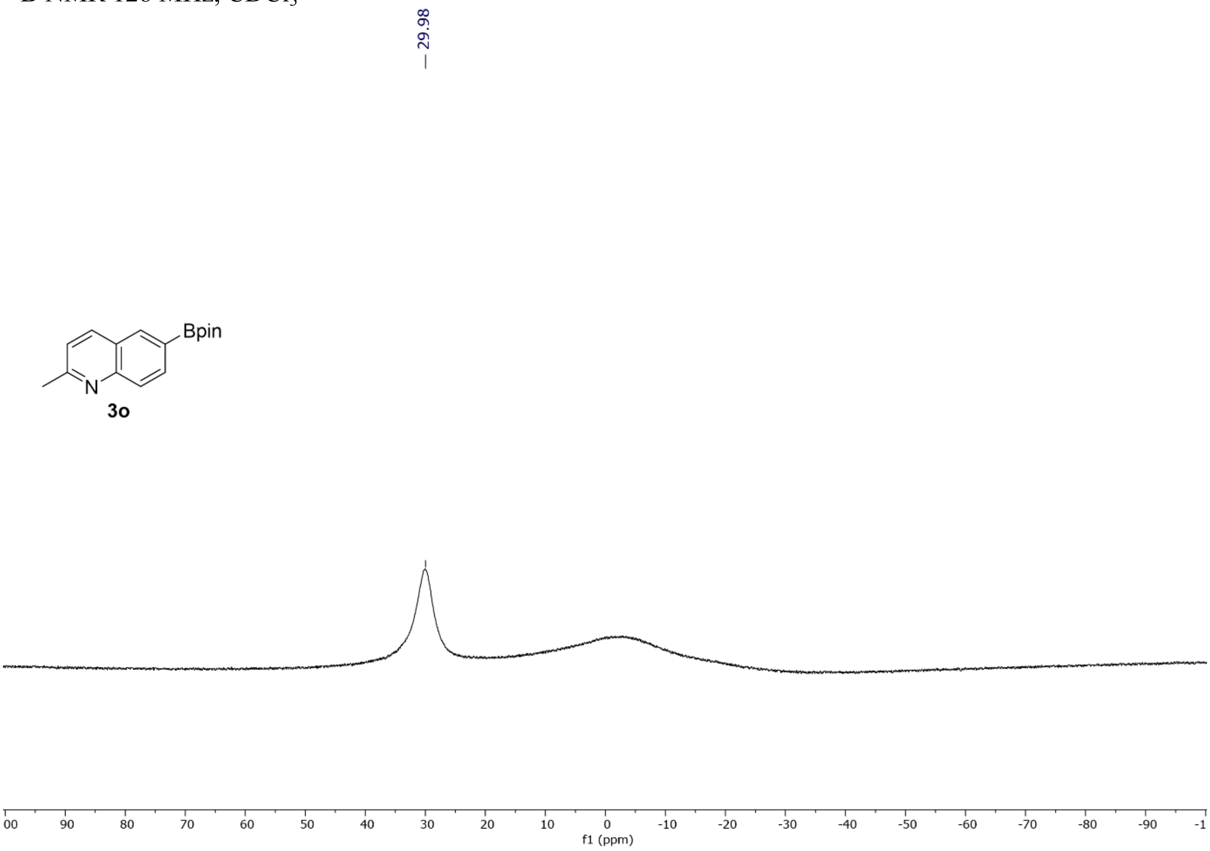

trimethyl(4-(4,4,5,5-tetramethyl-1,3,2-dioxaborolan-2-yl)phenyl)silane (3r)
^{1}H NMR 400 MHz, $CDCl_3$

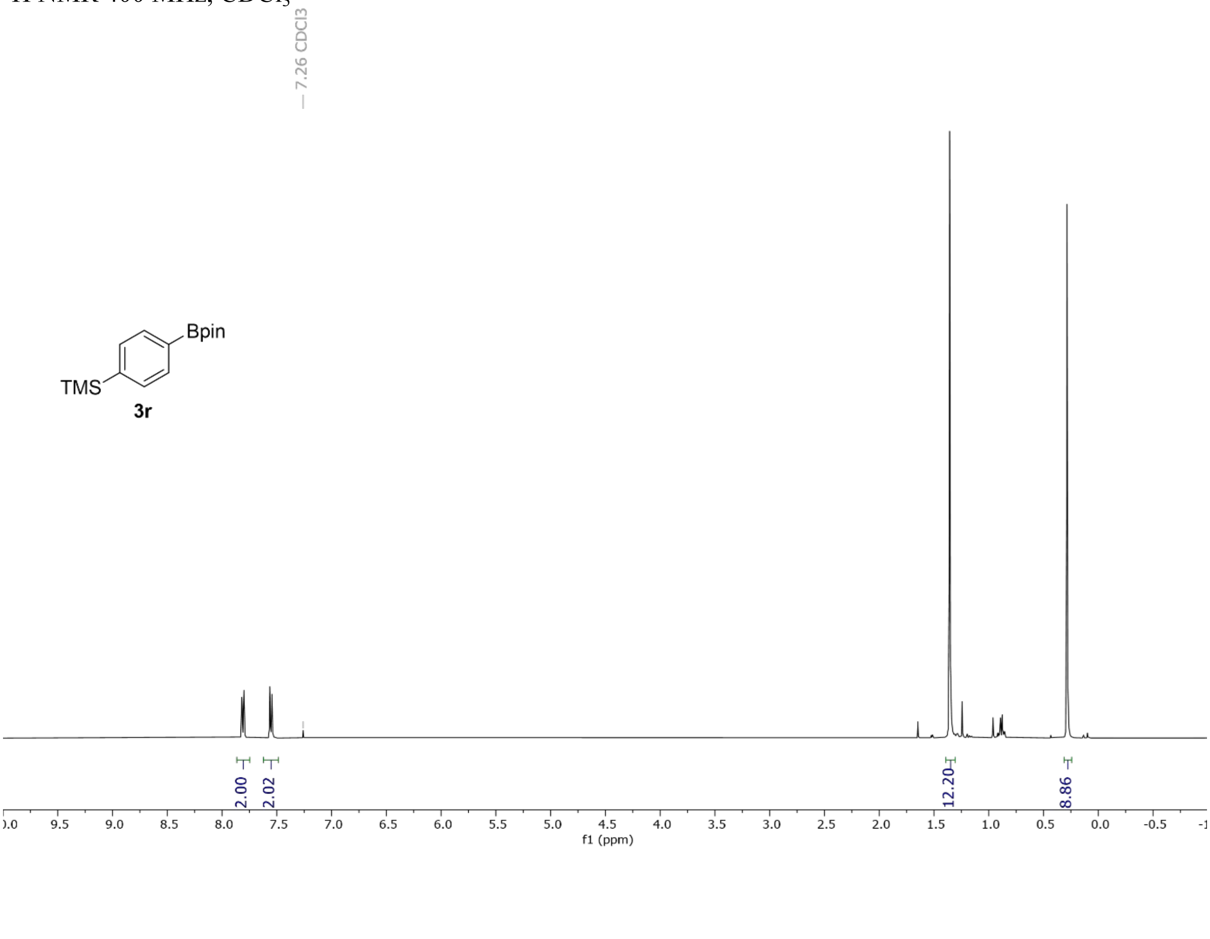

^{13}C NMR 101 MHz, $CDCl_3$

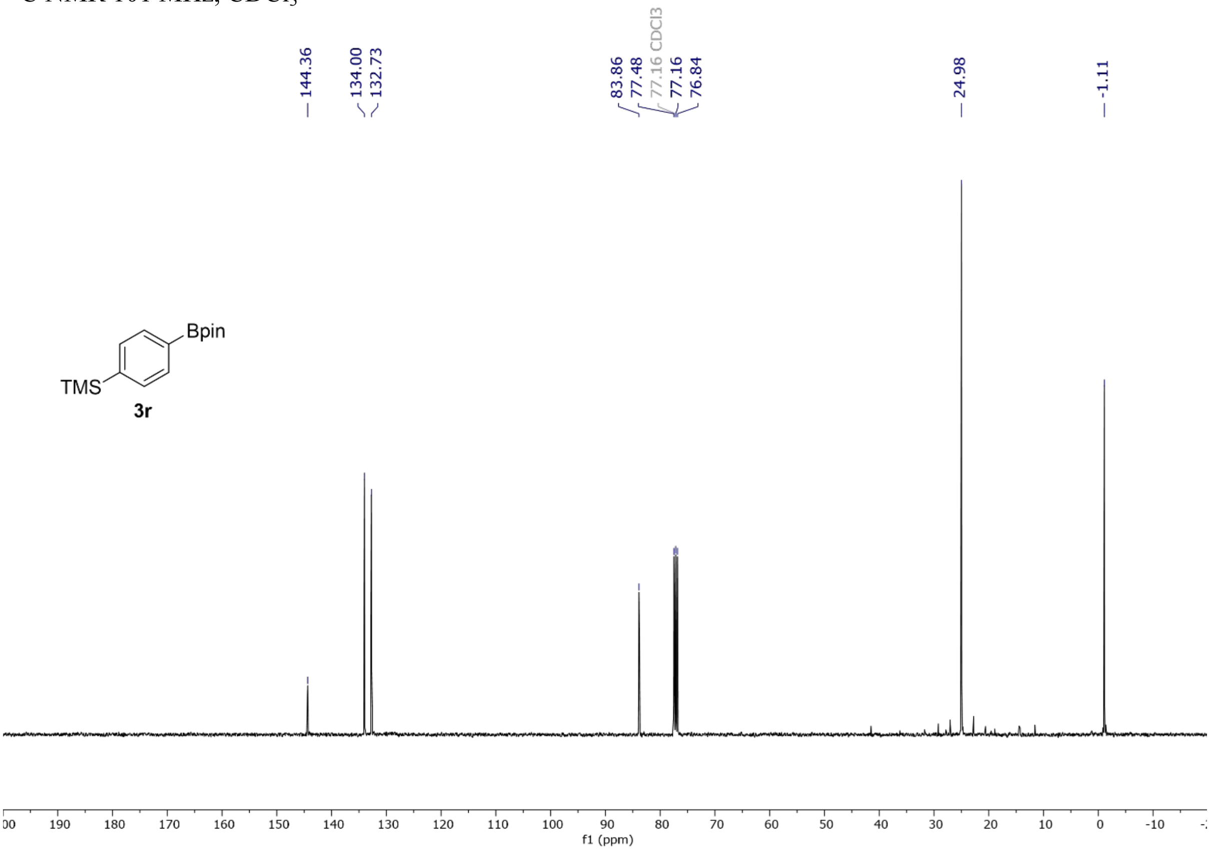

^{11}B NMR 128 MHz, $CDCl_3$

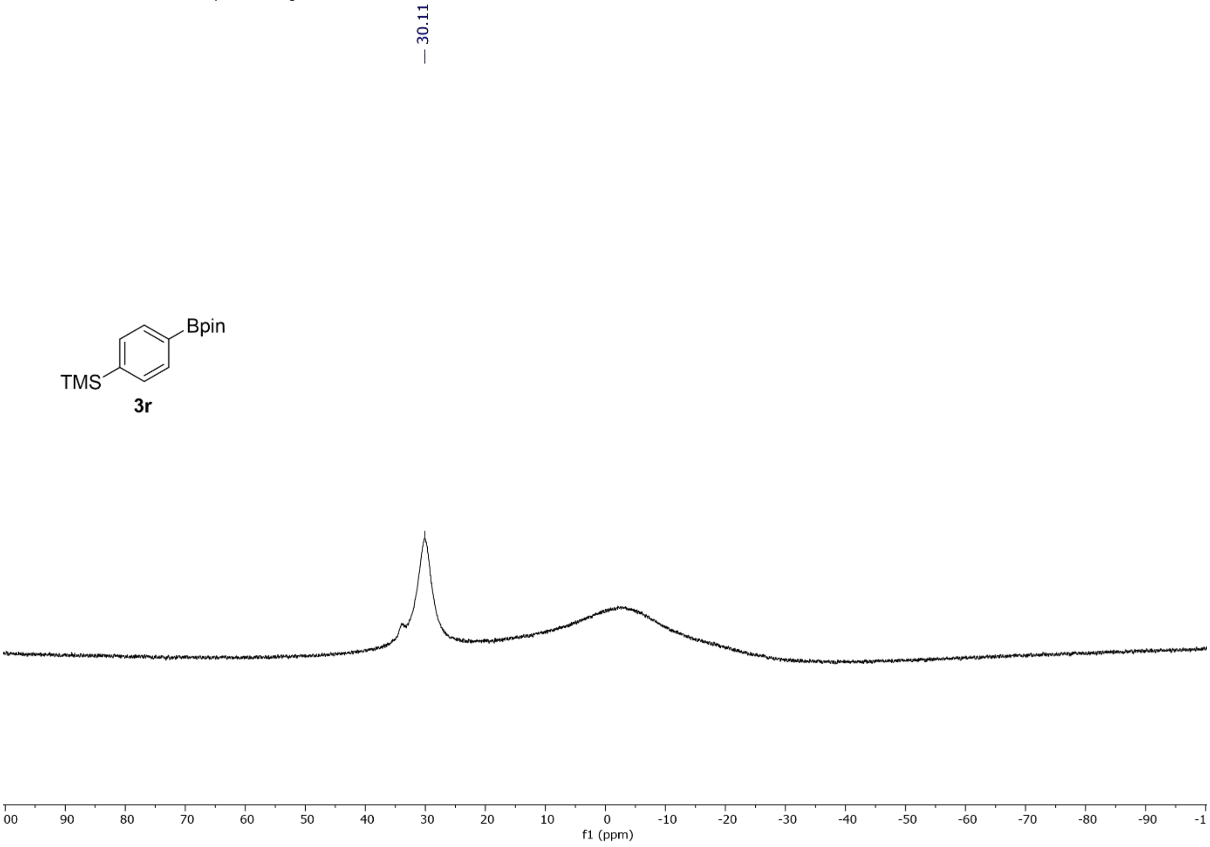

2-(benzo[d][1,3]dioxol-5-yl)-4,4,5,5-tetramethyl-1,3,2-dioxaborolane (3s)

^{1}H NMR 400 MHz, $CDCl_3$

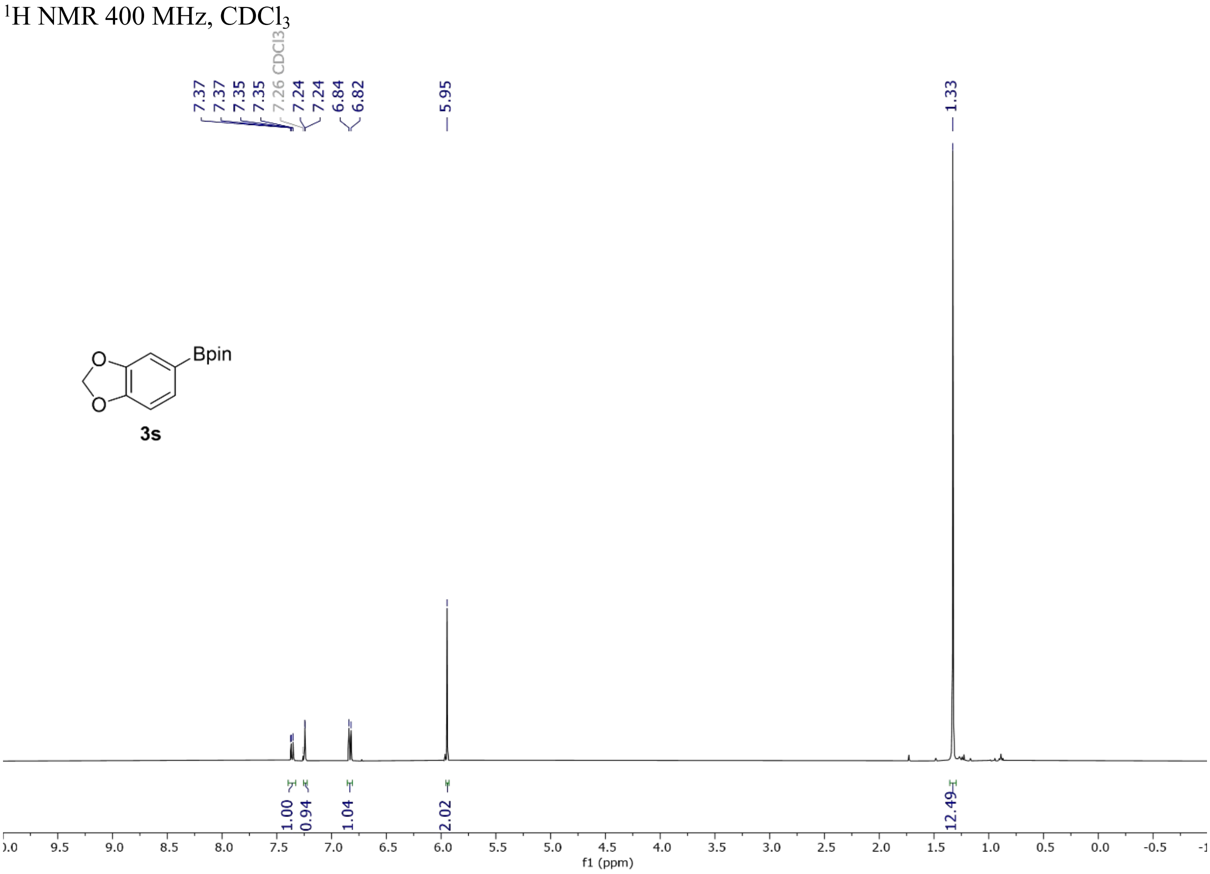

^{13}C NMR 101 MHz, $CDCl_3$

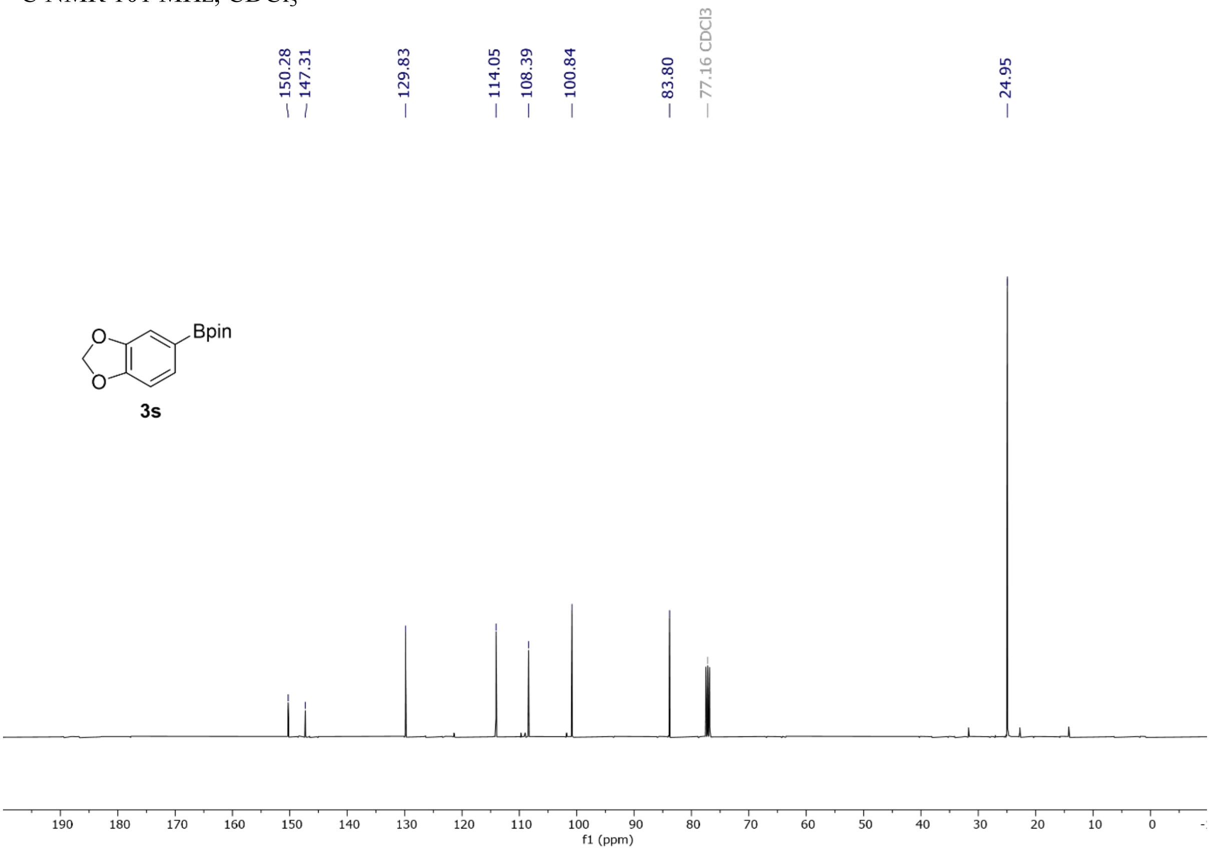

^{11}B NMR 128 MHz, $CDCl_3$

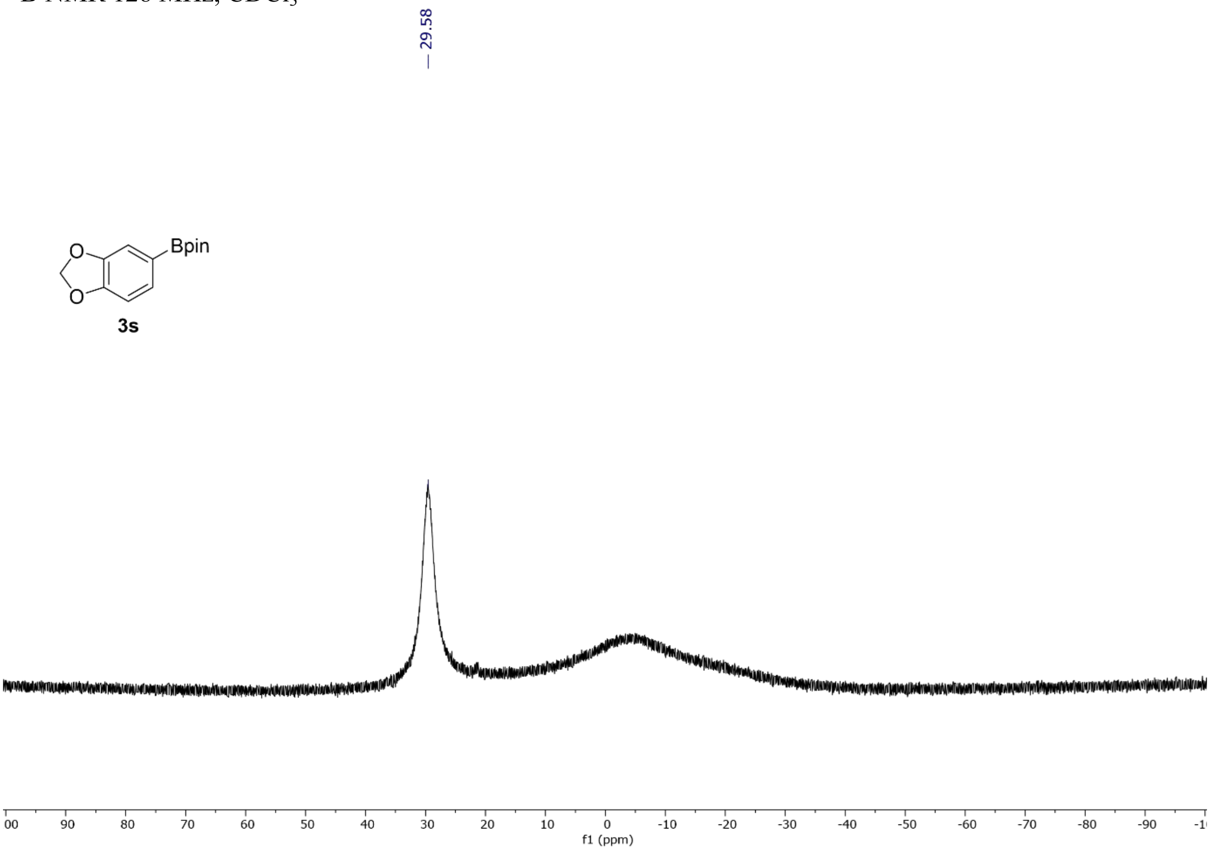

1,4-bis(4,4,5,5-tetramethyl-1,3,2-dioxaborolan-2-yl)benzene (3t)
^{1}H NMR 400 MHz, $CDCl_3$

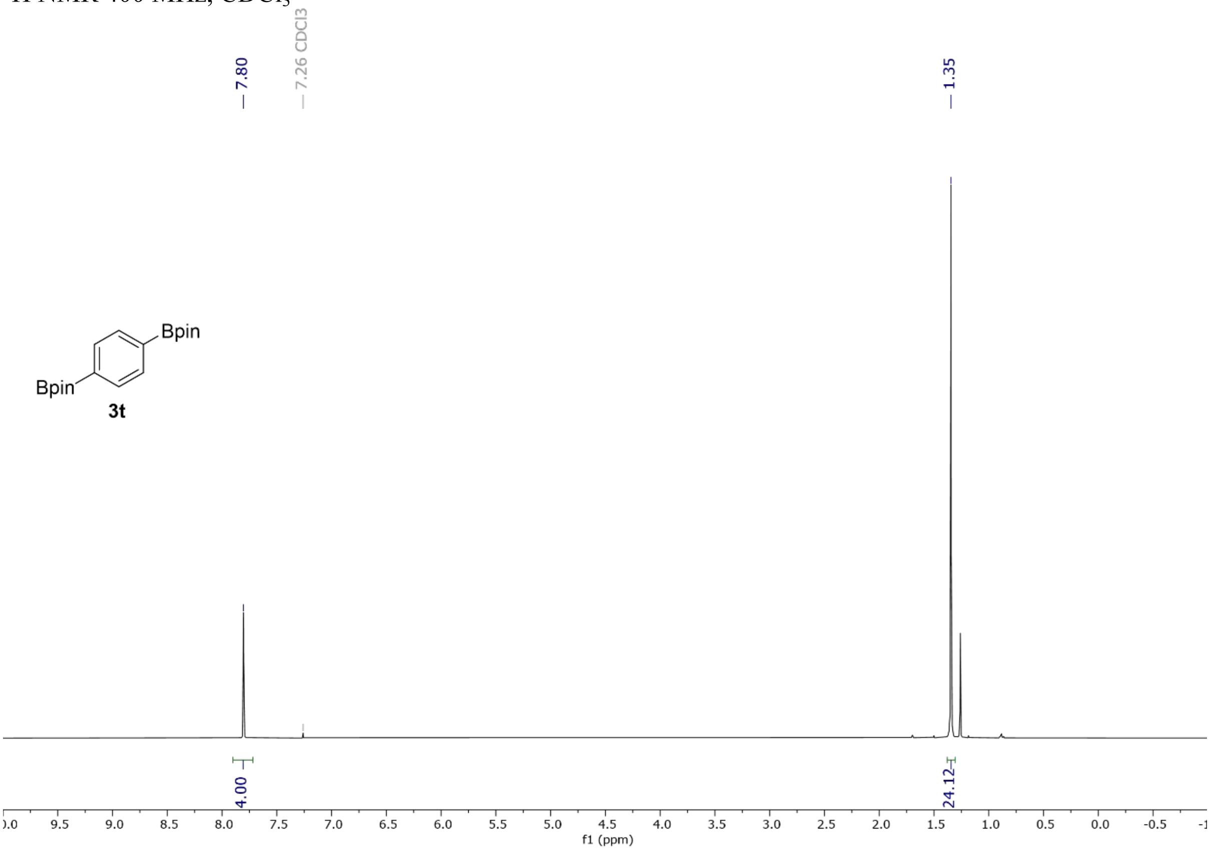

^{13}C NMR 101 MHz, $CDCl_3$

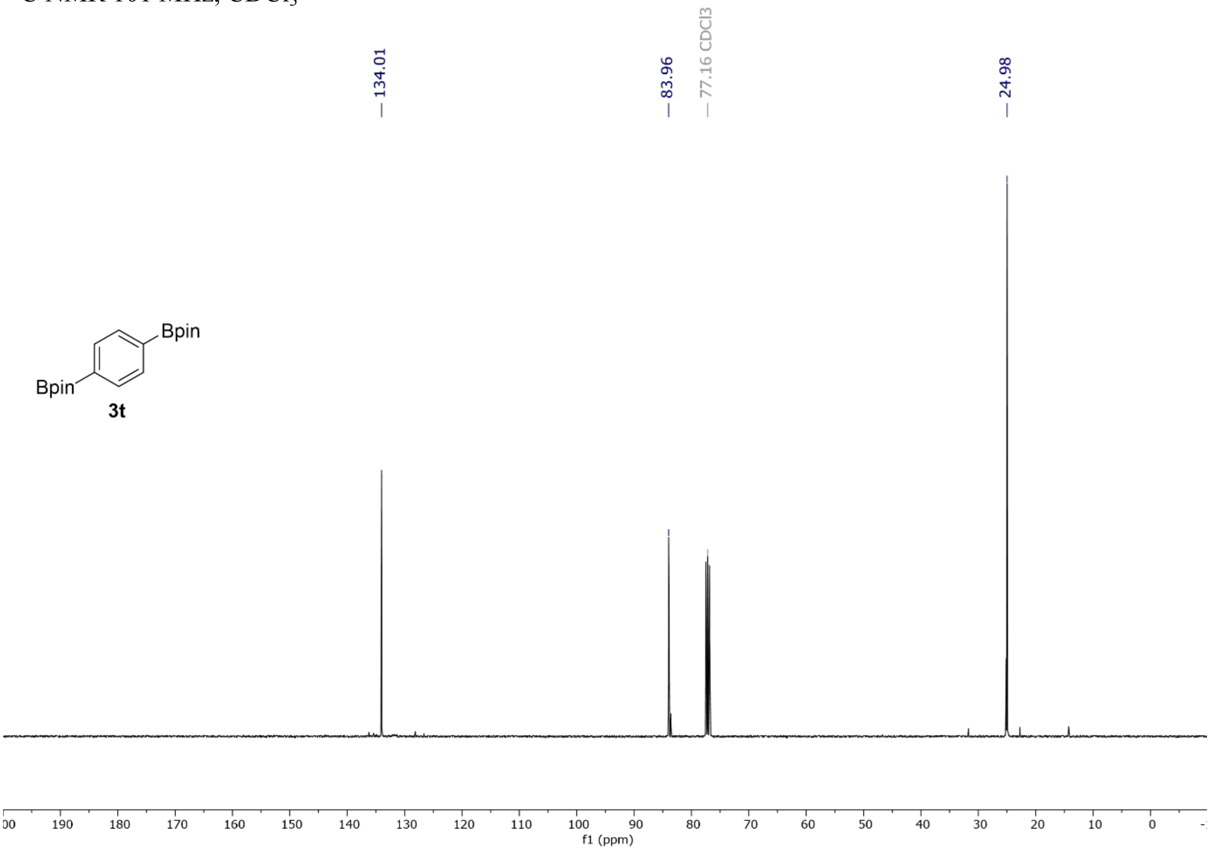

^{11}B NMR 128 MHz, $CDCl_3$

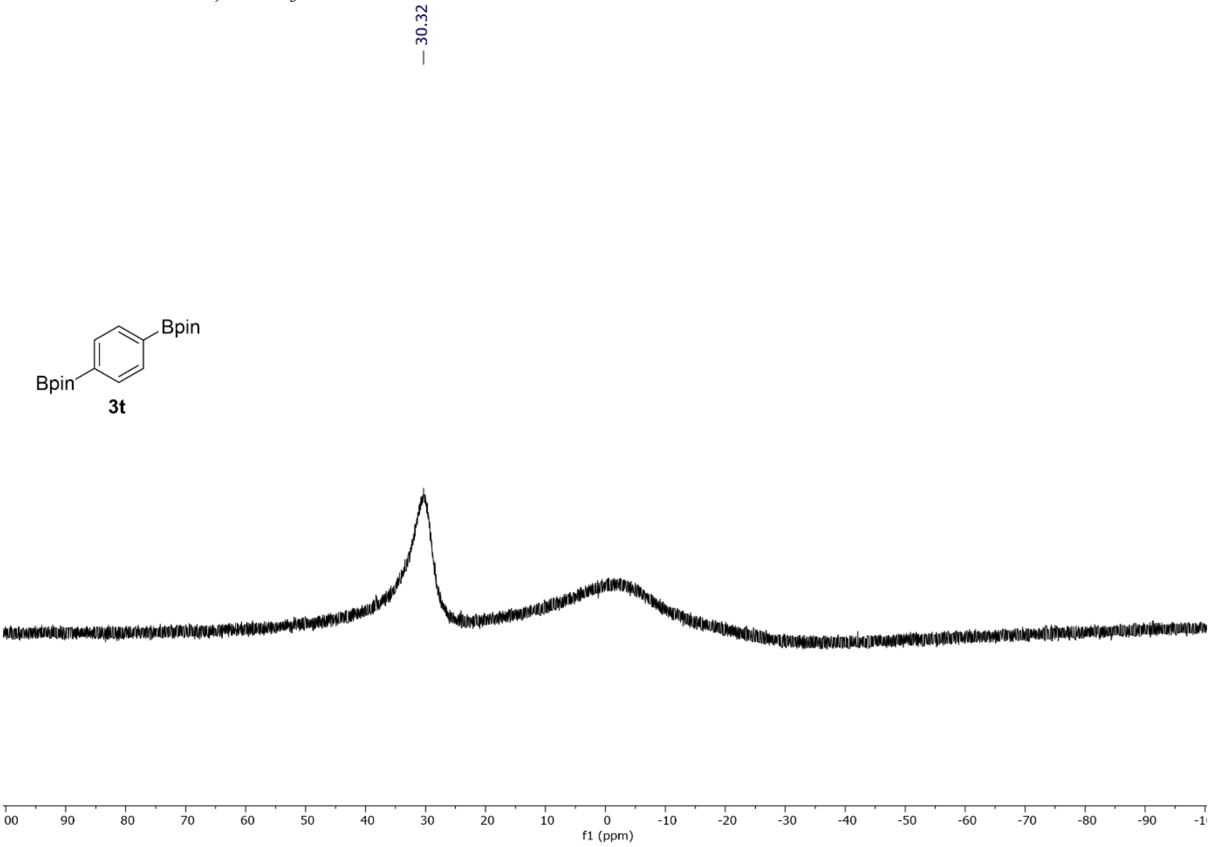

2-([1,1'-biphenyl]-4-yl)-4,4,5,5-tetramethyl-1,3,2-dioxaborolane (3u)

^{1}H NMR 400 MHz, $CDCl_3$

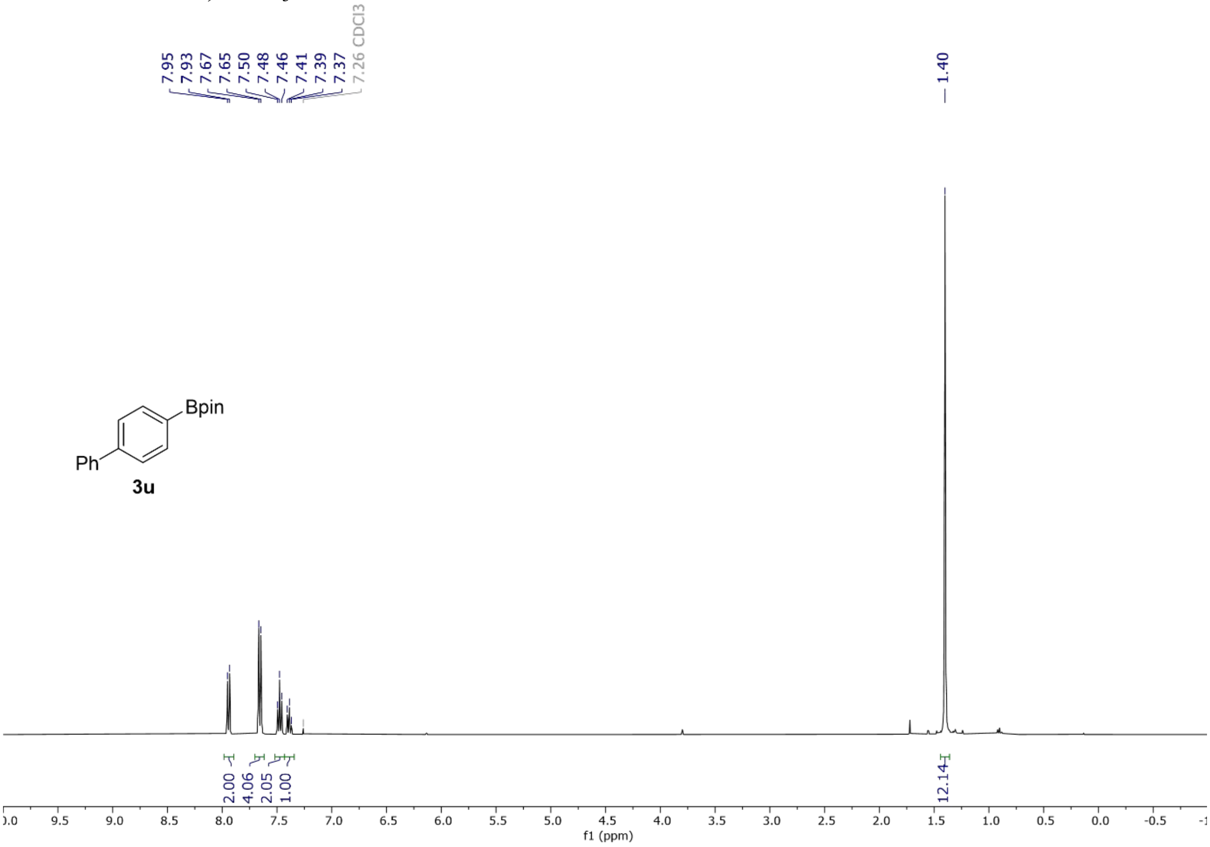

^{13}C NMR 101 MHz, $CDCl_3$

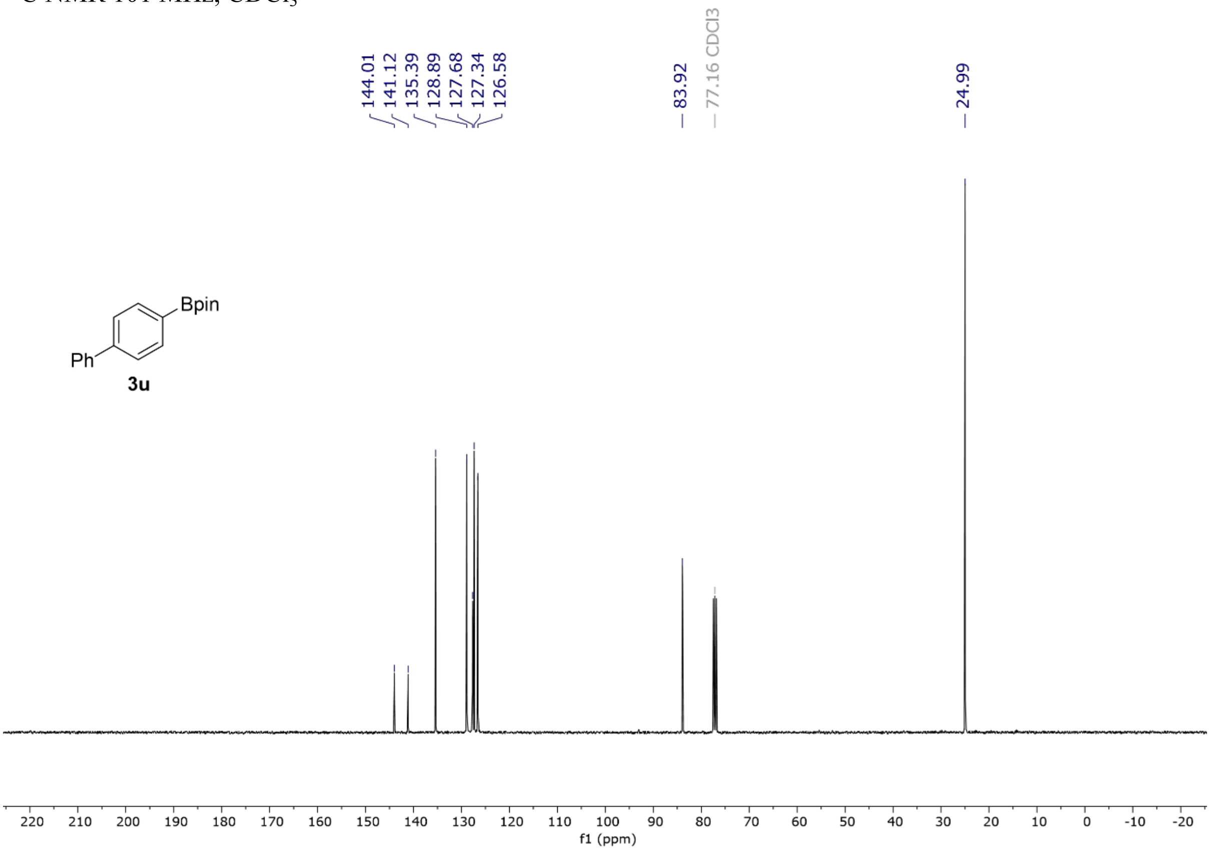

^{11}B NMR 128 MHz, $CDCl_3$

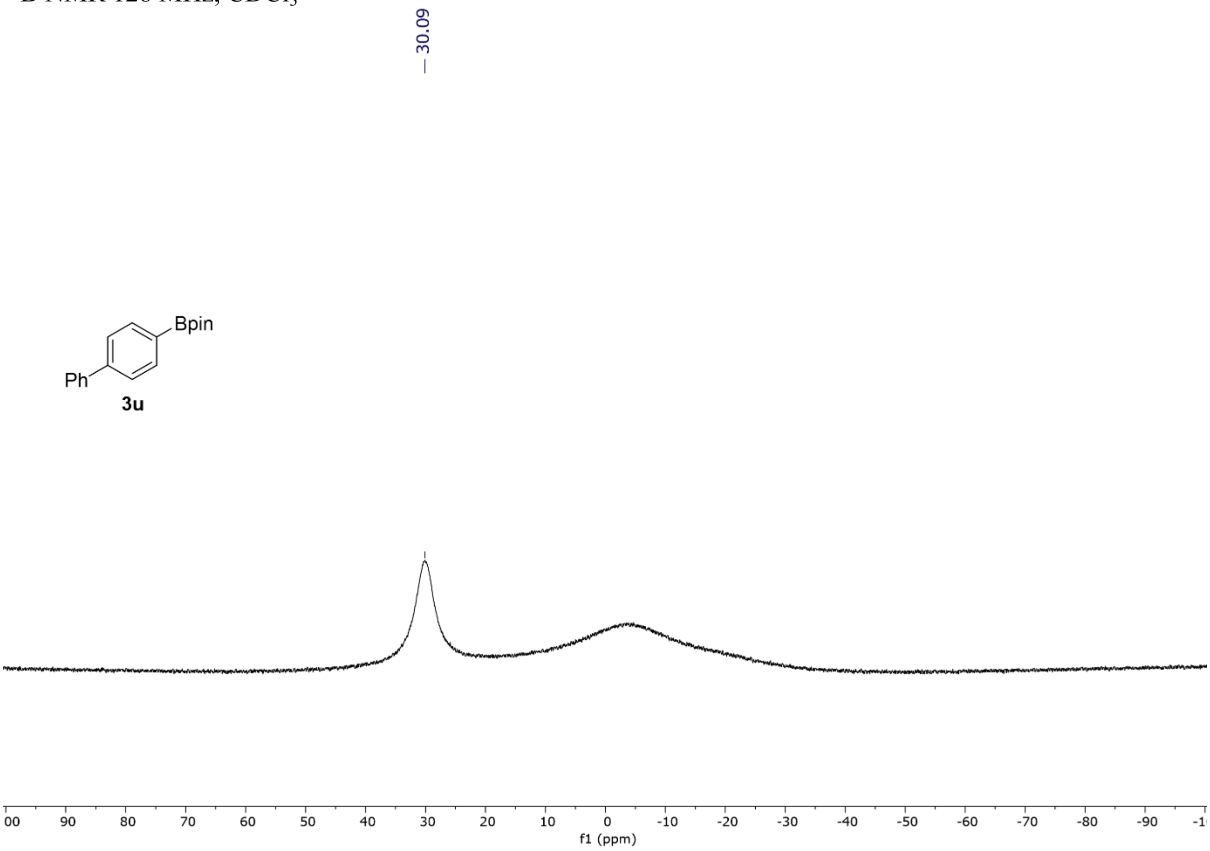

NMR spectra of 3a from 1mmol scale reaction

^{1}H NMR 400 MHz, $CDCl_3$

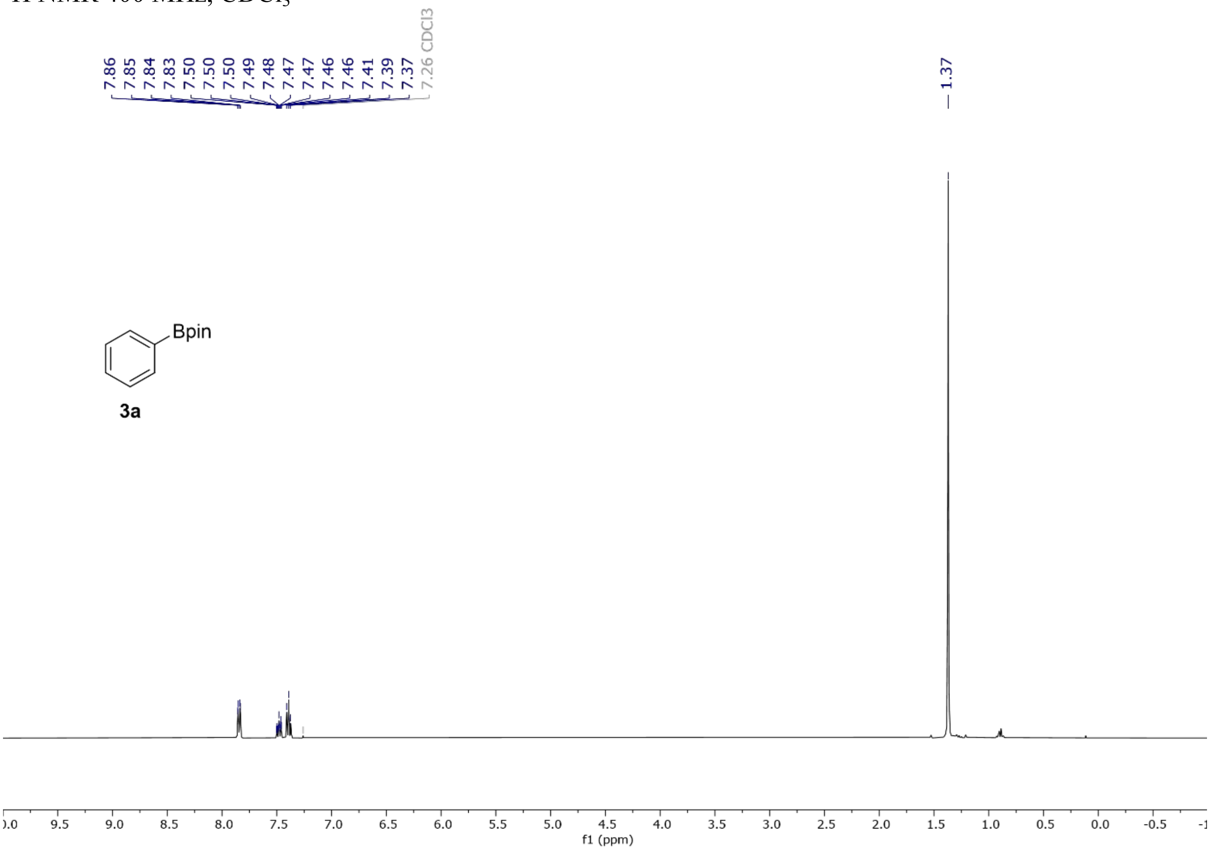

^{13}C NMR 101 MHz, $CDCl_3$

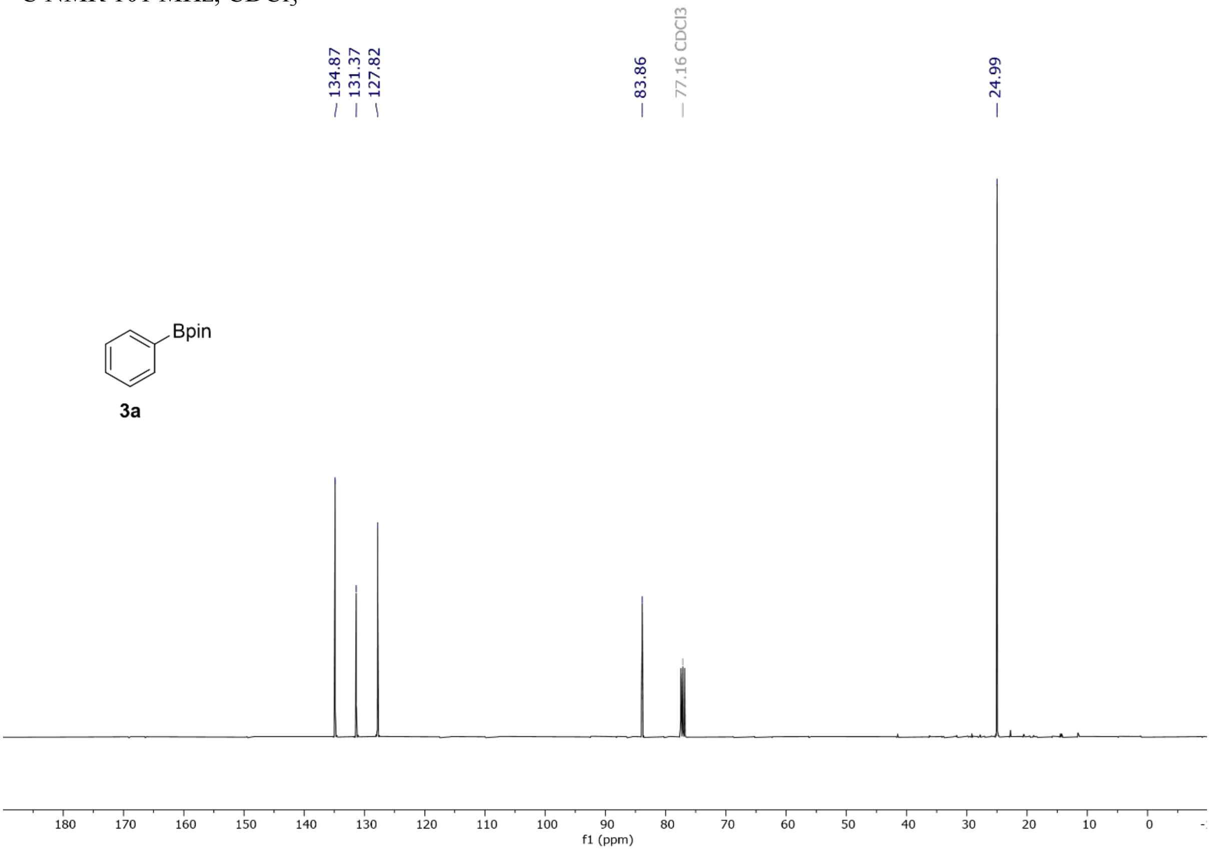

^{11}B NMR 128 MHz, $CDCl_3$

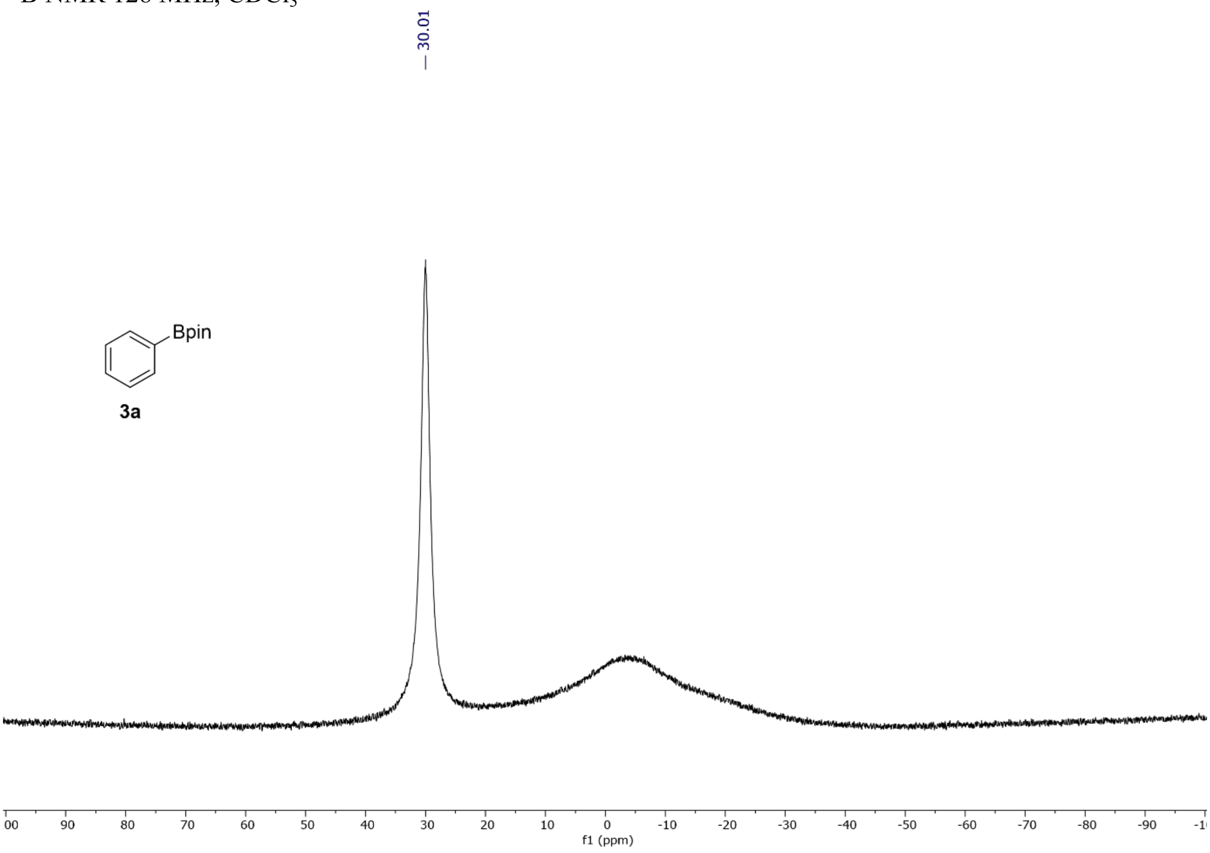

4-bromophenyl trifluoromethanesulfonate (see Figure S2)

^{1}H NMR 400 MHz, $CDCl_3$

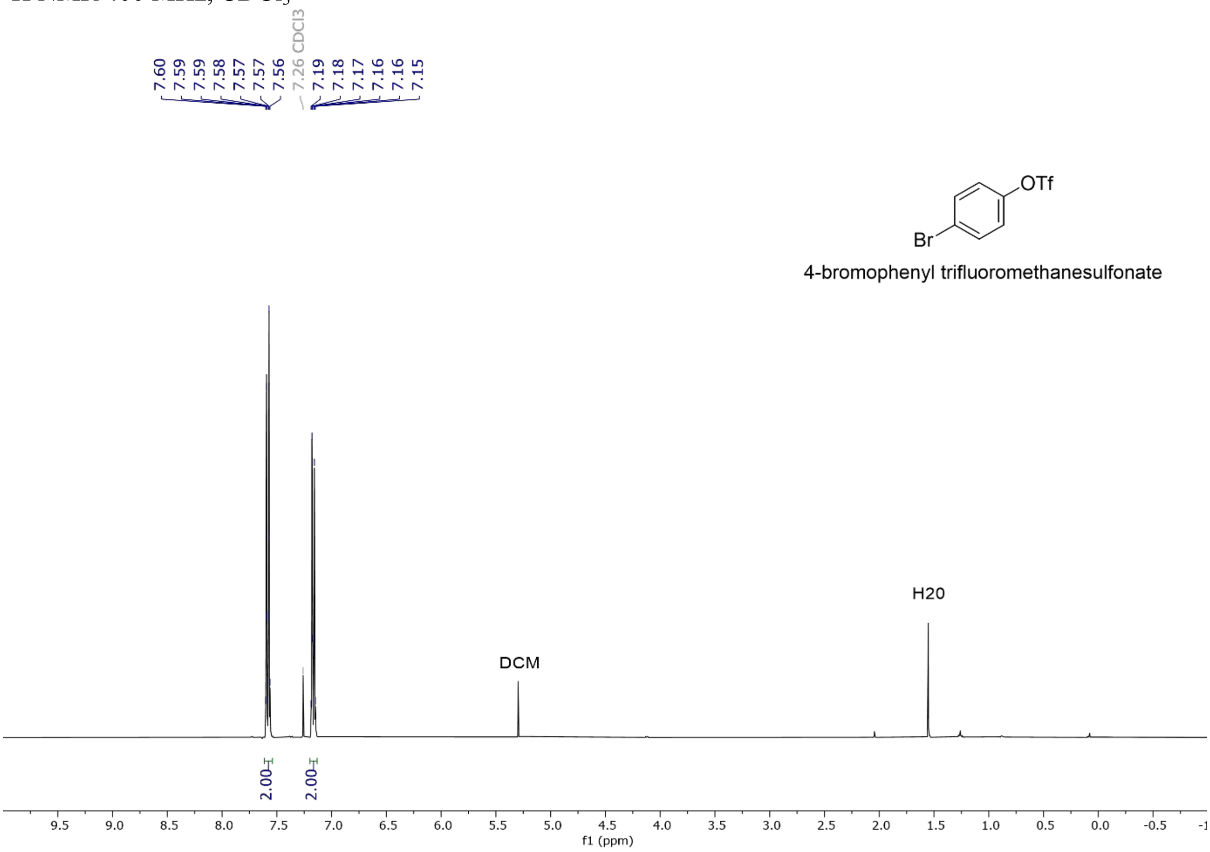

^{13}C NMR 101 MHz, $CDCl_3$

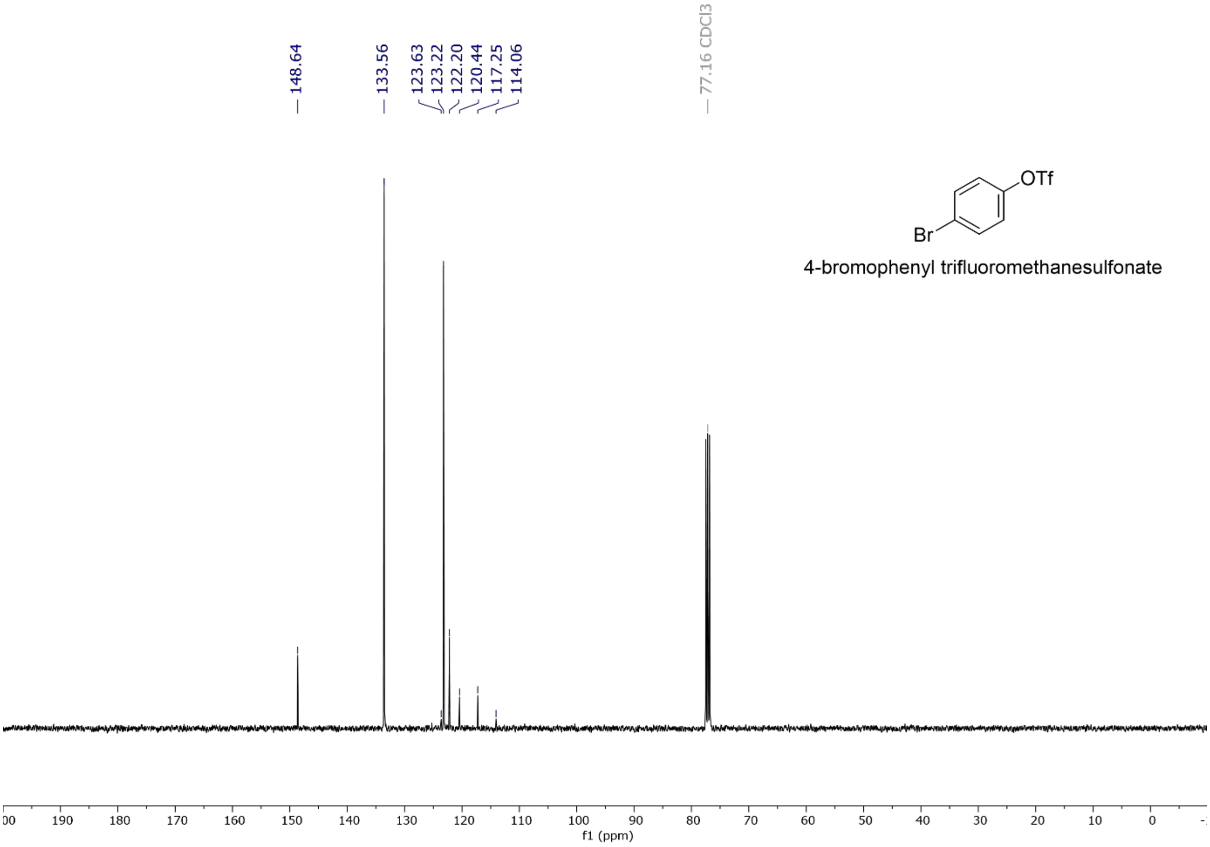

^{19}F NMR 377 MHz $CDCl_3$

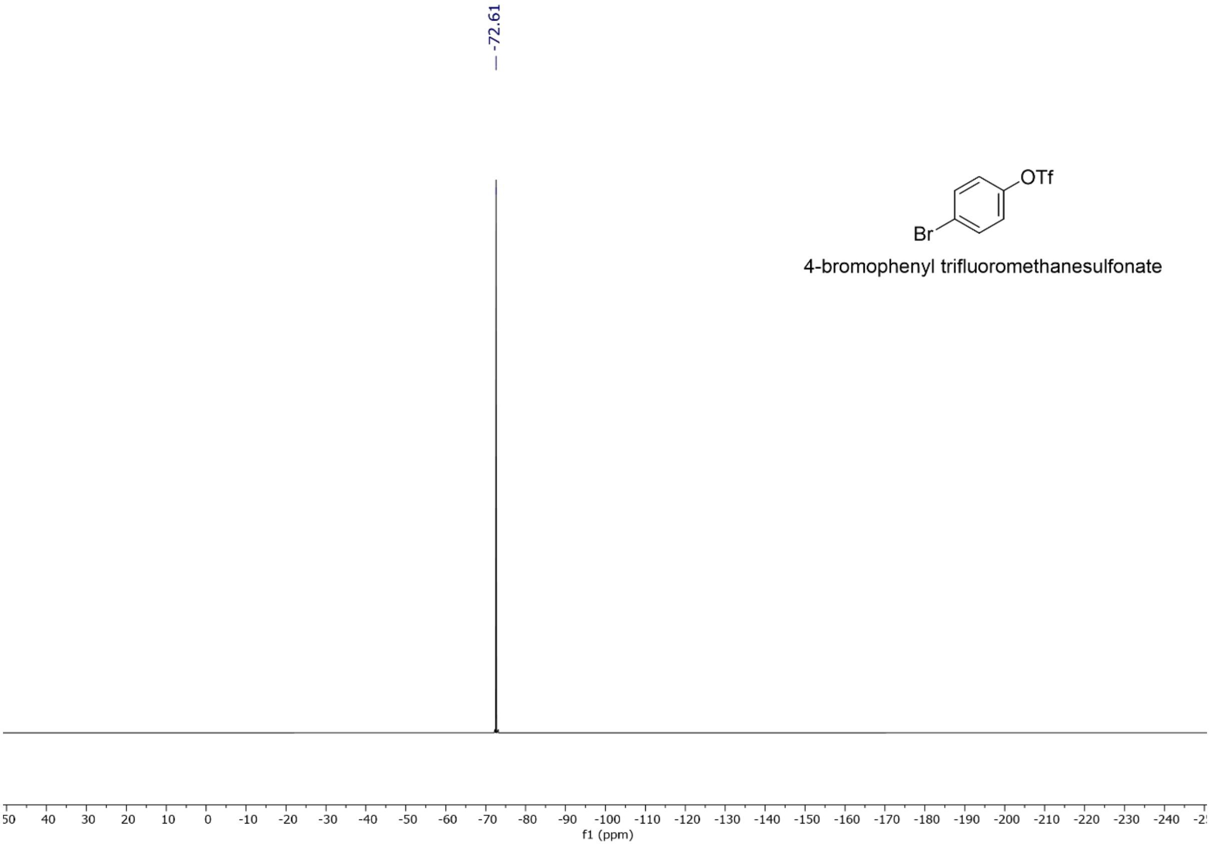

4-(4-bromophenoxy)phenyl trifluoromethanesulfonate (see Figure S2)

^{1}H NMR 400 MHz, $CDCl_3$

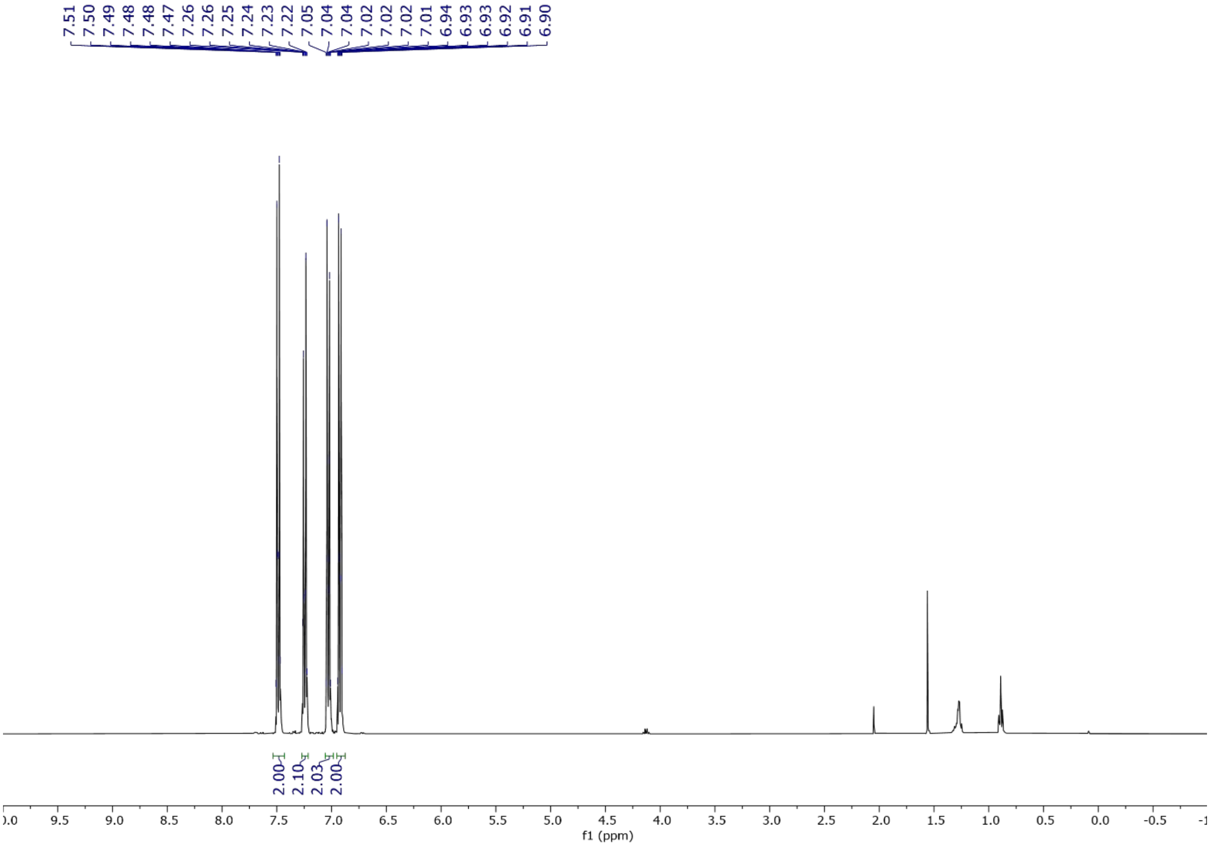

^{13}C NMR 101 MHz, $CDCl_3$

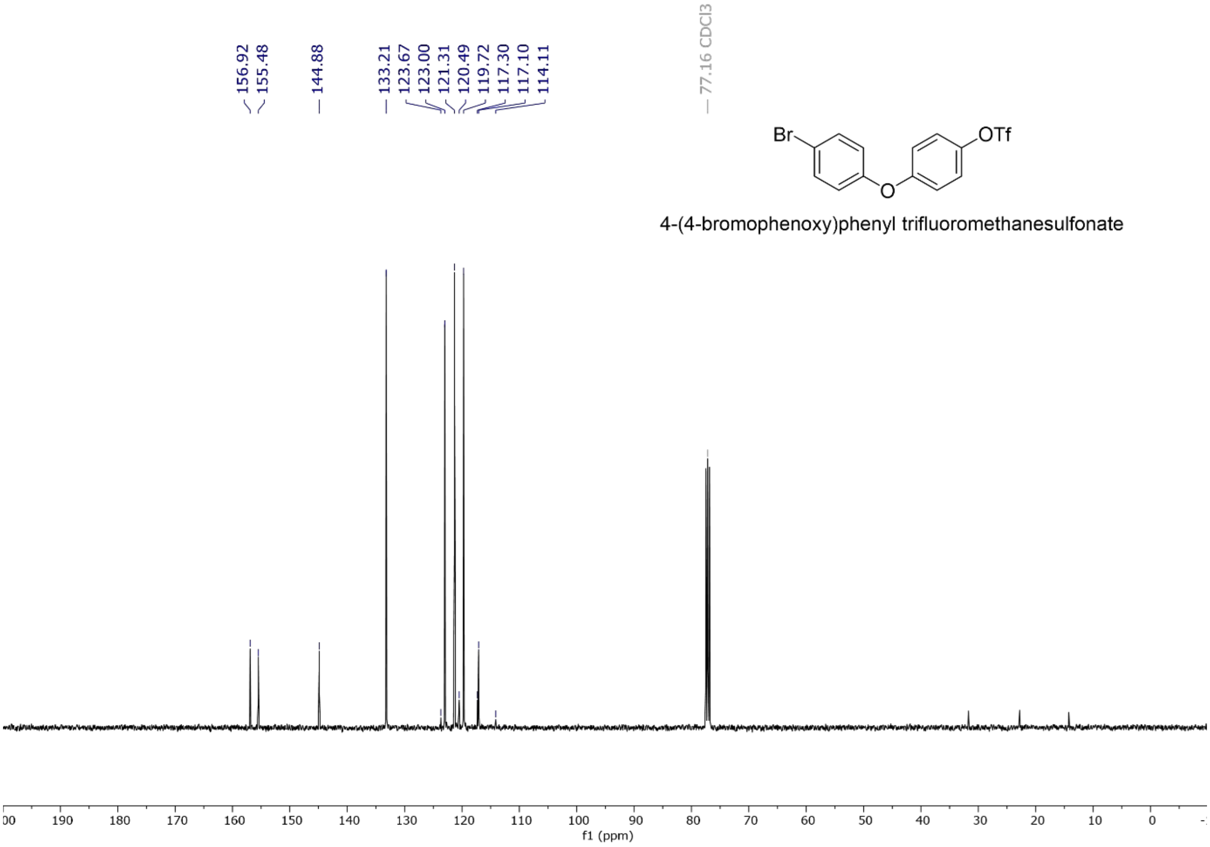

^{19}F NMR 377 MHz $CDCl_3$

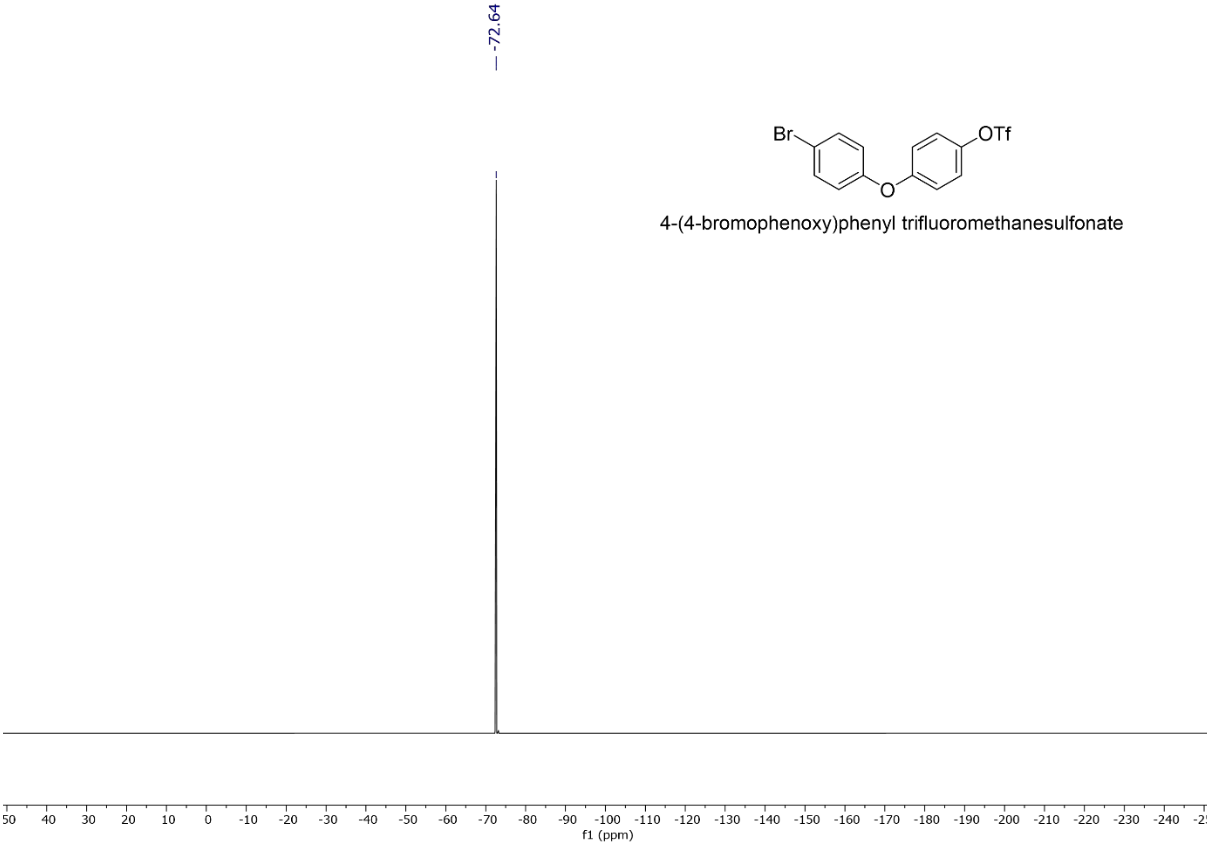

quinolin-7-yl trifluoromethanesulfonate (see Figure S2)

^{1}H NMR 400 MHz, $CDCl_3$

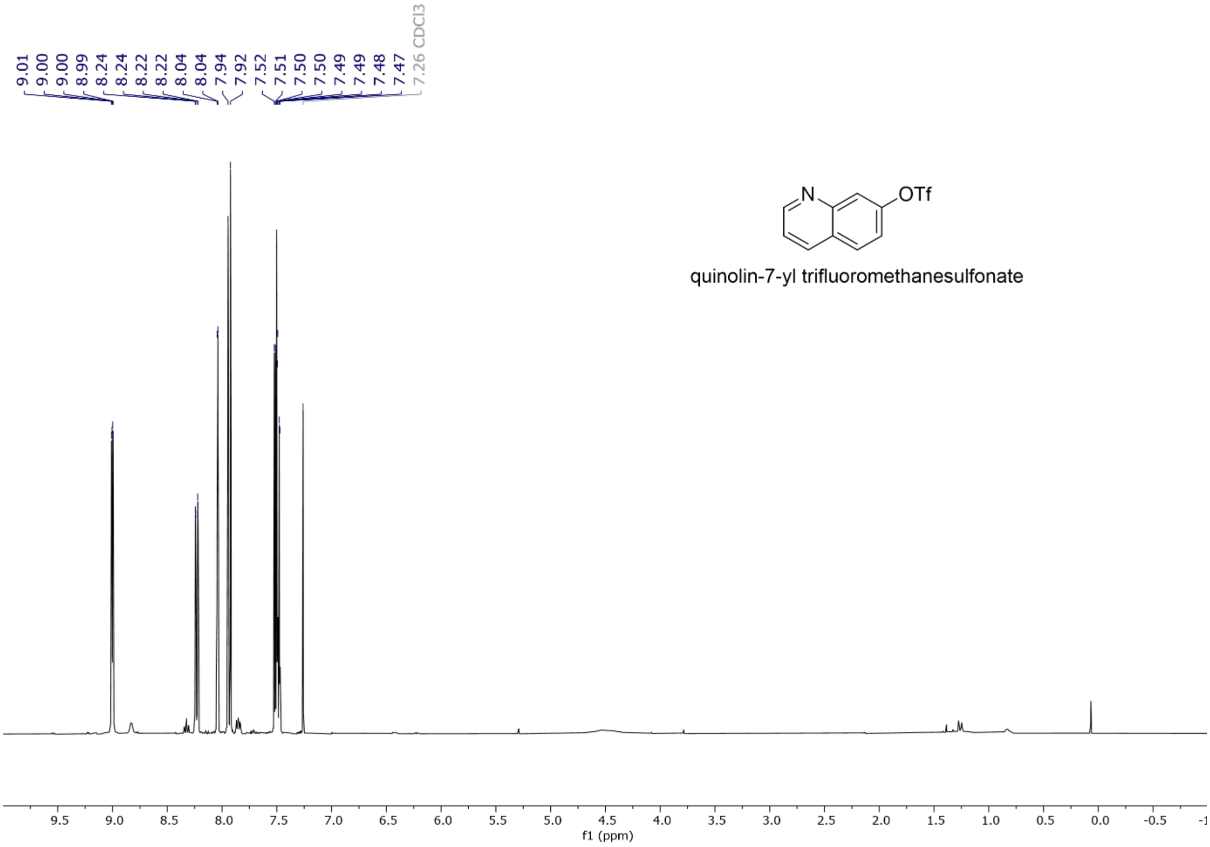

^{13}C NMR 101 MHz, $CDCl_3$

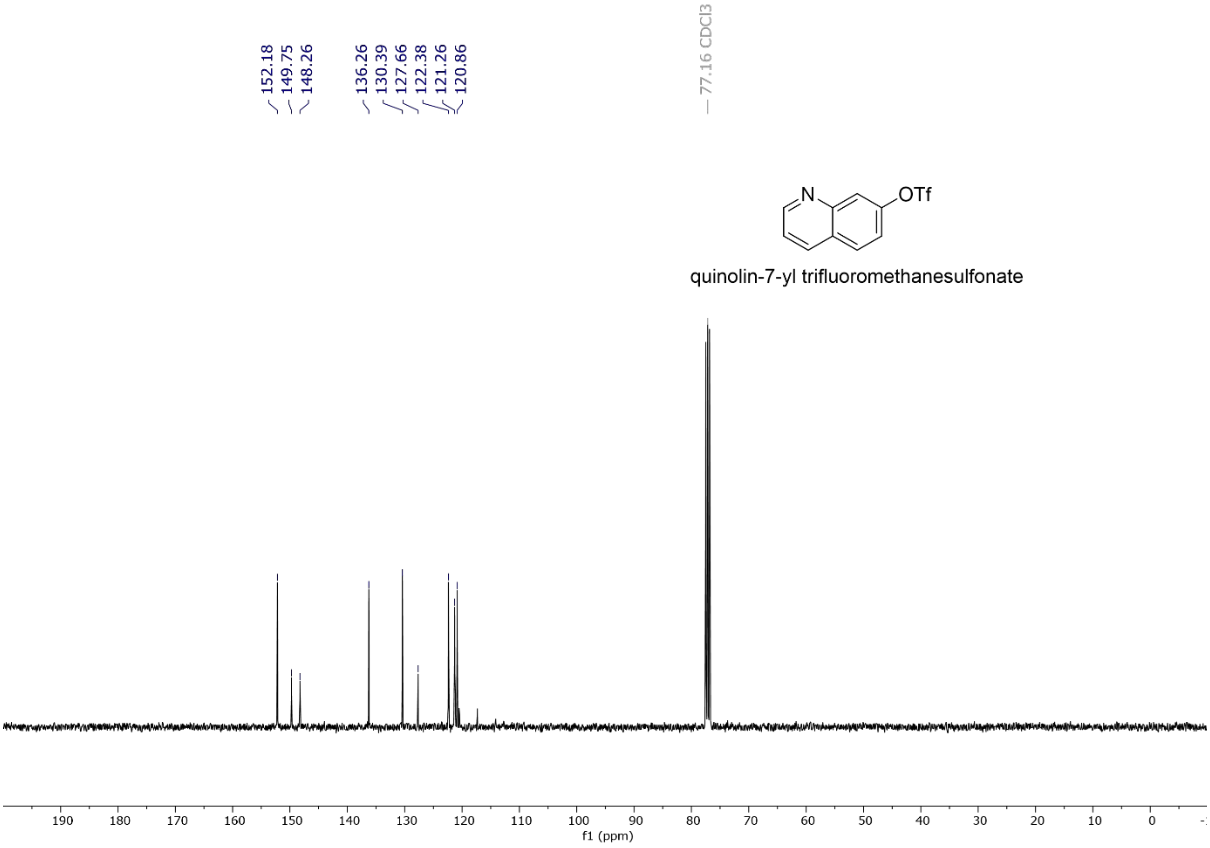

^{19}F NMR 377 MHz $CDCl_3$

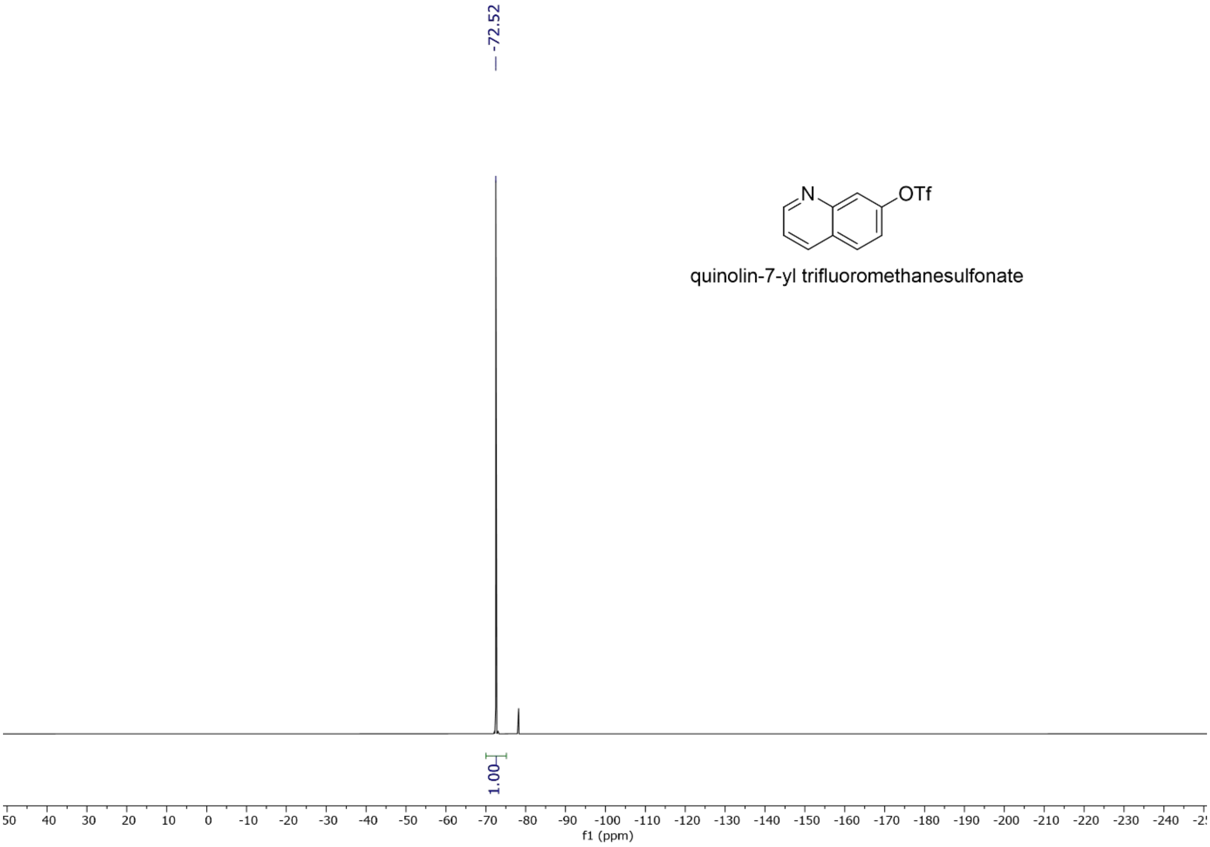

1-bromonaphthalen-2-yl trifluoromethanesulfonate (see Figure S2)

^{1}H NMR 400 MHz, $CDCl_3$

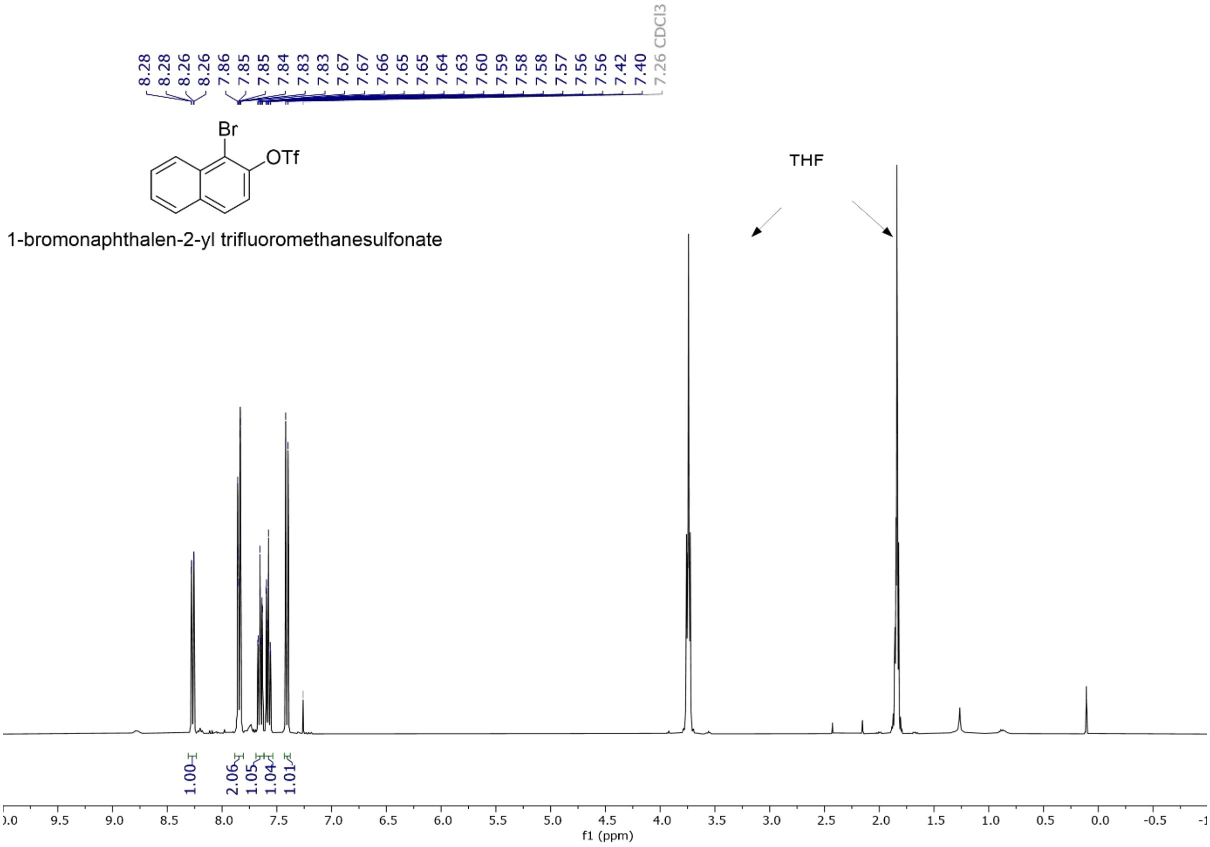

^{13}C NMR 101 MHz, $CDCl_3$

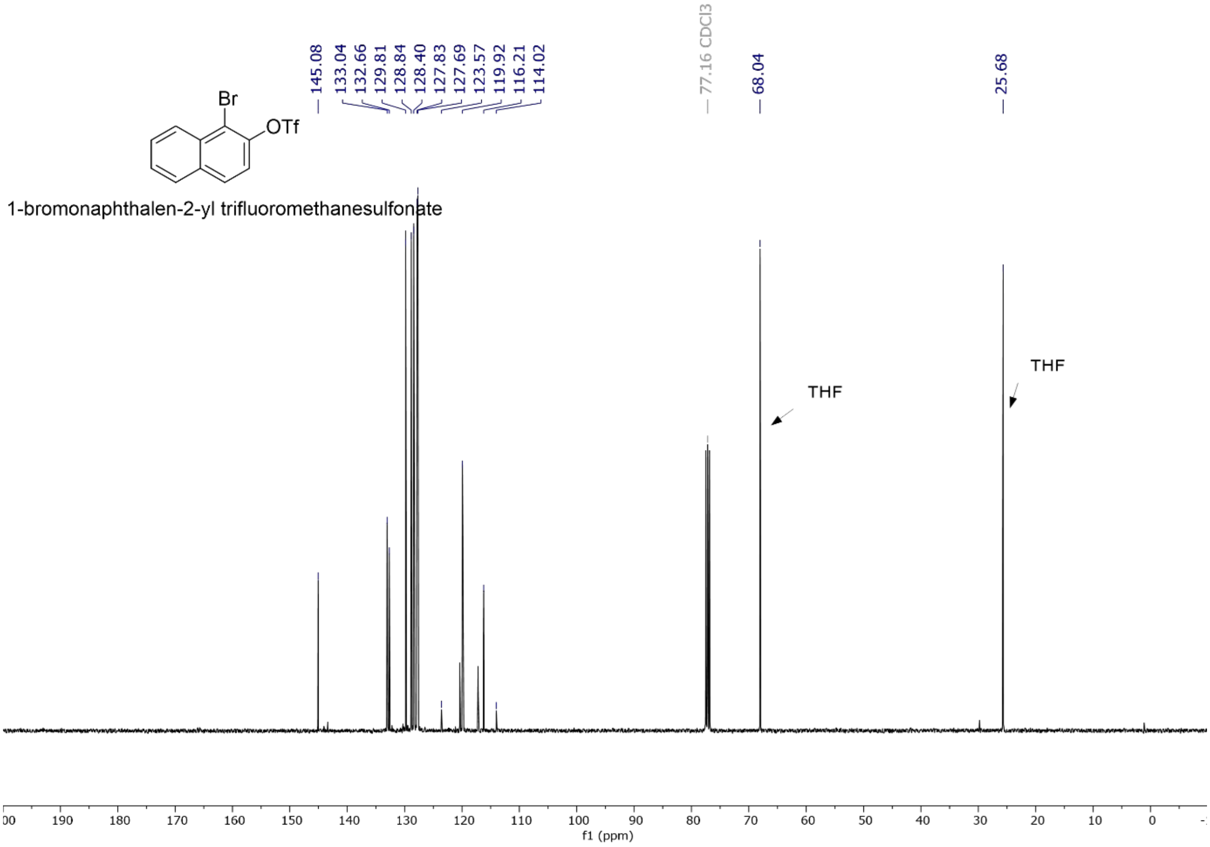

^{19}F NMR 377 MHz $CDCl_3$

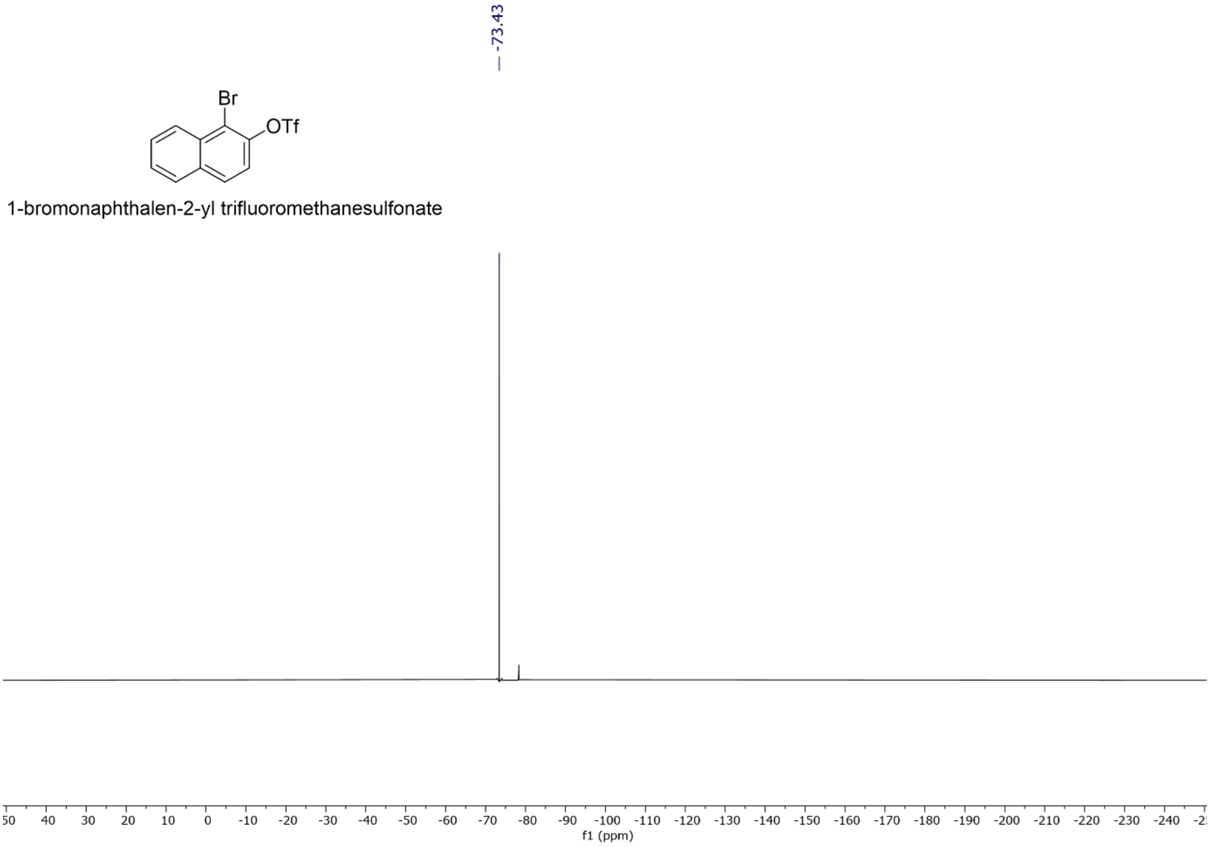

4-carbomethoxyphenyl trifluoromethanesulfonate

^{1}H NMR 400 MHz, $CDCl_3$

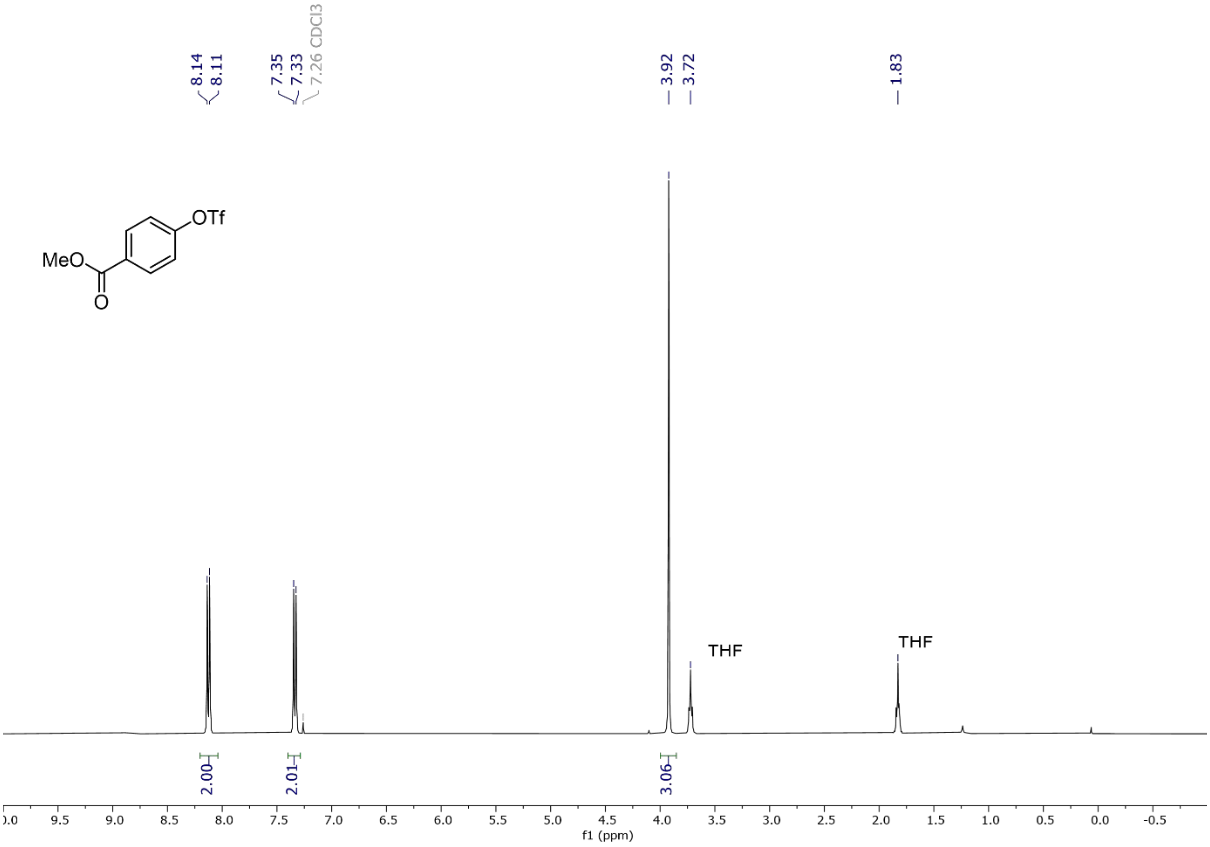

^{13}C NMR 101 MHz, $CDCl_3$

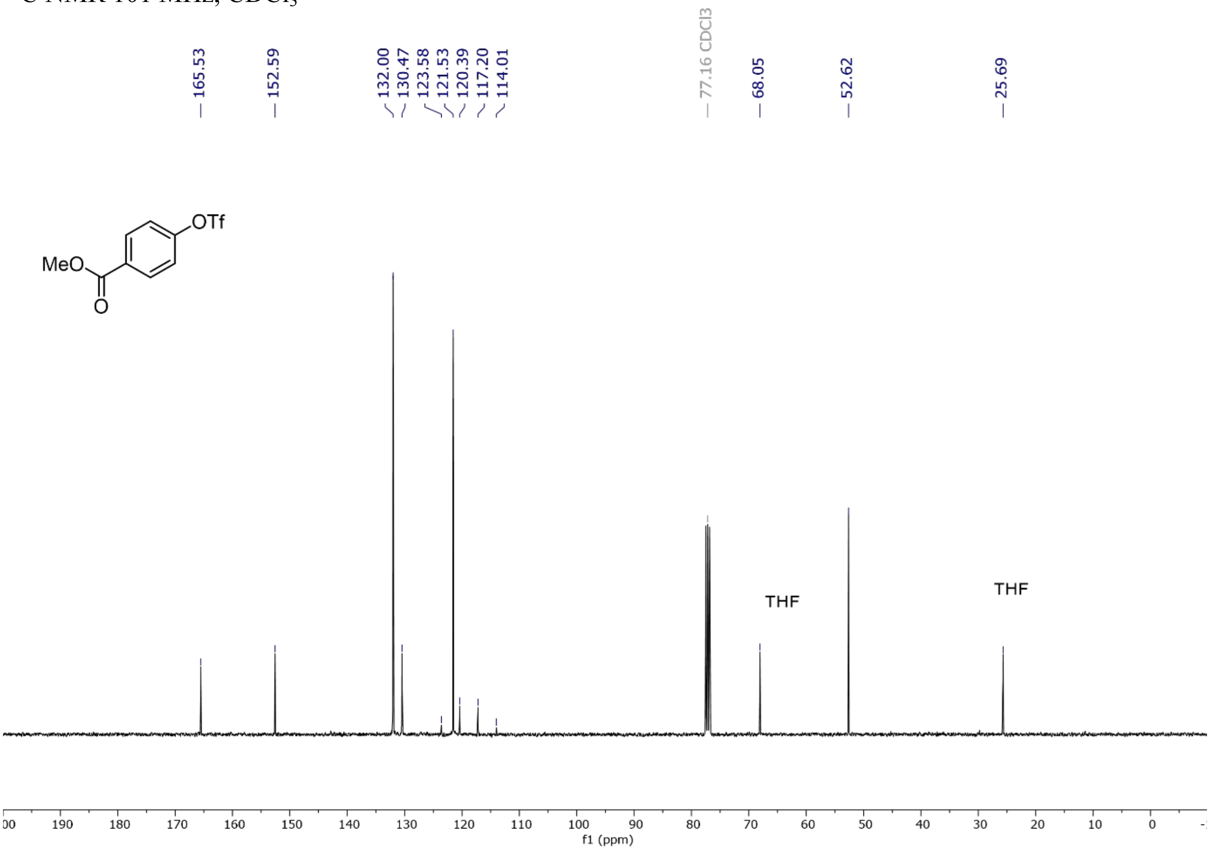

^{19}F NMR 377 MHz, $CDCl_3$

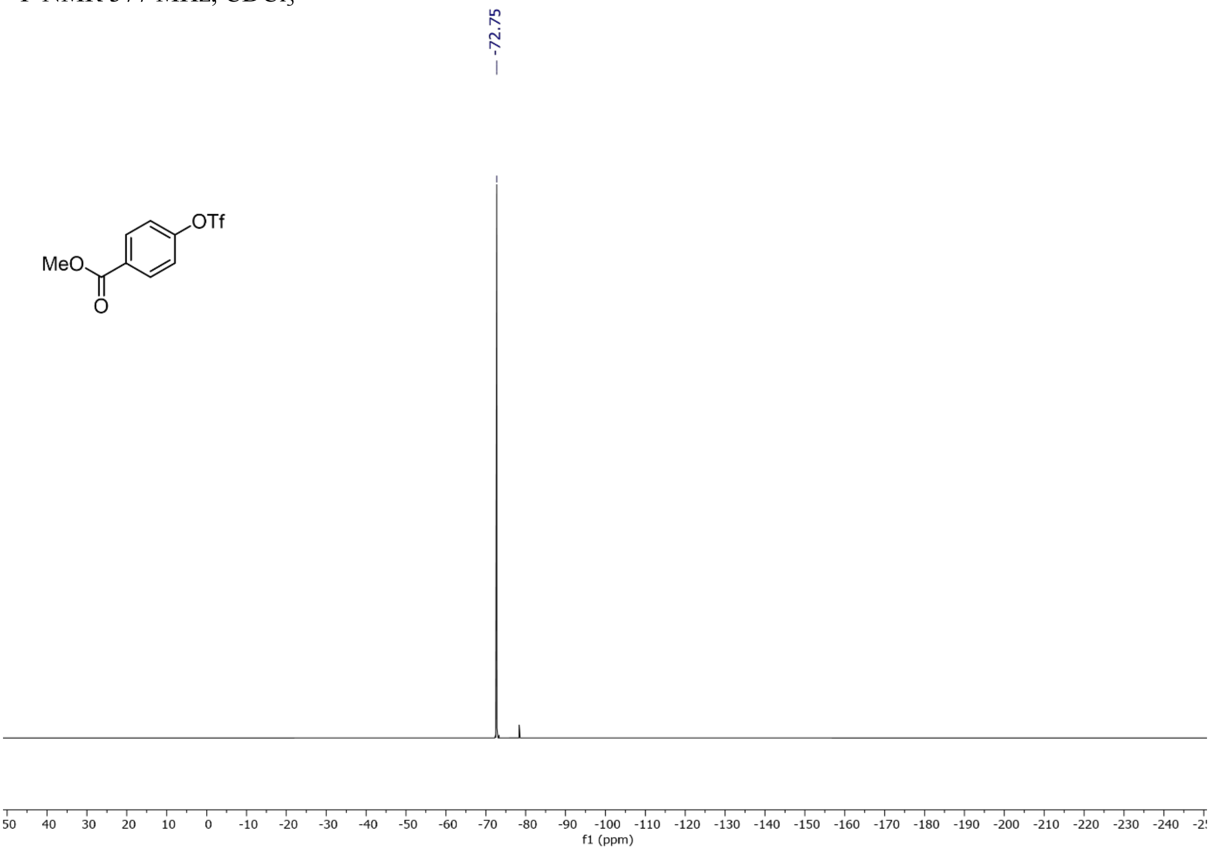

10 References

(1) Bedford, R. B.; Brenner, P. B.; Carter, E.; Gallagher, T.; Murphy, D. M.; Pye, D. R. Iron-Catalyzed Borylation of Alkyl, Allyl, and Aryl Halides: Isolation of an Iron(I) Boryl Complex. *Organometallics* **2014**, *33* (21), 5940–5943.

(2) Iglesias, M.; Beetstra, D. J.; Knight, J. C.; Ooi, L.-L.; Stasch, A.; Coles, S.; Male, L.; Hursthouse, M. B.; Cavell, K. J.; Dervisi, A.; Fallis, I. A. Novel Expanded Ring N-Heterocyclic Carbenes: Free Carbenes, Silver Complexes, And Structures. *Organometallics* **2008**, *27* (13), 3279–3289.

(3) Evans, D. J.; Hitchcock, P. B.; Leigh, G. J.; Nicholson, B. K.; Niedwieski, A. C.; Nunes, F. S.; Soares, J. F. The Synthesis of Triangulo-Trimetal Complexes Containing Both Iron(II) and Vanadium(II). *Inorganica Chim. Acta* **2001**, *319* (1), 147–158.

(4) Dogga, B.; Kumar, C. S. A.; Joseph, J. T. Palladium-Catalyzed Reductive Carbonylation of (Hetero) Aryl Halides and Triflates Using Cobalt Carbonyl as CO Source. *Eur. J. Org. Chem.* **2021**, *2021* (2), 309–313.

(5) Chang, J. W. W.; Chia, E. Y.; Chai, C. L. L.; Seayad, J. Scope of Direct Arylation of Fluorinated Aromatics with Aryl Sulfonates. *Org. Biomol. Chem.* **2012**, *10* (11), 2289–2299.

(6) Muto, K.; Yamaguchi, J.; Itami, K. Nickel-Catalyzed C–H/C–O Coupling of Azoles with Phenol Derivatives. *J. Am. Chem. Soc.* **2012**, *134* (1), 169–172.

(7) Planas, O.; Peciukenas, V.; Cornella, J. Bismuth-Catalyzed Oxidative Coupling of Arylboronic Acids with Triflate and Nonaflate Salts. *J. Am. Chem. Soc.* **2020**, *142* (26), 11382–11387.

(8) Wang, M.; Pang, W. H.; Yuen, O. Y.; Ng, S. S.; So, C. M. Palladium-Catalyzed Deuterodehalogenation of Halogenated Aryl Triflates Using Isopropanol-D8 as the Deuterium Source. *Org. Lett.* **2023**, *25* (47), 8429–8433.

(9) Peng, Z.; Pan, Y.; Xu, B.; Zhang, J. Synthesis and Optical Properties of Novel Unsymmetrical Conjugated Dendrimers. *J. Am. Chem. Soc.* **2000**, *122* (28), 6619–6623.

(10) Greenhalgh, M. D.; Kolodziej, A.; Sinclair, F.; Thomas, S. P. Iron-Catalyzed Hydromagnesiation: Synthesis and Characterization of Benzylic Grignard Reagent Intermediate and Application in the Synthesis of Ibuprofen. *Organometallics* **2014**, *33* (20), 5811–5819.

(11) Brown, M. F.; Che, Y.; Marfat, A.; Melnick, M. J.; Montgomery, J. I.; Reilly, U. N-Linked Hydroxamic Acid Derivatives Useful as Antibacterial Agents. WO2011073845A1, June 23, 2011.

(12) Weimar, M.; Correa da Costa, R.; Lee, F.-H.; Fuchter, M. J. A Scalable and Expedient Route to 1-Aza[6]Helicene Derivatives and Its Subsequent Application to a Chiral-Relay Asymmetric Strategy. *Org. Lett.* **2013**, *15* (7), 1706–1709.

(13) Boehm, P.; Roediger, S.; Bismuto, A.; Morandi, B. Palladium-Catalyzed Chlorocarbonylation of Aryl (Pseudo)Halides Through In Situ Generation of Carbon Monoxide. *Angew. Chem. Int. Ed.* **2020**, *59* (41), 17887–17896.

(14) Zhu, C.; Yamane, M. Transition-Metal-Free Borylation of Aryltriazene Mediated by BF3·OEt2. *Org. Lett.* **2012**, *14* (17), 4560–4563.

(15) Ishiyama, T.; Ishida, K.; Miyaura, N. Synthesis of Pinacol Arylboronates via Cross-Coupling Reaction of Bis(Pinacolato)Diboron with Chloroarenes Catalyzed by Palladium(0)–Tricyclohexylphosphine Complexes. *Tetrahedron* **2001**, *57* (49), 9813–9816.

(16) Labre, F.; Gimbert, Y.; Bannwarth, P.; Olivero, S.; Duñach, E.; Chavant, P. Y. Application of Cooperative Iron/Copper Catalysis to a Palladium-Free Borylation of Aryl Bromides with Pinacolborane. *Org. Lett.* **2014**, *16* (9), 2366–2369. https://doi.org/10.1021/ol500675q.

(17) Fan, Y.; Kang, D. W.; Labalme, S.; Li, J.; Lin, W. Enhanced Energy Transfer in A π-Conjugated Covalent Organic Framework Facilitates Excited-State Nickel Catalysis. *Angew. Chem. Int. Ed.* **2023**, *62* (11), e202218908.

(18) Zhang, J.; Wei, R.; Ren, C.; Liu, L. L.; Wu, L. Si–B Functional Group Exchange Reaction Enabled by a Catalytic Amount of BH3: Scope, Mechanism, and Application. *J. Am. Chem. Soc.* **2023**, *145* (28), 15619–15629.

(19) Kinuta, H.; Tobisu, M.; Chatani, N. Rhodium-Catalyzed Borylation of Aryl 2-Pyridyl Ethers through Cleavage of the Carbon–Oxygen Bond: Borylative Removal of the Directing Group. *J. Am. Chem. Soc.* **2015**, *137* (4), 1593–1600.

(20) Pan, L.; Deckert, M. M.; Cooke, M. V.; Bleeke, A. R.; Laulhé, S. Solvent Anions Enable Photoinduced Borylation and Phosphonation of Aryl Halides via EDA Complexes. *Org. Lett.* **2022**, *24* (35), 6466–6471.

(21) Murai, M.; Nishinaka, N.; Takai, K. Iridium-Catalyzed Sequential Silylation and Borylation of Heteroarenes Based on Regioselective C−H Bond Activation. *Angew. Chem. Int. Ed.* **2018**, *57* (20), 5843–5847.

(22) Wu, S.; Schiel, F.; Melchiorre, P. A General Light-Driven Organocatalytic Platform for the Activation of Inert Substrates. *Angew. Chem. Int. Ed.* **2023**, *62* (32), e202306364.

(23) Pathania, V.; Roy, V. J.; Roy, S. R. Transforming Non-Innocent Phenalenyl to a Potent Photoreductant: Captivating Reductive Functionalization of Aryl Halides through Visible-Light-Induced Electron Transfer Processes. *J. Org. Chem.* **2022**, *87* (24), 16550–16566.

(24) Bhattacherjee, D.; Thakur, V.; Sharma, S.; Kumar, S.; Bharti, R.; Reddy, C. B.; Das, P. Iodine(III)-Promoted Ring Contractive Cyanation of Exocyclic β-Enaminones for the Synthesis of Cyanocyclopentanones. *Adv. Synth. Catal.* **2017**, *359* (13), 2209–2214.

(25) Bhawar, R.; Saini, S.; Nagaraju, D. H.; Bose, S. K. CeO2-Nanorods-Catalyzed Protoboration of Alkenes and Alkynes with Bis(Pinacolato)Diboron. *Adv. Synth. & Catal.* **2023**, *365* (4), 584–593.
